# Thermally activated charge transport in microbial protein nanowires

**DOI:** 10.1038/srep23517

**Published:** 2016-03-24

**Authors:** Sanela Lampa-Pastirk, Joshua P. Veazey, Kathleen A. Walsh, Gustavo T. Feliciano, Rebecca J. Steidl, Stuart H. Tessmer, Gemma Reguera

**Affiliations:** 1 Department of Microbiology and Molecular Genetics, Michigan State University, East Lansing MI 48824, USA; 2 Department of Physics and Astronomy, Michigan State University, East Lansing MI 48824, USA; 3 Departamento de Físico-Química, Instituto de Química, Universidade Estadual Paulista Júlio de Mesquita Filho (UNESP), Nanobionics group, Araraquara, Säo Paulo, Brazil

## Abstract

The bacterium *Geobacter sulfurreducens* requires the expression of conductive protein filaments or pili to respire extracellular electron acceptors such as iron oxides and uranium and to wire electroactive biofilms, but the contribution of the protein fiber to charge transport has remained elusive. Here we demonstrate efficient long-range charge transport along individual pili purified free of metal and redox organic cofactors at rates high enough to satisfy the respiratory rates of the cell. Carrier characteristics were within the orders reported for organic semiconductors (mobility) and inorganic nanowires (concentration), and resistivity was within the lower ranges reported for moderately doped silicon nanowires. However, the pilus conductance and the carrier mobility decreased when one of the tyrosines of the predicted axial multistep hopping path was replaced with an alanine. Furthermore, low temperature scanning tunneling microscopy demonstrated the thermal dependence of the differential conductance at the low voltages that operate in biological systems. The results thus provide evidence for thermally activated multistep hopping as the mechanism that allows *Geobacter* pili to function as protein nanowires between the cell and extracellular electron acceptors.

At the most fundamental level energy transduction in all living systems depends on electron transfer (ET) reactions catalyzed by redox-active proteins, often using metal^1^, flavin^2^ and quinone^3^ cofactors. Amino acids in the protein can also serve as electron donors and acceptors and promote the tunneling of electrons across 20–25 Å distances through potential energy barriers, such as other amino acids^4^,^5^. Distances of sometimes more than 20 Å have been reported to permit successive short and fast hopping reactions in proteins^6^, where the charges (electron or holes) reside for small periods of time in some amino acids or in the peptide backbone and “hop” as they travel distances between donor and acceptor^7^. In enzymes such as oxygenases, dioxygenases, and peroxidases, hole hopping between tyrosines and tryptophans transports potentially oxidizing equivalents away from the active center and toward surface regions to prevent oxidative damage^8^. Charge transport across nanometer distances is also possible in multiprotein redox protein designs, which may align the individual redox components as a nanowire to facilitate intermolecular ET^9^ or use small, diffusible metalloprotein or quinone carriers to shuttle electrons between membrane-associated redox components^10^.

Evidence is also emerging that some bacteria can transport charges at micrometer and even centimeter distances to couple spatially separated redox reactions^11^,^12^,^13^,^14^. Among these microorganisms are metal-reducing bacteria that produce nanowire-like structures to transport electrons beyond the confines of the cell surface^11^,^14^. *Shewanella oneidensis*, for example, produces filamentous extensions of its cytochrome-loaded outer membrane and periplasm^15^, which behave as a microbial redox chain to transport charges at rates^16^ and with mobilities consistent with incoherent redox conductivity^17^. *Geobacter sulfurreducens*, on the other hand, produces a conductive version of a type IV pilus^14^, a protein filament formed through the dynamic assembly and retraction of a peptide subunit (pilin) on the inner membrane^18^. *Geobacter* assembles several pili monolaterally, maximizing interactions with insoluble electron acceptors such as Fe(III) oxides, which are often dispersed and otherwise difficult to reach^14^.

Unlike other bacterial type IV pilins, the structure of the *Geobacter* pilin is divergent and predicted to be optimized for ET^19^. The pilin retains, for example, the conserved α-helix and amino acids required for assembly but has a short, carboxy-terminal (C-t) random coil instead of the β-stranded head of other pilins^19^. The *Geobacter* pilin also contains aromatic residues (tyrosines and phenylalanines) and charged amino acids predicted to distribute the charges and density of states along the peptide to favor charge transport^19^. These predictions are in agreement with the conductivity measured on cell-associated and mechanically-sheared pili^14^,^20^,^21^ and the reduced *in vitro*^22^ and *in vivo*^23^ conductivity of pili carrying mutations in aromatic amino acids. Two incongruent models have been proposed to explain how the pili could transport charges. A metallic model was proposed to explain the metallic-like temperature-dependence of piliated, electrochemically active biofilms and preparations of pili and other proteins, including *c*-cytochromes, dried on gold electrodes^24^. However, *in situ* electrical conductivity measurements on living biofilms grown on an electrode demonstrated the thermal dependence of incoherent redox conductivity^25^. Similarly, a structural model of the pilus refined via molecular dynamics (MD) identified paths of aromatic residues for transversal and axial multistep hopping^23^. However, although some inter-aromatic distances were optimal for π stacking (~3.5 Å), the geometry of the contacts was never of the sandwich type needed for metallic conductance^24^. Furthermore, the aromatic contacts never formed at the same time, as would have been expected in a metal wire^23^.

In the present study, we isolated pili free of metals and organic redox cofactors and used scanning probe methods to evaluate the contribution of the protein fiber to charge transport. We demonstrate efficient charge transport along individual pili and the involvement of aromatic contacts of the predicted axial, multistep hopping path^23^. We also show a temperature effect on the pilus differential tunneling transversal conductance that is consistent with an incoherent hopping model at biological voltages. The implications of these findings for the role of these conductive appendages as biological protein nanowires and their applications in nanoelectronics are discussed.

## Results

### Charge transport along individual pili

**Figure 1:**
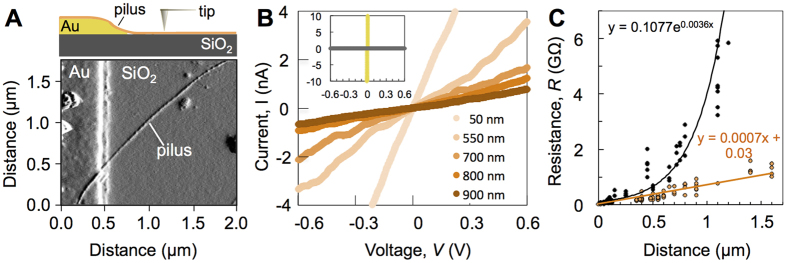
(**A**) Schematic (top) and AFM amplitude image (bottom) of the two-point electronic CP-AFM transport measurement along a pilus filament lying on an insulating SiO_2_ substrate across the edge of a gold electrode (Au). (**B**) Representative *I-V* plots collected with the conductive tip positioned on a pilus at various nm distances from the electrode. Inset, *I-V* control curves on bare gold (yellow) and silicon (gray; 1100 nm) (axes’ units are as in the main *I-V* plot). (**C**) Best correlation (linear or exponential; R^2^ is 0.84 and 0.90 for the WT and Y27A, respectively) between the electrical resistance of the WT (orange) and Y27A (black) pili and the distance of probing.

Individual pili were deposited across the edge of gold electrodes nanofabricated onto an electrically insulating SiO_2_ substrate and the tip of a conductive-probe atomic force microscope (CP-AFM) was positioned on regions of the pilus at various distances from the electrode edge to form the contacts for a two-point electronic transport measurement (Fig. 1A). The current along the pilus was then measured while ramping a bias voltage of ±1 V between the electrode and the conductive tip to acquire current-voltage (*I-V*) plots. Figure 1B illustrates the *I-V* responses for a pilus probed at distances between 50 to 900 nm from the gold edge and the linear fits (R^2^ = 0.98–0.99 for all) obtained within a ±0.6 V voltage range. Additional *I-V* curves are shown in Fig. S1. As controls, we periodically measured the response from the bare gold electrode and the lack of current detected when the tip was positioned on the insulating silica substrate (Fig. 1B, inset). To account for technical and biological variability, we performed transport measurements along 13 pilus fibers purified from four biological cultures and estimated the electrical resistance of the pilus from the linear portion of each of the *I-V* plots.

From the linear resistance-distance correlation (Fig. 1C) we estimated an average resistance value of approximately 730 MΩ along a 1-μm long pilus, with a negligible contribution from the contact resistance (30 ± 8 MΩ; Fig. S2). This resistance value corresponds to an average electron transport rate (~9 × 10^8^ electrons per second at 100 mV) two orders of magnitude higher than the cellular rate of Fe(III) oxide respiration (~9 × 10^6^ electrons per cell per second; Fig. S3). The measured pilus conductivity ranged from 1.4 to 4.3 S/cm, depending on whether the diameter of the immobilized pilus (2 nm height measured by AFM, Fig. S4) or the fiber core modeled in solution (3.5 nm)^23^ was considered. These values are within the orders reported for chemically-fixed membrane protrusions of *S. oneidensis* containing *c*-cytochrome complexes (1 S/cm)^16^, which are also high enough to satisfy the respiratory needs of the cell^26^.

### Contribution of redox cofactors to charge transport

**Figure 2:**
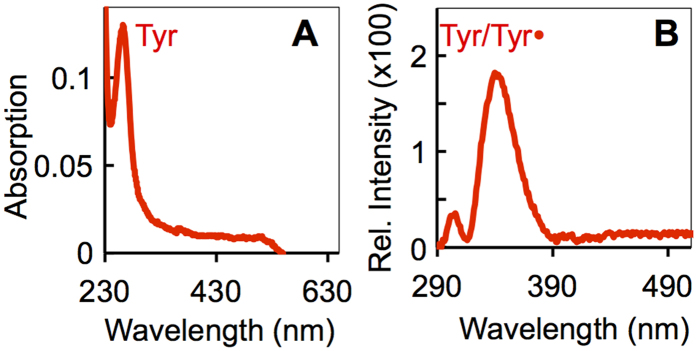
Absorption (**A**) and corrected fluorescence emission (**B**) spectra of the purified pili in a Tris:isopropanol solution showing the tyrosine (Tyr) and tyrosinate (Tyr•) peaks. Spectra from tyrosine, riboflavin, and menaquinone standard solutions are shown in Fig. S5.

The range of resistivity (0.23–0.70 Ω.cm) calculated for the immobilized and solvated pilus (2 and 3.5 nm, respectively) is within the lowest ranges reported for moderately doped Ag and Cu nanowires^27^. However, inductively coupled plasma-atomic emission spectroscopy (ICP-AES) did not detect any metals bound to the pili. The ICP-AES analyses did detect Ca^2+^ (1.7 ± 0.9 atoms per pilin subunit), a cation that is typically detected in pili purified from other bacteria^28^ due to its high affinity for the pilus surface negative charges^29^. Absorption and fluorescence spectroscopy did not detect organic redox cofactors either (Fig. 2). The pili’s absorption spectrum (Fig. 2A) displayed, for example, strong emission below 230 nm and at ~270 nm (peptide bonds and tyrosines, respectively)^30^ but had no peaks in the visible region (at ~360 and ~450 nm) where flavins absorb^31^ (Fig. S5). The Soret band (406 nm) and broad band (528 nm) of oxidized *c*-cytochromes such as OmcS^32^, which has been proposed to associate to the pilus and contribute to ET^33^, was not present either. Similarly, the pili’s fluorescence emission spectrum contained the excitation peaks of tyrosine and its ionized form, tyrosinate (ca. 300 and 340 nm, respectively)^34^, but did not have peaks in the spectral region of quinone and flavin emission (Fig. S5)^35^.

### Contribution of aromatic residues to charge transport

**Figure 3:**
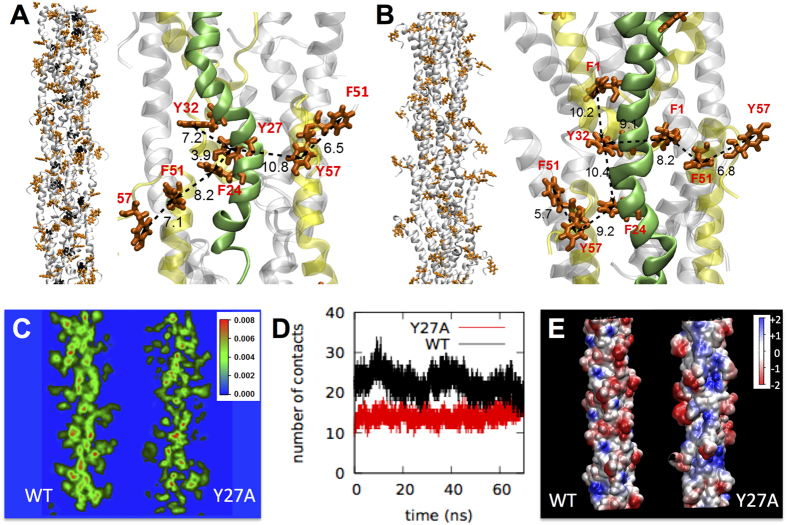
MD simulations of WT and Y27A pili. (**A,B**) Snapshots of the geometry-optimized WT (**A**) and Y27A (**B**) pilus model (black, Y27; orange, other aromatic residues) and detail of aromatic clusters with inter-aromatic distances. (**C,D**) 3D-projection of aromatic density (**C**), aromatic contacts (**D**), and electrostatic surface map (**E**) of WT and Y27A pili.

The MD pilus model revealed a potential axial multistep hopping path involving aromatic residues (tyrosines and phenylalanines)^23^. A snapshot from the MD simulations showing a predicted aromatic path is shown in Fig. 3A, as a reference. Fast rates of charge transport are predicted to require the formation of intramolecular (Y27–F24) and intermolecular (F24–F54) contacts (inter-aromatic distances less than 5 Å)^23^. Consistent with this prediction, replacing Y27 with an alanine (Y27A) maintained inter-aromatic distances (5.7 to 10.4 Å) similar to the wild type (WT) pilus (Fig. 3B), which are optimal for charge hopping^36^, but reduced the number of contacts in the Y27A pilus (Fig. 3C). The Y27A aromatic contacts were also more transient because they formed between F54 and a tyrosine (Y57) of the pilin’s C-t random coil, which fluctuates greatly, and between F1 and Y32 or F51, which are located further apart (8–10 Å) (Fig. 3B). As a result, whereas each WT pilin forms, on average, two or more contacts, Y27A pilins only form 1 to 1.5 contacts, thereby reducing the average number of contacts by almost half throughout the MD simulations (Fig. 3D). Not surprisingly, the electrical resistance (~4 GΩ; 1 μm pilus) of the Y27A pilus increased 5-fold (Fig. 1C and Fig. S6). Furthermore, the pilus conductivity decreased from 4.3 to 0.77 S/cm and its resistivity increased from 0.23 to 1.3 Ω.cm (2-nm diameter).

The conformational arrangements in the Y27A pilus also exposed more positively charged side chains on the pilus surface (Fig. 3E). These regions are smaller and neutralized in the WT pilus, an arrangement predicted to minimize charge trapping^23^. Thus, the exponential increases in Y27A pilus resistance with distance (Fig. 1C) are likely to result from the cumulative scattering effects resulting from the filling of the surface charge traps.

### Carrier mobility and concentration

**Figure 4:**
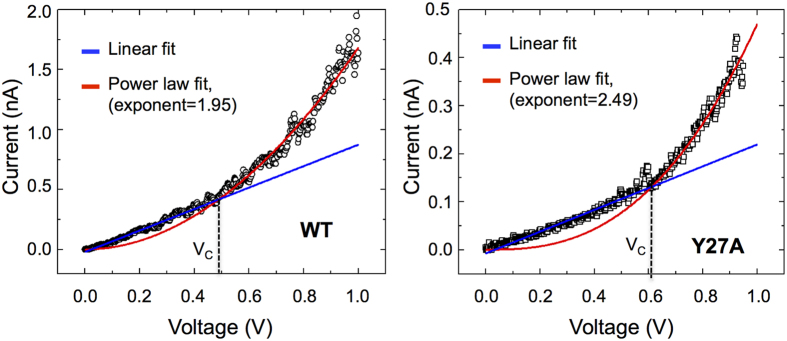
Representative *I-V* curves for the WT (1.6 μm) and Y27A (1.1 μm) pili showing the transition from linear (blue) to a power law fit curve (red) with an exponent close to 2 (red) at the crossover voltage (V_C_).

We screened the *I-V* curves acquired for the WT and Y27A pili to identify those that exhibited nonlinear features at high voltages that could result from space charge limited transport (SCLT), as described in the Supplementary Information. This behavior is dramatically enhanced in thin wires because their large surface-to-volume ratio effectively lowers the carrier concentration at the point of contact with the electrode and a layer of depleted charge forms in the contact region that can be injected at a sufficiently high voltage (the crossover voltage or *V*_*C*_)^37^. As a result, the ohmic (*I* ~ *V*) regime transitions into an electronic transport regime in which *I* ~ *V*^ 2^ 38. Figure 4 shows representative *I-V* curves for the WT and the Y27A pili showing this type of transition. The power-law fit above the *V*_*C*_ follows the equation *I* = *αV*^*b*^, where the exponent *b* is close to 2, as expected for SCLT curves^38^. From the WT scale factor (*a* = 1.7 × 10^−9^ A/V^2^) and the length of probing (1.6 μm) we estimated a carrier mobility of 3.2 × 10^−2^ cm^2^/Vs (see Supplementary Information). This value is small compared to most metal and semiconductor nanowires, including carbon nanotubes, whose mobilities are typically in the tens or hundreds of cm^2^/Vs^39^, but is within the ranges reported for some organic semiconductors such as the hole mobilities of alkyl-substituted polythiophenes (~0.1 cm^2^/Vs)^40^ and the electron mobilities of poly(fluorene)-based polymers (10^−3^–10^−2^ cm^2^/Vs)^41^.

From the fitting parameters (scale factor, 4.8 × 10^−10^ A/V^2^; distance, 1.1 μm) of the Y27A *I-V* curve (Fig. 4) we estimated a carrier mobility (6.3 × 10^−3^ cm^2^/Vs) one order of magnitude lower than the WT, as expected for a pilus with reduced aromatic contacts. By contrast, the effective carrier concentration, calculated as the number of conducting electrons (or holes) per unit volume, was similar in the WT (2 × 10^20^ cm^−3^) and Y27A (2.4 × 10^20^ cm^−3^) pili. This value is high compared to most nanowire systems and organic semiconductors, even after extensive doping. For example, InAs nanowires, which also exhibit SCLT, have carrier concentrations on the order of 10^16^ cm^−3^ 38, whereas the carrier concentration of the *p*-type doped organic semiconductor 2,7-bis(9-carbazolyl)-9,9-spirobifluorene reaches values of approximately 10^18^ cm^−3^ at a dopant concentration of 1 mol %^42^.

### Temperature-dependence of pilus electron energy spectrum

**Figure 5:**
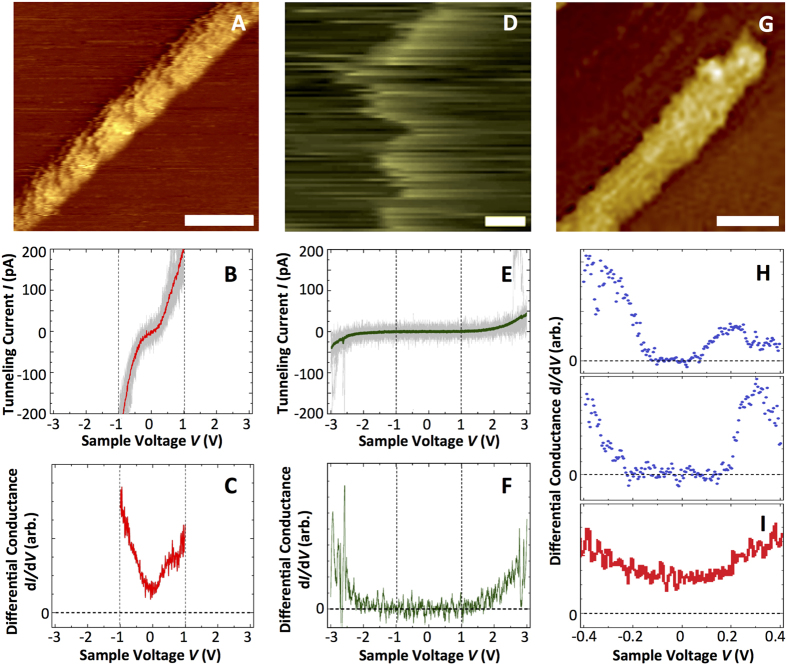
(**A–C**) Room temperature STM of *Geobacter* pili showing a topographic image (0.5 V, 110 pA; bar, 20 nm) (**A**), average *I*-*V* tunneling spectrum (red) of 20 curves (gray) acquired on the center of the pilus (**B**), and differential conductance, *dI/dV*, plot (**C**). (**D–F**) Room temperature STM of PAK pili showing topographic image (3.0 V, 45 pA; bar, 40 nm) (**D**), average *I*-*V* tunneling spectra (green) of 300 curves (20 representative curves are shown in gray) (**E**), and *dI/dV* curve (**F**). (**G,H**) Low-temperature (77 K) STM image of a *Geobacter* pilus (0.5 V, 80 pA; bar, 20 nm) (**G**) and *dI*/*dV* curves (**H**) calculated as the numerical derivative of average *I-V* spectrum obtained from five sequential *I-V* curves at two pilus locations. (**I**) Detail of the room temperature *dI/dV* curve of the *Geobacter* pilus (**C**) within the ±0.4 V voltage range.

We capitalized on the higher spatial resolution of scanning tunneling microscopy (STM) compared to CP-AFM to gain insights into the electronic structure of the WT pili and produce electron energy spectra of pili as a function of temperature. STM images of individual pili deposited on highly oriented pyrolytic graphite (HOPG) showed conducting filaments at room and cryogenic (77 K) temperatures (Fig. 5A,G, respectively), but more periodic molecular sub-structures were resolved at room temperature (Fig. 5A). These features correspond to regions on the pili that supply more tunneling current due to an increase in the local electronic density of states. The room temperature STM scans resolved, for example, 3–4 nm periodic topographic substructures interspersed with deeper 14 nm features (Fig. S7), which match well with the periodic grooves and ridges of type IV pili^43^. Furthermore, from the apparent pilus height (0.8–1.2 nm) and width (8 nm) (Fig. S7), we extrapolated the tip shape-induced convolution and estimated an actual pilus diameter of 4–5 nm, as reported for cell-associated pili by STM^21^ and for the solvated pilus fiber core with its C-t random coil (4.7 nm)^23^. By contrast, pili purified from the bacterium *Pseudomonas aeruginosa* strain K (PAK) could only be imaged at large sample voltages (~3 V) and low tunneling current set points (Fig. 5D). At these high voltages, tip instabilities often arise due to the large electric field between the tip and the sample, which causes distortions and noise in the imaged data.

*I*-*V* measurements taken at fixed locations on the pilus filaments are consistent with conducting behavior of the *Geobacter* pili in the ±1 V voltage range and the insulating behavior of the PAK pili in the ±2 V range (Fig. 5B,E, respectively). Furthermore, the plot of tunneling conductance, *dI*/*dV*, versus tip-sample bias voltage, *V*, revealed clear electronic states at low voltages in the *Geobacter* pili, never reaching zero conductance (Fig. 5C) but a large (~4 V) band gap in the PAK pili (Fig. 5F), which is characteristic of insulators. A band gap feature with zero differential conductance was also revealed in *dI/dV* curves of the *Geobacter* pili when probed at cryogenic (77 K) temperatures (Fig. 5H). As a control, we routinely checked the quality of the conductive tip by alternating *dI/dV* measurements on the pili with tunneling spectroscopy on the graphite substrate several nanometers away from the pili. The width and exact voltage location of the edges of the gap varied with the region of the pilus probed (±0.1–0.2 V), likely due to variations in the density of states at different locations on the pilus, but the gap feature was consistently observed in all. This contrasts with the room temperature *dI*/*dV* of the pili acquired in the same voltage range, which lacks the gap feature (Fig. 5I). The energy gap at 77 K is on the order of hundreds of millivolts; as Boltzmann constant times temperature is only 25 mV at room temperature, simple thermal broadening cannot account for the absence of the gap at room temperature. Rather, the low temperature STM data is consistent with thermally induced redox conductivity.

## Discussion

The results demonstrate that the pilus protein fiber transports charges, consistent with the predicted role of pili as protein nanowires between the cell and external electron acceptors such as Fe(III) oxides,^14^uranium^44^ and matrix-associated *c*-cytochromes in electrochemically active biofilms^23^. Using the average potential (100 mV) that exists between the menaquinone electron carriers of the cell’s inner membrane and the extracellular Fe(III) oxides that serve as electron acceptor^45^, we calculated average electron transport rates along a 1 μm-long pilus (~9 × 10^8^ electrons per second) high enough to support the respiratory rates of the cell during the reduction of Fe(III) oxides (~9 × 10^6^ electrons per cell per second; Fig. S4). The pili probed by CP-AFM were not chemically fixed and retained the topographic and electronic features (Fig. 5 and Fig. S7) of cell-associated pili^21^. However, the adsorption of the pili on the surface prevented any pilus dynamic motions that are predicted to promote electronic coupling and charge transport^23^. This suggests that the *in vivo* pilus charge transport rates could be within the orders of those measured by CP-AFM *in vitro*. As Fe(III) oxides are dispersed in the environment and difficult to access, cells maximize the chances of finding the electron acceptor and disposing respiratory electrons by assembling several pili per cell^14^.

The ability of pili to transport charges over micrometer distances challenges current understanding of protein-mediated electron transfer. The carrier mobility of the WT pilus (3.2 × 10^−2^ cm^2^/Vs) extracted from the discontinuous *I-V* plot (Fig. 4), which gives a measure for how easily the carriers (electrons or holes) move through the pilus, is too low to fit a coherent band conduction mechanism^46^. Furthermore, MD simulations did not reveal aromatic π-stacking^23^, as proposed in the metallic model of pilus conductance^24^. Even with π-π stacking, carrier mobilities far greater than 1 cm^2^/Vs would have been required to describe pilus charge transport according to band theory^46^. The experimental and modeling data support instead an incoherent multistep hopping mechanism, whereby aromatic residues cluster and form the contacts needed to conduct charges along the pilus. Consistent with this model, removing the aromatic ring of Y27 (Y27A pilus), a key relay amino acid in the axial multistep hopping pathway^23^, reduced the aromatic contacts in approximately half (Fig. 3) and decreased the carrier mobility of the pilus (6.3 × 10^−3^ cm^2^/Vs). The mutation also enlarged the regions of positive potential on the pilus surface (Fig. 3E). As the traps in the Y27A fill, they accumulate charges and scattering increases^40^. Scattering effects become more prominent with distance, as more trapped states accumulate, thereby increasing the electrical resistance exponentially as a function of distance (Fig. 1C). Charge trapping is further supported by the discontinuous plots of the Y27A pilus, which showed correlations *I* ~ *V*^*b*^ above the crossover voltage, where the exponent *b* was greater than 2 (~2.5 in the representative plot shown in Fig. 4), as in materials with trapped states^47^. The presence of traps reduces the space-charge-limited current and modifies the shape of the *I-V* plot from an ideal quadratic to a higher power dependence of current on voltage^48^. By contrast, the WT pilus, which has small surface regions of positive potential neutralized to minimize charge trapping^23^, produced discontinuous plots with the pure quadratic scaling of current with voltage above the *V*_*C*_ (Fig. 4) that is typical of SCLT^37^.

The Y27A mutation did not substantially affect the structure of the pilus (Fig. 3) or the number of conducting charges per volume unit (carrier concentration), which was ~10^20^ cm^−3^, as in the WT and within the orders (10^21^ cm^−3^) reported for cell-associated pili after charge injection with a conductive tip and visualization of the propagated charge by electrostatic force microscopy^20^. High carrier concentrations in nanowires are the result, at least partially, of their reduced diameter^38^, which in the pili results from the replacement of the C-terminal globular head of the pilin with a short random coil^23^. Thus, the carrier characteristics of the pili appear to combine features of the organic nature of the conductive material (low mobility) and the nanowire dimensions (high carrier concentration). Yet despite the low carrier mobility, the high carrier concentration delivers a charge transport rate high enough to provide an efficient respiratory strategy.

The diameter of the *Geobacter* pilus estimated from the STM data (4–5 nm) matched well the diameter of the favored MD conformer of the WT pilus in solution (4.7 nm)^23^, which accounts for the volume occupied by the flexible C-t random coil. The detection of tunneling current in the C-t random coils of the pilus fiber is not unexpected because this is the region of the pilin where the terminal tyrosine of the transversal pathway (Y57) is located^23^. This is also the region where anionic ligands are located that bind positively charged electron acceptors, such as the uranyl cation, and position them close to Y57 to facilitate that last step in ET^23^. The helical assembly of the pili also positions the exposed C-t segments helically and confers on the pilus characteristic topographic periodicities, as detected by STM (Fig. S7). Consistent with its unique structural and electronic features, the *Geobacter* pili were transversally conductive in the ±1 V voltage range by STM, and produced differential tunneling conductance plots without a full energy gap at room temperature. By contrast, PAK pili controls were insulating in the ±2 V range (Fig. 5). Furthermore, the cryogenic STM measurements demonstrated the thermal activation of the differential transversal conductance of the *Geobacter* pili at the low (±100–200 mV) voltages that operate in biological systems^45^ (Fig. 5), which is again consistent with incoherent redox conductivity.

The ability of a peptide assembly such as *Geobacter* pili to transport electrons at μm distances is especially significant for nanotechnological applications. Recombinant techniques can be applied to mass-produce pilin derivatives, which can be self-assembled in a cell-free environment using chemical methods^49^. Such approaches could be coupled to genetic engineering to produce protein nanowires with custom functionalities, including chemical tags for selective and specific integration into nanoelectronic devices. This contrasts with fabrication methods for inorganic nanowires, which involve high temperatures, toxic solvents, vacuums, and specialized equipment^50^ and the limited methods available for their functionalization^51^. Protein nanowires also circumvent major concerns regarding cyto- and genotoxicity that limit commercial applications of carbon, metal, and metal-oxide based nanomaterials^52^, making them suitable for the development of biodegradable and biocompatible nanoelectronic devices. Of special significance is the natural ability of *Geobacter* pili to bind and reductively precipitate cationic metal contaminants such as uranium^44^, a process that requires specific amino acid ligands on the pilus surface^23^ and could be harnessed to develop sensors and deployable devices for bioremediation.

## Methods

### Bacterial strains and culture conditions

*G. sulfurreducens* strain PCA and a Y27A mutant strain (constructed as described in the Supplementary Information) were used throughout the study. Cells were routinely grown anaerobically at 30 °C in NB medium^53^ with 30 mM acetate (electron donor) and 40 mM fumarate (electron acceptor) (NBAF). When indicated, cells were grown at 30 °C in fresh water (FW) medium^44^ with 30 mM acetate and 40 mM fumarate (FWAF), transferred to a fresh FWAF culture, and grown for 72 h at 25 °C to induce pili expression, as described elsewhere^14^,^44^. The headspace of the culture vessels was always N_2_:CO_2_ (80:20).

### Pili purification

WT and Y27A pili were isolated as reported elsewhere^44^, except that all the buffers used during the purification contained 1 mM ethylenediaminetetraacetic acid (EDTA) to remove metal impurities and all drying steps were carried out with a constant flow of filter-sterilized N_2_ gas to prevent contamination. The purified pili were incubated in a 2:1 (v/v) chloroform-methanol solution for 2 h at 4 °C to extract any cell-derived quinone contaminants in the chloroform phase^54^ and the methanol phase containing the pili was evaporated with a constant flow of filter-sterilized N_2_ gas. The dry pili samples were stored at −20 °C for short-term use or flash frozen in liquid nitrogen and stored at −80 °C for long-term use. Pili were also purified from *P. aeruginosa* strain K (PAK), as described in the Supplementary Information. Protein concentration in pili samples was determined with the bichinchoninic acid (BCA) assay (Pierce^®^, Thermo Scientific), as previously described^44^.

### Spectroscopy analyses

Three replicate pili samples (20 to 30 μg of pili protein) in 10 mM CHES buffer (pH 9.5, 1 mM EDTA) and buffer controls (no protein) were dispensed in acid-treated glass vessels and subjected to ICP-AES quantitative elemental analysis using a Thermo Jarrell-Ash Enviro 36 Inductively Coupled Argon Plasm (Chemical Analysis Laboratory, University of Georgia, Athens). The number of pilin subunits in each sample was computed for the pili protein values using the predicted pilin’s mass of 6,568.51 Da (or 1.09 × 10^−14^ μg). The concentration (in ppm) of elements was used to extrapolate the number of elemental atoms per pilin.

Purified pili were also suspended in a 1:1 (v/v) solution of 10 mM Tris buffer (pH 7) and isopropanol and their spectral properties were characterized by UV-VIS and fluorescence spectroscopy in reference to l-tyrosine (500 μM), riboflavin (100 μM) and menaquinone (100 μM) standard solutions in Tris:isopropanol (1:1, v/v). Absorption spectra were collected with a Cary100 UV-Vis spectrometer (Varian) set to 2 nm bandpass. Fluorescence spectra were acquired with a QuantaMaster spectrometer (Photon Technology International), with 270 nm excitation and 4 nm bandpass, and corrected with the fluorescence emission baseline from a Tris:isopropanol solution. All spectra were collected at room temperature, in quartz cuvettes with a 1 cm path length (Spectrocell Inc.).

### Conductivity analyses with Scanning Probe Microscopy

Pili were suspended in ddH_2_O and deposited on patterned gold electrodes nanofabricated onto a silicon chip for CP-AFM analysis, as described in the Supplementary Information. The AFM was first operated in tapping mode to identify pili lying across the gold edge. The CP-AFM tip was then positioned on regions of the pilus lying on the silicon substrate at various distances from the gold edge and current-voltage (*I-V*) curves were collected at each position (3 nN force, 1 Hz rate) while applying a bias voltage sweep (±1 V). *I-V* curves were also generated periodically on neighboring regions on the gold electrode and silicon substrate as positive and negative controls, respectively. Methods to calculate resistance values, transport rates, resistivity, conductivity, and carrier characteristics (mobility and concentration) of the pili from the *I-V* curves and cellular rates of Fe(III) oxide reduction for comparative analyses are described in the Supplementary Information.

Scanning Tunneling Microscopy (STM) was used to probe the topography and electronic properties of *Geobacter* and PAK pili after deposition on freshly cleaved HOPG. Information about sample deposition and STM imaging and spectroscopy at room and cryogenic (77 K) temperatures is available in the Supplementary Information.

### MD simulations

All the simulations were performed with the GROMACS software suite^55^,^56^,^57^, using the AMBER99SB force field parameterization^58^. The WT snaphots plot of number of contacts were extracted from the published pilus MD simulations^23^. The Y27A mutation was introduced into the geometry-optimized WT pilus and optimized in a 1-ns MD run, as previously described^23^. The concentration of Na and Cl ions used to neutralize the net charge of the Y27A fiber (−36 *e*) was as in the WT (0.1 M). The atomic coordinates of the Y27A pilus model are provided as a supplemental file (GPIL-Y27A.pdb).

The VMD program^59^ was used to generate maps of electrostatic potential and aromatic density. The latter averaged the atomic locations of the aromatic residues over the 70 ns MD trajectories. Three volumetric slices (xz plane; offset, 0.30, 0.50 and 0.70) of the aromatic density map were extracted for each pilus and overlaid using the Photoshop CS3 image analysis software to generate 3D-projections of the average pilus’ aromatic density distribution. The number of aromatic contacts in the modeled WT and Y27A pili was estimated throughout the 70-ns MD simulations using the routine g_select program of the GROMACS package^60^. The criterion to regard two aromatic residues as a contact was based on the proximity of at least one pair of carbon atoms at a distance of 5 Å or less regardless of the configuration of the dimers.

## Additional Information

**How to cite this article**: Lampa-Pastirk, S. *et al*. Thermally activated charge transport in microbial protein nanowires. *Sci. Rep*. **6**, 23517; doi: 10.1038/srep23517 (2016).

## Supplementary Material

Supplementary Information

Supplementary Data
