# Supplementary material for: Thermally activated charge transport in microbial protein nanowires
Source: Sci Rep. 2016 Mar 24;6:23517. doi: 10.1038/srep23517 (PMC4806346; doi:10.1038/srep23517)
Supplement: Supplementary Information [file srep23517-s1.pdf]

Supplementary Information for

Thermally activated charge transport in microbial protein nanowires

Sanela Lampa-Pastirk, Joshua P. Veazey, Kathleen A. Walsh, Gustavo T. Feliciano, Rebecca J. Steidl, Stuart H. Tessmer, and Gemma Reguera*

* Address correspondence to: reguera@msu.edu

This Supplementary Information includes:

Supplementary methods

Supplementary references

Supplementary figures S1-S7

Supplementary Methods

Construction of a Y27A pilus mutant strain. The Y27A mutation was introduced in several PCR steps using the general procedure and primers reported elsewhere.[1] The *pilA* gene (GSU1496) and upstream region as well as its 397-bp downstream region were first PCR-amplified using primer sets pilASpec F1 and pilASpec R1 and pilASpec F2 and pilASpec R2, respectively. A spectinomycin cassette, PCR-amplified from plasmid pRG5[2] using primer set SpecF and SpecR, was fused to the two other fragments by Overlap Extension PCR (2 min of denaturation at 95°C; 35 cycles of 20 s at 95°C, 25 s at 54°C, and 2 min at 72°C; and, finally, an extension time of 3 min at 72°C) using Herculase II Fusion DNA Polymerase (Agilent Technologies). The PCR product was separated on an agarose gel and purified using the Zymoclean™ Gel DNA Recovery Kit (Zymo Research). The final 1684-bp DNA construct was then cloned into pCR2.1 using the TOPO® TA Cloning kit (Life Technologies) and used as a template for site-directed mutagenesis using the QuikChange Lightning Site-Directed Mutagenesis kit (Agilent Technologies) using primer set pilAY27A-1 and pilAY27A-2. The Y27A mutation was confirmed by sequencing before PCR-amplifying the 1684-bp DNA construct with the external primers and electroporating into electrocompetent cells of *G. sulfurreducens*, prepared as described previously.[3] Selection of recombinant strains carrying the Y27A *pilA* gene was performed on NBAF plates supplemented with 75 μg/ml of spectinomycin.

Purification of PAK pili. PAK pili were purified from cells grown overnight on TBS agar plates at 37°C as a modification of a previously described protocol.[4] Briefly, the cells were scraped off the plate's surface and suspended in standard saline citrate (SSC) buffer (about 1 g of wet cell weight per 10 ml of SSC buffer) before mechanically shearing off the pili and flagella (2-h stirring at 4°C and vortexing 5 times in 1-min cycles). The cells were removed by

centrifugation (8000 x *g*, 20 min) and the pili and flagella were precipitated with 0.5 M NaCl and 1% (w/v) polyethylene glycol 6000 after overnight incubation at 4°C. After centrifugation (6000 x *g*, 25 min), the pili and flagella in the pellets were separated in a 10% (w/v) $(NH_4)_2SO_4$ solution (pH 4.0) for 2 h at 4°C. The supernatant fraction containing the flagella was then removed by centrifugation (6000 x *g* for 15 min. Any remaining flagella in the pili fraction were removed in three sequential steps of $(NH_4)_2SO_4$ precipitation. The purified PAK pili were suspended in PBS, dialyzed for 24 h to remove any remaining $(NH_4)_2SO_4$.

Conductive Probe Atomic Force Microscopy (CP-AFM). For CP-AFM transport measurements, pili purified from *G. sulfurreducens* and resuspended in ddH_2O were deposited on patterned gold electrodes nanofabricated onto a silicon chip. The gold electrode array was fabricated by spin-coating photoresist (Shipley S1813) onto a silicon chip covered by a 300 nm thermal oxide layer (SiO_2). After patterning by masked exposure to ultraviolet light and photoresist development, the electrodes were deposited by sputtering 5 nm of titanium followed by 25 nm of gold onto the surface of the patterned photoresist-covered chip. The photoresist was then removed with acetone and the chip was rinsed with isopropanol and deionized H_2O to leave a clean exposed Au and SiO_2 surface. A solution containing ~ 5 µg/ml of purified pili in ddH_2O was then deposited onto the electrode, allowed to adsorb for 25 min, and then wicked dry with absorbent paper. After pili deposition, the electrodes were stored in an air-tight container under a flow of N_2 gas for 1 h and immediately probed for axial conductivity.

CP-AFM was performed with Ti/Ir-coated silicon cantilevers having nominal spring constant 2 N/m (ASYELEC-01, Asylum Research) using a Cypher scanning probe microscope (Asylum Research). Pilus fibers lying across the gold-SiO_2 interface were first identified in intermittent amplitude modulation (tapping or AC) imaging mode and then probed by CP-AFM

to obtain curves of current (*I*) as a function of the applied voltage (*V*). When indicated, the pili were also imaged in tapping mode to measure the height of the pilus fibers lying on the silica substrate and estimate the pilus diameter (Fig. S4). In this work, the term "amplitude AFM" is used to refer to the amplitude channel (sometimes called the error, gradient, or derivative channel) of the intermittent-contact AFM data. This channel is useful for showing edges of features but does not give quantitative height measurements, so no color scale is provided for those images.

For the *I-V* measurements, the CP-AFM tip was first positioned on a region of the pilus lying on the SiO_2 at a distance from the gold edge and then moved to regions on the pilus closer to the electrode and, finally, on the region of the pilus in direct physical contact with gold (designated as 0 nm). Positive and negative controls were also generated periodically by positioning the tip on the gold electrode or on the SiO_2 substrate, respectively. All *I-V* curves were collected with a 3 nN force and at a rate of 1 Hz using a set of bias voltages within the ± 1 V range. The resistance (*R*) at each position along the pilus fibers was calculated from the slope of the regression line that fit the portion of the *I-V* curve exhibiting the most significant ohmic dependence of current on voltage (usually within the ± 0.5-0.7 V range). When indicated, *I-V* curves were smoothed using the 75 point Savitzky-Golay smoothing function prior to fitting using the IgorPro software (WaveMetrics).

Several pili purified from four WT or two Y27A independent cultures were probed to collect *I-V* curves on several regions per pilus fiber. Each pilus region was probed at several bias voltages (often ±0.1, ±0.5-0.7 and ±0.9-1 V) to account for technical replication. The average resistance, *R*, for each pilus region was calculated from the linear portion of the *I-V* curves. The resistance values calculated for all the regions and pili examined were then plotted as a function

of the distance (pilus length from the point of probing to the gold edge) using the Microsoft Excel software and the best fit (linear for the WT pili and exponential for the Y27A pili, Fig. 1C) was selected based on the trendline correlation coefficient (R^2). The equation obtained for the best fit was used to calculate the average resistance (R, in Ω) of a 1-µm long pilus and this value was used to estimate the current (I, in amps) along the pilus at an applied voltage (V, in volts) of 100 mV using Ohm's law ($I = \frac{V}{R}$). As one amp represents the flow of 1 coulomb of electrical charge per second, we calculated the electron transport rates by multiplying the current value (I) along a 1-µm long pilus by the number of electrons in one coulomb (6.2415×10^{18}).

From the average resistance value of a 1-µm long pilus we calculated the resistivity (ρ, in Ω.cm) of the pilus using the equation:

$$\rho = R\frac{A}{L}$$

where L is the nanowire length (in cm) and A is the cross-sectional area (in cm^2) of a cylindrical pilus fiber.

The conductivity G (in siemens, S, per cm) was calculated as the inverse of the average resistance, as in the formula:

$$G = \frac{1}{R}$$

Scanning Tunneling Microscopy (STM). Dried preparations of *G. sulfurreducens* or PAK pili were suspended in doubly deionized water to a concentration of approximately 5 µg/ml and deposited for 10 min on freshly cleaved highly oriented pyrolytic graphite (HOPG). The

excess liquid was wicked with absorbent paper and the adsorbed sample was rinsed twice with doubly deionized water, leaving the adsorbed sample in contact with the water for 5 min each time. After removing the excess water with absorbent paper the HOPG surface was dried briefly under a stream of N_2 gas. STM images were acquired by raster-scanning the tip at constant sample voltages, as indicated. A feedback circuit allows constant-current operation by adjusting the tip-sample distance during scanning. The apparent width of the pilus fibers in STM was obtained from cross sections and the tip broadening effect was corrected as described for carbon nanotubes[5] and applied to cell-associated pili.[6] The electronic structure of a segment of a pilus fiber imaged by STM was also analyzed to identify structural periodicities in the topography. *I-V* curves were obtained with the tip positioned on the center of the pilus fibers while suspending the feedback and ramping the bias voltage. The differential tunneling conductance, *dI/dV*, was then calculated as the numerical differentiation of *I* with respect to *V,* plotted against the tip-sample bias voltage, *V*.

The cryogenic STM measurements followed the same procedures except that the HOPG with the deposited pili was mounted on a cryogenic-capable STM and the microscope chamber was pumped down to a pressure of a few microtorr and filled with nitrogen gas before cooling down to 77 K by direct immersion in liquid nitrogen.

Calculation of Fe(III) oxide respiratory rates. We used the previously reported[7] rates of Fe(II) accumulation from the reduction of poorly crystalline Fe(III) oxide reduction and cell growth (measured as number of cells from cultures doubling every 15 h) to infer the amount of Fe(II) (in mol) solubilized per cell. The moles of Fe(II) per cell were then used to estimate the electron transport rate per cell shown in Fig. S3 by multiplying the moles of Fe(II) per cell by 6.0221413×10^{23} (Avogadro's number) (the reduction of Fe(III) to Fe(II) is a one-electron

reaction). The electrons exported to the Fe(III) oxides per cell during the logarithmic phase of growth are shown in Fig. S3. From the linear fit of this plot, we calculated transport rates of 9 x 10^6 electrons per cell per second.

Calculation of carrier mobility and concentration. We calculated the carrier mobility (μ) and carrier concentration (n) from *I-V* curves of the WT and Y27A pili exhibiting the transition from ohmic to quadratic electronic transport regimes characteristic of space-charge limited transport (SCLT).[8] Linear behavior with a crossover voltage can also result from two dissimilar Schottky barriers, a behavior that results in curves that cross over from linear behavior at low voltage to exponential regimes at high voltage.[9] In the transport geometry employed in this work, SCLT requires at least one ohmic contact for which the contact resistance is less than the nanowire resistance. The experimental data shows that this is indeed the case, as the average contact resistance (summing over both the pilus-gold and pilus-tip contacts) for individual WT pili was 30 (± 8) MΩ (Fig. S2), whereas the average resistance estimated for a 1-µm long WT pilus was 730 MΩ (and 5 times higher for the Y27A pilus). Furthermore, in the CP-AFM measurements the contacts (pilus-electrode and pilus-CP-AFM tip) are very dissimilar, yet the *I-V* curves are symmetric.[9]

Mobility gives the likelihood for collisions by the charge carriers and is inversely proportional to the average time between collisions. Carrier concentration is the number of conducting electrons (or holes) per unit volume. Both of these contribute to the current (I) and hence determine the electrical resistance ($R=V/I$, where V is voltage) of the material.

The carrier mobility (μ) was extracted from the scale factor of the quadratic portion of *I-V* curves collected at ±1 V with the equation:

$$I = \left(\frac{\mu\pi\varepsilon}{L}\right)V^2$$

where L is the length of the nanowire and ε is the permittivity. This equation applies to high aspect-ratio nanowires in the SCLT regime given by Katzenmeyer *et al.*[9] Solving for mobility, we obtain the following equation:

$$\mu = \frac{IL}{V^2\pi\varepsilon} \quad (1)$$

The effective carrier concentration n was calculated from the crossover voltage V_c, where the ohmic and space-charge-limited current are equal, as described elsewhere.[9] The relationship is:

$$n = \frac{\varepsilon V_c}{eR^2} \quad (2)$$

where e is the electron charge and R is the radius of the nanowire (1 nm).

Supplementary References

1 Feliciano, G. T., Steidl, R. J. & Reguera, G. Structural and functional insights into the conductive pili of *Geobacter sulfurreducens* revealed in molecular dynamics simulations. *Phys. Chem. Chem. Phys.* **17**, 22217-22226, doi:10.1039/C5CP03432A (2015).

2 Kim, B. C. *et al.* OmcF, a putative *c*-type monoheme outer membrane cytochrome required for the expression of other outer membrane cytochromes in *Geobacter sulfurreducens*. *J. Bacteriol.* **187**, 4505-4513 (2005).

3 Coppi, M. V., Leang, C., Sandler, S. J. & Lovley, D. R. Development of a genetic system for *Geobacter sulfurreducens*. *Appl. Environ. Microbiol.* **67**, 3180-3187 (2001).

4 Paranchych, W. *et al.* Biochemical studies on pili isolated from *Pseudomonas aeruginosa* strain PAO. *Can. J. Microbiol.* **25**, 1175-1181 (1979).

5 Biró, L. P. *et al.* Scanning tunnelling microscopy (STM) imaging of carbon nanotubes. *Carbon* **36**, 689-696 (1998).

6 Veazey, J. P., Reguera, G. & Tessmer, S. H. Electronic properties of conductive pili of the metal-reducing bacterium *Geobacter sulfurreducens* probed by scanning tunneling microscopy. *Phys. Rev. E* **84**, 060901, doi:10.1103/PhysRevE.84.060901 (2011).

7 Reguera, G. *et al.* Extracellular electron transfer via microbial nanowires. *Nature* **435**, 1098-1101 (2005).
8 Talin, A. A., Leonard, F., Swartzentruber, B. S., Wang, X. & Hersee, S. D. Unusually strong space-charge-limited current in thin wires. *Phys. Rev. Lett.* **101**, 076802 (2008).
9 Katzenmeyer, A. M. *et al.* Observation of space-charge-limited transport in InAs nanowires. *Ieee T Nanotechnol* **10**, 92-95, doi:Doi 10.1109/Tnano.2010.2062198 (2011).

Supplementary Figures

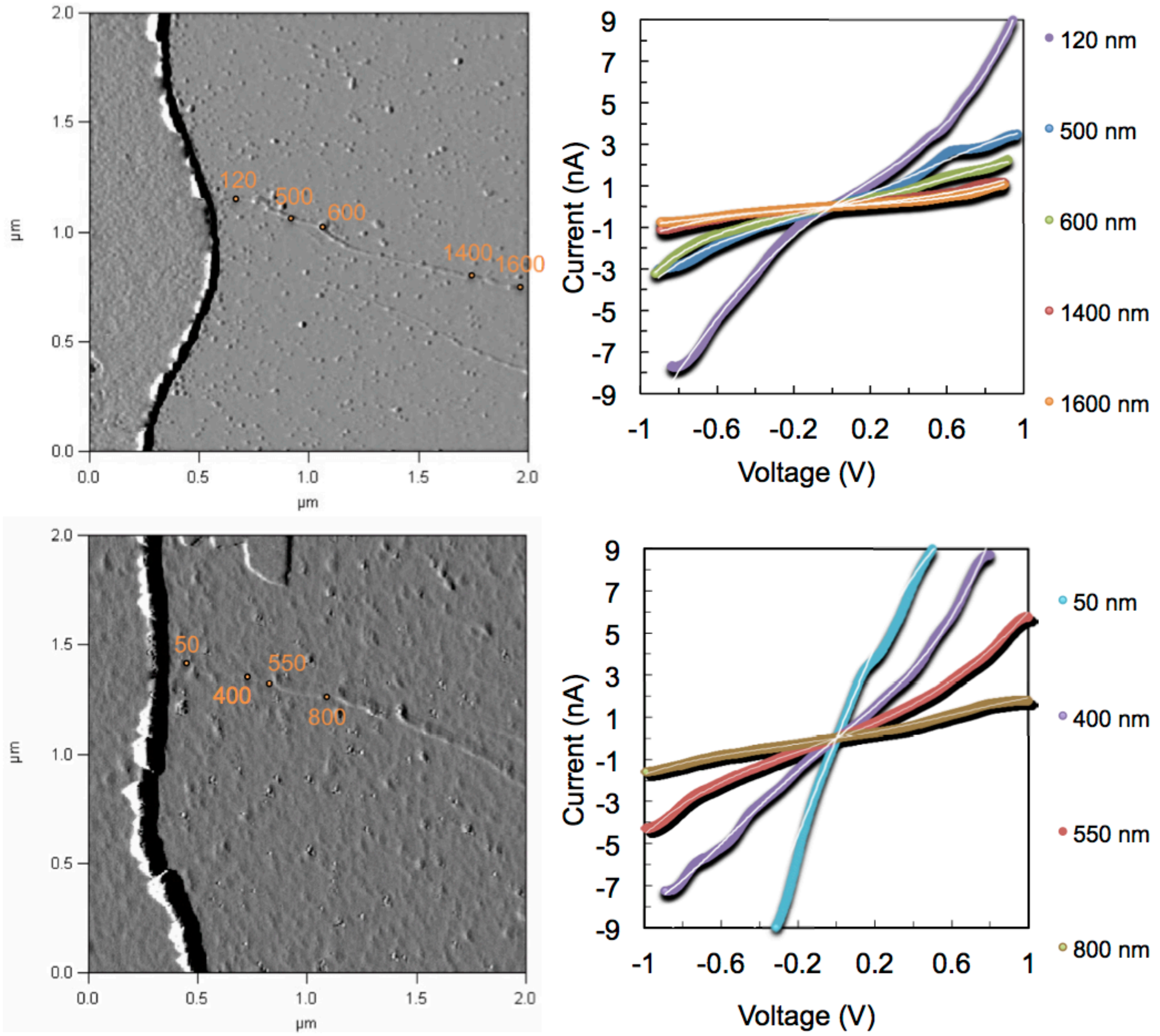

Fig. S1: CP-AFM axial transport measurements of two pilus fibers (top and bottom) deposited across the gold electrode and SiO_2 substrate. The amplitude AFM images on the left indicate the regions of the pilus fibers probed with the conducting tip. The smoothed *I-V* curves generated at ± 0.9-1.0 V bias voltages, their 4th-ordered polynomial fits (all R^2 were greater than 0.98) are shown on the right.

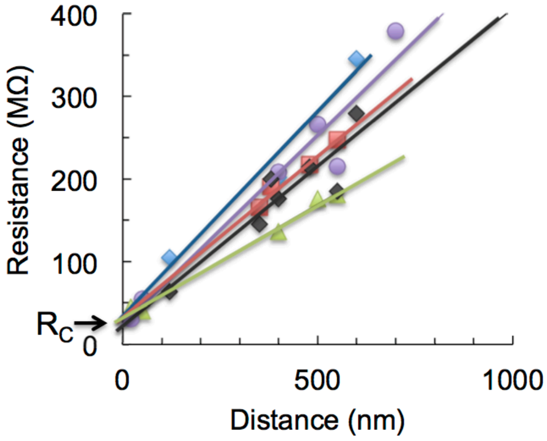

Fig. S2: Contact resistance (R_C) between the pili and the underlying gold electrode extrapolated from the resistance (*R*) values where the linear fit of independent CP-AFM transport measurements on 5 pili crossed the Y axis (zero distance).

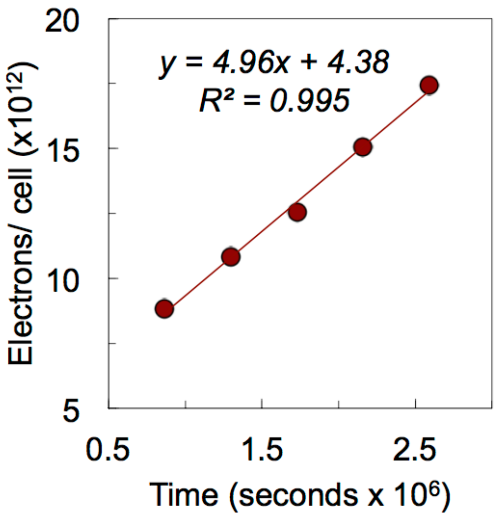

Fig. S3: Electron transport rates per cell during growth of *G. sulfurreducens* with poorly crystalline Fe(III) oxides, calculated from the rates of Fe(II) production and cell growth reported previously.[7]

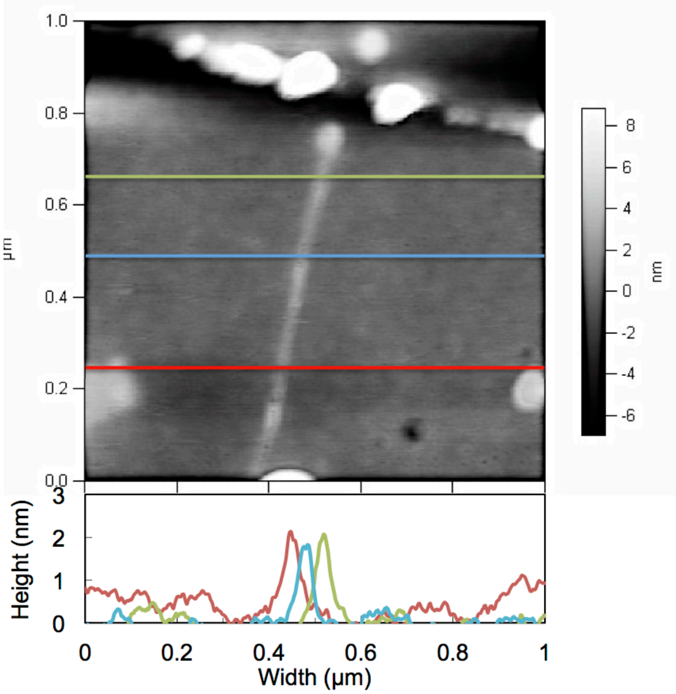

Fig. S4: AFM heights of 2 nm measured at various regions of a WT pilus deposited on the SiO_2 substrate.

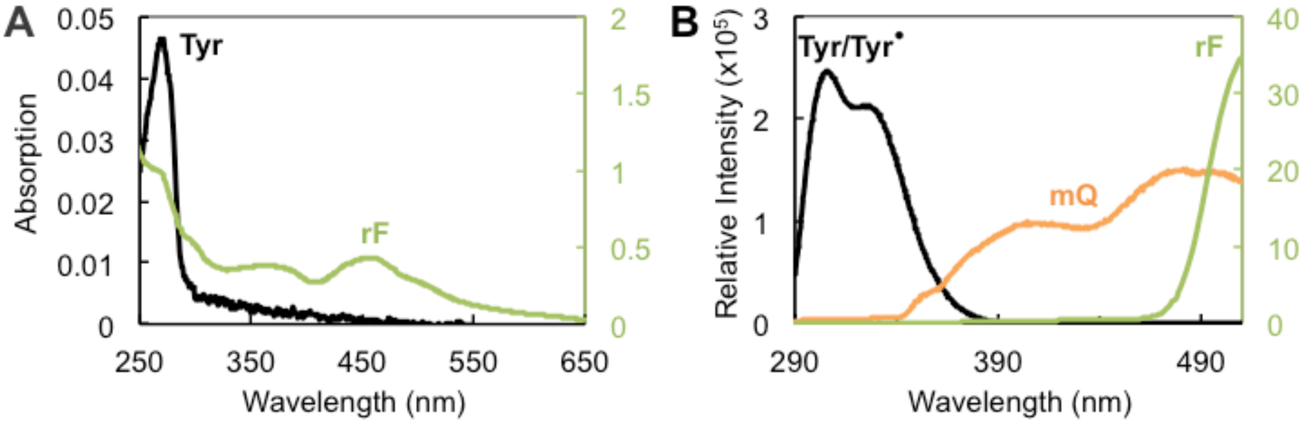

Fig. S5: Absorbance (A) and baseline-corrected relative fluorescence (B) spectra of tyrosine (Tyr), tyrosinate (Tyr•), riboflavin (rF) and menaquinone (mQ) standard solutions in Tris:isopropanol (1:1, v/v).

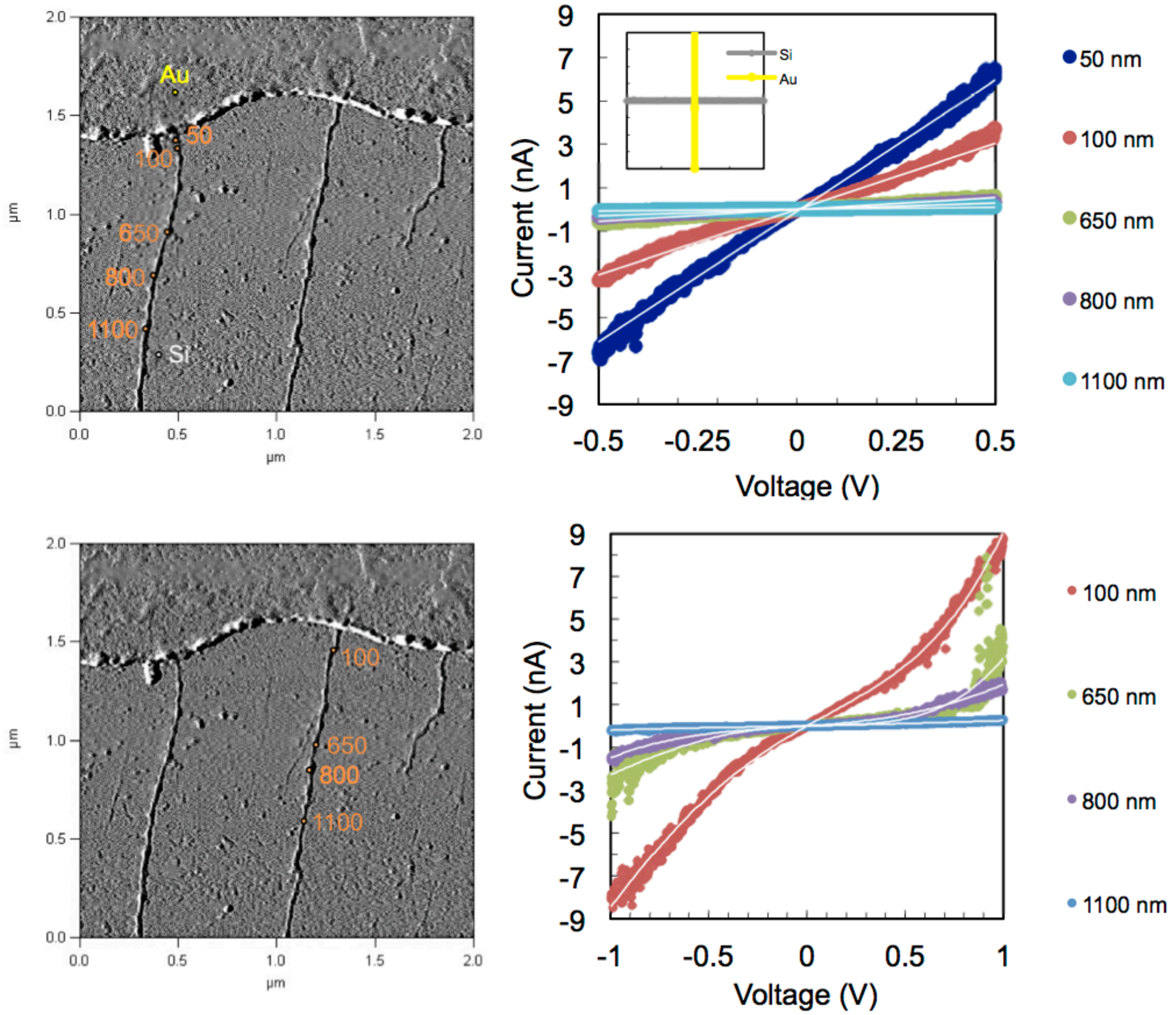

Fig. S6: CP-AFM axial transport measurements of Y27A pili deposited across the gold electrode (Au) and SiO_2 substrate (Si). The AFM amplitude image (left) shows the distance (in nm) along the regions of the pilus fibers probed and the corresponding *I-V* curves (right) generated with a ± 0.5 V (top) or a ± 1 V (bottom) voltage. Also shown are the best fits (linear and polynomial) for the *I-V* plots acquired at ± 0.5 V and ± 1 V, respectively (all R^2 were greater than 0.97).

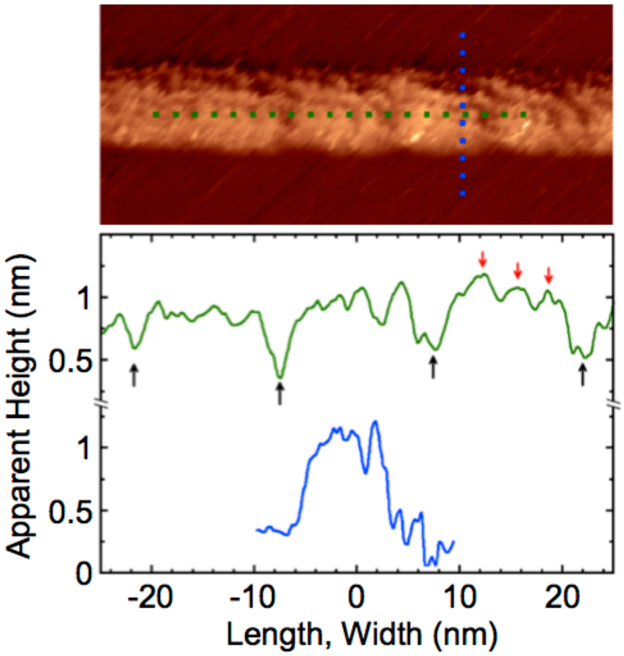

Fig. S7: STM topographical image (top) and height measurements (bottom) of a section of the pilus fiber shown in Figure 5 acquired in constant current mode (0.5V, 100 pA). The height graph shows a large (~ 10 nm) apparent width of the pilus fiber (in blue), due to the distortion caused by the broadening effect of the finite tip diameter. The axial length measurements, in green, show periodic features every 14 nm (black arrows) and topographical peaks every 3-4 nm (red arrows).
