# Supplementary material for: Thermally activated charge transport in microbial protein nanowires
Source: Sci Rep. 2016 Mar 24;6:23517. doi: 10.1038/srep23517 (PMC4806346; doi:10.1038/srep23517)
Supplement: Supplementary Data [file srep23517-s2.doc]

Atomic coordinates of the solvated Y27A pilus optimized via the molecular dynamics simulations.

TITLE: GPIL-Y27A.pdb in water t= 0.00000

REMARK THIS IS A SIMULATION BOX

CRYST1 67.013 74.344 180.000 90.00 90.00 90.00 P 1 1

MODEL 1

ATOM 1 N PHE 1 30.567 39.147 90.698 1.00 0.00

ATOM 2 H1 PHE 1 29.969 39.665 90.070 1.00 0.00

ATOM 3 H2 PHE 1 30.274 39.407 91.630 1.00 0.00

ATOM 4 H3 PHE 1 30.494 38.159 90.498 1.00 0.00

ATOM 5 CA PHE 1 31.968 39.560 90.537 1.00 0.00

ATOM 6 HA PHE 1 32.369 39.203 89.588 1.00 0.00

ATOM 7 CB PHE 1 32.089 41.081 90.497 1.00 0.00

ATOM 8 HB1 PHE 1 31.839 41.434 91.496 1.00 0.00

ATOM 9 HB2 PHE 1 31.337 41.398 89.774 1.00 0.00

ATOM 10 CG PHE 1 33.504 41.569 90.298 1.00 0.00

ATOM 11 CD1 PHE 1 34.074 42.536 91.136 1.00 0.00

ATOM 12 HD1 PHE 1 33.486 42.980 91.926 1.00 0.00

ATOM 13 CE1 PHE 1 35.330 43.101 90.885 1.00 0.00

ATOM 14 HE1 PHE 1 35.706 43.815 91.603 1.00 0.00

ATOM 15 CZ PHE 1 36.026 42.712 89.734 1.00 0.00

ATOM 16 HZ PHE 1 36.983 43.142 89.476 1.00 0.00

ATOM 17 CE2 PHE 1 35.464 41.783 88.851 1.00 0.00

ATOM 18 HE2 PHE 1 35.956 41.516 87.927 1.00 0.00

ATOM 19 CD2 PHE 1 34.220 41.215 89.150 1.00 0.00

ATOM 20 HD2 PHE 1 33.869 40.443 88.481 1.00 0.00

ATOM 21 C PHE 1 32.735 38.861 91.651 1.00 0.00

ATOM 22 O PHE 1 33.197 39.460 92.619 1.00 0.00

ATOM 23 N THR 2 33.007 37.573 91.429 1.00 0.00

ATOM 24 H THR 2 32.672 37.123 90.589 1.00 0.00

ATOM 25 CA THR 2 33.838 36.753 92.287 1.00 0.00

ATOM 26 HA THR 2 34.325 37.395 93.020 1.00 0.00

ATOM 27 CB THR 2 33.004 35.761 93.094 1.00 0.00

ATOM 28 HB THR 2 32.213 36.268 93.646 1.00 0.00

ATOM 29 CG2 THR 2 32.329 34.650 92.295 1.00 0.00

ATOM 30 1HG2 THR 2 33.053 34.236 91.593 1.00 0.00

ATOM 31 2HG2 THR 2 31.488 35.018 91.707 1.00 0.00

ATOM 32 3HG2 THR 2 31.932 33.917 92.997 1.00 0.00

ATOM 33 OG1 THR 2 33.779 35.096 94.066 1.00 0.00

ATOM 34 HG1 THR 2 33.167 34.795 94.742 1.00 0.00

ATOM 35 C THR 2 34.945 36.059 91.506 1.00 0.00

ATOM 36 O THR 2 34.943 36.043 90.277 1.00 0.00

ATOM 37 N LEU 3 35.934 35.535 92.234 1.00 0.00

ATOM 38 H LEU 3 35.944 35.727 93.225 1.00 0.00

ATOM 39 CA LEU 3 36.976 34.655 91.745 1.00 0.00

ATOM 40 HA LEU 3 37.510 35.108 90.911 1.00 0.00

ATOM 41 CB LEU 3 37.957 34.449 92.897 1.00 0.00

ATOM 42 HB1 LEU 3 37.493 33.908 93.722 1.00 0.00

ATOM 43 HB2 LEU 3 38.295 35.409 93.285 1.00 0.00

ATOM 44 CG LEU 3 39.285 33.780 92.557 1.00 0.00

ATOM 45 HG LEU 3 39.079 32.760 92.236 1.00 0.00

ATOM 46 CD1 LEU 3 40.264 34.448 91.594 1.00 0.00

ATOM 47 1HD1 LEU 3 39.747 34.612 90.648 1.00 0.00

ATOM 48 2HD1 LEU 3 41.207 33.915 91.470 1.00 0.00

ATOM 49 3HD1 LEU 3 40.526 35.455 91.916 1.00 0.00

ATOM 50 CD2 LEU 3 40.034 33.522 93.860 1.00 0.00

ATOM 51 1HD2 LEU 3 39.402 32.994 94.560 1.00 0.00

ATOM 52 2HD2 LEU 3 40.425 34.468 94.234 1.00 0.00

ATOM 53 3HD2 LEU 3 40.884 32.856 93.711 1.00 0.00

ATOM 54 C LEU 3 36.478 33.321 91.203 1.00 0.00

ATOM 55 O LEU 3 37.105 32.672 90.370 1.00 0.00

ATOM 56 N ILE 4 35.385 32.797 91.762 1.00 0.00

ATOM 57 H ILE 4 34.833 33.332 92.418 1.00 0.00

ATOM 58 CA ILE 4 34.765 31.535 91.409 1.00 0.00

ATOM 59 HA ILE 4 35.471 30.709 91.477 1.00 0.00

ATOM 60 CB ILE 4 33.613 31.192 92.349 1.00 0.00

ATOM 61 HB ILE 4 32.771 31.883 92.309 1.00 0.00

ATOM 62 CG2 ILE 4 33.043 29.823 91.986 1.00 0.00

ATOM 63 1HG2 ILE 4 32.227 29.623 92.680 1.00 0.00

ATOM 64 2HG2 ILE 4 33.754 28.998 92.032 1.00 0.00

ATOM 65 3HG2 ILE 4 32.585 29.940 91.004 1.00 0.00

ATOM 66 CG1 ILE 4 33.954 31.246 93.835 1.00 0.00

ATOM 67 1HG1 ILE 4 34.248 32.249 94.146 1.00 0.00

ATOM 68 2HG1 ILE 4 33.038 30.890 94.308 1.00 0.00

ATOM 69 CD ILE 4 35.218 30.435 94.103 1.00 0.00

ATOM 70 HD1 ILE 4 35.105 29.457 93.637 1.00 0.00

ATOM 71 HD2 ILE 4 35.207 30.163 95.157 1.00 0.00

ATOM 72 HD3 ILE 4 36.127 31.018 93.942 1.00 0.00

ATOM 73 C ILE 4 34.340 31.425 89.952 1.00 0.00

ATOM 74 O ILE 4 34.631 30.448 89.265 1.00 0.00

ATOM 75 N GLU 5 34.027 32.608 89.420 1.00 0.00

ATOM 76 H GLU 5 33.784 33.270 90.142 1.00 0.00

ATOM 77 CA GLU 5 33.722 32.827 88.020 1.00 0.00

ATOM 78 HA GLU 5 32.922 32.132 87.767 1.00 0.00

ATOM 79 CB GLU 5 33.173 34.233 87.796 1.00 0.00

ATOM 80 HB1 GLU 5 32.971 34.358 86.732 1.00 0.00

ATOM 81 HB2 GLU 5 33.905 34.975 88.114 1.00 0.00

ATOM 82 CG GLU 5 31.833 34.434 88.496 1.00 0.00

ATOM 83 HG1 GLU 5 31.050 34.008 87.868 1.00 0.00

ATOM 84 HG2 GLU 5 31.777 34.038 89.510 1.00 0.00

ATOM 85 CD GLU 5 31.479 35.913 88.569 1.00 0.00

ATOM 86 OE1 GLU 5 31.086 36.435 89.635 1.00 0.00

ATOM 87 OE2 GLU 5 31.654 36.582 87.528 1.00 0.00

ATOM 88 C GLU 5 34.950 32.554 87.163 1.00 0.00

ATOM 89 O GLU 5 34.928 31.754 86.230 1.00 0.00

ATOM 90 N LEU 6 36.091 33.151 87.515 1.00 0.00

ATOM 91 H LEU 6 36.152 33.510 88.457 1.00 0.00

ATOM 92 CA LEU 6 37.301 32.948 86.745 1.00 0.00

ATOM 93 HA LEU 6 37.086 33.153 85.696 1.00 0.00

ATOM 94 CB LEU 6 38.416 33.872 87.224 1.00 0.00

ATOM 95 HB1 LEU 6 39.327 33.560 86.715 1.00 0.00

ATOM 96 HB2 LEU 6 38.645 33.785 88.286 1.00 0.00

ATOM 97 CG LEU 6 38.212 35.345 86.881 1.00 0.00

ATOM 98 HG LEU 6 37.784 35.503 85.891 1.00 0.00

ATOM 99 CD1 LEU 6 37.417 36.236 87.832 1.00 0.00

ATOM 100 1HD1 LEU 6 37.920 36.152 88.796 1.00 0.00

ATOM 101 2HD1 LEU 6 36.418 35.807 87.879 1.00 0.00

ATOM 102 3HD1 LEU 6 37.162 37.177 87.346 1.00 0.00

ATOM 103 CD2 LEU 6 39.606 35.959 86.983 1.00 0.00

ATOM 104 1HD2 LEU 6 39.444 37.028 86.848 1.00 0.00

ATOM 105 2HD2 LEU 6 40.231 35.526 86.202 1.00 0.00

ATOM 106 3HD2 LEU 6 40.119 35.894 87.942 1.00 0.00

ATOM 107 C LEU 6 37.848 31.535 86.900 1.00 0.00

ATOM 108 O LEU 6 38.532 31.066 85.995 1.00 0.00

ATOM 109 N LEU 7 37.371 30.739 87.859 1.00 0.00

ATOM 110 H LEU 7 36.886 31.313 88.532 1.00 0.00

ATOM 111 CA LEU 7 37.725 29.371 88.183 1.00 0.00

ATOM 112 HA LEU 7 38.652 29.121 87.666 1.00 0.00

ATOM 113 CB LEU 7 38.050 29.310 89.672 1.00 0.00

ATOM 114 HB1 LEU 7 38.143 28.270 89.984 1.00 0.00

ATOM 115 HB2 LEU 7 37.195 29.715 90.213 1.00 0.00

ATOM 116 CG LEU 7 39.288 30.047 90.177 1.00 0.00

ATOM 117 HG LEU 7 39.174 31.084 89.860 1.00 0.00

ATOM 118 CD1 LEU 7 39.353 29.980 91.700 1.00 0.00

ATOM 119 1HD1 LEU 7 38.439 30.385 92.136 1.00 0.00

ATOM 120 2HD1 LEU 7 40.193 30.626 91.914 1.00 0.00

ATOM 121 3HD1 LEU 7 39.407 28.939 92.010 1.00 0.00

ATOM 122 CD2 LEU 7 40.586 29.457 89.634 1.00 0.00

ATOM 123 1HD2 LEU 7 41.474 30.072 89.777 1.00 0.00

ATOM 124 2HD2 LEU 7 40.460 29.319 88.560 1.00 0.00

ATOM 125 3HD2 LEU 7 40.723 28.461 90.055 1.00 0.00

ATOM 126 C LEU 7 36.663 28.412 87.666 1.00 0.00

ATOM 127 O LEU 7 36.873 27.207 87.784 1.00 0.00

ATOM 128 N ILE 8 35.689 28.912 86.900 1.00 0.00

ATOM 129 H ILE 8 35.463 29.892 86.984 1.00 0.00

ATOM 130 CA ILE 8 34.731 28.147 86.126 1.00 0.00

ATOM 131 HA ILE 8 34.772 27.103 86.431 1.00 0.00

ATOM 132 CB ILE 8 33.310 28.643 86.383 1.00 0.00

ATOM 133 HB ILE 8 33.320 29.703 86.637 1.00 0.00

ATOM 134 CG2 ILE 8 32.262 28.291 85.330 1.00 0.00

ATOM 135 1HG2 ILE 8 32.250 27.244 85.028 1.00 0.00

ATOM 136 2HG2 ILE 8 32.692 28.739 84.435 1.00 0.00

ATOM 137 3HG2 ILE 8 31.275 28.680 85.580 1.00 0.00

ATOM 138 CG1 ILE 8 32.772 27.929 87.620 1.00 0.00

ATOM 139 1HG1 ILE 8 32.619 26.859 87.487 1.00 0.00

ATOM 140 2HG1 ILE 8 33.543 27.920 88.390 1.00 0.00

ATOM 141 CD ILE 8 31.511 28.387 88.347 1.00 0.00

ATOM 142 HD1 ILE 8 30.624 28.197 87.742 1.00 0.00

ATOM 143 HD2 ILE 8 31.684 29.427 88.626 1.00 0.00

ATOM 144 HD3 ILE 8 31.509 27.810 89.271 1.00 0.00

ATOM 145 C ILE 8 35.071 28.006 84.649 1.00 0.00

ATOM 146 O ILE 8 34.631 27.077 83.975 1.00 0.00

ATOM 147 N VAL 9 35.824 28.964 84.105 1.00 0.00

ATOM 148 H VAL 9 36.036 29.766 84.682 1.00 0.00

ATOM 149 CA VAL 9 36.232 29.048 82.717 1.00 0.00

ATOM 150 HA VAL 9 35.308 29.184 82.155 1.00 0.00

ATOM 151 CB VAL 9 37.204 30.211 82.545 1.00 0.00

ATOM 152 HB VAL 9 38.049 29.976 83.143 1.00 0.00

ATOM 153 CG1 VAL 9 37.853 30.423 81.180 1.00 0.00

ATOM 154 1HG1 VAL 9 37.118 30.342 80.379 1.00 0.00

ATOM 155 2HG1 VAL 9 38.650 29.682 81.117 1.00 0.00

ATOM 156 3HG1 VAL 9 38.276 31.419 81.048 1.00 0.00

ATOM 157 CG2 VAL 9 36.520 31.533 82.878 1.00 0.00

ATOM 158 1HG2 VAL 9 36.049 31.557 83.861 1.00 0.00

ATOM 159 2HG2 VAL 9 35.800 31.758 82.092 1.00 0.00

ATOM 160 3HG2 VAL 9 37.243 32.349 82.891 1.00 0.00

ATOM 161 C VAL 9 36.891 27.810 82.125 1.00 0.00

ATOM 162 O VAL 9 36.629 27.497 80.966 1.00 0.00

ATOM 163 N VAL 10 37.749 27.094 82.856 1.00 0.00

ATOM 164 H VAL 10 37.983 27.501 83.749 1.00 0.00

ATOM 165 CA VAL 10 38.453 25.869 82.534 1.00 0.00

ATOM 166 HA VAL 10 38.781 26.122 81.526 1.00 0.00

ATOM 167 CB VAL 10 39.644 25.673 83.468 1.00 0.00

ATOM 168 HB VAL 10 40.164 24.774 83.137 1.00 0.00

ATOM 169 CG1 VAL 10 40.580 26.874 83.386 1.00 0.00

ATOM 170 1HG1 VAL 10 41.513 26.657 83.894 1.00 0.00

ATOM 171 2HG1 VAL 10 40.028 27.678 83.868 1.00 0.00

ATOM 172 3HG1 VAL 10 40.770 27.045 82.326 1.00 0.00

ATOM 173 CG2 VAL 10 39.215 25.471 84.918 1.00 0.00

ATOM 174 1HG2 VAL 10 40.158 25.415 85.462 1.00 0.00

ATOM 175 2HG2 VAL 10 38.695 24.513 84.944 1.00 0.00

ATOM 176 3HG2 VAL 10 38.590 26.302 85.247 1.00 0.00

ATOM 177 C VAL 10 37.620 24.597 82.455 1.00 0.00

ATOM 178 O VAL 10 38.117 23.560 82.026 1.00 0.00

ATOM 179 N ALA 11 36.349 24.675 82.858 1.00 0.00

ATOM 180 H ALA 11 36.047 25.559 83.238 1.00 0.00

ATOM 181 CA ALA 11 35.303 23.735 82.506 1.00 0.00

ATOM 182 HA ALA 11 35.776 22.785 82.747 1.00 0.00

ATOM 183 CB ALA 11 34.162 23.851 83.511 1.00 0.00

ATOM 184 HB1 ALA 11 34.537 23.997 84.525 1.00 0.00

ATOM 185 HB2 ALA 11 33.454 23.028 83.416 1.00 0.00

ATOM 186 HB3 ALA 11 33.613 24.721 83.153 1.00 0.00

ATOM 187 C ALA 11 34.839 23.752 81.055 1.00 0.00

ATOM 188 O ALA 11 34.432 22.702 80.564 1.00 0.00

ATOM 189 N ILE 12 34.907 24.910 80.394 1.00 0.00

ATOM 190 H ILE 12 35.251 25.706 80.911 1.00 0.00

ATOM 191 CA ILE 12 34.289 25.199 79.116 1.00 0.00

ATOM 192 HA ILE 12 33.881 24.261 78.742 1.00 0.00

ATOM 193 CB ILE 12 33.111 26.138 79.365 1.00 0.00

ATOM 194 HB ILE 12 32.642 26.268 78.390 1.00 0.00

ATOM 195 CG2 ILE 12 32.105 25.593 80.374 1.00 0.00

ATOM 196 1HG2 ILE 12 31.893 24.566 80.078 1.00 0.00

ATOM 197 2HG2 ILE 12 31.261 26.259 80.556 1.00 0.00

ATOM 198 3HG2 ILE 12 32.606 25.625 81.341 1.00 0.00

ATOM 199 CG1 ILE 12 33.503 27.547 79.800 1.00 0.00

ATOM 200 1HG1 ILE 12 33.879 27.508 80.822 1.00 0.00

ATOM 201 2HG1 ILE 12 34.289 27.885 79.125 1.00 0.00

ATOM 202 CD ILE 12 32.325 28.518 79.825 1.00 0.00

ATOM 203 HD1 ILE 12 31.790 28.532 78.875 1.00 0.00

ATOM 204 HD2 ILE 12 32.656 29.548 79.949 1.00 0.00

ATOM 205 HD3 ILE 12 31.624 28.401 80.652 1.00 0.00

ATOM 206 C ILE 12 35.159 25.644 77.949 1.00 0.00

ATOM 207 O ILE 12 34.863 25.428 76.775 1.00 0.00

ATOM 208 N ILE 13 36.248 26.367 78.216 1.00 0.00

ATOM 209 H ILE 13 36.540 26.406 79.182 1.00 0.00

ATOM 210 CA ILE 13 37.100 26.967 77.208 1.00 0.00

ATOM 211 HA ILE 13 36.490 27.667 76.638 1.00 0.00

ATOM 212 CB ILE 13 38.198 27.726 77.946 1.00 0.00

ATOM 213 HB ILE 13 37.788 28.370 78.724 1.00 0.00

ATOM 214 CG2 ILE 13 39.120 26.745 78.667 1.00 0.00

ATOM 215 1HG2 ILE 13 39.711 26.149 77.973 1.00 0.00

ATOM 216 2HG2 ILE 13 38.576 25.993 79.240 1.00 0.00

ATOM 217 3HG2 ILE 13 39.881 27.286 79.228 1.00 0.00

ATOM 218 CG1 ILE 13 38.973 28.664 77.025 1.00 0.00

ATOM 219 1HG1 ILE 13 39.827 29.019 77.602 1.00 0.00

ATOM 220 2HG1 ILE 13 39.431 28.178 76.164 1.00 0.00

ATOM 221 CD ILE 13 38.178 29.934 76.732 1.00 0.00

ATOM 222 HD1 ILE 13 38.906 30.553 76.209 1.00 0.00

ATOM 223 HD2 ILE 13 37.861 30.349 77.689 1.00 0.00

ATOM 224 HD3 ILE 13 37.321 29.803 76.071 1.00 0.00

ATOM 225 C ILE 13 37.636 26.014 76.148 1.00 0.00

ATOM 226 O ILE 13 37.936 26.502 75.061 1.00 0.00

ATOM 227 N GLY 14 37.863 24.712 76.330 1.00 0.00

ATOM 228 H GLY 14 37.633 24.376 77.253 1.00 0.00

ATOM 229 CA GLY 14 38.271 23.779 75.298 1.00 0.00

ATOM 230 HA1 GLY 14 39.128 24.299 74.869 1.00 0.00

ATOM 231 HA2 GLY 14 38.556 22.832 75.756 1.00 0.00

ATOM 232 C GLY 14 37.233 23.599 74.197 1.00 0.00

ATOM 233 O GLY 14 37.515 23.288 73.042 1.00 0.00

ATOM 234 N ILE 15 35.952 23.838 74.479 1.00 0.00

ATOM 235 H ILE 15 35.802 24.066 75.450 1.00 0.00

ATOM 236 CA ILE 15 34.785 23.747 73.622 1.00 0.00

ATOM 237 HA ILE 15 34.984 22.918 72.943 1.00 0.00

ATOM 238 CB ILE 15 33.568 23.367 74.460 1.00 0.00

ATOM 239 HB ILE 15 33.395 24.112 75.237 1.00 0.00

ATOM 240 CG2 ILE 15 32.279 23.234 73.654 1.00 0.00

ATOM 241 1HG2 ILE 15 32.175 22.312 73.082 1.00 0.00

ATOM 242 2HG2 ILE 15 32.156 24.080 72.978 1.00 0.00

ATOM 243 3HG2 ILE 15 31.456 23.273 74.368 1.00 0.00

ATOM 244 CG1 ILE 15 33.742 22.034 75.182 1.00 0.00

ATOM 245 1HG1 ILE 15 33.865 21.225 74.462 1.00 0.00

ATOM 246 2HG1 ILE 15 34.664 22.110 75.757 1.00 0.00

ATOM 247 CD ILE 15 32.716 21.735 76.271 1.00 0.00

ATOM 248 HD1 ILE 15 32.782 22.563 76.976 1.00 0.00

ATOM 249 HD2 ILE 15 32.972 20.825 76.814 1.00 0.00

ATOM 250 HD3 ILE 15 31.673 21.637 75.971 1.00 0.00

ATOM 251 C ILE 15 34.598 25.028 72.822 1.00 0.00

ATOM 252 O ILE 15 34.183 25.000 71.666 1.00 0.00

ATOM 253 N LEU 16 34.831 26.205 73.409 1.00 0.00

ATOM 254 H LEU 16 35.024 26.240 74.400 1.00 0.00

ATOM 255 CA LEU 16 34.511 27.480 72.798 1.00 0.00

ATOM 256 HA LEU 16 33.775 27.329 72.010 1.00 0.00

ATOM 257 CB LEU 16 33.808 28.369 73.819 1.00 0.00

ATOM 258 HB1 LEU 16 33.631 29.371 73.428 1.00 0.00

ATOM 259 HB2 LEU 16 34.557 28.498 74.599 1.00 0.00

ATOM 260 CG LEU 16 32.509 27.848 74.430 1.00 0.00

ATOM 261 HG LEU 16 32.631 26.860 74.874 1.00 0.00

ATOM 262 CD1 LEU 16 32.052 28.770 75.558 1.00 0.00

ATOM 263 1HD1 LEU 16 31.545 29.642 75.146 1.00 0.00

ATOM 264 2HD1 LEU 16 32.854 29.027 76.250 1.00 0.00

ATOM 265 3HD1 LEU 16 31.395 28.218 76.230 1.00 0.00

ATOM 266 CD2 LEU 16 31.408 27.762 73.377 1.00 0.00

ATOM 267 1HD2 LEU 16 31.693 27.107 72.554 1.00 0.00

ATOM 268 2HD2 LEU 16 31.111 28.765 73.071 1.00 0.00

ATOM 269 3HD2 LEU 16 30.508 27.383 73.861 1.00 0.00

ATOM 270 C LEU 16 35.659 28.169 72.072 1.00 0.00

ATOM 271 O LEU 16 35.421 28.804 71.047 1.00 0.00

ATOM 272 N ALA 17 36.875 28.106 72.618 1.00 0.00

ATOM 273 H ALA 17 37.022 27.464 73.382 1.00 0.00

ATOM 274 CA ALA 17 38.067 28.823 72.209 1.00 0.00

ATOM 275 HA ALA 17 37.797 29.405 71.327 1.00 0.00

ATOM 276 CB ALA 17 38.466 29.821 73.292 1.00 0.00

ATOM 277 HB1 ALA 17 39.315 30.409 72.943 1.00 0.00

ATOM 278 HB2 ALA 17 38.650 29.279 74.220 1.00 0.00

ATOM 279 HB3 ALA 17 37.725 30.615 73.384 1.00 0.00

ATOM 280 C ALA 17 39.259 27.970 71.801 1.00 0.00

ATOM 281 O ALA 17 39.924 28.357 70.842 1.00 0.00

ATOM 282 N ALA 18 39.566 26.849 72.457 1.00 0.00

ATOM 283 H ALA 18 38.867 26.443 73.062 1.00 0.00

ATOM 284 CA ALA 18 40.897 26.273 72.427 1.00 0.00

ATOM 285 HA ALA 18 41.521 27.154 72.581 1.00 0.00

ATOM 286 CB ALA 18 41.117 25.268 73.553 1.00 0.00

ATOM 287 HB1 ALA 18 42.154 24.942 73.642 1.00 0.00

ATOM 288 HB2 ALA 18 40.724 25.762 74.441 1.00 0.00

ATOM 289 HB3 ALA 18 40.497 24.413 73.287 1.00 0.00

ATOM 290 C ALA 18 41.354 25.740 71.077 1.00 0.00

ATOM 291 O ALA 18 42.540 25.542 70.822 1.00 0.00

ATOM 292 N ILE 19 40.343 25.422 70.264 1.00 0.00

ATOM 293 H ILE 19 39.437 25.360 70.704 1.00 0.00

ATOM 294 CA ILE 19 40.494 24.978 68.893 1.00 0.00

ATOM 295 HA ILE 19 41.554 24.915 68.652 1.00 0.00

ATOM 296 CB ILE 19 40.136 23.507 68.694 1.00 0.00

ATOM 297 HB ILE 19 40.302 23.346 67.639 1.00 0.00

ATOM 298 CG2 ILE 19 41.018 22.595 69.543 1.00 0.00

ATOM 299 1HG2 ILE 19 40.776 21.533 69.530 1.00 0.00

ATOM 300 2HG2 ILE 19 40.981 22.980 70.562 1.00 0.00

ATOM 301 3HG2 ILE 19 42.050 22.648 69.202 1.00 0.00

ATOM 302 CG1 ILE 19 38.699 23.241 69.131 1.00 0.00

ATOM 303 1HG1 ILE 19 38.608 23.014 70.194 1.00 0.00

ATOM 304 2HG1 ILE 19 38.218 24.189 68.889 1.00 0.00

ATOM 305 CD ILE 19 38.015 22.118 68.356 1.00 0.00

ATOM 306 HD1 ILE 19 36.948 22.206 68.486 1.00 0.00

ATOM 307 HD2 ILE 19 38.281 21.171 68.825 1.00 0.00

ATOM 308 HD3 ILE 19 38.228 22.018 67.294 1.00 0.00

ATOM 309 C ILE 19 39.912 25.898 67.828 1.00 0.00

ATOM 310 O ILE 19 39.586 25.395 66.759 1.00 0.00

ATOM 311 N ALA 20 39.615 27.171 68.097 1.00 0.00

ATOM 312 H ALA 20 39.683 27.471 69.058 1.00 0.00

ATOM 313 CA ALA 20 39.129 28.182 67.177 1.00 0.00

ATOM 314 HA ALA 20 38.381 27.710 66.542 1.00 0.00

ATOM 315 CB ALA 20 38.408 29.351 67.841 1.00 0.00

ATOM 316 HB1 ALA 20 37.485 29.085 68.356 1.00 0.00

ATOM 317 HB2 ALA 20 39.070 29.912 68.500 1.00 0.00

ATOM 318 HB3 ALA 20 38.179 29.973 67.093 1.00 0.00

ATOM 319 C ALA 20 40.162 28.729 66.204 1.00 0.00

ATOM 320 O ALA 20 39.763 28.950 65.063 1.00 0.00

ATOM 321 N ILE 21 41.372 28.997 66.702 1.00 0.00

ATOM 322 H ILE 21 41.621 28.743 67.647 1.00 0.00

ATOM 323 CA ILE 21 42.394 29.578 65.853 1.00 0.00

ATOM 324 HA ILE 21 41.963 30.415 65.304 1.00 0.00

ATOM 325 CB ILE 21 43.538 30.249 66.606 1.00 0.00

ATOM 326 HB ILE 21 44.172 29.505 67.087 1.00 0.00

ATOM 327 CG2 ILE 21 44.423 31.159 65.758 1.00 0.00

ATOM 328 1HG2 ILE 21 44.765 30.594 64.892 1.00 0.00

ATOM 329 2HG2 ILE 21 45.386 31.328 66.240 1.00 0.00

ATOM 330 3HG2 ILE 21 43.931 32.064 65.401 1.00 0.00

ATOM 331 CG1 ILE 21 43.078 31.216 67.693 1.00 0.00

ATOM 332 1HG1 ILE 21 42.420 32.011 67.343 1.00 0.00

ATOM 333 2HG1 ILE 21 42.452 30.632 68.366 1.00 0.00

ATOM 334 CD ILE 21 44.181 31.827 68.553 1.00 0.00

ATOM 335 HD1 ILE 21 44.929 32.406 68.011 1.00 0.00

ATOM 336 HD2 ILE 21 44.575 30.953 69.070 1.00 0.00

ATOM 337 HD3 ILE 21 43.803 32.491 69.330 1.00 0.00

ATOM 338 C ILE 21 43.001 28.641 64.818 1.00 0.00

ATOM 339 O ILE 21 43.206 29.094 63.695 1.00 0.00

ATOM 340 N PRO 22 43.134 27.325 65.000 1.00 0.00

ATOM 341 CD PRO 22 43.293 26.710 66.302 1.00 0.00

ATOM 342 HD1 PRO 22 42.331 26.432 66.733 1.00 0.00

ATOM 343 HD2 PRO 22 43.897 27.133 67.105 1.00 0.00

ATOM 344 CG PRO 22 43.973 25.372 66.029 1.00 0.00

ATOM 345 HG1 PRO 22 43.659 24.661 66.794 1.00 0.00

ATOM 346 HG2 PRO 22 45.042 25.580 66.056 1.00 0.00

ATOM 347 CB PRO 22 43.649 24.966 64.594 1.00 0.00

ATOM 348 HB1 PRO 22 42.715 24.405 64.596 1.00 0.00

ATOM 349 HB2 PRO 22 44.412 24.451 64.010 1.00 0.00

ATOM 350 CA PRO 22 43.477 26.353 63.981 1.00 0.00

ATOM 351 HA PRO 22 44.470 26.587 63.599 1.00 0.00

ATOM 352 C PRO 22 42.400 26.289 62.908 1.00 0.00

ATOM 353 O PRO 22 42.749 26.182 61.735 1.00 0.00

ATOM 354 N GLN 23 41.132 26.287 63.334 1.00 0.00

ATOM 355 H GLN 23 40.849 26.250 64.302 1.00 0.00

ATOM 356 CA GLN 23 40.037 26.233 62.388 1.00 0.00

ATOM 357 HA GLN 23 40.248 25.482 61.627 1.00 0.00

ATOM 358 CB GLN 23 38.694 25.889 63.027 1.00 0.00

ATOM 359 HB1 GLN 23 37.867 26.356 62.493 1.00 0.00

ATOM 360 HB2 GLN 23 38.497 26.350 63.994 1.00 0.00

ATOM 361 CG GLN 23 38.547 24.381 63.210 1.00 0.00

ATOM 362 HG1 GLN 23 38.408 23.906 62.239 1.00 0.00

ATOM 363 HG2 GLN 23 39.390 24.011 63.796 1.00 0.00

ATOM 364 CD GLN 23 37.257 24.061 63.951 1.00 0.00

ATOM 365 OE1 GLN 23 36.308 23.483 63.427 1.00 0.00

ATOM 366 NE2 GLN 23 37.246 24.370 65.249 1.00 0.00

ATOM 367 1HE2 GLN 23 38.043 24.825 65.670 1.00 0.00

ATOM 368 2HE2 GLN 23 36.447 24.161 65.831 1.00 0.00

ATOM 369 C GLN 23 39.906 27.511 61.572 1.00 0.00

ATOM 370 O GLN 23 39.974 27.399 60.350 1.00 0.00

ATOM 371 N PHE 24 39.914 28.697 62.184 1.00 0.00

ATOM 372 H PHE 24 40.118 28.554 63.163 1.00 0.00

ATOM 373 CA PHE 24 40.216 29.932 61.487 1.00 0.00

ATOM 374 HA PHE 24 39.308 30.227 60.961 1.00 0.00

ATOM 375 CB PHE 24 40.579 30.933 62.580 1.00 0.00

ATOM 376 HB1 PHE 24 41.531 30.697 63.056 1.00 0.00

ATOM 377 HB2 PHE 24 39.779 30.917 63.320 1.00 0.00

ATOM 378 CG PHE 24 40.785 32.373 62.177 1.00 0.00

ATOM 379 CD1 PHE 24 40.458 32.858 60.906 1.00 0.00

ATOM 380 HD1 PHE 24 39.829 32.308 60.222 1.00 0.00

ATOM 381 CE1 PHE 24 40.823 34.169 60.571 1.00 0.00

ATOM 382 HE1 PHE 24 40.603 34.586 59.601 1.00 0.00

ATOM 383 CZ PHE 24 41.457 35.056 61.450 1.00 0.00

ATOM 384 HZ PHE 24 41.519 36.116 61.261 1.00 0.00

ATOM 385 CE2 PHE 24 41.772 34.597 62.733 1.00 0.00

ATOM 386 HE2 PHE 24 42.261 35.317 63.373 1.00 0.00

ATOM 387 CD2 PHE 24 41.448 33.266 63.029 1.00 0.00

ATOM 388 HD2 PHE 24 41.714 32.769 63.948 1.00 0.00

ATOM 389 C PHE 24 41.331 29.884 60.452 1.00 0.00

ATOM 390 O PHE 24 41.183 30.324 59.314 1.00 0.00

ATOM 391 N SER 25 42.453 29.269 60.833 1.00 0.00

ATOM 392 H SER 25 42.517 28.811 61.731 1.00 0.00

ATOM 393 CA SER 25 43.587 29.048 59.959 1.00 0.00

ATOM 394 HA SER 25 43.737 30.011 59.473 1.00 0.00

ATOM 395 CB SER 25 44.833 28.692 60.766 1.00 0.00

ATOM 396 HB1 SER 25 45.674 28.643 60.075 1.00 0.00

ATOM 397 HB2 SER 25 44.822 27.683 61.177 1.00 0.00

ATOM 398 OG SER 25 45.136 29.609 61.794 1.00 0.00

ATOM 399 HG SER 25 44.529 29.522 62.533 1.00 0.00

ATOM 400 C SER 25 43.370 28.135 58.760 1.00 0.00

ATOM 401 O SER 25 43.728 28.496 57.641 1.00 0.00

ATOM 402 N ALA 26 42.731 26.980 58.957 1.00 0.00

ATOM 403 H ALA 26 42.604 26.732 59.928 1.00 0.00

ATOM 404 CA ALA 26 42.388 25.985 57.961 1.00 0.00

ATOM 405 HA ALA 26 43.296 25.688 57.436 1.00 0.00

ATOM 406 CB ALA 26 41.834 24.749 58.664 1.00 0.00

ATOM 407 HB1 ALA 26 40.900 24.976 59.178 1.00 0.00

ATOM 408 HB2 ALA 26 41.623 24.063 57.845 1.00 0.00

ATOM 409 HB3 ALA 26 42.562 24.374 59.383 1.00 0.00

ATOM 410 C ALA 26 41.437 26.581 56.931 1.00 0.00

ATOM 411 O ALA 26 41.624 26.687 55.721 1.00 0.00

ATOM 412 N ALA 27 40.413 27.261 57.447 1.00 0.00

ATOM 413 H ALA 27 40.230 27.071 58.422 1.00 0.00

ATOM 414 CA ALA 27 39.429 28.018 56.699 1.00 0.00

ATOM 415 HA ALA 27 38.906 27.377 55.989 1.00 0.00

ATOM 416 CB ALA 27 38.526 28.562 57.801 1.00 0.00

ATOM 417 HB1 ALA 27 38.216 27.680 58.347 1.00 0.00

ATOM 418 HB2 ALA 27 37.630 28.998 57.358 1.00 0.00

ATOM 419 HB3 ALA 27 39.040 29.358 58.340 1.00 0.00

ATOM 420 C ALA 27 39.913 29.200 55.871 1.00 0.00

ATOM 421 O ALA 27 39.266 29.549 54.886 1.00 0.00

ATOM 422 N ARG 28 41.022 29.838 56.251 1.00 0.00

ATOM 423 H ARG 28 41.427 29.537 57.125 1.00 0.00

ATOM 424 CA ARG 28 41.782 30.780 55.452 1.00 0.00

ATOM 425 HA ARG 28 41.052 31.499 55.082 1.00 0.00

ATOM 426 CB ARG 28 42.863 31.530 56.226 1.00 0.00

ATOM 427 HB1 ARG 28 43.619 32.010 55.603 1.00 0.00

ATOM 428 HB2 ARG 28 43.386 30.842 56.889 1.00 0.00

ATOM 429 CG ARG 28 42.328 32.603 57.170 1.00 0.00

ATOM 430 HG1 ARG 28 41.596 33.257 56.698 1.00 0.00

ATOM 431 HG2 ARG 28 41.842 32.269 58.087 1.00 0.00

ATOM 432 CD ARG 28 43.334 33.663 57.607 1.00 0.00

ATOM 433 HD1 ARG 28 43.602 34.397 56.848 1.00 0.00

ATOM 434 HD2 ARG 28 42.843 34.245 58.387 1.00 0.00

ATOM 435 NE ARG 28 44.587 33.055 58.057 1.00 0.00

ATOM 436 HE ARG 28 45.358 32.990 57.407 1.00 0.00

ATOM 437 CZ ARG 28 44.890 32.740 59.323 1.00 0.00

ATOM 438 NH1 ARG 28 44.038 32.845 60.349 1.00 0.00

ATOM 439 1HH1 ARG 28 43.143 33.290 60.198 1.00 0.00

ATOM 440 2HH1 ARG 28 44.430 32.781 61.279 1.00 0.00

ATOM 441 NH2 ARG 28 46.101 32.222 59.563 1.00 0.00

ATOM 442 1HH2 ARG 28 46.723 32.105 58.775 1.00 0.00

ATOM 443 2HH2 ARG 28 46.401 31.918 60.478 1.00 0.00

ATOM 444 C ARG 28 42.406 30.032 54.283 1.00 0.00

ATOM 445 O ARG 28 42.354 30.539 53.165 1.00 0.00

ATOM 446 N VAL 29 42.876 28.797 54.475 1.00 0.00

ATOM 447 H VAL 29 42.892 28.328 55.370 1.00 0.00

ATOM 448 CA VAL 29 43.590 28.203 53.363 1.00 0.00

ATOM 449 HA VAL 29 44.274 28.972 53.002 1.00 0.00

ATOM 450 CB VAL 29 44.409 27.003 53.833 1.00 0.00

ATOM 451 HB VAL 29 43.760 26.160 54.067 1.00 0.00

ATOM 452 CG1 VAL 29 45.434 26.584 52.784 1.00 0.00

ATOM 453 1HG1 VAL 29 46.026 25.675 52.885 1.00 0.00

ATOM 454 2HG1 VAL 29 46.104 27.413 52.560 1.00 0.00

ATOM 455 3HG1 VAL 29 45.027 26.541 51.775 1.00 0.00

ATOM 456 CG2 VAL 29 45.319 27.306 55.019 1.00 0.00

ATOM 457 1HG2 VAL 29 46.136 26.596 55.155 1.00 0.00

ATOM 458 2HG2 VAL 29 44.664 27.226 55.886 1.00 0.00

ATOM 459 3HG2 VAL 29 45.648 28.342 54.965 1.00 0.00

ATOM 460 C VAL 29 42.682 27.772 52.221 1.00 0.00

ATOM 461 O VAL 29 43.011 28.088 51.079 1.00 0.00

ATOM 462 N LYS 30 41.525 27.266 52.652 1.00 0.00

ATOM 463 H LYS 30 41.494 27.175 53.657 1.00 0.00

ATOM 464 CA LYS 30 40.422 26.991 51.754 1.00 0.00

ATOM 465 HA LYS 30 40.899 26.236 51.129 1.00 0.00

ATOM 466 CB LYS 30 39.235 26.441 52.540 1.00 0.00

ATOM 467 HB1 LYS 30 38.352 26.831 52.034 1.00 0.00

ATOM 468 HB2 LYS 30 39.322 26.738 53.585 1.00 0.00

ATOM 469 CG LYS 30 39.254 24.916 52.472 1.00 0.00

ATOM 470 HG1 LYS 30 38.882 24.744 51.462 1.00 0.00

ATOM 471 HG2 LYS 30 40.237 24.483 52.659 1.00 0.00

ATOM 472 CD LYS 30 38.314 24.327 53.519 1.00 0.00

ATOM 473 HD1 LYS 30 38.799 24.643 54.443 1.00 0.00

ATOM 474 HD2 LYS 30 37.331 24.798 53.519 1.00 0.00

ATOM 475 CE LYS 30 38.203 22.808 53.430 1.00 0.00

ATOM 476 HE1 LYS 30 37.675 22.530 52.518 1.00 0.00

ATOM 477 HE2 LYS 30 39.208 22.418 53.272 1.00 0.00

ATOM 478 NZ LYS 30 37.569 22.137 54.576 1.00 0.00

ATOM 479 HZ1 LYS 30 36.571 22.285 54.617 1.00 0.00

ATOM 480 HZ2 LYS 30 38.062 22.390 55.420 1.00 0.00

ATOM 481 HZ3 LYS 30 37.753 21.150 54.471 1.00 0.00

ATOM 482 C LYS 30 40.008 28.226 50.967 1.00 0.00

ATOM 483 O LYS 30 39.702 28.131 49.780 1.00 0.00

ATOM 484 N ALA 31 39.889 29.407 51.576 1.00 0.00

ATOM 485 H ALA 31 40.226 29.477 52.526 1.00 0.00

ATOM 486 CA ALA 31 39.403 30.545 50.821 1.00 0.00

ATOM 487 HA ALA 31 38.553 30.400 50.153 1.00 0.00

ATOM 488 CB ALA 31 39.024 31.553 51.901 1.00 0.00

ATOM 489 HB1 ALA 31 38.167 31.122 52.418 1.00 0.00

ATOM 490 HB2 ALA 31 39.780 31.782 52.652 1.00 0.00

ATOM 491 HB3 ALA 31 38.704 32.447 51.365 1.00 0.00

ATOM 492 C ALA 31 40.462 31.101 49.880 1.00 0.00

ATOM 493 O ALA 31 40.141 31.341 48.718 1.00 0.00

ATOM 494 N TYR 32 41.735 31.147 50.279 1.00 0.00

ATOM 495 H TYR 32 42.015 30.841 51.200 1.00 0.00

ATOM 496 CA TYR 32 42.816 31.544 49.399 1.00 0.00

ATOM 497 HA TYR 32 42.598 32.544 49.023 1.00 0.00

ATOM 498 CB TYR 32 44.093 31.647 50.227 1.00 0.00

ATOM 499 HB1 TYR 32 44.489 30.669 50.503 1.00 0.00

ATOM 500 HB2 TYR 32 43.854 32.203 51.133 1.00 0.00

ATOM 501 CG TYR 32 45.242 32.253 49.456 1.00 0.00

ATOM 502 CD1 TYR 32 45.580 33.560 49.822 1.00 0.00

ATOM 503 HD1 TYR 32 44.975 34.070 50.557 1.00 0.00

ATOM 504 CE1 TYR 32 46.575 34.246 49.114 1.00 0.00

ATOM 505 HE1 TYR 32 46.762 35.285 49.342 1.00 0.00

ATOM 506 CZ TYR 32 47.318 33.539 48.149 1.00 0.00

ATOM 507 OH TYR 32 48.416 34.107 47.578 1.00 0.00

ATOM 508 HH TYR 32 48.351 35.048 47.736 1.00 0.00

ATOM 509 CE2 TYR 32 47.020 32.208 47.794 1.00 0.00

ATOM 510 HE2 TYR 32 47.585 31.687 47.035 1.00 0.00

ATOM 511 CD2 TYR 32 45.925 31.602 48.421 1.00 0.00

ATOM 512 HD2 TYR 32 45.559 30.641 48.090 1.00 0.00

ATOM 513 C TYR 32 42.913 30.627 48.188 1.00 0.00

ATOM 514 O TYR 32 42.862 31.103 47.056 1.00 0.00

ATOM 515 N ASN 33 42.821 29.308 48.369 1.00 0.00

ATOM 516 H ASN 33 42.915 28.927 49.300 1.00 0.00

ATOM 517 CA ASN 33 42.899 28.267 47.363 1.00 0.00

ATOM 518 HA ASN 33 43.893 28.225 46.918 1.00 0.00

ATOM 519 CB ASN 33 42.826 26.937 48.107 1.00 0.00

ATOM 520 HB1 ASN 33 43.853 26.646 48.329 1.00 0.00

ATOM 521 HB2 ASN 33 42.323 26.947 49.074 1.00 0.00

ATOM 522 CG ASN 33 42.139 25.826 47.325 1.00 0.00

ATOM 523 OD1 ASN 33 40.942 25.622 47.513 1.00 0.00

ATOM 524 ND2 ASN 33 42.863 25.054 46.511 1.00 0.00

ATOM 525 1HD2 ASN 33 43.672 25.480 46.082 1.00 0.00

ATOM 526 2HD2 ASN 33 42.497 24.173 46.179 1.00 0.00

ATOM 527 C ASN 33 41.871 28.475 46.260 1.00 0.00

ATOM 528 O ASN 33 42.195 28.548 45.077 1.00 0.00

ATOM 529 N SER 34 40.640 28.696 46.727 1.00 0.00

ATOM 530 H SER 34 40.521 28.578 47.723 1.00 0.00

ATOM 531 CA SER 34 39.482 29.030 45.922 1.00 0.00

ATOM 532 HA SER 34 39.307 28.181 45.264 1.00 0.00

ATOM 533 CB SER 34 38.254 29.191 46.814 1.00 0.00

ATOM 534 HB1 SER 34 37.358 29.303 46.204 1.00 0.00

ATOM 535 HB2 SER 34 38.317 30.160 47.307 1.00 0.00

ATOM 536 OG SER 34 38.060 28.200 47.798 1.00 0.00

ATOM 537 HG SER 34 38.506 28.309 48.643 1.00 0.00

ATOM 538 C SER 34 39.590 30.284 45.069 1.00 0.00

ATOM 539 O SER 34 38.945 30.305 44.022 1.00 0.00

ATOM 540 N ALA 35 40.585 31.128 45.351 1.00 0.00

ATOM 541 H ALA 35 41.128 31.069 46.201 1.00 0.00

ATOM 542 CA ALA 35 40.994 32.164 44.424 1.00 0.00

ATOM 543 HA ALA 35 40.310 32.242 43.580 1.00 0.00

ATOM 544 CB ALA 35 41.008 33.541 45.082 1.00 0.00

ATOM 545 HB1 ALA 35 41.699 33.481 45.923 1.00 0.00

ATOM 546 HB2 ALA 35 41.481 34.289 44.446 1.00 0.00

ATOM 547 HB3 ALA 35 39.988 33.837 45.324 1.00 0.00

ATOM 548 C ALA 35 42.274 31.931 43.634 1.00 0.00

ATOM 549 O ALA 35 42.455 32.531 42.577 1.00 0.00

ATOM 550 N ALA 36 43.161 31.041 44.086 1.00 0.00

ATOM 551 H ALA 36 42.915 30.564 44.941 1.00 0.00

ATOM 552 CA ALA 36 44.562 30.949 43.726 1.00 0.00

ATOM 553 HA ALA 36 45.065 31.843 44.094 1.00 0.00

ATOM 554 CB ALA 36 45.151 29.805 44.546 1.00 0.00

ATOM 555 HB1 ALA 36 44.716 28.835 44.302 1.00 0.00

ATOM 556 HB2 ALA 36 44.931 30.051 45.585 1.00 0.00

ATOM 557 HB3 ALA 36 46.219 29.865 44.336 1.00 0.00

ATOM 558 C ALA 36 44.933 30.810 42.257 1.00 0.00

ATOM 559 O ALA 36 45.879 31.483 41.862 1.00 0.00

ATOM 560 N SER 37 44.207 30.039 41.444 1.00 0.00

ATOM 561 H SER 37 43.339 29.744 41.864 1.00 0.00

ATOM 562 CA SER 37 44.382 29.801 40.024 1.00 0.00

ATOM 563 HA SER 37 45.075 30.573 39.692 1.00 0.00

ATOM 564 CB SER 37 45.138 28.495 39.798 1.00 0.00

ATOM 565 HB1 SER 37 44.571 27.606 40.073 1.00 0.00

ATOM 566 HB2 SER 37 45.362 28.395 38.737 1.00 0.00

ATOM 567 OG SER 37 46.357 28.462 40.506 1.00 0.00

ATOM 568 HG SER 37 46.875 29.269 40.549 1.00 0.00

ATOM 569 C SER 37 43.052 29.852 39.284 1.00 0.00

ATOM 570 O SER 37 42.949 29.395 38.148 1.00 0.00

ATOM 571 N SER 38 42.030 30.432 39.919 1.00 0.00

ATOM 572 H SER 38 42.212 30.824 40.832 1.00 0.00

ATOM 573 CA SER 38 40.675 30.320 39.417 1.00 0.00

ATOM 574 HA SER 38 40.453 29.287 39.149 1.00 0.00

ATOM 575 CB SER 38 39.721 30.737 40.533 1.00 0.00

ATOM 576 HB1 SER 38 38.712 30.839 40.135 1.00 0.00

ATOM 577 HB2 SER 38 40.090 31.651 40.997 1.00 0.00

ATOM 578 OG SER 38 39.745 29.721 41.511 1.00 0.00

ATOM 579 HG SER 38 39.299 29.926 42.337 1.00 0.00

ATOM 580 C SER 38 40.472 31.106 38.130 1.00 0.00

ATOM 581 O SER 38 39.629 30.610 37.385 1.00 0.00

ATOM 582 N ASP 39 41.234 32.166 37.848 1.00 0.00

ATOM 583 H ASP 39 41.917 32.422 38.547 1.00 0.00

ATOM 584 CA ASP 39 41.136 32.899 36.602 1.00 0.00

ATOM 585 HA ASP 39 40.075 32.992 36.373 1.00 0.00

ATOM 586 CB ASP 39 41.770 34.282 36.730 1.00 0.00

ATOM 587 HB1 ASP 39 41.533 34.727 37.696 1.00 0.00

ATOM 588 HB2 ASP 39 42.854 34.199 36.712 1.00 0.00

ATOM 589 CG ASP 39 41.310 35.249 35.649 1.00 0.00

ATOM 590 OD1 ASP 39 40.101 35.556 35.617 1.00 0.00

ATOM 591 OD2 ASP 39 42.174 35.736 34.891 1.00 0.00

ATOM 592 C ASP 39 41.629 32.147 35.373 1.00 0.00

ATOM 593 O ASP 39 41.009 32.068 34.314 1.00 0.00

ATOM 594 N LEU 40 42.743 31.439 35.563 1.00 0.00

ATOM 595 H LEU 40 43.232 31.553 36.440 1.00 0.00

ATOM 596 CA LEU 40 43.369 30.515 34.639 1.00 0.00

ATOM 597 HA LEU 40 43.443 31.002 33.666 1.00 0.00

ATOM 598 CB LEU 40 44.745 30.064 35.119 1.00 0.00

ATOM 599 HB1 LEU 40 44.614 29.464 36.020 1.00 0.00

ATOM 600 HB2 LEU 40 45.390 30.930 35.262 1.00 0.00

ATOM 601 CG LEU 40 45.438 29.187 34.079 1.00 0.00

ATOM 602 HG LEU 40 44.915 28.233 34.055 1.00 0.00

ATOM 603 CD1 LEU 40 45.736 29.751 32.692 1.00 0.00

ATOM 604 1HD1 LEU 40 44.809 30.098 32.237 1.00 0.00

ATOM 605 2HD1 LEU 40 46.296 29.018 32.111 1.00 0.00

ATOM 606 3HD1 LEU 40 46.416 30.594 32.819 1.00 0.00

ATOM 607 CD2 LEU 40 46.798 28.900 34.707 1.00 0.00

ATOM 608 1HD2 LEU 40 47.252 29.886 34.804 1.00 0.00

ATOM 609 2HD2 LEU 40 47.394 28.366 33.968 1.00 0.00

ATOM 610 3HD2 LEU 40 46.705 28.266 35.590 1.00 0.00

ATOM 611 C LEU 40 42.505 29.279 34.433 1.00 0.00

ATOM 612 O LEU 40 42.453 28.805 33.300 1.00 0.00

ATOM 613 N ARG 41 41.823 28.779 35.465 1.00 0.00

ATOM 614 H ARG 41 41.846 29.332 36.310 1.00 0.00

ATOM 615 CA ARG 41 40.945 27.626 35.484 1.00 0.00

ATOM 616 HA ARG 41 41.393 26.810 34.916 1.00 0.00

ATOM 617 CB ARG 41 40.637 27.154 36.902 1.00 0.00

ATOM 618 HB1 ARG 41 39.817 26.444 36.803 1.00 0.00

ATOM 619 HB2 ARG 41 40.238 28.020 37.430 1.00 0.00

ATOM 620 CG ARG 41 41.767 26.490 37.685 1.00 0.00

ATOM 621 HG1 ARG 41 41.708 25.424 37.469 1.00 0.00

ATOM 622 HG2 ARG 41 42.803 26.791 37.526 1.00 0.00

ATOM 623 CD ARG 41 41.502 26.583 39.186 1.00 0.00

ATOM 624 HD1 ARG 41 42.289 26.152 39.804 1.00 0.00

ATOM 625 HD2 ARG 41 41.621 27.638 39.424 1.00 0.00

ATOM 626 NE ARG 41 40.229 26.046 39.668 1.00 0.00

ATOM 627 HE ARG 41 39.522 25.992 38.949 1.00 0.00

ATOM 628 CZ ARG 41 39.891 25.567 40.874 1.00 0.00

ATOM 629 NH1 ARG 41 40.756 25.641 41.894 1.00 0.00

ATOM 630 1HH1 ARG 41 41.694 25.969 41.716 1.00 0.00

ATOM 631 2HH1 ARG 41 40.514 25.315 42.818 1.00 0.00

ATOM 632 NH2 ARG 41 38.680 25.054 41.125 1.00 0.00

ATOM 633 1HH2 ARG 41 38.046 25.026 40.339 1.00 0.00

ATOM 634 2HH2 ARG 41 38.449 24.862 42.089 1.00 0.00

ATOM 635 C ARG 41 39.627 27.850 34.756 1.00 0.00

ATOM 636 O ARG 41 39.259 26.941 34.016 1.00 0.00

ATOM 637 N ASN 42 39.079 29.066 34.820 1.00 0.00

ATOM 638 H ASN 42 39.479 29.707 35.491 1.00 0.00

ATOM 639 CA ASN 42 38.041 29.601 33.964 1.00 0.00

ATOM 640 HA ASN 42 37.187 28.957 34.166 1.00 0.00

ATOM 641 CB ASN 42 37.680 30.951 34.581 1.00 0.00

ATOM 642 HB1 ASN 42 38.533 31.270 35.178 1.00 0.00

ATOM 643 HB2 ASN 42 37.661 31.771 33.866 1.00 0.00

ATOM 644 CG ASN 42 36.479 31.063 35.510 1.00 0.00

ATOM 645 OD1 ASN 42 35.647 30.162 35.585 1.00 0.00

ATOM 646 ND2 ASN 42 36.322 32.207 36.179 1.00 0.00

ATOM 647 1HD2 ASN 42 37.048 32.896 36.029 1.00 0.00

ATOM 648 2HD2 ASN 42 35.437 32.371 36.636 1.00 0.00

ATOM 649 C ASN 42 38.455 29.571 32.498 1.00 0.00

ATOM 650 O ASN 42 37.695 29.085 31.663 1.00 0.00

ATOM 651 N LEU 43 39.656 30.079 32.212 1.00 0.00

ATOM 652 H LEU 43 40.158 30.452 33.006 1.00 0.00

ATOM 653 CA LEU 43 40.208 30.207 30.879 1.00 0.00

ATOM 654 HA LEU 43 39.497 30.781 30.287 1.00 0.00

ATOM 655 CB LEU 43 41.563 30.906 30.936 1.00 0.00

ATOM 656 HB1 LEU 43 42.274 30.333 31.532 1.00 0.00

ATOM 657 HB2 LEU 43 41.348 31.799 31.521 1.00 0.00

ATOM 658 CG LEU 43 42.186 31.413 29.638 1.00 0.00

ATOM 659 HG LEU 43 42.197 30.755 28.770 1.00 0.00

ATOM 660 CD1 LEU 43 41.344 32.530 29.029 1.00 0.00

ATOM 661 1HD1 LEU 43 40.410 32.193 28.579 1.00 0.00

ATOM 662 2HD1 LEU 43 41.895 32.784 28.124 1.00 0.00

ATOM 663 3HD1 LEU 43 41.110 33.276 29.787 1.00 0.00

ATOM 664 CD2 LEU 43 43.607 31.936 29.824 1.00 0.00

ATOM 665 1HD2 LEU 43 44.353 31.157 29.986 1.00 0.00

ATOM 666 2HD2 LEU 43 43.595 32.559 30.713 1.00 0.00

ATOM 667 3HD2 LEU 43 43.813 32.575 28.969 1.00 0.00

ATOM 668 C LEU 43 40.331 28.827 30.249 1.00 0.00

ATOM 669 O LEU 43 39.802 28.514 29.185 1.00 0.00

ATOM 670 N LYS 44 40.965 27.894 30.964 1.00 0.00

ATOM 671 H LYS 44 41.558 28.229 31.709 1.00 0.00

ATOM 672 CA LYS 44 41.062 26.493 30.605 1.00 0.00

ATOM 673 HA LYS 44 41.578 26.315 29.662 1.00 0.00

ATOM 674 CB LYS 44 41.784 25.716 31.703 1.00 0.00

ATOM 675 HB1 LYS 44 41.151 25.579 32.580 1.00 0.00

ATOM 676 HB2 LYS 44 42.722 26.211 31.952 1.00 0.00

ATOM 677 CG LYS 44 42.123 24.297 31.257 1.00 0.00

ATOM 678 HG1 LYS 44 42.534 23.763 32.113 1.00 0.00

ATOM 679 HG2 LYS 44 41.214 23.741 31.026 1.00 0.00

ATOM 680 CD LYS 44 43.042 24.087 30.057 1.00 0.00

ATOM 681 HD1 LYS 44 42.467 24.126 29.132 1.00 0.00

ATOM 682 HD2 LYS 44 43.820 24.849 30.024 1.00 0.00

ATOM 683 CE LYS 44 43.672 22.709 30.240 1.00 0.00

ATOM 684 HE1 LYS 44 44.327 22.675 31.112 1.00 0.00

ATOM 685 HE2 LYS 44 42.888 21.965 30.379 1.00 0.00

ATOM 686 NZ LYS 44 44.320 22.311 28.980 1.00 0.00

ATOM 687 HZ1 LYS 44 43.751 22.227 28.150 1.00 0.00

ATOM 688 HZ2 LYS 44 44.656 21.360 29.030 1.00 0.00

ATOM 689 HZ3 LYS 44 45.130 22.812 28.648 1.00 0.00

ATOM 690 C LYS 44 39.677 25.957 30.271 1.00 0.00

ATOM 691 O LYS 44 39.512 25.214 29.307 1.00 0.00

ATOM 692 N THR 45 38.721 26.175 31.177 1.00 0.00

ATOM 693 H THR 45 39.043 26.756 31.937 1.00 0.00

ATOM 694 CA THR 45 37.451 25.477 31.138 1.00 0.00

ATOM 695 HA THR 45 37.619 24.495 30.695 1.00 0.00

ATOM 696 CB THR 45 36.930 25.068 32.513 1.00 0.00

ATOM 697 HB THR 45 35.985 24.544 32.375 1.00 0.00

ATOM 698 CG2 THR 45 37.825 24.065 33.235 1.00 0.00

ATOM 699 1HG2 THR 45 37.360 23.143 32.887 1.00 0.00

ATOM 700 2HG2 THR 45 37.826 24.164 34.320 1.00 0.00

ATOM 701 3HG2 THR 45 38.850 24.086 32.867 1.00 0.00

ATOM 702 OG1 THR 45 36.734 26.180 33.357 1.00 0.00

ATOM 703 HG1 THR 45 37.581 26.469 33.704 1.00 0.00

ATOM 704 C THR 45 36.431 26.148 30.227 1.00 0.00

ATOM 705 O THR 45 35.459 25.476 29.892 1.00 0.00

ATOM 706 N ALA 46 36.589 27.390 29.765 1.00 0.00

ATOM 707 H ALA 46 37.147 28.031 30.312 1.00 0.00

ATOM 708 CA ALA 46 35.614 28.092 28.955 1.00 0.00

ATOM 709 HA ALA 46 34.868 27.428 28.519 1.00 0.00

ATOM 710 CB ALA 46 34.865 29.096 29.826 1.00 0.00

ATOM 711 HB1 ALA 46 34.497 28.601 30.725 1.00 0.00

ATOM 712 HB2 ALA 46 34.013 29.428 29.232 1.00 0.00

ATOM 713 HB3 ALA 46 35.460 29.971 30.089 1.00 0.00

ATOM 714 C ALA 46 36.241 28.793 27.758 1.00 0.00

ATOM 715 O ALA 46 35.561 29.492 27.011 1.00 0.00

ATOM 716 N LEU 47 37.528 28.536 27.517 1.00 0.00

ATOM 717 H LEU 47 38.024 28.098 28.281 1.00 0.00

ATOM 718 CA LEU 47 38.209 28.795 26.264 1.00 0.00

ATOM 719 HA LEU 47 37.524 29.065 25.461 1.00 0.00

ATOM 720 CB LEU 47 39.163 29.976 26.417 1.00 0.00

ATOM 721 HB1 LEU 47 40.053 29.761 27.007 1.00 0.00

ATOM 722 HB2 LEU 47 38.636 30.778 26.932 1.00 0.00

ATOM 723 CG LEU 47 39.665 30.573 25.105 1.00 0.00

ATOM 724 HG LEU 47 40.377 31.377 25.291 1.00 0.00

ATOM 725 CD1 LEU 47 40.473 29.566 24.293 1.00 0.00

ATOM 726 1HD1 LEU 47 39.760 28.920 23.782 1.00 0.00

ATOM 727 2HD1 LEU 47 41.037 28.857 24.901 1.00 0.00

ATOM 728 3HD1 LEU 47 41.027 30.056 23.492 1.00 0.00

ATOM 729 CD2 LEU 47 38.551 31.164 24.246 1.00 0.00

ATOM 730 1HD2 LEU 47 38.131 32.066 24.692 1.00 0.00

ATOM 731 2HD2 LEU 47 37.832 30.383 23.997 1.00 0.00

ATOM 732 3HD2 LEU 47 39.038 31.350 23.290 1.00 0.00

ATOM 733 C LEU 47 38.859 27.496 25.810 1.00 0.00

ATOM 734 O LEU 47 38.390 26.910 24.836 1.00 0.00

ATOM 735 N GLU 48 39.944 27.013 26.420 1.00 0.00

ATOM 736 H GLU 48 40.154 27.436 27.312 1.00 0.00

ATOM 737 CA GLU 48 40.904 26.129 25.790 1.00 0.00

ATOM 738 HA GLU 48 41.313 26.528 24.862 1.00 0.00

ATOM 739 CB GLU 48 42.089 25.808 26.695 1.00 0.00

ATOM 740 HB1 GLU 48 42.601 24.963 26.234 1.00 0.00

ATOM 741 HB2 GLU 48 41.553 25.478 27.584 1.00 0.00

ATOM 742 CG GLU 48 43.002 27.005 26.953 1.00 0.00

ATOM 743 HG1 GLU 48 42.479 27.940 27.153 1.00 0.00

ATOM 744 HG2 GLU 48 43.657 26.788 27.797 1.00 0.00

ATOM 745 CD GLU 48 43.806 27.490 25.754 1.00 0.00

ATOM 746 OE1 GLU 48 43.601 27.047 24.604 1.00 0.00

ATOM 747 OE2 GLU 48 44.677 28.370 25.922 1.00 0.00

ATOM 748 C GLU 48 40.273 24.786 25.452 1.00 0.00

ATOM 749 O GLU 48 40.479 24.294 24.345 1.00 0.00

ATOM 750 N SER 49 39.456 24.259 26.367 1.00 0.00

ATOM 751 H SER 49 39.255 24.674 27.265 1.00 0.00

ATOM 752 CA SER 49 38.751 22.996 26.266 1.00 0.00

ATOM 753 HA SER 49 39.509 22.214 26.262 1.00 0.00

ATOM 754 CB SER 49 37.826 22.740 27.452 1.00 0.00

ATOM 755 HB1 SER 49 38.441 22.808 28.350 1.00 0.00

ATOM 756 HB2 SER 49 37.426 21.726 27.449 1.00 0.00

ATOM 757 OG SER 49 36.856 23.759 27.540 1.00 0.00

ATOM 758 HG SER 49 36.364 23.710 28.363 1.00 0.00

ATOM 759 C SER 49 37.889 22.834 25.022 1.00 0.00

ATOM 760 O SER 49 37.803 21.779 24.398 1.00 0.00

ATOM 761 N ALA 50 37.332 23.953 24.552 1.00 0.00

ATOM 762 H ALA 50 37.382 24.768 25.146 1.00 0.00

ATOM 763 CA ALA 50 36.580 23.980 23.314 1.00 0.00

ATOM 764 HA ALA 50 35.873 23.170 23.318 1.00 0.00

ATOM 765 CB ALA 50 35.677 25.209 23.334 1.00 0.00

ATOM 766 HB1 ALA 50 35.074 25.250 22.427 1.00 0.00

ATOM 767 HB2 ALA 50 35.057 25.170 24.229 1.00 0.00

ATOM 768 HB3 ALA 50 36.227 26.145 23.429 1.00 0.00

ATOM 769 C ALA 50 37.398 23.850 22.036 1.00 0.00

ATOM 770 O ALA 50 37.016 23.114 21.129 1.00 0.00

ATOM 771 N PHE 51 38.568 24.491 22.027 1.00 0.00

ATOM 772 H PHE 51 38.934 24.848 22.898 1.00 0.00

ATOM 773 CA PHE 51 39.537 24.393 20.954 1.00 0.00

ATOM 774 HA PHE 51 38.954 24.564 20.049 1.00 0.00

ATOM 775 CB PHE 51 40.551 25.520 21.134 1.00 0.00

ATOM 776 HB1 PHE 51 41.484 25.390 20.590 1.00 0.00

ATOM 777 HB2 PHE 51 40.862 25.700 22.163 1.00 0.00

ATOM 778 CG PHE 51 40.019 26.830 20.605 1.00 0.00

ATOM 779 CD1 PHE 51 39.262 27.644 21.457 1.00 0.00

ATOM 780 HD1 PHE 51 38.925 27.306 22.425 1.00 0.00

ATOM 781 CE1 PHE 51 38.623 28.803 21.001 1.00 0.00

ATOM 782 HE1 PHE 51 38.010 29.428 21.633 1.00 0.00

ATOM 783 CZ PHE 51 38.964 29.346 19.757 1.00 0.00

ATOM 784 HZ PHE 51 38.551 30.298 19.456 1.00 0.00

ATOM 785 CE2 PHE 51 39.761 28.567 18.909 1.00 0.00

ATOM 786 HE2 PHE 51 39.876 28.945 17.904 1.00 0.00

ATOM 787 CD2 PHE 51 40.250 27.318 19.314 1.00 0.00

ATOM 788 HD2 PHE 51 40.809 26.671 18.655 1.00 0.00

ATOM 789 C PHE 51 40.170 23.009 20.884 1.00 0.00

ATOM 790 O PHE 51 40.602 22.520 19.843 1.00 0.00

ATOM 791 N ALA 52 40.413 22.368 22.028 1.00 0.00

ATOM 792 H ALA 52 40.162 22.980 22.790 1.00 0.00

ATOM 793 CA ALA 52 40.981 21.041 22.168 1.00 0.00

ATOM 794 HA ALA 52 41.928 21.024 21.627 1.00 0.00

ATOM 795 CB ALA 52 41.250 20.821 23.653 1.00 0.00

ATOM 796 HB1 ALA 52 41.881 19.934 23.678 1.00 0.00

ATOM 797 HB2 ALA 52 41.748 21.649 24.158 1.00 0.00

ATOM 798 HB3 ALA 52 40.408 20.436 24.228 1.00 0.00

ATOM 799 C ALA 52 40.014 19.956 21.716 1.00 0.00

ATOM 800 O ALA 52 40.398 18.980 21.075 1.00 0.00

ATOM 801 N ASP 53 38.722 20.119 22.012 1.00 0.00

ATOM 802 H ASP 53 38.437 20.872 22.622 1.00 0.00

ATOM 803 CA ASP 53 37.676 19.286 21.455 1.00 0.00

ATOM 804 HA ASP 53 37.874 18.241 21.695 1.00 0.00

ATOM 805 CB ASP 53 36.368 19.639 22.158 1.00 0.00

ATOM 806 HB1 ASP 53 36.492 19.545 23.236 1.00 0.00

ATOM 807 HB2 ASP 53 36.094 20.692 22.113 1.00 0.00

ATOM 808 CG ASP 53 35.197 18.798 21.670 1.00 0.00

ATOM 809 OD1 ASP 53 34.366 19.450 21.009 1.00 0.00

ATOM 810 OD2 ASP 53 35.146 17.579 21.942 1.00 0.00

ATOM 811 C ASP 53 37.461 19.349 19.948 1.00 0.00

ATOM 812 O ASP 53 37.123 18.349 19.321 1.00 0.00

ATOM 813 N ASP 54 37.662 20.487 19.282 1.00 0.00

ATOM 814 H ASP 54 37.880 21.285 19.862 1.00 0.00

ATOM 815 CA ASP 54 37.605 20.625 17.839 1.00 0.00

ATOM 816 HA ASP 54 36.645 20.243 17.492 1.00 0.00

ATOM 817 CB ASP 54 37.805 22.090 17.458 1.00 0.00

ATOM 818 HB1 ASP 54 37.255 22.709 18.166 1.00 0.00

ATOM 819 HB2 ASP 54 38.850 22.388 17.539 1.00 0.00

ATOM 820 CG ASP 54 37.442 22.556 16.056 1.00 0.00

ATOM 821 OD1 ASP 54 36.222 22.682 15.818 1.00 0.00

ATOM 822 OD2 ASP 54 38.251 22.894 15.163 1.00 0.00

ATOM 823 C ASP 54 38.659 19.836 17.076 1.00 0.00

ATOM 824 O ASP 54 38.564 19.650 15.865 1.00 0.00

ATOM 825 N GLN 55 39.620 19.250 17.793 1.00 0.00

ATOM 826 H GLN 55 39.649 19.455 18.781 1.00 0.00

ATOM 827 CA GLN 55 40.813 18.553 17.353 1.00 0.00

ATOM 828 HA GLN 55 40.649 18.318 16.302 1.00 0.00

ATOM 829 CB GLN 55 42.111 19.357 17.328 1.00 0.00

ATOM 830 HB1 GLN 55 41.783 20.305 16.905 1.00 0.00

ATOM 831 HB2 GLN 55 42.899 18.876 16.748 1.00 0.00

ATOM 832 CG GLN 55 42.642 19.876 18.661 1.00 0.00

ATOM 833 HG1 GLN 55 41.849 20.063 19.385 1.00 0.00

ATOM 834 HG2 GLN 55 43.168 19.068 19.167 1.00 0.00

ATOM 835 CD GLN 55 43.625 21.038 18.638 1.00 0.00

ATOM 836 OE1 GLN 55 44.799 20.841 18.334 1.00 0.00

ATOM 837 NE2 GLN 55 43.216 22.197 19.156 1.00 0.00

ATOM 838 1HE2 GLN 55 42.257 22.303 19.459 1.00 0.00

ATOM 839 2HE2 GLN 55 44.018 22.761 19.398 1.00 0.00

ATOM 840 C GLN 55 40.946 17.212 18.060 1.00 0.00

ATOM 841 O GLN 55 42.011 16.603 18.027 1.00 0.00

ATOM 842 N THR 56 39.870 16.735 18.691 1.00 0.00

ATOM 843 H THR 56 38.977 17.186 18.558 1.00 0.00

ATOM 844 CA THR 56 39.639 15.402 19.215 1.00 0.00

ATOM 845 HA THR 56 38.691 15.404 19.753 1.00 0.00

ATOM 846 CB THR 56 39.407 14.305 18.181 1.00 0.00

ATOM 847 HB THR 56 40.379 13.998 17.799 1.00 0.00

ATOM 848 CG2 THR 56 38.670 13.068 18.687 1.00 0.00

ATOM 849 1HG2 THR 56 38.546 12.273 17.952 1.00 0.00

ATOM 850 2HG2 THR 56 37.676 13.423 18.960 1.00 0.00

ATOM 851 3HG2 THR 56 39.197 12.674 19.555 1.00 0.00

ATOM 852 OG1 THR 56 38.693 14.807 17.074 1.00 0.00

ATOM 853 HG1 THR 56 37.989 15.414 17.311 1.00 0.00

ATOM 854 C THR 56 40.676 15.024 20.263 1.00 0.00

ATOM 855 O THR 56 41.164 13.907 20.423 1.00 0.00

ATOM 856 N TYR 57 40.952 16.033 21.091 1.00 0.00

ATOM 857 H TYR 57 40.503 16.926 20.946 1.00 0.00

ATOM 858 CA TYR 57 41.722 15.977 22.318 1.00 0.00

ATOM 859 HA TYR 57 42.172 14.987 22.379 1.00 0.00

ATOM 860 CB TYR 57 42.810 17.030 22.132 1.00 0.00

ATOM 861 HB1 TYR 57 42.375 18.007 22.343 1.00 0.00

ATOM 862 HB2 TYR 57 43.047 16.938 21.073 1.00 0.00

ATOM 863 CG TYR 57 44.072 16.933 22.955 1.00 0.00

ATOM 864 CD1 TYR 57 45.121 16.042 22.695 1.00 0.00

ATOM 865 HD1 TYR 57 44.955 15.227 22.009 1.00 0.00

ATOM 866 CE1 TYR 57 46.135 15.861 23.644 1.00 0.00

ATOM 867 HE1 TYR 57 46.679 14.928 23.653 1.00 0.00

ATOM 868 CZ TYR 57 46.279 16.764 24.716 1.00 0.00

ATOM 869 OH TYR 57 47.312 16.656 25.600 1.00 0.00

ATOM 870 HH TYR 57 47.105 17.236 26.336 1.00 0.00

ATOM 871 CE2 TYR 57 45.343 17.812 24.820 1.00 0.00

ATOM 872 HE2 TYR 57 45.409 18.459 25.682 1.00 0.00

ATOM 873 CD2 TYR 57 44.222 17.869 23.984 1.00 0.00

ATOM 874 HD2 TYR 57 43.447 18.590 24.202 1.00 0.00

ATOM 875 C TYR 57 40.925 16.284 23.579 1.00 0.00

ATOM 876 O TYR 57 39.808 16.789 23.504 1.00 0.00

ATOM 877 N PRO 58 41.413 16.022 24.794 1.00 0.00

ATOM 878 CD PRO 58 42.656 15.346 25.109 1.00 0.00

ATOM 879 HD1 PRO 58 43.400 16.034 25.513 1.00 0.00

ATOM 880 HD2 PRO 58 43.065 14.728 24.311 1.00 0.00

ATOM 881 CG PRO 58 42.295 14.321 26.180 1.00 0.00

ATOM 882 HG1 PRO 58 43.086 13.826 26.745 1.00 0.00

ATOM 883 HG2 PRO 58 41.696 13.546 25.706 1.00 0.00

ATOM 884 CB PRO 58 41.308 15.121 27.024 1.00 0.00

ATOM 885 HB1 PRO 58 41.824 15.849 27.651 1.00 0.00

ATOM 886 HB2 PRO 58 40.625 14.523 27.628 1.00 0.00

ATOM 887 CA PRO 58 40.540 15.872 25.940 1.00 0.00

ATOM 888 HA PRO 58 39.734 15.221 25.602 1.00 0.00

ATOM 889 C PRO 58 40.149 17.263 26.418 1.00 0.00

ATOM 890 O PRO 58 40.892 18.188 26.095 1.00 0.00

ATOM 891 N PRO 59 39.017 17.439 27.104 1.00 0.00

ATOM 892 CD PRO 59 37.851 16.606 26.883 1.00 0.00

ATOM 893 HD1 PRO 59 37.641 16.087 27.818 1.00 0.00

ATOM 894 HD2 PRO 59 37.915 15.872 26.079 1.00 0.00

ATOM 895 CG PRO 59 36.720 17.585 26.584 1.00 0.00

ATOM 896 HG1 PRO 59 35.761 17.110 26.793 1.00 0.00

ATOM 897 HG2 PRO 59 36.777 17.964 25.565 1.00 0.00

ATOM 898 CB PRO 59 37.032 18.736 27.536 1.00 0.00

ATOM 899 HB1 PRO 59 36.668 18.408 28.506 1.00 0.00

ATOM 900 HB2 PRO 59 36.462 19.631 27.291 1.00 0.00

ATOM 901 CA PRO 59 38.559 18.748 27.526 1.00 0.00

ATOM 902 HA PRO 59 38.863 19.416 26.721 1.00 0.00

ATOM 903 C PRO 59 39.182 19.271 28.812 1.00 0.00

ATOM 904 O PRO 59 39.146 20.470 29.078 1.00 0.00

ATOM 905 N GLU 60 39.764 18.374 29.610 1.00 0.00

ATOM 906 H GLU 60 39.770 17.437 29.235 1.00 0.00

ATOM 907 CA GLU 60 40.321 18.636 30.923 1.00 0.00

ATOM 908 HA GLU 60 40.417 17.680 31.437 1.00 0.00

ATOM 909 CB GLU 60 41.689 19.307 30.812 1.00 0.00

ATOM 910 HB1 GLU 60 42.073 19.661 31.769 1.00 0.00

ATOM 911 HB2 GLU 60 41.649 20.225 30.227 1.00 0.00

ATOM 912 CG GLU 60 42.816 18.552 30.115 1.00 0.00

ATOM 913 HG1 GLU 60 42.877 17.553 30.544 1.00 0.00

ATOM 914 HG2 GLU 60 42.548 18.428 29.066 1.00 0.00

ATOM 915 CD GLU 60 44.167 19.255 30.127 1.00 0.00

ATOM 916 OE1 GLU 60 44.772 19.492 31.188 1.00 0.00

ATOM 917 OE2 GLU 60 44.749 19.565 29.066 1.00 0.00

ATOM 918 C GLU 60 39.313 19.260 31.879 1.00 0.00

ATOM 919 O GLU 60 39.730 20.010 32.758 1.00 0.00

ATOM 920 N SER 61 38.015 19.074 31.629 1.00 0.00

ATOM 921 H SER 61 37.723 18.472 30.872 1.00 0.00

ATOM 922 CA SER 61 36.902 19.704 32.311 1.00 0.00

ATOM 923 HA SER 61 37.253 20.603 32.817 1.00 0.00

ATOM 924 CB SER 61 35.885 19.969 31.203 1.00 0.00

ATOM 925 HB1 SER 61 35.526 19.055 30.739 1.00 0.00

ATOM 926 HB2 SER 61 35.058 20.495 31.679 1.00 0.00

ATOM 927 OG SER 61 36.367 20.876 30.238 1.00 0.00

ATOM 928 HG SER 61 37.289 20.958 30.494 1.00 0.00

ATOM 929 C SER 61 36.348 18.876 33.462 1.00 0.00

ATOM 930 OC1 SER 61 36.327 19.351 34.618 1.00 0.00

ATOM 931 OC2 SER 61 35.790 17.808 33.128 1.00 0.00

ATOM 932 N PHE 1 31.267 29.299 100.137 1.00 0.00

ATOM 933 H1 PHE 1 32.101 28.733 100.059 1.00 0.00

ATOM 934 H2 PHE 1 30.603 28.908 99.485 1.00 0.00

ATOM 935 H3 PHE 1 30.935 29.255 101.090 1.00 0.00

ATOM 936 CA PHE 1 31.540 30.666 99.669 1.00 0.00

ATOM 937 HA PHE 1 32.081 30.631 98.724 1.00 0.00

ATOM 938 CB PHE 1 30.215 31.341 99.329 1.00 0.00

ATOM 939 HB1 PHE 1 29.803 30.950 98.398 1.00 0.00

ATOM 940 HB2 PHE 1 30.460 32.348 98.990 1.00 0.00

ATOM 941 CG PHE 1 29.194 31.328 100.441 1.00 0.00

ATOM 942 CD1 PHE 1 28.335 30.238 100.629 1.00 0.00

ATOM 943 HD1 PHE 1 28.485 29.368 100.008 1.00 0.00

ATOM 944 CE1 PHE 1 27.369 30.239 101.641 1.00 0.00

ATOM 945 HE1 PHE 1 26.819 29.325 101.810 1.00 0.00

ATOM 946 CZ PHE 1 27.295 31.370 102.464 1.00 0.00

ATOM 947 HZ PHE 1 26.470 31.452 103.155 1.00 0.00

ATOM 948 CE2 PHE 1 28.121 32.488 102.286 1.00 0.00

ATOM 949 HE2 PHE 1 27.995 33.381 102.879 1.00 0.00

ATOM 950 CD2 PHE 1 29.012 32.486 101.206 1.00 0.00

ATOM 951 HD2 PHE 1 29.643 33.286 100.993 1.00 0.00

ATOM 952 C PHE 1 32.271 31.407 100.780 1.00 0.00

ATOM 953 O PHE 1 31.837 31.270 101.921 1.00 0.00

ATOM 954 N THR 2 33.363 32.109 100.466 1.00 0.00

ATOM 955 H THR 2 33.658 32.076 99.501 1.00 0.00

ATOM 956 CA THR 2 34.229 32.808 101.395 1.00 0.00

ATOM 957 HA THR 2 33.646 32.951 102.305 1.00 0.00

ATOM 958 CB THR 2 35.555 32.104 101.673 1.00 0.00

ATOM 959 HB THR 2 36.021 32.583 102.529 1.00 0.00

ATOM 960 CG2 THR 2 35.429 30.748 102.362 1.00 0.00

ATOM 961 1HG2 THR 2 36.420 30.382 102.623 1.00 0.00

ATOM 962 2HG2 THR 2 35.017 30.034 101.650 1.00 0.00

ATOM 963 3HG2 THR 2 34.750 30.742 103.216 1.00 0.00

ATOM 964 OG1 THR 2 36.474 32.121 100.612 1.00 0.00

ATOM 965 HG1 THR 2 36.104 31.629 99.870 1.00 0.00

ATOM 966 C THR 2 34.429 34.265 101.008 1.00 0.00

ATOM 967 O THR 2 33.982 34.663 99.935 1.00 0.00

ATOM 968 N LEU 3 35.004 35.077 101.898 1.00 0.00

ATOM 969 H LEU 3 35.255 34.704 102.802 1.00 0.00

ATOM 970 CA LEU 3 35.179 36.506 101.737 1.00 0.00

ATOM 971 HA LEU 3 34.282 36.887 101.250 1.00 0.00

ATOM 972 CB LEU 3 35.155 37.145 103.122 1.00 0.00

ATOM 973 HB1 LEU 3 36.108 36.949 103.613 1.00 0.00

ATOM 974 HB2 LEU 3 34.391 36.640 103.714 1.00 0.00

ATOM 975 CG LEU 3 35.044 38.661 103.270 1.00 0.00

ATOM 976 HG LEU 3 35.914 39.173 102.860 1.00 0.00

ATOM 977 CD1 LEU 3 33.747 39.230 102.704 1.00 0.00

ATOM 978 1HD1 LEU 3 32.883 38.669 103.061 1.00 0.00

ATOM 979 2HD1 LEU 3 33.790 39.167 101.616 1.00 0.00

ATOM 980 3HD1 LEU 3 33.682 40.297 102.917 1.00 0.00

ATOM 981 CD2 LEU 3 35.190 39.176 104.699 1.00 0.00

ATOM 982 1HD2 LEU 3 35.934 38.543 105.181 1.00 0.00

ATOM 983 2HD2 LEU 3 34.288 38.917 105.252 1.00 0.00

ATOM 984 3HD2 LEU 3 35.532 40.196 104.878 1.00 0.00

ATOM 985 C LEU 3 36.379 36.887 100.881 1.00 0.00

ATOM 986 O LEU 3 36.346 37.912 100.203 1.00 0.00

ATOM 987 N ILE 4 37.443 36.082 100.918 1.00 0.00

ATOM 988 H ILE 4 37.481 35.289 101.542 1.00 0.00

ATOM 989 CA ILE 4 38.640 36.292 100.129 1.00 0.00

ATOM 990 HA ILE 4 38.852 37.351 100.281 1.00 0.00

ATOM 991 CB ILE 4 39.766 35.447 100.718 1.00 0.00

ATOM 992 HB ILE 4 39.823 35.751 101.763 1.00 0.00

ATOM 993 CG2 ILE 4 39.498 33.967 100.448 1.00 0.00

ATOM 994 1HG2 ILE 4 38.577 33.540 100.843 1.00 0.00

ATOM 995 2HG2 ILE 4 40.278 33.390 100.945 1.00 0.00

ATOM 996 3HG2 ILE 4 39.442 33.729 99.386 1.00 0.00

ATOM 997 CG1 ILE 4 41.065 35.866 100.037 1.00 0.00

ATOM 998 1HG1 ILE 4 41.034 35.473 99.020 1.00 0.00

ATOM 999 2HG1 ILE 4 41.226 36.944 100.012 1.00 0.00

ATOM 1000 CD ILE 4 42.340 35.292 100.648 1.00 0.00

ATOM 1001 HD1 ILE 4 43.185 35.610 100.037 1.00 0.00

ATOM 1002 HD2 ILE 4 42.312 34.203 100.688 1.00 0.00

ATOM 1003 HD3 ILE 4 42.527 35.752 101.618 1.00 0.00

ATOM 1004 C ILE 4 38.397 36.007 98.654 1.00 0.00

ATOM 1005 O ILE 4 39.042 36.644 97.824 1.00 0.00

ATOM 1006 N GLU 5 37.440 35.132 98.339 1.00 0.00

ATOM 1007 H GLU 5 36.825 34.978 99.126 1.00 0.00

ATOM 1008 CA GLU 5 36.985 34.888 96.985 1.00 0.00

ATOM 1009 HA GLU 5 37.761 34.480 96.338 1.00 0.00

ATOM 1010 CB GLU 5 35.790 33.941 96.946 1.00 0.00

ATOM 1011 HB1 GLU 5 35.307 33.934 95.969 1.00 0.00

ATOM 1012 HB2 GLU 5 34.981 34.281 97.593 1.00 0.00

ATOM 1013 CG GLU 5 36.245 32.501 97.166 1.00 0.00

ATOM 1014 HG1 GLU 5 36.529 32.179 96.165 1.00 0.00

ATOM 1015 HG2 GLU 5 37.148 32.453 97.775 1.00 0.00

ATOM 1016 CD GLU 5 35.189 31.498 97.610 1.00 0.00

ATOM 1017 OE1 GLU 5 35.539 30.728 98.530 1.00 0.00

ATOM 1018 OE2 GLU 5 34.015 31.576 97.187 1.00 0.00

ATOM 1019 C GLU 5 36.500 36.172 96.329 1.00 0.00

ATOM 1020 O GLU 5 36.770 36.310 95.138 1.00 0.00

ATOM 1021 N LEU 6 36.007 37.134 97.112 1.00 0.00

ATOM 1022 H LEU 6 35.946 36.883 98.088 1.00 0.00

ATOM 1023 CA LEU 6 35.595 38.442 96.642 1.00 0.00

ATOM 1024 HA LEU 6 35.426 38.431 95.566 1.00 0.00

ATOM 1025 CB LEU 6 34.253 38.855 97.239 1.00 0.00

ATOM 1026 HB1 LEU 6 33.938 39.728 96.667 1.00 0.00

ATOM 1027 HB2 LEU 6 34.463 39.274 98.222 1.00 0.00

ATOM 1028 CG LEU 6 33.082 37.875 97.254 1.00 0.00

ATOM 1029 HG LEU 6 33.429 36.843 97.301 1.00 0.00

ATOM 1030 CD1 LEU 6 32.222 38.249 98.458 1.00 0.00

ATOM 1031 1HD1 LEU 6 31.313 37.671 98.297 1.00 0.00

ATOM 1032 2HD1 LEU 6 32.114 39.331 98.434 1.00 0.00

ATOM 1033 3HD1 LEU 6 32.645 37.955 99.418 1.00 0.00

ATOM 1034 CD2 LEU 6 32.232 37.903 95.988 1.00 0.00

ATOM 1035 1HD2 LEU 6 32.927 37.618 95.199 1.00 0.00

ATOM 1036 2HD2 LEU 6 31.916 38.937 95.847 1.00 0.00

ATOM 1037 3HD2 LEU 6 31.398 37.206 96.031 1.00 0.00

ATOM 1038 C LEU 6 36.666 39.497 96.879 1.00 0.00

ATOM 1039 O LEU 6 36.353 40.645 96.573 1.00 0.00

ATOM 1040 N LEU 7 37.873 39.130 97.318 1.00 0.00

ATOM 1041 H LEU 7 38.035 38.142 97.452 1.00 0.00

ATOM 1042 CA LEU 7 39.035 39.984 97.464 1.00 0.00

ATOM 1043 HA LEU 7 38.681 41.011 97.387 1.00 0.00

ATOM 1044 CB LEU 7 39.739 39.751 98.799 1.00 0.00

ATOM 1045 HB1 LEU 7 40.162 38.748 98.780 1.00 0.00

ATOM 1046 HB2 LEU 7 39.039 39.677 99.631 1.00 0.00

ATOM 1047 CG LEU 7 40.891 40.705 99.105 1.00 0.00

ATOM 1048 HG LEU 7 40.985 40.605 100.186 1.00 0.00

ATOM 1049 CD1 LEU 7 42.232 40.208 98.574 1.00 0.00

ATOM 1050 1HD1 LEU 7 42.245 40.232 97.484 1.00 0.00

ATOM 1051 2HD1 LEU 7 42.393 39.187 98.920 1.00 0.00

ATOM 1052 3HD1 LEU 7 43.062 40.868 98.823 1.00 0.00

ATOM 1053 CD2 LEU 7 40.674 42.184 98.800 1.00 0.00

ATOM 1054 1HD2 LEU 7 41.064 42.498 97.832 1.00 0.00

ATOM 1055 2HD2 LEU 7 41.316 42.754 99.470 1.00 0.00

ATOM 1056 3HD2 LEU 7 39.671 42.479 99.058 1.00 0.00

ATOM 1057 C LEU 7 39.913 39.803 96.235 1.00 0.00

ATOM 1058 O LEU 7 40.352 40.775 95.624 1.00 0.00

ATOM 1059 N ILE 8 40.343 38.582 95.916 1.00 0.00

ATOM 1060 H ILE 8 39.899 37.864 96.472 1.00 0.00

ATOM 1061 CA ILE 8 41.457 38.372 95.012 1.00 0.00

ATOM 1062 HA ILE 8 42.326 38.786 95.524 1.00 0.00

ATOM 1063 CB ILE 8 41.651 36.863 94.908 1.00 0.00

ATOM 1064 HB ILE 8 40.687 36.388 94.725 1.00 0.00

ATOM 1065 CG2 ILE 8 42.603 36.639 93.737 1.00 0.00

ATOM 1066 1HG2 ILE 8 42.789 35.571 93.628 1.00 0.00

ATOM 1067 2HG2 ILE 8 43.574 37.114 93.881 1.00 0.00

ATOM 1068 3HG2 ILE 8 42.180 37.024 92.809 1.00 0.00

ATOM 1069 CG1 ILE 8 42.299 36.374 96.201 1.00 0.00

ATOM 1070 1HG1 ILE 8 43.336 36.699 96.132 1.00 0.00

ATOM 1071 2HG1 ILE 8 41.758 36.923 96.972 1.00 0.00

ATOM 1072 CD ILE 8 42.139 34.861 96.324 1.00 0.00

ATOM 1073 HD1 ILE 8 42.726 34.521 97.177 1.00 0.00

ATOM 1074 HD2 ILE 8 42.492 34.340 95.434 1.00 0.00

ATOM 1075 HD3 ILE 8 41.087 34.659 96.527 1.00 0.00

ATOM 1076 C ILE 8 41.273 39.001 93.637 1.00 0.00

ATOM 1077 O ILE 8 42.087 39.789 93.162 1.00 0.00

ATOM 1078 N VAL 9 40.086 38.839 93.047 1.00 0.00

ATOM 1079 H VAL 9 39.431 38.176 93.436 1.00 0.00

ATOM 1080 CA VAL 9 39.701 39.470 91.801 1.00 0.00

ATOM 1081 HA VAL 9 40.476 39.264 91.062 1.00 0.00

ATOM 1082 CB VAL 9 38.406 38.935 91.194 1.00 0.00

ATOM 1083 HB VAL 9 38.419 37.846 91.164 1.00 0.00

ATOM 1084 CG1 VAL 9 37.102 39.248 91.921 1.00 0.00

ATOM 1085 1HG1 VAL 9 36.901 40.312 91.792 1.00 0.00

ATOM 1086 2HG1 VAL 9 37.166 38.999 92.980 1.00 0.00

ATOM 1087 3HG1 VAL 9 36.270 38.665 91.526 1.00 0.00

ATOM 1088 CG2 VAL 9 38.240 39.282 89.718 1.00 0.00

ATOM 1089 1HG2 VAL 9 37.309 38.808 89.405 1.00 0.00

ATOM 1090 2HG2 VAL 9 39.189 38.989 89.269 1.00 0.00

ATOM 1091 3HG2 VAL 9 38.162 40.316 89.385 1.00 0.00

ATOM 1092 C VAL 9 39.714 40.990 91.870 1.00 0.00

ATOM 1093 O VAL 9 40.015 41.621 90.859 1.00 0.00

ATOM 1094 N VAL 10 39.548 41.639 93.024 1.00 0.00

ATOM 1095 H VAL 10 39.295 41.081 93.826 1.00 0.00

ATOM 1096 CA VAL 10 39.835 43.050 93.195 1.00 0.00

ATOM 1097 HA VAL 10 39.549 43.602 92.301 1.00 0.00

ATOM 1098 CB VAL 10 38.884 43.542 94.282 1.00 0.00

ATOM 1099 HB VAL 10 39.039 42.898 95.148 1.00 0.00

ATOM 1100 CG1 VAL 10 39.077 45.022 94.599 1.00 0.00

ATOM 1101 1HG1 VAL 10 38.272 45.319 95.267 1.00 0.00

ATOM 1102 2HG1 VAL 10 39.059 45.706 93.750 1.00 0.00

ATOM 1103 3HG1 VAL 10 39.998 45.184 95.158 1.00 0.00

ATOM 1104 CG2 VAL 10 37.441 43.319 93.838 1.00 0.00

ATOM 1105 1HG2 VAL 10 37.187 42.270 93.690 1.00 0.00

ATOM 1106 2HG2 VAL 10 37.294 43.827 92.885 1.00 0.00

ATOM 1107 3HG2 VAL 10 36.733 43.687 94.581 1.00 0.00

ATOM 1108 C VAL 10 41.280 43.410 93.510 1.00 0.00

ATOM 1109 O VAL 10 41.713 44.490 93.112 1.00 0.00

ATOM 1110 N ALA 11 42.072 42.566 94.175 1.00 0.00

ATOM 1111 H ALA 11 41.678 41.743 94.609 1.00 0.00

ATOM 1112 CA ALA 11 43.512 42.712 94.198 1.00 0.00

ATOM 1113 HA ALA 11 43.728 43.720 94.554 1.00 0.00

ATOM 1114 CB ALA 11 44.104 41.759 95.233 1.00 0.00

ATOM 1115 HB1 ALA 11 43.988 40.707 94.972 1.00 0.00

ATOM 1116 HB2 ALA 11 43.734 41.782 96.258 1.00 0.00

ATOM 1117 HB3 ALA 11 45.162 42.018 95.277 1.00 0.00

ATOM 1118 C ALA 11 44.120 42.593 92.808 1.00 0.00

ATOM 1119 O ALA 11 45.165 43.218 92.646 1.00 0.00

ATOM 1120 N ILE 12 43.620 41.722 91.927 1.00 0.00

ATOM 1121 H ILE 12 42.805 41.180 92.177 1.00 0.00

ATOM 1122 CA ILE 12 44.071 41.542 90.562 1.00 0.00

ATOM 1123 HA ILE 12 45.017 42.079 90.510 1.00 0.00

ATOM 1124 CB ILE 12 44.353 40.099 90.148 1.00 0.00

ATOM 1125 HB ILE 12 44.830 40.158 89.170 1.00 0.00

ATOM 1126 CG2 ILE 12 45.361 39.488 91.116 1.00 0.00

ATOM 1127 1HG2 ILE 12 46.117 40.265 91.217 1.00 0.00

ATOM 1128 2HG2 ILE 12 45.997 38.706 90.699 1.00 0.00

ATOM 1129 3HG2 ILE 12 45.027 39.188 92.110 1.00 0.00

ATOM 1130 CG1 ILE 12 43.090 39.258 89.982 1.00 0.00

ATOM 1131 1HG1 ILE 12 42.599 38.958 90.908 1.00 0.00

ATOM 1132 2HG1 ILE 12 42.371 39.825 89.392 1.00 0.00

ATOM 1133 CD ILE 12 43.365 38.003 89.158 1.00 0.00

ATOM 1134 HD1 ILE 12 43.317 38.395 88.142 1.00 0.00

ATOM 1135 HD2 ILE 12 42.496 37.350 89.244 1.00 0.00

ATOM 1136 HD3 ILE 12 44.305 37.498 89.379 1.00 0.00

ATOM 1137 C ILE 12 43.274 42.315 89.522 1.00 0.00

ATOM 1138 O ILE 12 43.226 41.841 88.390 1.00 0.00

ATOM 1139 N ILE 13 42.535 43.363 89.893 1.00 0.00

ATOM 1140 H ILE 13 42.440 43.507 90.888 1.00 0.00

ATOM 1141 CA ILE 13 41.714 44.014 88.892 1.00 0.00

ATOM 1142 HA ILE 13 40.948 43.301 88.584 1.00 0.00

ATOM 1143 CB ILE 13 40.903 45.127 89.549 1.00 0.00

ATOM 1144 HB ILE 13 40.427 44.655 90.408 1.00 0.00

ATOM 1145 CG2 ILE 13 41.726 46.272 90.130 1.00 0.00

ATOM 1146 1HG2 ILE 13 41.970 46.983 89.340 1.00 0.00

ATOM 1147 2HG2 ILE 13 42.615 45.891 90.632 1.00 0.00

ATOM 1148 3HG2 ILE 13 41.133 46.798 90.879 1.00 0.00

ATOM 1149 CG1 ILE 13 39.681 45.548 88.738 1.00 0.00

ATOM 1150 1HG1 ILE 13 40.013 46.106 87.862 1.00 0.00

ATOM 1151 2HG1 ILE 13 39.150 44.645 88.434 1.00 0.00

ATOM 1152 CD ILE 13 38.847 46.436 89.659 1.00 0.00

ATOM 1153 HD1 ILE 13 39.354 47.387 89.821 1.00 0.00

ATOM 1154 HD2 ILE 13 38.533 46.023 90.618 1.00 0.00

ATOM 1155 HD3 ILE 13 37.912 46.701 89.170 1.00 0.00

ATOM 1156 C ILE 13 42.537 44.588 87.749 1.00 0.00

ATOM 1157 O ILE 13 41.985 44.602 86.652 1.00 0.00

ATOM 1158 N GLY 14 43.818 44.872 88.007 1.00 0.00

ATOM 1159 H GLY 14 44.198 44.834 88.942 1.00 0.00

ATOM 1160 CA GLY 14 44.847 45.233 87.052 1.00 0.00

ATOM 1161 HA1 GLY 14 44.597 46.182 86.580 1.00 0.00

ATOM 1162 HA2 GLY 14 45.786 45.432 87.568 1.00 0.00

ATOM 1163 C GLY 14 45.134 44.239 85.936 1.00 0.00

ATOM 1164 O GLY 14 45.319 44.634 84.789 1.00 0.00

ATOM 1165 N ILE 15 45.075 42.966 86.334 1.00 0.00

ATOM 1166 H ILE 15 44.821 42.825 87.301 1.00 0.00

ATOM 1167 CA ILE 15 45.466 41.867 85.474 1.00 0.00

ATOM 1168 HA ILE 15 46.346 42.186 84.915 1.00 0.00

ATOM 1169 CB ILE 15 45.904 40.644 86.274 1.00 0.00

ATOM 1170 HB ILE 15 45.157 40.460 87.044 1.00 0.00

ATOM 1171 CG2 ILE 15 45.969 39.376 85.426 1.00 0.00

ATOM 1172 1HG2 ILE 15 45.024 38.914 85.136 1.00 0.00

ATOM 1173 2HG2 ILE 15 46.525 38.664 86.034 1.00 0.00

ATOM 1174 3HG2 ILE 15 46.613 39.562 84.568 1.00 0.00

ATOM 1175 CG1 ILE 15 47.217 40.852 87.026 1.00 0.00

ATOM 1176 1HG1 ILE 15 48.049 41.109 86.371 1.00 0.00

ATOM 1177 2HG1 ILE 15 47.080 41.723 87.667 1.00 0.00

ATOM 1178 CD ILE 15 47.719 39.713 87.909 1.00 0.00

ATOM 1179 HD1 ILE 15 47.953 38.808 87.348 1.00 0.00

ATOM 1180 HD2 ILE 15 47.007 39.569 88.722 1.00 0.00

ATOM 1181 HD3 ILE 15 48.677 40.101 88.252 1.00 0.00

ATOM 1182 C ILE 15 44.371 41.589 84.455 1.00 0.00

ATOM 1183 O ILE 15 44.665 41.378 83.281 1.00 0.00

ATOM 1184 N LEU 16 43.111 41.670 84.889 1.00 0.00

ATOM 1185 H LEU 16 42.969 41.808 85.879 1.00 0.00

ATOM 1186 CA LEU 16 41.951 41.724 84.022 1.00 0.00

ATOM 1187 HA LEU 16 41.931 40.851 83.374 1.00 0.00

ATOM 1188 CB LEU 16 40.754 41.760 84.967 1.00 0.00

ATOM 1189 HB1 LEU 16 39.931 42.180 84.390 1.00 0.00

ATOM 1190 HB2 LEU 16 40.880 42.579 85.674 1.00 0.00

ATOM 1191 CG LEU 16 40.366 40.476 85.699 1.00 0.00

ATOM 1192 HG LEU 16 40.044 39.740 84.964 1.00 0.00

ATOM 1193 CD1 LEU 16 41.423 39.627 86.398 1.00 0.00

ATOM 1194 1HD1 LEU 16 42.236 39.362 85.722 1.00 0.00

ATOM 1195 2HD1 LEU 16 41.053 38.678 86.786 1.00 0.00

ATOM 1196 3HD1 LEU 16 41.766 40.129 87.303 1.00 0.00

ATOM 1197 CD2 LEU 16 39.187 40.794 86.615 1.00 0.00

ATOM 1198 1HD2 LEU 16 38.793 39.831 86.938 1.00 0.00

ATOM 1199 2HD2 LEU 16 38.415 41.354 86.087 1.00 0.00

ATOM 1200 3HD2 LEU 16 39.556 41.432 87.417 1.00 0.00

ATOM 1201 C LEU 16 42.104 42.946 83.129 1.00 0.00

ATOM 1202 O LEU 16 41.874 42.770 81.934 1.00 0.00

ATOM 1203 N ALA 17 42.441 44.126 83.651 1.00 0.00

ATOM 1204 H ALA 17 42.305 44.156 84.651 1.00 0.00

ATOM 1205 CA ALA 17 42.338 45.350 82.879 1.00 0.00

ATOM 1206 HA ALA 17 41.323 45.555 82.540 1.00 0.00

ATOM 1207 CB ALA 17 42.622 46.545 83.784 1.00 0.00

ATOM 1208 HB1 ALA 17 43.663 46.392 84.067 1.00 0.00

ATOM 1209 HB2 ALA 17 41.898 46.498 84.597 1.00 0.00

ATOM 1210 HB3 ALA 17 42.497 47.475 83.228 1.00 0.00

ATOM 1211 C ALA 17 43.313 45.382 81.711 1.00 0.00

ATOM 1212 O ALA 17 43.098 45.970 80.653 1.00 0.00

ATOM 1213 N ALA 18 44.374 44.599 81.914 1.00 0.00

ATOM 1214 H ALA 18 44.561 44.287 82.856 1.00 0.00

ATOM 1215 CA ALA 18 45.486 44.589 80.985 1.00 0.00

ATOM 1216 HA ALA 18 45.812 45.562 80.619 1.00 0.00

ATOM 1217 CB ALA 18 46.667 43.924 81.686 1.00 0.00

ATOM 1218 HB1 ALA 18 46.283 42.927 81.899 1.00 0.00

ATOM 1219 HB2 ALA 18 47.598 43.953 81.118 1.00 0.00

ATOM 1220 HB3 ALA 18 46.820 44.452 82.608 1.00 0.00

ATOM 1221 C ALA 18 45.107 43.913 79.676 1.00 0.00

ATOM 1222 O ALA 18 45.798 44.107 78.678 1.00 0.00

ATOM 1223 N ILE 19 44.036 43.117 79.613 1.00 0.00

ATOM 1224 H ILE 19 43.605 42.847 80.486 1.00 0.00

ATOM 1225 CA ILE 19 43.502 42.570 78.382 1.00 0.00

ATOM 1226 HA ILE 19 44.165 42.942 77.601 1.00 0.00

ATOM 1227 CB ILE 19 43.499 41.045 78.424 1.00 0.00

ATOM 1228 HB ILE 19 43.487 40.686 77.395 1.00 0.00

ATOM 1229 CG2 ILE 19 44.888 40.558 78.826 1.00 0.00

ATOM 1230 1HG2 ILE 19 44.934 39.497 78.579 1.00 0.00

ATOM 1231 2HG2 ILE 19 45.148 40.773 79.861 1.00 0.00

ATOM 1232 3HG2 ILE 19 45.610 41.131 78.249 1.00 0.00

ATOM 1233 CG1 ILE 19 42.508 40.348 79.353 1.00 0.00

ATOM 1234 1HG1 ILE 19 42.857 39.352 79.627 1.00 0.00

ATOM 1235 2HG1 ILE 19 42.472 40.949 80.261 1.00 0.00

ATOM 1236 CD ILE 19 41.079 40.204 78.835 1.00 0.00

ATOM 1237 HD1 ILE 19 40.570 41.167 78.878 1.00 0.00

ATOM 1238 HD2 ILE 19 40.516 39.428 79.352 1.00 0.00

ATOM 1239 HD3 ILE 19 41.052 39.939 77.779 1.00 0.00

ATOM 1240 C ILE 19 42.110 43.119 78.102 1.00 0.00

ATOM 1241 O ILE 19 41.562 43.093 77.003 1.00 0.00

ATOM 1242 N ALA 20 41.501 43.693 79.141 1.00 0.00

ATOM 1243 H ALA 20 41.877 43.642 80.078 1.00 0.00

ATOM 1244 CA ALA 20 40.180 44.248 78.925 1.00 0.00

ATOM 1245 HA ALA 20 39.564 43.573 78.330 1.00 0.00

ATOM 1246 CB ALA 20 39.568 44.271 80.323 1.00 0.00

ATOM 1247 HB1 ALA 20 38.654 44.862 80.311 1.00 0.00

ATOM 1248 HB2 ALA 20 39.408 43.228 80.600 1.00 0.00

ATOM 1249 HB3 ALA 20 40.121 44.887 81.031 1.00 0.00

ATOM 1250 C ALA 20 40.226 45.578 78.185 1.00 0.00

ATOM 1251 O ALA 20 39.538 45.890 77.215 1.00 0.00

ATOM 1252 N ILE 21 41.232 46.411 78.454 1.00 0.00

ATOM 1253 H ILE 21 41.927 46.222 79.161 1.00 0.00

ATOM 1254 CA ILE 21 41.327 47.756 77.922 1.00 0.00

ATOM 1255 HA ILE 21 40.344 48.221 77.985 1.00 0.00

ATOM 1256 CB ILE 21 42.204 48.634 78.811 1.00 0.00

ATOM 1257 HB ILE 21 43.121 48.078 78.996 1.00 0.00

ATOM 1258 CG2 ILE 21 42.603 49.949 78.148 1.00 0.00

ATOM 1259 1HG2 ILE 21 41.679 50.390 77.773 1.00 0.00

ATOM 1260 2HG2 ILE 21 43.188 49.687 77.267 1.00 0.00

ATOM 1261 3HG2 ILE 21 43.140 50.551 78.845 1.00 0.00

ATOM 1262 CG1 ILE 21 41.577 48.687 80.202 1.00 0.00

ATOM 1263 1HG1 ILE 21 40.617 49.181 80.061 1.00 0.00

ATOM 1264 2HG1 ILE 21 41.489 47.695 80.648 1.00 0.00

ATOM 1265 CD ILE 21 42.361 49.515 81.217 1.00 0.00

ATOM 1266 HD1 ILE 21 43.370 49.129 81.358 1.00 0.00

ATOM 1267 HD2 ILE 21 41.919 49.485 82.213 1.00 0.00

ATOM 1268 HD3 ILE 21 42.512 50.536 80.869 1.00 0.00

ATOM 1269 C ILE 21 41.693 47.812 76.446 1.00 0.00

ATOM 1270 O ILE 21 41.006 48.531 75.725 1.00 0.00

ATOM 1271 N PRO 22 42.613 47.024 75.883 1.00 0.00

ATOM 1272 CD PRO 22 43.570 46.238 76.634 1.00 0.00

ATOM 1273 HD1 PRO 22 42.972 45.553 77.236 1.00 0.00

ATOM 1274 HD2 PRO 22 44.252 46.815 77.259 1.00 0.00

ATOM 1275 CG PRO 22 44.394 45.498 75.584 1.00 0.00

ATOM 1276 HG1 PRO 22 43.984 44.506 75.397 1.00 0.00

ATOM 1277 HG2 PRO 22 45.416 45.324 75.918 1.00 0.00

ATOM 1278 CB PRO 22 44.319 46.432 74.379 1.00 0.00

ATOM 1279 HB1 PRO 22 44.477 45.842 73.476 1.00 0.00

ATOM 1280 HB2 PRO 22 45.066 47.222 74.460 1.00 0.00

ATOM 1281 CA PRO 22 42.914 47.023 74.466 1.00 0.00

ATOM 1282 HA PRO 22 42.855 48.071 74.173 1.00 0.00

ATOM 1283 C PRO 22 41.879 46.267 73.646 1.00 0.00

ATOM 1284 O PRO 22 41.746 46.482 72.443 1.00 0.00

ATOM 1285 N GLN 23 41.047 45.463 74.314 1.00 0.00

ATOM 1286 H GLN 23 41.100 45.216 75.292 1.00 0.00

ATOM 1287 CA GLN 23 39.929 44.817 73.659 1.00 0.00

ATOM 1288 HA GLN 23 40.348 44.389 72.749 1.00 0.00

ATOM 1289 CB GLN 23 39.323 43.815 74.637 1.00 0.00

ATOM 1290 HB1 GLN 23 38.942 44.367 75.496 1.00 0.00

ATOM 1291 HB2 GLN 23 40.097 43.088 74.882 1.00 0.00

ATOM 1292 CG GLN 23 38.181 42.969 74.081 1.00 0.00

ATOM 1293 HG1 GLN 23 37.218 43.471 73.984 1.00 0.00

ATOM 1294 HG2 GLN 23 38.448 42.661 73.070 1.00 0.00

ATOM 1295 CD GLN 23 37.745 41.803 74.958 1.00 0.00

ATOM 1296 OE1 GLN 23 38.212 40.670 74.859 1.00 0.00

ATOM 1297 NE2 GLN 23 36.756 41.988 75.832 1.00 0.00

ATOM 1298 1HE2 GLN 23 36.296 42.877 75.969 1.00 0.00

ATOM 1299 2HE2 GLN 23 36.368 41.118 76.170 1.00 0.00

ATOM 1300 C GLN 23 38.843 45.806 73.260 1.00 0.00

ATOM 1301 O GLN 23 38.215 45.669 72.213 1.00 0.00

ATOM 1302 N PHE 24 38.613 46.761 74.164 1.00 0.00

ATOM 1303 H PHE 24 39.135 46.738 75.029 1.00 0.00

ATOM 1304 CA PHE 24 37.582 47.740 73.882 1.00 0.00

ATOM 1305 HA PHE 24 36.818 47.182 73.341 1.00 0.00

ATOM 1306 CB PHE 24 36.813 48.056 75.162 1.00 0.00

ATOM 1307 HB1 PHE 24 37.524 48.456 75.885 1.00 0.00

ATOM 1308 HB2 PHE 24 36.411 47.095 75.482 1.00 0.00

ATOM 1309 CG PHE 24 35.666 49.016 74.955 1.00 0.00

ATOM 1310 CD1 PHE 24 34.384 48.490 74.754 1.00 0.00

ATOM 1311 HD1 PHE 24 34.225 47.425 74.677 1.00 0.00

ATOM 1312 CE1 PHE 24 33.273 49.328 74.594 1.00 0.00

ATOM 1313 HE1 PHE 24 32.287 48.897 74.506 1.00 0.00

ATOM 1314 CZ PHE 24 33.448 50.716 74.574 1.00 0.00

ATOM 1315 HZ PHE 24 32.560 51.323 74.673 1.00 0.00

ATOM 1316 CE2 PHE 24 34.728 51.258 74.736 1.00 0.00

ATOM 1317 HE2 PHE 24 34.762 52.337 74.774 1.00 0.00

ATOM 1318 CD2 PHE 24 35.830 50.403 74.867 1.00 0.00

ATOM 1319 HD2 PHE 24 36.798 50.838 75.068 1.00 0.00

ATOM 1320 C PHE 24 38.116 48.911 73.070 1.00 0.00

ATOM 1321 O PHE 24 37.415 49.487 72.240 1.00 0.00

ATOM 1322 N SER 25 39.362 49.345 73.272 1.00 0.00

ATOM 1323 H SER 25 39.969 48.907 73.950 1.00 0.00

ATOM 1324 CA SER 25 39.708 50.697 72.885 1.00 0.00

ATOM 1325 HA SER 25 38.850 51.318 73.143 1.00 0.00

ATOM 1326 CB SER 25 40.886 51.281 73.659 1.00 0.00

ATOM 1327 HB1 SER 25 40.629 51.244 74.718 1.00 0.00

ATOM 1328 HB2 SER 25 41.015 52.307 73.314 1.00 0.00

ATOM 1329 OG SER 25 42.097 50.644 73.322 1.00 0.00

ATOM 1330 HG SER 25 42.704 50.790 74.051 1.00 0.00

ATOM 1331 C SER 25 39.814 50.922 71.383 1.00 0.00

ATOM 1332 O SER 25 39.935 52.074 70.975 1.00 0.00

ATOM 1333 N ALA 26 39.723 49.947 70.474 1.00 0.00

ATOM 1334 H ALA 26 39.608 48.993 70.782 1.00 0.00

ATOM 1335 CA ALA 26 39.671 50.111 69.035 1.00 0.00

ATOM 1336 HA ALA 26 39.960 51.150 68.881 1.00 0.00

ATOM 1337 CB ALA 26 40.821 49.318 68.423 1.00 0.00

ATOM 1338 HB1 ALA 26 41.820 49.692 68.645 1.00 0.00

ATOM 1339 HB2 ALA 26 40.761 48.276 68.738 1.00 0.00

ATOM 1340 HB3 ALA 26 40.756 49.441 67.342 1.00 0.00

ATOM 1341 C ALA 26 38.290 49.926 68.421 1.00 0.00

ATOM 1342 O ALA 26 38.099 50.086 67.217 1.00 0.00

ATOM 1343 N ALA 27 37.347 49.445 69.234 1.00 0.00

ATOM 1344 H ALA 27 37.636 49.544 70.196 1.00 0.00

ATOM 1345 CA ALA 27 36.028 48.968 68.869 1.00 0.00

ATOM 1346 HA ALA 27 36.045 48.051 68.279 1.00 0.00

ATOM 1347 CB ALA 27 35.311 48.448 70.113 1.00 0.00

ATOM 1348 HB1 ALA 27 35.796 47.600 70.598 1.00 0.00

ATOM 1349 HB2 ALA 27 34.386 47.968 69.799 1.00 0.00

ATOM 1350 HB3 ALA 27 35.072 49.211 70.854 1.00 0.00

ATOM 1351 C ALA 27 35.261 49.948 67.992 1.00 0.00

ATOM 1352 O ALA 27 34.625 49.542 67.023 1.00 0.00

ATOM 1353 N ARG 28 35.150 51.217 68.391 1.00 0.00

ATOM 1354 H ARG 28 35.525 51.446 69.301 1.00 0.00

ATOM 1355 CA ARG 28 34.299 52.132 67.658 1.00 0.00

ATOM 1356 HA ARG 28 33.315 51.664 67.618 1.00 0.00

ATOM 1357 CB ARG 28 34.174 53.464 68.392 1.00 0.00

ATOM 1358 HB1 ARG 28 35.130 53.905 68.672 1.00 0.00

ATOM 1359 HB2 ARG 28 33.669 53.213 69.324 1.00 0.00

ATOM 1360 CG ARG 28 33.347 54.600 67.794 1.00 0.00

ATOM 1361 HG1 ARG 28 32.333 54.313 67.514 1.00 0.00

ATOM 1362 HG2 ARG 28 33.157 55.287 68.618 1.00 0.00

ATOM 1363 CD ARG 28 33.951 55.524 66.741 1.00 0.00

ATOM 1364 HD1 ARG 28 34.126 54.906 65.862 1.00 0.00

ATOM 1365 HD2 ARG 28 33.139 56.210 66.507 1.00 0.00

ATOM 1366 NE ARG 28 35.239 56.126 67.090 1.00 0.00

ATOM 1367 HE ARG 28 35.505 56.152 68.064 1.00 0.00

ATOM 1368 CZ ARG 28 36.123 56.817 66.357 1.00 0.00

ATOM 1369 NH1 ARG 28 35.910 57.007 65.050 1.00 0.00

ATOM 1370 1HH1 ARG 28 35.056 56.667 64.630 1.00 0.00

ATOM 1371 2HH1 ARG 28 36.616 57.485 64.506 1.00 0.00

ATOM 1372 NH2 ARG 28 37.289 57.207 66.886 1.00 0.00

ATOM 1373 1HH2 ARG 28 37.342 56.924 67.855 1.00 0.00

ATOM 1374 2HH2 ARG 28 38.125 57.417 66.361 1.00 0.00

ATOM 1375 C ARG 28 34.736 52.331 66.214 1.00 0.00

ATOM 1376 O ARG 28 33.948 52.252 65.274 1.00 0.00

ATOM 1377 N VAL 29 36.042 52.424 65.952 1.00 0.00

ATOM 1378 H VAL 29 36.698 52.378 66.717 1.00 0.00

ATOM 1379 CA VAL 29 36.662 52.499 64.644 1.00 0.00

ATOM 1380 HA VAL 29 36.124 53.244 64.059 1.00 0.00

ATOM 1381 CB VAL 29 38.143 52.847 64.773 1.00 0.00

ATOM 1382 HB VAL 29 38.698 52.156 65.408 1.00 0.00

ATOM 1383 CG1 VAL 29 38.811 52.724 63.408 1.00 0.00

ATOM 1384 1HG1 VAL 29 38.376 53.470 62.743 1.00 0.00

ATOM 1385 2HG1 VAL 29 38.791 51.744 62.932 1.00 0.00

ATOM 1386 3HG1 VAL 29 39.849 52.990 63.608 1.00 0.00

ATOM 1387 CG2 VAL 29 38.421 54.268 65.257 1.00 0.00

ATOM 1388 1HG2 VAL 29 39.462 54.427 65.527 1.00 0.00

ATOM 1389 2HG2 VAL 29 37.958 54.307 66.242 1.00 0.00

ATOM 1390 3HG2 VAL 29 37.891 55.031 64.687 1.00 0.00

ATOM 1391 C VAL 29 36.441 51.213 63.861 1.00 0.00

ATOM 1392 O VAL 29 36.031 51.202 62.703 1.00 0.00

ATOM 1393 N LYS 30 36.679 50.091 64.546 1.00 0.00

ATOM 1394 H LYS 30 37.026 50.196 65.489 1.00 0.00

ATOM 1395 CA LYS 30 36.420 48.780 63.983 1.00 0.00

ATOM 1396 HA LYS 30 37.087 48.632 63.134 1.00 0.00

ATOM 1397 CB LYS 30 36.660 47.701 65.035 1.00 0.00

ATOM 1398 HB1 LYS 30 36.247 46.809 64.565 1.00 0.00

ATOM 1399 HB2 LYS 30 36.115 47.934 65.950 1.00 0.00

ATOM 1400 CG LYS 30 38.063 47.265 65.445 1.00 0.00

ATOM 1401 HG1 LYS 30 38.606 46.715 64.677 1.00 0.00

ATOM 1402 HG2 LYS 30 38.590 48.203 65.626 1.00 0.00

ATOM 1403 CD LYS 30 38.011 46.325 66.647 1.00 0.00

ATOM 1404 HD1 LYS 30 37.569 46.801 67.522 1.00 0.00

ATOM 1405 HD2 LYS 30 37.551 45.348 66.497 1.00 0.00

ATOM 1406 CE LYS 30 39.445 45.929 66.983 1.00 0.00

ATOM 1407 HE1 LYS 30 39.888 45.410 66.133 1.00 0.00

ATOM 1408 HE2 LYS 30 39.983 46.844 67.232 1.00 0.00

ATOM 1409 NZ LYS 30 39.552 45.124 68.208 1.00 0.00

ATOM 1410 HZ1 LYS 30 39.219 44.187 68.034 1.00 0.00

ATOM 1411 HZ2 LYS 30 38.890 45.462 68.891 1.00 0.00

ATOM 1412 HZ3 LYS 30 40.531 45.167 68.460 1.00 0.00

ATOM 1413 C LYS 30 35.037 48.526 63.401 1.00 0.00

ATOM 1414 O LYS 30 34.909 47.996 62.305 1.00 0.00

ATOM 1415 N ALA 31 34.027 48.975 64.149 1.00 0.00

ATOM 1416 H ALA 31 34.234 49.444 65.019 1.00 0.00

ATOM 1417 CA ALA 31 32.617 48.892 63.822 1.00 0.00

ATOM 1418 HA ALA 31 32.459 47.917 63.362 1.00 0.00

ATOM 1419 CB ALA 31 31.835 49.097 65.118 1.00 0.00

ATOM 1420 HB1 ALA 31 32.385 48.610 65.923 1.00 0.00

ATOM 1421 HB2 ALA 31 31.890 50.140 65.428 1.00 0.00

ATOM 1422 HB3 ALA 31 30.808 48.744 65.028 1.00 0.00

ATOM 1423 C ALA 31 32.194 49.826 62.698 1.00 0.00

ATOM 1424 O ALA 31 31.590 49.399 61.717 1.00 0.00

ATOM 1425 N TYR 32 32.336 51.133 62.933 1.00 0.00

ATOM 1426 H TYR 32 32.780 51.383 63.804 1.00 0.00

ATOM 1427 CA TYR 32 31.726 52.152 62.102 1.00 0.00

ATOM 1428 HA TYR 32 30.964 51.584 61.570 1.00 0.00

ATOM 1429 CB TYR 32 30.945 53.124 62.981 1.00 0.00

ATOM 1430 HB1 TYR 32 30.306 53.799 62.412 1.00 0.00

ATOM 1431 HB2 TYR 32 31.681 53.753 63.480 1.00 0.00

ATOM 1432 CG TYR 32 30.151 52.447 64.073 1.00 0.00

ATOM 1433 CD1 TYR 32 30.528 52.622 65.410 1.00 0.00

ATOM 1434 HD1 TYR 32 31.250 53.376 65.683 1.00 0.00

ATOM 1435 CE1 TYR 32 30.052 51.777 66.420 1.00 0.00

ATOM 1436 HE1 TYR 32 30.469 51.844 67.414 1.00 0.00

ATOM 1437 CZ TYR 32 29.017 50.865 66.131 1.00 0.00

ATOM 1438 OH TYR 32 28.467 50.063 67.085 1.00 0.00

ATOM 1439 HH TYR 32 28.935 50.214 67.912 1.00 0.00

ATOM 1440 CE2 TYR 32 28.570 50.783 64.798 1.00 0.00

ATOM 1441 HE2 TYR 32 27.772 50.097 64.552 1.00 0.00

ATOM 1442 CD2 TYR 32 29.163 51.507 63.756 1.00 0.00

ATOM 1443 HD2 TYR 32 28.774 51.419 62.753 1.00 0.00

ATOM 1444 C TYR 32 32.610 52.970 61.169 1.00 0.00

ATOM 1445 O TYR 32 32.385 54.101 60.742 1.00 0.00

ATOM 1446 N ASN 33 33.780 52.422 60.838 1.00 0.00

ATOM 1447 H ASN 33 33.885 51.491 61.213 1.00 0.00

ATOM 1448 CA ASN 33 34.657 52.913 59.794 1.00 0.00

ATOM 1449 HA ASN 33 34.042 53.430 59.057 1.00 0.00

ATOM 1450 CB ASN 33 35.721 53.760 60.488 1.00 0.00

ATOM 1451 HB1 ASN 33 36.053 53.433 61.473 1.00 0.00

ATOM 1452 HB2 ASN 33 36.623 53.632 59.890 1.00 0.00

ATOM 1453 CG ASN 33 35.387 55.244 60.542 1.00 0.00

ATOM 1454 OD1 ASN 33 35.312 55.890 61.585 1.00 0.00

ATOM 1455 ND2 ASN 33 35.136 55.826 59.367 1.00 0.00

ATOM 1456 1HD2 ASN 33 35.408 55.398 58.494 1.00 0.00

ATOM 1457 2HD2 ASN 33 34.919 56.811 59.420 1.00 0.00

ATOM 1458 C ASN 33 35.330 51.854 58.933 1.00 0.00

ATOM 1459 O ASN 33 35.376 51.956 57.708 1.00 0.00

ATOM 1460 N SER 34 35.980 50.927 59.639 1.00 0.00

ATOM 1461 H SER 34 35.880 50.898 60.645 1.00 0.00

ATOM 1462 CA SER 34 37.025 50.153 59.000 1.00 0.00

ATOM 1463 HA SER 34 37.811 50.830 58.671 1.00 0.00

ATOM 1464 CB SER 34 37.700 49.205 59.986 1.00 0.00

ATOM 1465 HB1 SER 34 38.499 48.656 59.488 1.00 0.00

ATOM 1466 HB2 SER 34 37.016 48.436 60.343 1.00 0.00

ATOM 1467 OG SER 34 38.300 49.871 61.075 1.00 0.00

ATOM 1468 HG SER 34 38.793 49.225 61.585 1.00 0.00

ATOM 1469 C SER 34 36.676 49.385 57.733 1.00 0.00

ATOM 1470 O SER 34 37.478 49.282 56.807 1.00 0.00

ATOM 1471 N ALA 35 35.443 48.878 57.658 1.00 0.00

ATOM 1472 H ALA 35 34.896 48.950 58.504 1.00 0.00

ATOM 1473 CA ALA 35 34.923 48.189 56.494 1.00 0.00

ATOM 1474 HA ALA 35 35.707 47.503 56.174 1.00 0.00

ATOM 1475 CB ALA 35 33.656 47.426 56.871 1.00 0.00

ATOM 1476 HB1 ALA 35 33.618 47.399 57.960 1.00 0.00

ATOM 1477 HB2 ALA 35 32.784 47.976 56.519 1.00 0.00

ATOM 1478 HB3 ALA 35 33.564 46.394 56.534 1.00 0.00

ATOM 1479 C ALA 35 34.719 49.119 55.306 1.00 0.00

ATOM 1480 O ALA 35 35.152 48.885 54.179 1.00 0.00

ATOM 1481 N ALA 36 34.039 50.244 55.532 1.00 0.00

ATOM 1482 H ALA 36 33.965 50.569 56.486 1.00 0.00

ATOM 1483 CA ALA 36 33.930 51.331 54.580 1.00 0.00

ATOM 1484 HA ALA 36 33.327 51.006 53.732 1.00 0.00

ATOM 1485 CB ALA 36 33.116 52.479 55.171 1.00 0.00

ATOM 1486 HB1 ALA 36 33.231 53.356 54.533 1.00 0.00

ATOM 1487 HB2 ALA 36 33.418 52.844 56.153 1.00 0.00

ATOM 1488 HB3 ALA 36 32.067 52.188 55.168 1.00 0.00

ATOM 1489 C ALA 36 35.286 51.786 54.058 1.00 0.00

ATOM 1490 O ALA 36 35.569 51.876 52.866 1.00 0.00

ATOM 1491 N SER 37 36.270 51.901 54.951 1.00 0.00

ATOM 1492 H SER 37 35.993 51.836 55.920 1.00 0.00

ATOM 1493 CA SER 37 37.631 52.241 54.587 1.00 0.00

ATOM 1494 HA SER 37 37.590 53.218 54.108 1.00 0.00

ATOM 1495 CB SER 37 38.467 52.329 55.861 1.00 0.00

ATOM 1496 HB1 SER 37 38.526 51.290 56.188 1.00 0.00

ATOM 1497 HB2 SER 37 39.449 52.735 55.617 1.00 0.00

ATOM 1498 OG SER 37 37.946 53.157 56.876 1.00 0.00

ATOM 1499 HG SER 37 38.619 53.283 57.549 1.00 0.00

ATOM 1500 C SER 37 38.264 51.297 53.574 1.00 0.00

ATOM 1501 O SER 37 38.997 51.692 52.669 1.00 0.00

ATOM 1502 N SER 38 38.015 50.013 53.840 1.00 0.00

ATOM 1503 H SER 38 37.488 49.786 54.671 1.00 0.00

ATOM 1504 CA SER 38 38.656 48.931 53.121 1.00 0.00

ATOM 1505 HA SER 38 39.716 49.181 53.072 1.00 0.00

ATOM 1506 CB SER 38 38.532 47.645 53.933 1.00 0.00

ATOM 1507 HB1 SER 38 38.982 46.862 53.323 1.00 0.00

ATOM 1508 HB2 SER 38 37.487 47.418 54.146 1.00 0.00

ATOM 1509 OG SER 38 39.313 47.701 55.105 1.00 0.00

ATOM 1510 HG SER 38 38.711 48.108 55.733 1.00 0.00

ATOM 1511 C SER 38 38.080 48.664 51.738 1.00 0.00

ATOM 1512 O SER 38 38.824 48.528 50.768 1.00 0.00

ATOM 1513 N ASP 39 36.758 48.794 51.612 1.00 0.00

ATOM 1514 H ASP 39 36.204 49.072 52.409 1.00 0.00

ATOM 1515 CA ASP 39 36.047 48.597 50.364 1.00 0.00

ATOM 1516 HA ASP 39 36.483 47.761 49.818 1.00 0.00

ATOM 1517 CB ASP 39 34.589 48.237 50.632 1.00 0.00

ATOM 1518 HB1 ASP 39 34.593 47.570 51.492 1.00 0.00

ATOM 1519 HB2 ASP 39 34.064 49.137 50.949 1.00 0.00

ATOM 1520 CG ASP 39 33.846 47.555 49.490 1.00 0.00

ATOM 1521 OD1 ASP 39 34.345 46.496 49.052 1.00 0.00

ATOM 1522 OD2 ASP 39 32.790 47.990 48.984 1.00 0.00

ATOM 1523 C ASP 39 36.211 49.828 49.483 1.00 0.00

ATOM 1524 O ASP 39 36.496 49.691 48.296 1.00 0.00

ATOM 1525 N LEU 40 36.162 51.048 50.024 1.00 0.00

ATOM 1526 H LEU 40 36.070 51.081 51.028 1.00 0.00

ATOM 1527 CA LEU 40 36.420 52.287 49.316 1.00 0.00

ATOM 1528 HA LEU 40 35.745 52.424 48.471 1.00 0.00

ATOM 1529 CB LEU 40 36.391 53.505 50.235 1.00 0.00

ATOM 1530 HB1 LEU 40 36.899 54.369 49.805 1.00 0.00

ATOM 1531 HB2 LEU 40 36.932 53.321 51.162 1.00 0.00

ATOM 1532 CG LEU 40 35.011 54.015 50.639 1.00 0.00

ATOM 1533 HG LEU 40 34.457 53.301 51.248 1.00 0.00

ATOM 1534 CD1 LEU 40 35.159 55.196 51.593 1.00 0.00

ATOM 1535 1HD1 LEU 40 35.648 54.843 52.501 1.00 0.00

ATOM 1536 2HD1 LEU 40 34.170 55.606 51.795 1.00 0.00

ATOM 1537 3HD1 LEU 40 35.810 55.946 51.143 1.00 0.00

ATOM 1538 CD2 LEU 40 34.200 54.523 49.450 1.00 0.00

ATOM 1539 1HD2 LEU 40 33.425 55.248 49.697 1.00 0.00

ATOM 1540 2HD2 LEU 40 33.699 53.681 48.973 1.00 0.00

ATOM 1541 3HD2 LEU 40 34.887 55.005 48.754 1.00 0.00

ATOM 1542 C LEU 40 37.810 52.347 48.699 1.00 0.00

ATOM 1543 O LEU 40 37.956 52.724 47.538 1.00 0.00

ATOM 1544 N ARG 41 38.818 51.857 49.423 1.00 0.00

ATOM 1545 H ARG 41 38.615 51.374 50.287 1.00 0.00

ATOM 1546 CA ARG 41 40.149 51.621 48.903 1.00 0.00

ATOM 1547 HA ARG 41 40.511 52.516 48.398 1.00 0.00

ATOM 1548 CB ARG 41 41.056 51.407 50.113 1.00 0.00

ATOM 1549 HB1 ARG 41 40.620 50.662 50.777 1.00 0.00

ATOM 1550 HB2 ARG 41 40.963 52.272 50.769 1.00 0.00

ATOM 1551 CG ARG 41 42.541 51.142 49.879 1.00 0.00

ATOM 1552 HG1 ARG 41 42.602 50.162 49.407 1.00 0.00

ATOM 1553 HG2 ARG 41 42.982 51.918 49.253 1.00 0.00

ATOM 1554 CD ARG 41 43.375 51.112 51.157 1.00 0.00

ATOM 1555 HD1 ARG 41 44.401 50.851 50.903 1.00 0.00

ATOM 1556 HD2 ARG 41 43.453 52.132 51.533 1.00 0.00

ATOM 1557 NE ARG 41 43.037 50.103 52.162 1.00 0.00

ATOM 1558 HE ARG 41 42.625 50.544 52.972 1.00 0.00

ATOM 1559 CZ ARG 41 43.167 48.769 52.162 1.00 0.00

ATOM 1560 NH1 ARG 41 43.801 48.112 51.183 1.00 0.00

ATOM 1561 1HH1 ARG 41 44.520 48.625 50.692 1.00 0.00

ATOM 1562 2HH1 ARG 41 43.840 47.103 51.182 1.00 0.00

ATOM 1563 NH2 ARG 41 42.704 48.117 53.235 1.00 0.00

ATOM 1564 1HH2 ARG 41 42.209 48.581 53.985 1.00 0.00

ATOM 1565 2HH2 ARG 41 43.023 47.171 53.379 1.00 0.00

ATOM 1566 C ARG 41 40.250 50.502 47.875 1.00 0.00

ATOM 1567 O ARG 41 40.921 50.750 46.877 1.00 0.00

ATOM 1568 N ASN 42 39.586 49.347 47.954 1.00 0.00

ATOM 1569 H ASN 42 39.220 49.143 48.872 1.00 0.00

ATOM 1570 CA ASN 42 39.551 48.376 46.876 1.00 0.00

ATOM 1571 HA ASN 42 40.580 48.158 46.591 1.00 0.00

ATOM 1572 CB ASN 42 38.998 47.101 47.503 1.00 0.00

ATOM 1573 HB1 ASN 42 39.543 46.901 48.426 1.00 0.00

ATOM 1574 HB2 ASN 42 37.955 47.335 47.715 1.00 0.00

ATOM 1575 CG ASN 42 39.169 45.754 46.813 1.00 0.00

ATOM 1576 OD1 ASN 42 40.245 45.164 46.752 1.00 0.00

ATOM 1577 ND2 ASN 42 38.112 45.284 46.149 1.00 0.00

ATOM 1578 1HD2 ASN 42 37.258 45.816 46.055 1.00 0.00

ATOM 1579 2HD2 ASN 42 38.282 44.426 45.643 1.00 0.00

ATOM 1580 C ASN 42 38.800 48.821 45.629 1.00 0.00

ATOM 1581 O ASN 42 39.249 48.591 44.508 1.00 0.00

ATOM 1582 N LEU 43 37.686 49.543 45.770 1.00 0.00

ATOM 1583 H LEU 43 37.212 49.708 46.647 1.00 0.00

ATOM 1584 CA LEU 43 37.073 50.172 44.617 1.00 0.00

ATOM 1585 HA LEU 43 36.972 49.487 43.775 1.00 0.00

ATOM 1586 CB LEU 43 35.696 50.694 45.019 1.00 0.00

ATOM 1587 HB1 LEU 43 35.836 51.396 45.839 1.00 0.00

ATOM 1588 HB2 LEU 43 35.172 49.901 45.552 1.00 0.00

ATOM 1589 CG LEU 43 34.729 51.243 43.974 1.00 0.00

ATOM 1590 HG LEU 43 35.338 51.948 43.408 1.00 0.00

ATOM 1591 CD1 LEU 43 34.339 50.117 43.021 1.00 0.00

ATOM 1592 1HD1 LEU 43 35.201 49.652 42.542 1.00 0.00

ATOM 1593 2HD1 LEU 43 33.565 50.481 42.344 1.00 0.00

ATOM 1594 3HD1 LEU 43 33.878 49.256 43.503 1.00 0.00

ATOM 1595 CD2 LEU 43 33.440 51.848 44.524 1.00 0.00

ATOM 1596 1HD2 LEU 43 33.675 52.612 45.266 1.00 0.00

ATOM 1597 2HD2 LEU 43 32.836 51.083 45.012 1.00 0.00

ATOM 1598 3HD2 LEU 43 32.836 52.324 43.758 1.00 0.00

ATOM 1599 C LEU 43 37.964 51.267 44.049 1.00 0.00

ATOM 1600 O LEU 43 38.074 51.262 42.826 1.00 0.00

ATOM 1601 N LYS 44 38.697 52.104 44.786 1.00 0.00

ATOM 1602 H LYS 44 38.763 51.869 45.766 1.00 0.00

ATOM 1603 CA LYS 44 39.692 53.030 44.282 1.00 0.00

ATOM 1604 HA LYS 44 39.128 53.714 43.648 1.00 0.00

ATOM 1605 CB LYS 44 40.237 53.798 45.482 1.00 0.00

ATOM 1606 HB1 LYS 44 40.661 53.070 46.173 1.00 0.00

ATOM 1607 HB2 LYS 44 39.362 54.122 46.022 1.00 0.00

ATOM 1608 CG LYS 44 41.236 54.877 45.075 1.00 0.00

ATOM 1609 HG1 LYS 44 42.174 54.398 44.795 1.00 0.00

ATOM 1610 HG2 LYS 44 40.868 55.583 44.329 1.00 0.00

ATOM 1611 CD LYS 44 41.436 55.700 46.343 1.00 0.00

ATOM 1612 HD1 LYS 44 40.447 56.132 46.488 1.00 0.00

ATOM 1613 HD2 LYS 44 41.572 55.077 47.227 1.00 0.00

ATOM 1614 CE LYS 44 42.462 56.816 46.165 1.00 0.00

ATOM 1615 HE1 LYS 44 42.394 57.389 47.088 1.00 0.00

ATOM 1616 HE2 LYS 44 43.397 56.257 46.151 1.00 0.00

ATOM 1617 NZ LYS 44 42.232 57.722 45.028 1.00 0.00

ATOM 1618 HZ1 LYS 44 41.262 57.843 44.774 1.00 0.00

ATOM 1619 HZ2 LYS 44 42.593 57.385 44.147 1.00 0.00

ATOM 1620 HZ3 LYS 44 42.580 58.653 45.207 1.00 0.00

ATOM 1621 C LYS 44 40.734 52.412 43.361 1.00 0.00

ATOM 1622 O LYS 44 40.976 52.981 42.299 1.00 0.00

ATOM 1623 N THR 45 41.326 51.256 43.669 1.00 0.00

ATOM 1624 H THR 45 41.121 50.823 44.557 1.00 0.00

ATOM 1625 CA THR 45 42.245 50.510 42.832 1.00 0.00

ATOM 1626 HA THR 45 42.997 51.200 42.448 1.00 0.00

ATOM 1627 CB THR 45 43.085 49.480 43.585 1.00 0.00

ATOM 1628 HB THR 45 43.868 49.059 42.956 1.00 0.00

ATOM 1629 CG2 THR 45 43.876 50.075 44.746 1.00 0.00

ATOM 1630 1HG2 THR 45 44.611 49.363 45.123 1.00 0.00

ATOM 1631 2HG2 THR 45 43.229 50.509 45.508 1.00 0.00

ATOM 1632 3HG2 THR 45 44.462 50.917 44.381 1.00 0.00

ATOM 1633 OG1 THR 45 42.415 48.418 44.227 1.00 0.00

ATOM 1634 HG1 THR 45 43.043 47.779 44.573 1.00 0.00

ATOM 1635 C THR 45 41.549 49.804 41.677 1.00 0.00

ATOM 1636 O THR 45 42.160 49.531 40.647 1.00 0.00

ATOM 1637 N ALA 46 40.280 49.400 41.774 1.00 0.00

ATOM 1638 H ALA 46 39.768 49.594 42.623 1.00 0.00

ATOM 1639 CA ALA 46 39.568 48.687 40.733 1.00 0.00

ATOM 1640 HA ALA 46 40.207 48.044 40.127 1.00 0.00

ATOM 1641 CB ALA 46 38.560 47.763 41.410 1.00 0.00

ATOM 1642 HB1 ALA 46 39.121 47.023 41.979 1.00 0.00

ATOM 1643 HB2 ALA 46 38.116 47.159 40.621 1.00 0.00

ATOM 1644 HB3 ALA 46 37.835 48.259 42.055 1.00 0.00

ATOM 1645 C ALA 46 38.868 49.632 39.767 1.00 0.00

ATOM 1646 O ALA 46 39.219 49.570 38.591 1.00 0.00

ATOM 1647 N LEU 47 37.922 50.467 40.204 1.00 0.00

ATOM 1648 H LEU 47 37.753 50.672 41.179 1.00 0.00

ATOM 1649 CA LEU 47 37.076 51.232 39.312 1.00 0.00

ATOM 1650 HA LEU 47 37.127 50.636 38.401 1.00 0.00

ATOM 1651 CB LEU 47 35.625 51.238 39.788 1.00 0.00

ATOM 1652 HB1 LEU 47 35.597 51.774 40.736 1.00 0.00

ATOM 1653 HB2 LEU 47 35.379 50.230 40.120 1.00 0.00

ATOM 1654 CG LEU 47 34.532 51.839 38.908 1.00 0.00

ATOM 1655 HG LEU 47 33.555 51.703 39.372 1.00 0.00

ATOM 1656 CD1 LEU 47 34.592 53.317 38.531 1.00 0.00

ATOM 1657 1HD1 LEU 47 34.844 53.864 39.440 1.00 0.00

ATOM 1658 2HD1 LEU 47 33.610 53.611 38.161 1.00 0.00

ATOM 1659 3HD1 LEU 47 35.376 53.458 37.787 1.00 0.00

ATOM 1660 CD2 LEU 47 34.491 51.105 37.572 1.00 0.00

ATOM 1661 1HD2 LEU 47 35.421 51.230 37.017 1.00 0.00

ATOM 1662 2HD2 LEU 47 33.700 51.507 36.939 1.00 0.00

ATOM 1663 3HD2 LEU 47 34.308 50.053 37.794 1.00 0.00

ATOM 1664 C LEU 47 37.601 52.646 39.103 1.00 0.00

ATOM 1665 O LEU 47 37.685 53.185 38.002 1.00 0.00

ATOM 1666 N GLU 48 37.968 53.355 40.172 1.00 0.00

ATOM 1667 H GLU 48 37.904 52.932 41.086 1.00 0.00

ATOM 1668 CA GLU 48 38.292 54.766 40.099 1.00 0.00

ATOM 1669 HA GLU 48 37.484 55.170 39.490 1.00 0.00

ATOM 1670 CB GLU 48 38.172 55.407 41.478 1.00 0.00

ATOM 1671 HB1 GLU 48 38.476 56.447 41.353 1.00 0.00

ATOM 1672 HB2 GLU 48 38.904 54.881 42.091 1.00 0.00

ATOM 1673 CG GLU 48 36.796 55.402 42.141 1.00 0.00

ATOM 1674 HG1 GLU 48 36.049 55.634 41.384 1.00 0.00

ATOM 1675 HG2 GLU 48 36.580 54.470 42.663 1.00 0.00

ATOM 1676 CD GLU 48 36.684 56.514 43.174 1.00 0.00

ATOM 1677 OE1 GLU 48 37.568 56.452 44.055 1.00 0.00

ATOM 1678 OE2 GLU 48 35.874 57.462 43.078 1.00 0.00

ATOM 1679 C GLU 48 39.601 55.112 39.402 1.00 0.00

ATOM 1680 O GLU 48 39.731 56.114 38.702 1.00 0.00

ATOM 1681 N SER 49 40.550 54.184 39.543 1.00 0.00

ATOM 1682 H SER 49 40.397 53.507 40.276 1.00 0.00

ATOM 1683 CA SER 49 41.864 54.338 38.951 1.00 0.00

ATOM 1684 HA SER 49 42.247 55.340 39.147 1.00 0.00

ATOM 1685 CB SER 49 42.791 53.229 39.443 1.00 0.00

ATOM 1686 HB1 SER 49 43.756 53.510 39.024 1.00 0.00

ATOM 1687 HB2 SER 49 42.529 52.190 39.241 1.00 0.00

ATOM 1688 OG SER 49 43.099 53.212 40.819 1.00 0.00

ATOM 1689 HG SER 49 42.306 53.168 41.357 1.00 0.00

ATOM 1690 C SER 49 41.699 54.207 37.444 1.00 0.00

ATOM 1691 O SER 49 42.268 54.926 36.628 1.00 0.00

ATOM 1692 N ALA 50 40.922 53.192 37.056 1.00 0.00

ATOM 1693 H ALA 50 40.440 52.718 37.807 1.00 0.00

ATOM 1694 CA ALA 50 40.533 52.949 35.681 1.00 0.00

ATOM 1695 HA ALA 50 41.417 52.910 35.046 1.00 0.00

ATOM 1696 CB ALA 50 39.808 51.610 35.575 1.00 0.00

ATOM 1697 HB1 ALA 50 39.062 51.630 36.368 1.00 0.00

ATOM 1698 HB2 ALA 50 39.424 51.427 34.571 1.00 0.00

ATOM 1699 HB3 ALA 50 40.355 50.762 35.951 1.00 0.00

ATOM 1700 C ALA 50 39.713 54.045 35.017 1.00 0.00

ATOM 1701 O ALA 50 40.043 54.360 33.875 1.00 0.00

ATOM 1702 N PHE 51 38.718 54.582 35.726 1.00 0.00

ATOM 1703 H PHE 51 38.557 54.242 36.664 1.00 0.00

ATOM 1704 CA PHE 51 37.992 55.735 35.235 1.00 0.00

ATOM 1705 HA PHE 51 37.287 55.478 34.444 1.00 0.00

ATOM 1706 CB PHE 51 37.094 56.168 36.390 1.00 0.00

ATOM 1707 HB1 PHE 51 37.566 56.349 37.357 1.00 0.00

ATOM 1708 HB2 PHE 51 36.478 55.292 36.587 1.00 0.00

ATOM 1709 CG PHE 51 36.113 57.273 36.082 1.00 0.00

ATOM 1710 CD1 PHE 51 35.044 57.090 35.195 1.00 0.00

ATOM 1711 HD1 PHE 51 34.856 56.109 34.785 1.00 0.00

ATOM 1712 CE1 PHE 51 34.216 58.175 34.883 1.00 0.00

ATOM 1713 HE1 PHE 51 33.357 58.153 34.230 1.00 0.00

ATOM 1714 CZ PHE 51 34.598 59.434 35.362 1.00 0.00

ATOM 1715 HZ PHE 51 33.939 60.264 35.153 1.00 0.00

ATOM 1716 CE2 PHE 51 35.678 59.616 36.233 1.00 0.00

ATOM 1717 HE2 PHE 51 35.789 60.619 36.618 1.00 0.00

ATOM 1718 CD2 PHE 51 36.432 58.513 36.649 1.00 0.00

ATOM 1719 HD2 PHE 51 37.257 58.702 37.320 1.00 0.00

ATOM 1720 C PHE 51 38.915 56.897 34.899 1.00 0.00

ATOM 1721 O PHE 51 38.795 57.561 33.872 1.00 0.00

ATOM 1722 N ALA 52 39.943 57.145 35.712 1.00 0.00

ATOM 1723 H ALA 52 39.956 56.634 36.583 1.00 0.00

ATOM 1724 CA ALA 52 40.752 58.326 35.482 1.00 0.00

ATOM 1725 HA ALA 52 40.133 59.187 35.228 1.00 0.00

ATOM 1726 CB ALA 52 41.503 58.672 36.764 1.00 0.00

ATOM 1727 HB1 ALA 52 41.970 57.740 37.080 1.00 0.00

ATOM 1728 HB2 ALA 52 42.270 59.427 36.582 1.00 0.00

ATOM 1729 HB3 ALA 52 40.836 58.905 37.594 1.00 0.00

ATOM 1730 C ALA 52 41.721 58.183 34.317 1.00 0.00

ATOM 1731 O ALA 52 41.845 59.074 33.480 1.00 0.00

ATOM 1732 N ASP 53 42.456 57.077 34.181 1.00 0.00

ATOM 1733 H ASP 53 42.193 56.316 34.790 1.00 0.00

ATOM 1734 CA ASP 53 43.405 56.853 33.108 1.00 0.00

ATOM 1735 HA ASP 53 43.999 57.756 32.968 1.00 0.00

ATOM 1736 CB ASP 53 44.314 55.653 33.358 1.00 0.00

ATOM 1737 HB1 ASP 53 44.742 55.696 34.359 1.00 0.00

ATOM 1738 HB2 ASP 53 43.750 54.722 33.303 1.00 0.00

ATOM 1739 CG ASP 53 45.469 55.556 32.371 1.00 0.00

ATOM 1740 OD1 ASP 53 46.344 56.448 32.410 1.00 0.00

ATOM 1741 OD2 ASP 53 45.440 54.594 31.576 1.00 0.00

ATOM 1742 C ASP 53 42.775 56.610 31.745 1.00 0.00

ATOM 1743 O ASP 53 43.387 57.066 30.782 1.00 0.00

ATOM 1744 N ASP 54 41.542 56.101 31.696 1.00 0.00

ATOM 1745 H ASP 54 41.182 55.714 32.556 1.00 0.00

ATOM 1746 CA ASP 54 40.720 56.039 30.504 1.00 0.00

ATOM 1747 HA ASP 54 41.292 55.453 29.785 1.00 0.00

ATOM 1748 CB ASP 54 39.466 55.221 30.803 1.00 0.00

ATOM 1749 HB1 ASP 54 39.823 54.222 31.051 1.00 0.00

ATOM 1750 HB2 ASP 54 38.859 55.654 31.598 1.00 0.00

ATOM 1751 CG ASP 54 38.631 54.982 29.553 1.00 0.00

ATOM 1752 OD1 ASP 54 39.069 54.138 28.744 1.00 0.00

ATOM 1753 OD2 ASP 54 37.461 55.423 29.562 1.00 0.00

ATOM 1754 C ASP 54 40.501 57.382 29.823 1.00 0.00

ATOM 1755 O ASP 54 40.310 57.432 28.610 1.00 0.00

ATOM 1756 N GLN 55 40.503 58.488 30.569 1.00 0.00

ATOM 1757 H GLN 55 40.766 58.310 31.528 1.00 0.00

ATOM 1758 CA GLN 55 40.397 59.871 30.145 1.00 0.00

ATOM 1759 HA GLN 55 40.073 59.914 29.106 1.00 0.00

ATOM 1760 CB GLN 55 39.209 60.569 30.802 1.00 0.00

ATOM 1761 HB1 GLN 55 38.323 60.043 30.446 1.00 0.00

ATOM 1762 HB2 GLN 55 39.128 61.601 30.459 1.00 0.00

ATOM 1763 CG GLN 55 39.235 60.626 32.326 1.00 0.00

ATOM 1764 HG1 GLN 55 39.351 59.657 32.813 1.00 0.00

ATOM 1765 HG2 GLN 55 40.153 61.140 32.613 1.00 0.00

ATOM 1766 CD GLN 55 38.098 61.473 32.880 1.00 0.00

ATOM 1767 OE1 GLN 55 36.939 61.066 32.921 1.00 0.00

ATOM 1768 NE2 GLN 55 38.472 62.668 33.341 1.00 0.00

ATOM 1769 1HE2 GLN 55 39.433 62.975 33.291 1.00 0.00

ATOM 1770 2HE2 GLN 55 37.717 63.311 33.534 1.00 0.00

ATOM 1771 C GLN 55 41.738 60.587 30.234 1.00 0.00

ATOM 1772 O GLN 55 41.884 61.805 30.170 1.00 0.00

ATOM 1773 N THR 56 42.807 59.813 30.421 1.00 0.00

ATOM 1774 H THR 56 42.583 58.841 30.580 1.00 0.00

ATOM 1775 CA THR 56 44.173 60.289 30.515 1.00 0.00

ATOM 1776 HA THR 56 44.629 59.329 30.753 1.00 0.00

ATOM 1777 CB THR 56 44.796 60.759 29.204 1.00 0.00

ATOM 1778 HB THR 56 45.843 61.003 29.381 1.00 0.00

ATOM 1779 CG2 THR 56 44.637 59.719 28.098 1.00 0.00

ATOM 1780 1HG2 THR 56 45.208 58.802 28.239 1.00 0.00

ATOM 1781 2HG2 THR 56 44.885 60.141 27.124 1.00 0.00

ATOM 1782 3HG2 THR 56 43.583 59.461 28.031 1.00 0.00

ATOM 1783 OG1 THR 56 44.163 61.946 28.783 1.00 0.00

ATOM 1784 HG1 THR 56 43.283 61.885 29.163 1.00 0.00

ATOM 1785 C THR 56 44.406 61.261 31.665 1.00 0.00

ATOM 1786 O THR 56 45.195 62.192 31.526 1.00 0.00

ATOM 1787 N TYR 57 43.622 61.220 32.743 1.00 0.00

ATOM 1788 H TYR 57 43.138 60.335 32.798 1.00 0.00

ATOM 1789 CA TYR 57 43.812 62.048 33.917 1.00 0.00

ATOM 1790 HA TYR 57 44.367 62.925 33.586 1.00 0.00

ATOM 1791 CB TYR 57 42.385 62.440 34.296 1.00 0.00

ATOM 1792 HB1 TYR 57 41.892 61.544 34.668 1.00 0.00

ATOM 1793 HB2 TYR 57 41.782 62.829 33.474 1.00 0.00

ATOM 1794 CG TYR 57 42.221 63.458 35.398 1.00 0.00

ATOM 1795 CD1 TYR 57 42.273 64.833 35.139 1.00 0.00

ATOM 1796 HD1 TYR 57 42.173 65.172 34.118 1.00 0.00

ATOM 1797 CE1 TYR 57 42.324 65.748 36.196 1.00 0.00

ATOM 1798 HE1 TYR 57 42.142 66.760 35.899 1.00 0.00

ATOM 1799 CZ TYR 57 42.250 65.263 37.518 1.00 0.00

ATOM 1800 OH TYR 57 42.179 66.156 38.546 1.00 0.00

ATOM 1801 HH TYR 57 41.955 65.671 39.344 1.00 0.00

ATOM 1802 CE2 TYR 57 42.108 63.887 37.785 1.00 0.00

ATOM 1803 HE2 TYR 57 41.916 63.585 38.805 1.00 0.00

ATOM 1804 CD2 TYR 57 42.092 62.990 36.711 1.00 0.00

ATOM 1805 HD2 TYR 57 42.062 61.920 36.853 1.00 0.00

ATOM 1806 C TYR 57 44.672 61.302 34.929 1.00 0.00

ATOM 1807 O TYR 57 44.910 60.096 34.906 1.00 0.00

ATOM 1808 N PRO 58 45.306 62.029 35.851 1.00 0.00

ATOM 1809 CD PRO 58 45.478 63.467 35.900 1.00 0.00

ATOM 1810 HD1 PRO 58 44.611 63.969 36.329 1.00 0.00

ATOM 1811 HD2 PRO 58 45.676 63.879 34.911 1.00 0.00

ATOM 1812 CG PRO 58 46.715 63.627 36.777 1.00 0.00

ATOM 1813 HG1 PRO 58 46.666 64.601 37.261 1.00 0.00

ATOM 1814 HG2 PRO 58 47.648 63.389 36.265 1.00 0.00

ATOM 1815 CB PRO 58 46.477 62.583 37.863 1.00 0.00

ATOM 1816 HB1 PRO 58 45.687 62.944 38.524 1.00 0.00

ATOM 1817 HB2 PRO 58 47.454 62.346 38.286 1.00 0.00

ATOM 1818 CA PRO 58 45.965 61.436 36.998 1.00 0.00

ATOM 1819 HA PRO 58 46.851 60.896 36.665 1.00 0.00

ATOM 1820 C PRO 58 45.150 60.455 37.831 1.00 0.00

ATOM 1821 O PRO 58 44.090 60.886 38.270 1.00 0.00

ATOM 1822 N PRO 59 45.591 59.249 38.196 1.00 0.00

ATOM 1823 CD PRO 59 46.807 58.659 37.677 1.00 0.00

ATOM 1824 HD1 PRO 59 47.683 59.303 37.765 1.00 0.00

ATOM 1825 HD2 PRO 59 46.627 58.416 36.629 1.00 0.00

ATOM 1826 CG PRO 59 47.070 57.425 38.533 1.00 0.00

ATOM 1827 HG1 PRO 59 47.418 57.751 39.515 1.00 0.00

ATOM 1828 HG2 PRO 59 47.764 56.747 38.037 1.00 0.00

ATOM 1829 CB PRO 59 45.647 56.876 38.510 1.00 0.00

ATOM 1830 HB1 PRO 59 45.614 56.153 39.324 1.00 0.00

ATOM 1831 HB2 PRO 59 45.435 56.450 37.528 1.00 0.00

ATOM 1832 CA PRO 59 44.910 58.162 38.872 1.00 0.00

ATOM 1833 HA PRO 59 43.895 58.133 38.474 1.00 0.00

ATOM 1834 C PRO 59 44.902 58.417 40.372 1.00 0.00

ATOM 1835 O PRO 59 44.027 57.853 41.023 1.00 0.00

ATOM 1836 N GLU 60 45.753 59.263 40.959 1.00 0.00

ATOM 1837 H GLU 60 46.495 59.650 40.393 1.00 0.00

ATOM 1838 CA GLU 60 45.664 59.759 42.317 1.00 0.00

ATOM 1839 HA GLU 60 46.631 60.222 42.506 1.00 0.00

ATOM 1840 CB GLU 60 44.574 60.824 42.413 1.00 0.00

ATOM 1841 HB1 GLU 60 44.311 61.106 43.433 1.00 0.00

ATOM 1842 HB2 GLU 60 43.752 60.274 41.955 1.00 0.00

ATOM 1843 CG GLU 60 44.974 62.129 41.731 1.00 0.00

ATOM 1844 HG1 GLU 60 45.943 62.460 42.105 1.00 0.00

ATOM 1845 HG2 GLU 60 45.106 62.049 40.653 1.00 0.00

ATOM 1846 CD GLU 60 43.876 63.171 41.897 1.00 0.00

ATOM 1847 OE1 GLU 60 44.124 64.353 42.208 1.00 0.00

ATOM 1848 OE2 GLU 60 42.684 62.796 41.888 1.00 0.00

ATOM 1849 C GLU 60 45.622 58.724 43.432 1.00 0.00

ATOM 1850 O GLU 60 45.343 59.054 44.582 1.00 0.00

ATOM 1851 N SER 61 45.798 57.438 43.121 1.00 0.00

ATOM 1852 H SER 61 46.171 57.184 42.217 1.00 0.00

ATOM 1853 CA SER 61 45.370 56.332 43.954 1.00 0.00

ATOM 1854 HA SER 61 44.816 56.783 44.778 1.00 0.00

ATOM 1855 CB SER 61 44.428 55.363 43.247 1.00 0.00

ATOM 1856 HB1 SER 61 44.920 54.849 42.421 1.00 0.00

ATOM 1857 HB2 SER 61 44.125 54.598 43.961 1.00 0.00

ATOM 1858 OG SER 61 43.309 56.107 42.820 1.00 0.00

ATOM 1859 HG SER 61 43.654 56.672 42.124 1.00 0.00

ATOM 1860 C SER 61 46.498 55.506 44.557 1.00 0.00

ATOM 1861 OC1 SER 61 46.459 55.259 45.781 1.00 0.00

ATOM 1862 OC2 SER 61 47.513 55.164 43.914 1.00 0.00

ATOM 1863 N PHE 1 38.667 30.525 115.795 1.00 0.00

ATOM 1864 H1 PHE 1 38.596 30.414 116.796 1.00 0.00

ATOM 1865 H2 PHE 1 39.384 31.181 115.521 1.00 0.00

ATOM 1866 H3 PHE 1 38.911 29.626 115.408 1.00 0.00

ATOM 1867 CA PHE 1 37.362 30.959 115.273 1.00 0.00

ATOM 1868 HA PHE 1 37.320 30.930 114.184 1.00 0.00

ATOM 1869 CB PHE 1 36.279 29.985 115.727 1.00 0.00

ATOM 1870 HB1 PHE 1 35.275 30.356 115.521 1.00 0.00

ATOM 1871 HB2 PHE 1 36.488 29.870 116.790 1.00 0.00

ATOM 1872 CG PHE 1 36.440 28.594 115.162 1.00 0.00

ATOM 1873 CD1 PHE 1 35.977 28.330 113.868 1.00 0.00

ATOM 1874 HD1 PHE 1 35.579 29.148 113.286 1.00 0.00

ATOM 1875 CE1 PHE 1 35.904 26.998 113.442 1.00 0.00

ATOM 1876 HE1 PHE 1 35.420 26.700 112.524 1.00 0.00

ATOM 1877 CZ PHE 1 36.490 25.974 114.195 1.00 0.00

ATOM 1878 HZ PHE 1 36.575 24.973 113.798 1.00 0.00

ATOM 1879 CE2 PHE 1 36.921 26.246 115.499 1.00 0.00

ATOM 1880 HE2 PHE 1 37.293 25.484 116.150 1.00 0.00

ATOM 1881 CD2 PHE 1 36.804 27.542 116.013 1.00 0.00

ATOM 1882 HD2 PHE 1 36.984 27.594 117.076 1.00 0.00

ATOM 1883 C PHE 1 37.036 32.354 115.784 1.00 0.00

ATOM 1884 O PHE 1 37.170 32.598 116.980 1.00 0.00

ATOM 1885 N THR 2 36.417 33.046 114.825 1.00 0.00

ATOM 1886 H THR 2 36.303 32.643 113.906 1.00 0.00

ATOM 1887 CA THR 2 35.866 34.373 115.011 1.00 0.00

ATOM 1888 HA THR 2 35.440 34.408 116.014 1.00 0.00

ATOM 1889 CB THR 2 36.978 35.417 114.964 1.00 0.00

ATOM 1890 HB THR 2 37.723 35.120 115.701 1.00 0.00

ATOM 1891 CG2 THR 2 37.624 35.454 113.582 1.00 0.00

ATOM 1892 1HG2 THR 2 38.293 34.595 113.631 1.00 0.00

ATOM 1893 2HG2 THR 2 38.089 36.423 113.399 1.00 0.00

ATOM 1894 3HG2 THR 2 36.933 35.206 112.776 1.00 0.00

ATOM 1895 OG1 THR 2 36.522 36.730 115.198 1.00 0.00

ATOM 1896 HG1 THR 2 37.180 37.306 114.799 1.00 0.00

ATOM 1897 C THR 2 34.641 34.609 114.140 1.00 0.00

ATOM 1898 O THR 2 34.449 34.016 113.081 1.00 0.00

ATOM 1899 N LEU 3 33.717 35.434 114.636 1.00 0.00

ATOM 1900 H LEU 3 34.054 35.908 115.461 1.00 0.00

ATOM 1901 CA LEU 3 32.365 35.674 114.170 1.00 0.00

ATOM 1902 HA LEU 3 32.070 34.785 113.611 1.00 0.00

ATOM 1903 CB LEU 3 31.446 35.909 115.365 1.00 0.00

ATOM 1904 HB1 LEU 3 31.848 36.718 115.959 1.00 0.00

ATOM 1905 HB2 LEU 3 31.551 35.019 115.985 1.00 0.00

ATOM 1906 CG LEU 3 29.979 36.264 115.141 1.00 0.00

ATOM 1907 HG LEU 3 29.916 37.166 114.532 1.00 0.00

ATOM 1908 CD1 LEU 3 29.199 35.133 114.477 1.00 0.00

ATOM 1909 1HD1 LEU 3 29.210 34.248 115.112 1.00 0.00

ATOM 1910 2HD1 LEU 3 29.665 34.906 113.518 1.00 0.00

ATOM 1911 3HD1 LEU 3 28.179 35.431 114.233 1.00 0.00

ATOM 1912 CD2 LEU 3 29.251 36.547 116.451 1.00 0.00

ATOM 1913 1HD2 LEU 3 29.188 35.610 117.003 1.00 0.00

ATOM 1914 2HD2 LEU 3 28.258 36.880 116.252 1.00 0.00

ATOM 1915 3HD2 LEU 3 29.842 37.231 117.060 1.00 0.00

ATOM 1916 C LEU 3 32.320 36.839 113.191 1.00 0.00

ATOM 1917 O LEU 3 31.540 36.868 112.243 1.00 0.00

ATOM 1918 N ILE 4 33.267 37.776 113.274 1.00 0.00

ATOM 1919 H ILE 4 33.902 37.685 114.054 1.00 0.00

ATOM 1920 CA ILE 4 33.585 38.894 112.407 1.00 0.00

ATOM 1921 HA ILE 4 32.760 39.591 112.552 1.00 0.00

ATOM 1922 CB ILE 4 34.817 39.553 113.021 1.00 0.00

ATOM 1923 HB ILE 4 34.758 39.545 114.109 1.00 0.00

ATOM 1924 CG2 ILE 4 36.100 38.799 112.687 1.00 0.00

ATOM 1925 1HG2 ILE 4 35.965 37.719 112.748 1.00 0.00

ATOM 1926 2HG2 ILE 4 36.937 39.084 113.317 1.00 0.00

ATOM 1927 3HG2 ILE 4 36.342 39.076 111.661 1.00 0.00

ATOM 1928 CG1 ILE 4 35.156 41.016 112.750 1.00 0.00

ATOM 1929 1HG1 ILE 4 36.168 41.158 113.126 1.00 0.00

ATOM 1930 2HG1 ILE 4 35.106 41.287 111.696 1.00 0.00

ATOM 1931 CD ILE 4 34.345 41.967 113.627 1.00 0.00

ATOM 1932 HD1 ILE 4 34.784 42.044 114.622 1.00 0.00

ATOM 1933 HD2 ILE 4 33.270 41.782 113.641 1.00 0.00

ATOM 1934 HD3 ILE 4 34.480 42.986 113.269 1.00 0.00

ATOM 1935 C ILE 4 33.797 38.685 110.914 1.00 0.00

ATOM 1936 O ILE 4 33.879 39.685 110.204 1.00 0.00

ATOM 1937 N GLU 5 33.977 37.464 110.406 1.00 0.00

ATOM 1938 H GLU 5 34.168 36.728 111.071 1.00 0.00

ATOM 1939 CA GLU 5 33.977 37.115 108.999 1.00 0.00

ATOM 1940 HA GLU 5 33.945 37.982 108.340 1.00 0.00

ATOM 1941 CB GLU 5 35.308 36.450 108.660 1.00 0.00

ATOM 1942 HB1 GLU 5 35.464 35.507 109.184 1.00 0.00

ATOM 1943 HB2 GLU 5 36.141 37.103 108.921 1.00 0.00

ATOM 1944 CG GLU 5 35.376 36.188 107.158 1.00 0.00

ATOM 1945 HG1 GLU 5 34.514 35.568 106.913 1.00 0.00

ATOM 1946 HG2 GLU 5 35.294 37.100 106.567 1.00 0.00

ATOM 1947 CD GLU 5 36.693 35.511 106.803 1.00 0.00

ATOM 1948 OE1 GLU 5 36.865 34.296 107.041 1.00 0.00

ATOM 1949 OE2 GLU 5 37.582 36.285 106.392 1.00 0.00

ATOM 1950 C GLU 5 32.662 36.444 108.629 1.00 0.00

ATOM 1951 O GLU 5 32.179 36.784 107.552 1.00 0.00

ATOM 1952 N LEU 6 31.945 35.811 109.560 1.00 0.00

ATOM 1953 H LEU 6 32.177 36.005 110.524 1.00 0.00

ATOM 1954 CA LEU 6 30.759 35.078 109.162 1.00 0.00

ATOM 1955 HA LEU 6 31.001 34.570 108.228 1.00 0.00

ATOM 1956 CB LEU 6 30.481 33.998 110.204 1.00 0.00

ATOM 1957 HB1 LEU 6 30.125 34.458 111.124 1.00 0.00

ATOM 1958 HB2 LEU 6 31.364 33.482 110.581 1.00 0.00

ATOM 1959 CG LEU 6 29.595 32.822 109.800 1.00 0.00

ATOM 1960 HG LEU 6 29.507 32.028 110.542 1.00 0.00

ATOM 1961 CD1 LEU 6 28.161 33.250 109.501 1.00 0.00

ATOM 1962 1HD1 LEU 6 27.701 33.519 110.452 1.00 0.00

ATOM 1963 2HD1 LEU 6 27.631 32.410 109.052 1.00 0.00

ATOM 1964 3HD1 LEU 6 28.091 34.086 108.806 1.00 0.00

ATOM 1965 CD2 LEU 6 30.139 32.162 108.537 1.00 0.00

ATOM 1966 1HD2 LEU 6 31.213 32.002 108.630 1.00 0.00

ATOM 1967 2HD2 LEU 6 29.985 32.821 107.682 1.00 0.00

ATOM 1968 3HD2 LEU 6 29.670 31.182 108.442 1.00 0.00

ATOM 1969 C LEU 6 29.636 36.091 108.992 1.00 0.00

ATOM 1970 O LEU 6 28.968 36.189 107.965 1.00 0.00

ATOM 1971 N LEU 7 29.573 37.008 109.961 1.00 0.00

ATOM 1972 H LEU 7 30.320 36.973 110.639 1.00 0.00

ATOM 1973 CA LEU 7 28.612 38.092 109.933 1.00 0.00

ATOM 1974 HA LEU 7 27.637 37.688 109.661 1.00 0.00

ATOM 1975 CB LEU 7 28.431 38.575 111.369 1.00 0.00

ATOM 1976 HB1 LEU 7 28.368 39.659 111.462 1.00 0.00

ATOM 1977 HB2 LEU 7 29.368 38.334 111.870 1.00 0.00

ATOM 1978 CG LEU 7 27.209 38.022 112.099 1.00 0.00

ATOM 1979 HG LEU 7 27.287 38.142 113.180 1.00 0.00

ATOM 1980 CD1 LEU 7 26.116 39.014 111.711 1.00 0.00

ATOM 1981 1HD1 LEU 7 26.507 40.024 111.828 1.00 0.00

ATOM 1982 2HD1 LEU 7 25.225 38.824 112.310 1.00 0.00

ATOM 1983 3HD1 LEU 7 25.851 38.925 110.657 1.00 0.00

ATOM 1984 CD2 LEU 7 26.894 36.564 111.779 1.00 0.00

ATOM 1985 1HD2 LEU 7 26.080 36.257 112.428 1.00 0.00

ATOM 1986 2HD2 LEU 7 27.733 35.941 112.088 1.00 0.00

ATOM 1987 3HD2 LEU 7 26.511 36.439 110.766 1.00 0.00

ATOM 1988 C LEU 7 28.962 39.171 108.918 1.00 0.00

ATOM 1989 O LEU 7 28.295 40.195 108.783 1.00 0.00

ATOM 1990 N ILE 8 29.907 38.897 108.018 1.00 0.00

ATOM 1991 H ILE 8 30.412 38.039 108.186 1.00 0.00

ATOM 1992 CA ILE 8 29.950 39.513 106.706 1.00 0.00

ATOM 1993 HA ILE 8 29.365 40.423 106.567 1.00 0.00

ATOM 1994 CB ILE 8 31.381 39.993 106.485 1.00 0.00

ATOM 1995 HB ILE 8 32.007 39.111 106.354 1.00 0.00

ATOM 1996 CG2 ILE 8 31.382 40.681 105.124 1.00 0.00

ATOM 1997 1HG2 ILE 8 32.373 41.010 104.811 1.00 0.00

ATOM 1998 2HG2 ILE 8 30.701 41.528 105.039 1.00 0.00

ATOM 1999 3HG2 ILE 8 31.177 39.920 104.372 1.00 0.00

ATOM 2000 CG1 ILE 8 31.912 40.883 107.607 1.00 0.00

ATOM 2001 1HG1 ILE 8 31.379 41.833 107.663 1.00 0.00

ATOM 2002 2HG1 ILE 8 31.898 40.411 108.590 1.00 0.00

ATOM 2003 CD ILE 8 33.399 41.212 107.524 1.00 0.00

ATOM 2004 HD1 ILE 8 34.011 40.344 107.279 1.00 0.00

ATOM 2005 HD2 ILE 8 33.709 41.586 108.500 1.00 0.00

ATOM 2006 HD3 ILE 8 33.618 42.007 106.811 1.00 0.00

ATOM 2007 C ILE 8 29.350 38.553 105.689 1.00 0.00

ATOM 2008 O ILE 8 28.335 38.952 105.122 1.00 0.00

ATOM 2009 N VAL 9 29.868 37.352 105.429 1.00 0.00

ATOM 2010 H VAL 9 30.687 37.063 105.946 1.00 0.00

ATOM 2011 CA VAL 9 29.352 36.577 104.318 1.00 0.00

ATOM 2012 HA VAL 9 29.402 37.211 103.433 1.00 0.00

ATOM 2013 CB VAL 9 30.181 35.329 104.032 1.00 0.00

ATOM 2014 HB VAL 9 29.833 34.892 103.096 1.00 0.00

ATOM 2015 CG1 VAL 9 31.631 35.700 103.736 1.00 0.00

ATOM 2016 1HG1 VAL 9 32.058 35.923 104.714 1.00 0.00

ATOM 2017 2HG1 VAL 9 31.561 36.492 102.991 1.00 0.00

ATOM 2018 3HG1 VAL 9 32.147 34.894 103.213 1.00 0.00

ATOM 2019 CG2 VAL 9 30.133 34.349 105.202 1.00 0.00

ATOM 2020 1HG2 VAL 9 31.014 33.708 105.174 1.00 0.00

ATOM 2021 2HG2 VAL 9 29.222 33.752 105.173 1.00 0.00

ATOM 2022 3HG2 VAL 9 30.063 34.869 106.157 1.00 0.00

ATOM 2023 C VAL 9 27.863 36.261 104.310 1.00 0.00

ATOM 2024 O VAL 9 27.223 36.286 103.261 1.00 0.00

ATOM 2025 N VAL 10 27.270 36.053 105.487 1.00 0.00

ATOM 2026 H VAL 10 27.760 36.354 106.316 1.00 0.00

ATOM 2027 CA VAL 10 25.830 35.897 105.557 1.00 0.00

ATOM 2028 HA VAL 10 25.513 35.282 104.715 1.00 0.00

ATOM 2029 CB VAL 10 25.531 35.051 106.790 1.00 0.00

ATOM 2030 HB VAL 10 25.847 35.533 107.715 1.00 0.00

ATOM 2031 CG1 VAL 10 24.047 34.692 106.792 1.00 0.00

ATOM 2032 1HG1 VAL 10 23.435 35.568 106.996 1.00 0.00

ATOM 2033 2HG1 VAL 10 23.863 33.996 107.610 1.00 0.00

ATOM 2034 3HG1 VAL 10 23.751 34.093 105.930 1.00 0.00

ATOM 2035 CG2 VAL 10 26.236 33.698 106.745 1.00 0.00

ATOM 2036 1HG2 VAL 10 27.321 33.793 106.694 1.00 0.00

ATOM 2037 2HG2 VAL 10 25.958 33.250 105.792 1.00 0.00

ATOM 2038 3HG2 VAL 10 25.965 33.014 107.547 1.00 0.00

ATOM 2039 C VAL 10 25.041 37.189 105.401 1.00 0.00

ATOM 2040 O VAL 10 23.889 37.158 104.974 1.00 0.00

ATOM 2041 N ALA 11 25.636 38.336 105.738 1.00 0.00

ATOM 2042 H ALA 11 26.613 38.405 105.986 1.00 0.00

ATOM 2043 CA ALA 11 24.997 39.617 105.513 1.00 0.00

ATOM 2044 HA ALA 11 23.969 39.564 105.873 1.00 0.00

ATOM 2045 CB ALA 11 25.703 40.722 106.292 1.00 0.00

ATOM 2046 HB1 ALA 11 26.713 40.859 105.906 1.00 0.00

ATOM 2047 HB2 ALA 11 25.825 40.406 107.328 1.00 0.00

ATOM 2048 HB3 ALA 11 25.075 41.613 106.308 1.00 0.00

ATOM 2049 C ALA 11 25.009 40.002 104.039 1.00 0.00

ATOM 2050 O ALA 11 23.955 40.189 103.438 1.00 0.00

ATOM 2051 N ILE 12 26.111 39.855 103.298 1.00 0.00

ATOM 2052 H ILE 12 26.942 39.486 103.735 1.00 0.00

ATOM 2053 CA ILE 12 26.300 40.345 101.947 1.00 0.00

ATOM 2054 HA ILE 12 25.677 41.235 101.898 1.00 0.00

ATOM 2055 CB ILE 12 27.725 40.797 101.633 1.00 0.00

ATOM 2056 HB ILE 12 27.831 41.348 100.699 1.00 0.00

ATOM 2057 CG2 ILE 12 28.218 41.801 102.671 1.00 0.00

ATOM 2058 1HG2 ILE 12 28.117 41.384 103.672 1.00 0.00

ATOM 2059 2HG2 ILE 12 27.556 42.667 102.652 1.00 0.00

ATOM 2060 3HG2 ILE 12 29.208 42.200 102.448 1.00 0.00

ATOM 2061 CG1 ILE 12 28.613 39.560 101.555 1.00 0.00

ATOM 2062 1HG1 ILE 12 28.545 39.069 102.526 1.00 0.00

ATOM 2063 2HG1 ILE 12 28.224 38.921 100.762 1.00 0.00

ATOM 2064 CD ILE 12 30.107 39.770 101.323 1.00 0.00

ATOM 2065 HD1 ILE 12 30.318 40.270 100.378 1.00 0.00

ATOM 2066 HD2 ILE 12 30.514 38.769 101.188 1.00 0.00

ATOM 2067 HD3 ILE 12 30.680 40.192 102.149 1.00 0.00

ATOM 2068 C ILE 12 25.801 39.399 100.862 1.00 0.00

ATOM 2069 O ILE 12 25.826 39.775 99.693 1.00 0.00

ATOM 2070 N ILE 13 25.417 38.150 101.134 1.00 0.00

ATOM 2071 H ILE 13 25.365 37.908 102.112 1.00 0.00

ATOM 2072 CA ILE 13 24.944 37.163 100.182 1.00 0.00

ATOM 2073 HA ILE 13 25.681 37.134 99.379 1.00 0.00

ATOM 2074 CB ILE 13 24.903 35.784 100.832 1.00 0.00

ATOM 2075 HB ILE 13 25.915 35.630 101.207 1.00 0.00

ATOM 2076 CG2 ILE 13 23.811 35.650 101.890 1.00 0.00

ATOM 2077 1HG2 ILE 13 24.054 34.828 102.563 1.00 0.00

ATOM 2078 2HG2 ILE 13 22.809 35.501 101.486 1.00 0.00

ATOM 2079 3HG2 ILE 13 23.936 36.539 102.509 1.00 0.00

ATOM 2080 CG1 ILE 13 24.737 34.709 99.761 1.00 0.00

ATOM 2081 1HG1 ILE 13 24.811 33.730 100.233 1.00 0.00

ATOM 2082 2HG1 ILE 13 23.800 34.784 99.209 1.00 0.00

ATOM 2083 CD ILE 13 25.883 34.610 98.757 1.00 0.00

ATOM 2084 HD1 ILE 13 26.818 34.404 99.276 1.00 0.00

ATOM 2085 HD2 ILE 13 26.071 35.489 98.142 1.00 0.00

ATOM 2086 HD3 ILE 13 25.782 33.824 98.009 1.00 0.00

ATOM 2087 C ILE 13 23.650 37.565 99.490 1.00 0.00

ATOM 2088 O ILE 13 23.509 37.254 98.309 1.00 0.00

ATOM 2089 N GLY 14 22.870 38.440 100.130 1.00 0.00

ATOM 2090 H GLY 14 23.055 38.629 101.105 1.00 0.00

ATOM 2091 CA GLY 14 21.665 38.992 99.545 1.00 0.00

ATOM 2092 HA1 GLY 14 21.065 38.229 99.050 1.00 0.00

ATOM 2093 HA2 GLY 14 21.106 39.500 100.331 1.00 0.00

ATOM 2094 C GLY 14 21.970 39.922 98.379 1.00 0.00

ATOM 2095 O GLY 14 21.360 39.809 97.318 1.00 0.00

ATOM 2096 N ILE 15 23.010 40.753 98.462 1.00 0.00

ATOM 2097 H ILE 15 23.494 40.695 99.345 1.00 0.00

ATOM 2098 CA ILE 15 23.290 41.768 97.463 1.00 0.00

ATOM 2099 HA ILE 15 22.326 42.174 97.160 1.00 0.00

ATOM 2100 CB ILE 15 23.930 43.011 98.073 1.00 0.00

ATOM 2101 HB ILE 15 23.298 43.407 98.867 1.00 0.00

ATOM 2102 CG2 ILE 15 25.214 42.786 98.865 1.00 0.00

ATOM 2103 1HG2 ILE 15 25.127 42.344 99.857 1.00 0.00

ATOM 2104 2HG2 ILE 15 25.566 43.794 99.084 1.00 0.00

ATOM 2105 3HG2 ILE 15 25.945 42.174 98.337 1.00 0.00

ATOM 2106 CG1 ILE 15 24.214 44.118 97.059 1.00 0.00

ATOM 2107 1HG1 ILE 15 24.691 44.989 97.505 1.00 0.00

ATOM 2108 2HG1 ILE 15 24.902 43.818 96.268 1.00 0.00

ATOM 2109 CD ILE 15 22.927 44.775 96.570 1.00 0.00

ATOM 2110 HD1 ILE 15 22.452 45.378 97.345 1.00 0.00

ATOM 2111 HD2 ILE 15 22.252 44.072 96.083 1.00 0.00

ATOM 2112 HD3 ILE 15 23.127 45.497 95.780 1.00 0.00

ATOM 2113 C ILE 15 24.141 41.352 96.271 1.00 0.00

ATOM 2114 O ILE 15 23.999 41.798 95.134 1.00 0.00

ATOM 2115 N LEU 16 24.900 40.267 96.438 1.00 0.00

ATOM 2116 H LEU 16 25.129 39.970 97.375 1.00 0.00

ATOM 2117 CA LEU 16 25.780 39.789 95.390 1.00 0.00

ATOM 2118 HA LEU 16 26.383 40.636 95.064 1.00 0.00

ATOM 2119 CB LEU 16 26.848 38.881 95.991 1.00 0.00

ATOM 2120 HB1 LEU 16 27.313 38.449 95.105 1.00 0.00

ATOM 2121 HB2 LEU 16 26.425 37.993 96.460 1.00 0.00

ATOM 2122 CG LEU 16 27.942 39.559 96.812 1.00 0.00

ATOM 2123 HG LEU 16 27.616 40.440 97.364 1.00 0.00

ATOM 2124 CD1 LEU 16 28.555 38.524 97.752 1.00 0.00

ATOM 2125 1HD1 LEU 16 27.899 38.084 98.503 1.00 0.00

ATOM 2126 2HD1 LEU 16 29.276 38.975 98.432 1.00 0.00

ATOM 2127 3HD1 LEU 16 29.172 37.832 97.179 1.00 0.00

ATOM 2128 CD2 LEU 16 29.010 40.116 95.875 1.00 0.00

ATOM 2129 1HD2 LEU 16 28.638 40.921 95.241 1.00 0.00

ATOM 2130 2HD2 LEU 16 29.475 39.288 95.341 1.00 0.00

ATOM 2131 3HD2 LEU 16 29.732 40.442 96.477 1.00 0.00

ATOM 2132 C LEU 16 25.227 39.315 94.053 1.00 0.00

ATOM 2133 O LEU 16 25.467 39.877 92.986 1.00 0.00

ATOM 2134 N ALA 17 24.293 38.362 94.072 1.00 0.00

ATOM 2135 H ALA 17 24.005 37.939 94.943 1.00 0.00

ATOM 2136 CA ALA 17 23.432 38.025 92.956 1.00 0.00

ATOM 2137 HA ALA 17 23.934 37.729 92.034 1.00 0.00

ATOM 2138 CB ALA 17 22.499 36.895 93.381 1.00 0.00

ATOM 2139 HB1 ALA 17 21.788 37.048 94.194 1.00 0.00

ATOM 2140 HB2 ALA 17 23.109 36.043 93.677 1.00 0.00

ATOM 2141 HB3 ALA 17 21.870 36.630 92.531 1.00 0.00

ATOM 2142 C ALA 17 22.497 39.151 92.541 1.00 0.00

ATOM 2143 O ALA 17 22.108 39.200 91.376 1.00 0.00

ATOM 2144 N ALA 18 22.102 40.062 93.433 1.00 0.00

ATOM 2145 H ALA 18 22.463 39.991 94.374 1.00 0.00

ATOM 2146 CA ALA 18 21.174 41.094 93.015 1.00 0.00

ATOM 2147 HA ALA 18 20.244 40.723 92.586 1.00 0.00

ATOM 2148 CB ALA 18 20.643 41.874 94.214 1.00 0.00

ATOM 2149 HB1 ALA 18 21.343 42.649 94.525 1.00 0.00

ATOM 2150 HB2 ALA 18 19.728 42.330 93.835 1.00 0.00

ATOM 2151 HB3 ALA 18 20.326 41.174 94.987 1.00 0.00

ATOM 2152 C ALA 18 21.760 41.999 91.940 1.00 0.00

ATOM 2153 O ALA 18 21.058 42.520 91.078 1.00 0.00

ATOM 2154 N ILE 19 23.065 42.274 91.938 1.00 0.00

ATOM 2155 H ILE 19 23.603 41.942 92.726 1.00 0.00

ATOM 2156 CA ILE 19 23.667 43.096 90.906 1.00 0.00

ATOM 2157 HA ILE 19 22.985 43.808 90.439 1.00 0.00

ATOM 2158 CB ILE 19 24.712 43.929 91.640 1.00 0.00

ATOM 2159 HB ILE 19 25.172 44.534 90.860 1.00 0.00

ATOM 2160 CG2 ILE 19 23.978 44.832 92.628 1.00 0.00

ATOM 2161 1HG2 ILE 19 23.583 44.266 93.471 1.00 0.00

ATOM 2162 2HG2 ILE 19 23.058 45.227 92.199 1.00 0.00

ATOM 2163 3HG2 ILE 19 24.587 45.637 93.041 1.00 0.00

ATOM 2164 CG1 ILE 19 25.866 43.152 92.269 1.00 0.00

ATOM 2165 1HG1 ILE 19 25.551 42.458 93.048 1.00 0.00

ATOM 2166 2HG1 ILE 19 26.388 42.606 91.483 1.00 0.00

ATOM 2167 CD ILE 19 26.820 44.065 93.033 1.00 0.00

ATOM 2168 HD1 ILE 19 26.278 44.532 93.855 1.00 0.00

ATOM 2169 HD2 ILE 19 27.275 44.865 92.449 1.00 0.00

ATOM 2170 HD3 ILE 19 27.663 43.460 93.368 1.00 0.00

ATOM 2171 C ILE 19 24.278 42.305 89.756 1.00 0.00

ATOM 2172 O ILE 19 24.485 42.749 88.629 1.00 0.00

ATOM 2173 N ALA 20 24.501 40.999 89.913 1.00 0.00

ATOM 2174 H ALA 20 24.248 40.646 90.823 1.00 0.00

ATOM 2175 CA ALA 20 25.045 40.101 88.914 1.00 0.00

ATOM 2176 HA ALA 20 25.865 40.744 88.595 1.00 0.00

ATOM 2177 CB ALA 20 25.752 38.909 89.552 1.00 0.00

ATOM 2178 HB1 ALA 20 26.464 39.379 90.232 1.00 0.00

ATOM 2179 HB2 ALA 20 25.038 38.296 90.102 1.00 0.00

ATOM 2180 HB3 ALA 20 26.249 38.361 88.752 1.00 0.00

ATOM 2181 C ALA 20 24.011 39.668 87.882 1.00 0.00

ATOM 2182 O ALA 20 24.278 39.615 86.684 1.00 0.00

ATOM 2183 N ILE 21 22.737 39.468 88.223 1.00 0.00

ATOM 2184 H ILE 21 22.583 39.360 89.214 1.00 0.00

ATOM 2185 CA ILE 21 21.740 38.878 87.350 1.00 0.00

ATOM 2186 HA ILE 21 22.086 38.177 86.590 1.00 0.00

ATOM 2187 CB ILE 21 20.779 37.990 88.136 1.00 0.00

ATOM 2188 HB ILE 21 20.342 38.601 88.924 1.00 0.00

ATOM 2189 CG2 ILE 21 19.715 37.369 87.234 1.00 0.00

ATOM 2190 1HG2 ILE 21 18.866 36.893 87.725 1.00 0.00

ATOM 2191 2HG2 ILE 21 20.279 36.628 86.667 1.00 0.00

ATOM 2192 3HG2 ILE 21 19.154 38.150 86.720 1.00 0.00

ATOM 2193 CG1 ILE 21 21.576 36.896 88.839 1.00 0.00

ATOM 2194 1HG1 ILE 21 22.047 36.199 88.145 1.00 0.00

ATOM 2195 2HG1 ILE 21 22.366 37.331 89.450 1.00 0.00

ATOM 2196 CD ILE 21 20.752 35.959 89.718 1.00 0.00

ATOM 2197 HD1 ILE 21 20.157 36.420 90.506 1.00 0.00

ATOM 2198 HD2 ILE 21 21.492 35.265 90.117 1.00 0.00

ATOM 2199 HD3 ILE 21 20.076 35.357 89.120 1.00 0.00

ATOM 2200 C ILE 21 21.112 39.895 86.408 1.00 0.00

ATOM 2201 O ILE 21 21.025 39.700 85.197 1.00 0.00

ATOM 2202 N PRO 22 20.939 41.135 86.867 1.00 0.00

ATOM 2203 CD PRO 22 20.771 41.589 88.233 1.00 0.00

ATOM 2204 HD1 PRO 22 21.648 42.106 88.624 1.00 0.00

ATOM 2205 HD2 PRO 22 20.571 40.757 88.908 1.00 0.00

ATOM 2206 CG PRO 22 19.638 42.607 88.135 1.00 0.00

ATOM 2207 HG1 PRO 22 19.610 43.202 89.044 1.00 0.00

ATOM 2208 HG2 PRO 22 18.676 42.105 88.033 1.00 0.00

ATOM 2209 CB PRO 22 20.077 43.348 86.874 1.00 0.00

ATOM 2210 HB1 PRO 22 20.994 43.930 86.963 1.00 0.00

ATOM 2211 HB2 PRO 22 19.207 43.815 86.413 1.00 0.00

ATOM 2212 CA PRO 22 20.423 42.161 85.982 1.00 0.00

ATOM 2213 HA PRO 22 19.463 41.917 85.527 1.00 0.00

ATOM 2214 C PRO 22 21.397 42.566 84.885 1.00 0.00

ATOM 2215 O PRO 22 21.002 42.944 83.785 1.00 0.00

ATOM 2216 N GLN 23 22.707 42.548 85.142 1.00 0.00

ATOM 2217 H GLN 23 22.960 42.228 86.065 1.00 0.00

ATOM 2218 CA GLN 23 23.732 42.982 84.213 1.00 0.00

ATOM 2219 HA GLN 23 23.314 43.837 83.683 1.00 0.00

ATOM 2220 CB GLN 23 25.023 43.451 84.879 1.00 0.00

ATOM 2221 HB1 GLN 23 24.730 44.102 85.703 1.00 0.00

ATOM 2222 HB2 GLN 23 25.620 44.102 84.238 1.00 0.00

ATOM 2223 CG GLN 23 25.902 42.314 85.387 1.00 0.00

ATOM 2224 HG1 GLN 23 25.285 41.615 85.953 1.00 0.00

ATOM 2225 HG2 GLN 23 26.293 41.880 84.471 1.00 0.00

ATOM 2226 CD GLN 23 27.086 42.774 86.226 1.00 0.00

ATOM 2227 OE1 GLN 23 28.219 42.745 85.754 1.00 0.00

ATOM 2228 NE2 GLN 23 26.971 43.311 87.441 1.00 0.00

ATOM 2229 1HE2 GLN 23 26.043 43.310 87.839 1.00 0.00

ATOM 2230 2HE2 GLN 23 27.754 43.792 87.864 1.00 0.00

ATOM 2231 C GLN 23 23.891 41.868 83.188 1.00 0.00

ATOM 2232 O GLN 23 24.119 42.140 82.012 1.00 0.00

ATOM 2233 N PHE 24 23.890 40.603 83.613 1.00 0.00

ATOM 2234 H PHE 24 24.006 40.448 84.604 1.00 0.00

ATOM 2235 CA PHE 24 23.778 39.440 82.754 1.00 0.00

ATOM 2236 HA PHE 24 24.627 39.459 82.071 1.00 0.00

ATOM 2237 CB PHE 24 23.782 38.133 83.542 1.00 0.00

ATOM 2238 HB1 PHE 24 23.234 38.247 84.475 1.00 0.00

ATOM 2239 HB2 PHE 24 24.748 37.908 83.994 1.00 0.00

ATOM 2240 CG PHE 24 23.403 36.910 82.740 1.00 0.00

ATOM 2241 CD1 PHE 24 22.132 36.338 82.875 1.00 0.00

ATOM 2242 HD1 PHE 24 21.515 36.857 83.593 1.00 0.00

ATOM 2243 CE1 PHE 24 21.801 35.113 82.281 1.00 0.00

ATOM 2244 HE1 PHE 24 20.859 34.613 82.451 1.00 0.00

ATOM 2245 CZ PHE 24 22.801 34.479 81.534 1.00 0.00

ATOM 2246 HZ PHE 24 22.701 33.534 81.019 1.00 0.00

ATOM 2247 CE2 PHE 24 24.057 35.081 81.387 1.00 0.00

ATOM 2248 HE2 PHE 24 24.822 34.656 80.761 1.00 0.00

ATOM 2249 CD2 PHE 24 24.373 36.341 81.907 1.00 0.00

ATOM 2250 HD2 PHE 24 25.353 36.790 81.827 1.00 0.00

ATOM 2251 C PHE 24 22.582 39.451 81.813 1.00 0.00

ATOM 2252 O PHE 24 22.848 39.258 80.629 1.00 0.00

ATOM 2253 N SER 25 21.344 39.606 82.287 1.00 0.00

ATOM 2254 H SER 25 21.244 39.770 83.279 1.00 0.00

ATOM 2255 CA SER 25 20.144 39.504 81.481 1.00 0.00

ATOM 2256 HA SER 25 20.275 38.519 81.035 1.00 0.00

ATOM 2257 CB SER 25 18.913 39.447 82.381 1.00 0.00

ATOM 2258 HB1 SER 25 19.158 38.692 83.129 1.00 0.00

ATOM 2259 HB2 SER 25 18.066 39.144 81.764 1.00 0.00

ATOM 2260 OG SER 25 18.672 40.719 82.939 1.00 0.00

ATOM 2261 HG SER 25 17.858 40.687 83.450 1.00 0.00

ATOM 2262 C SER 25 20.087 40.458 80.297 1.00 0.00

ATOM 2263 O SER 25 19.815 40.041 79.172 1.00 0.00

ATOM 2264 N ALA 26 20.449 41.710 80.585 1.00 0.00

ATOM 2265 H ALA 26 20.658 41.938 81.546 1.00 0.00

ATOM 2266 CA ALA 26 20.533 42.772 79.602 1.00 0.00

ATOM 2267 HA ALA 26 19.542 42.987 79.204 1.00 0.00

ATOM 2268 CB ALA 26 21.052 44.036 80.281 1.00 0.00

ATOM 2269 HB1 ALA 26 20.403 44.425 81.060 1.00 0.00

ATOM 2270 HB2 ALA 26 22.086 43.820 80.552 1.00 0.00

ATOM 2271 HB3 ALA 26 21.053 44.833 79.538 1.00 0.00

ATOM 2272 C ALA 26 21.461 42.351 78.471 1.00 0.00

ATOM 2273 O ALA 26 21.176 42.414 77.277 1.00 0.00

ATOM 2274 N ALA 27 22.680 41.938 78.820 1.00 0.00

ATOM 2275 H ALA 27 22.805 41.763 79.807 1.00 0.00

ATOM 2276 CA ALA 27 23.719 41.552 77.886 1.00 0.00

ATOM 2277 HA ALA 27 23.739 42.347 77.141 1.00 0.00

ATOM 2278 CB ALA 27 25.019 41.486 78.683 1.00 0.00

ATOM 2279 HB1 ALA 27 25.330 42.508 78.903 1.00 0.00

ATOM 2280 HB2 ALA 27 25.687 40.842 78.111 1.00 0.00

ATOM 2281 HB3 ALA 27 24.842 40.992 79.638 1.00 0.00

ATOM 2282 C ALA 27 23.423 40.297 77.078 1.00 0.00

ATOM 2283 O ALA 27 23.893 40.244 75.943 1.00 0.00

ATOM 2284 N ARG 28 22.641 39.378 77.649 1.00 0.00

ATOM 2285 H ARG 28 22.453 39.539 78.628 1.00 0.00

ATOM 2286 CA ARG 28 21.968 38.247 77.042 1.00 0.00

ATOM 2287 HA ARG 28 22.735 37.694 76.500 1.00 0.00

ATOM 2288 CB ARG 28 21.241 37.384 78.071 1.00 0.00

ATOM 2289 HB1 ARG 28 20.285 37.846 78.319 1.00 0.00

ATOM 2290 HB2 ARG 28 21.735 37.445 79.041 1.00 0.00

ATOM 2291 CG ARG 28 21.041 35.967 77.542 1.00 0.00

ATOM 2292 HG1 ARG 28 20.752 35.933 76.492 1.00 0.00

ATOM 2293 HG2 ARG 28 22.017 35.493 77.645 1.00 0.00

ATOM 2294 CD ARG 28 20.009 35.163 78.328 1.00 0.00

ATOM 2295 HD1 ARG 28 20.268 35.050 79.381 1.00 0.00

ATOM 2296 HD2 ARG 28 19.027 35.636 78.341 1.00 0.00

ATOM 2297 NE ARG 28 20.016 33.772 77.875 1.00 0.00

ATOM 2298 HE ARG 28 20.576 33.111 78.400 1.00 0.00

ATOM 2299 CZ ARG 28 19.191 33.277 76.946 1.00 0.00

ATOM 2300 NH1 ARG 28 18.396 34.069 76.214 1.00 0.00

ATOM 2301 1HH1 ARG 28 18.323 35.067 76.351 1.00 0.00

ATOM 2302 2HH1 ARG 28 17.870 33.603 75.488 1.00 0.00

ATOM 2303 NH2 ARG 28 19.214 31.975 76.643 1.00 0.00

ATOM 2304 1HH2 ARG 28 19.925 31.458 77.143 1.00 0.00

ATOM 2305 2HH2 ARG 28 18.512 31.597 76.022 1.00 0.00

ATOM 2306 C ARG 28 21.023 38.732 75.953 1.00 0.00

ATOM 2307 O ARG 28 21.148 38.287 74.815 1.00 0.00

ATOM 2308 N VAL 29 20.103 39.660 76.227 1.00 0.00

ATOM 2309 H VAL 29 19.752 39.720 77.172 1.00 0.00

ATOM 2310 CA VAL 29 19.229 40.297 75.261 1.00 0.00

ATOM 2311 HA VAL 29 18.677 39.513 74.743 1.00 0.00

ATOM 2312 CB VAL 29 18.252 41.253 75.939 1.00 0.00

ATOM 2313 HB VAL 29 18.748 42.047 76.498 1.00 0.00

ATOM 2314 CG1 VAL 29 17.408 42.039 74.940 1.00 0.00

ATOM 2315 1HG1 VAL 29 17.965 42.678 74.254 1.00 0.00

ATOM 2316 2HG1 VAL 29 16.759 42.724 75.486 1.00 0.00

ATOM 2317 3HG1 VAL 29 16.783 41.420 74.297 1.00 0.00

ATOM 2318 CG2 VAL 29 17.247 40.465 76.773 1.00 0.00

ATOM 2319 1HG2 VAL 29 16.511 40.072 76.073 1.00 0.00

ATOM 2320 2HG2 VAL 29 16.777 41.170 77.459 1.00 0.00

ATOM 2321 3HG2 VAL 29 17.790 39.607 77.170 1.00 0.00

ATOM 2322 C VAL 29 19.947 40.944 74.086 1.00 0.00

ATOM 2323 O VAL 29 19.815 40.540 72.936 1.00 0.00

ATOM 2324 N LYS 30 20.863 41.851 74.433 1.00 0.00

ATOM 2325 H LYS 30 20.935 42.035 75.423 1.00 0.00

ATOM 2326 CA LYS 30 21.684 42.582 73.489 1.00 0.00

ATOM 2327 HA LYS 30 20.967 43.096 72.847 1.00 0.00

ATOM 2328 CB LYS 30 22.461 43.627 74.284 1.00 0.00

ATOM 2329 HB1 LYS 30 23.293 44.079 73.745 1.00 0.00

ATOM 2330 HB2 LYS 30 22.819 43.183 75.212 1.00 0.00

ATOM 2331 CG LYS 30 21.542 44.781 74.673 1.00 0.00

ATOM 2332 HG1 LYS 30 21.458 45.490 73.850 1.00 0.00

ATOM 2333 HG2 LYS 30 20.569 44.388 74.967 1.00 0.00

ATOM 2334 CD LYS 30 22.137 45.428 75.921 1.00 0.00

ATOM 2335 HD1 LYS 30 22.292 44.652 76.670 1.00 0.00

ATOM 2336 HD2 LYS 30 23.060 45.933 75.651 1.00 0.00

ATOM 2337 CE LYS 30 21.106 46.403 76.481 1.00 0.00

ATOM 2338 HE1 LYS 30 20.187 45.825 76.576 1.00 0.00

ATOM 2339 HE2 LYS 30 21.394 46.678 77.496 1.00 0.00

ATOM 2340 NZ LYS 30 20.929 47.599 75.642 1.00 0.00

ATOM 2341 HZ1 LYS 30 20.506 48.384 76.115 1.00 0.00

ATOM 2342 HZ2 LYS 30 20.391 47.441 74.801 1.00 0.00

ATOM 2343 HZ3 LYS 30 21.819 47.996 75.378 1.00 0.00

ATOM 2344 C LYS 30 22.569 41.707 72.612 1.00 0.00

ATOM 2345 O LYS 30 22.916 42.123 71.508 1.00 0.00

ATOM 2346 N ALA 31 22.705 40.430 72.976 1.00 0.00

ATOM 2347 H ALA 31 22.313 40.173 73.871 1.00 0.00

ATOM 2348 CA ALA 31 23.419 39.421 72.220 1.00 0.00

ATOM 2349 HA ALA 31 24.210 39.886 71.630 1.00 0.00

ATOM 2350 CB ALA 31 24.056 38.445 73.204 1.00 0.00

ATOM 2351 HB1 ALA 31 24.760 37.748 72.749 1.00 0.00

ATOM 2352 HB2 ALA 31 24.580 39.088 73.911 1.00 0.00

ATOM 2353 HB3 ALA 31 23.275 37.883 73.717 1.00 0.00

ATOM 2354 C ALA 31 22.490 38.680 71.268 1.00 0.00

ATOM 2355 O ALA 31 22.683 38.601 70.057 1.00 0.00

ATOM 2356 N TYR 32 21.399 38.120 71.794 1.00 0.00

ATOM 2357 H TYR 32 21.270 38.153 72.796 1.00 0.00

ATOM 2358 CA TYR 32 20.373 37.454 71.018 1.00 0.00

ATOM 2359 HA TYR 32 20.892 36.656 70.488 1.00 0.00

ATOM 2360 CB TYR 32 19.274 36.827 71.874 1.00 0.00

ATOM 2361 HB1 TYR 32 18.489 37.498 72.223 1.00 0.00

ATOM 2362 HB2 TYR 32 19.757 36.420 72.761 1.00 0.00

ATOM 2363 CG TYR 32 18.585 35.653 71.222 1.00 0.00

ATOM 2364 CD1 TYR 32 18.994 34.330 71.435 1.00 0.00

ATOM 2365 HD1 TYR 32 19.832 34.161 72.094 1.00 0.00

ATOM 2366 CE1 TYR 32 18.394 33.246 70.782 1.00 0.00

ATOM 2367 HE1 TYR 32 18.680 32.213 70.898 1.00 0.00

ATOM 2368 CZ TYR 32 17.347 33.526 69.882 1.00 0.00

ATOM 2369 OH TYR 32 16.760 32.546 69.138 1.00 0.00

ATOM 2370 HH TYR 32 17.033 31.730 69.560 1.00 0.00

ATOM 2371 CE2 TYR 32 16.868 34.838 69.698 1.00 0.00

ATOM 2372 HE2 TYR 32 16.056 34.962 68.998 1.00 0.00

ATOM 2373 CD2 TYR 32 17.509 35.892 70.359 1.00 0.00

ATOM 2374 HD2 TYR 32 17.200 36.912 70.194 1.00 0.00

ATOM 2375 C TYR 32 19.728 38.350 69.970 1.00 0.00

ATOM 2376 O TYR 32 19.503 37.851 68.870 1.00 0.00

ATOM 2377 N ASN 33 19.431 39.627 70.222 1.00 0.00

ATOM 2378 H ASN 33 19.671 40.015 71.123 1.00 0.00

ATOM 2379 CA ASN 33 18.964 40.561 69.217 1.00 0.00

ATOM 2380 HA ASN 33 17.975 40.294 68.844 1.00 0.00

ATOM 2381 CB ASN 33 18.713 41.889 69.925 1.00 0.00

ATOM 2382 HB1 ASN 33 18.121 41.630 70.794 1.00 0.00

ATOM 2383 HB2 ASN 33 19.651 42.389 70.165 1.00 0.00

ATOM 2384 CG ASN 33 17.875 42.864 69.111 1.00 0.00

ATOM 2385 OD1 ASN 33 16.647 42.892 69.156 1.00 0.00

ATOM 2386 ND2 ASN 33 18.509 43.674 68.260 1.00 0.00

ATOM 2387 1HD2 ASN 33 19.498 43.586 68.078 1.00 0.00

ATOM 2388 2HD2 ASN 33 17.911 44.313 67.755 1.00 0.00

ATOM 2389 C ASN 33 19.959 40.642 68.068 1.00 0.00

ATOM 2390 O ASN 33 19.607 40.536 66.895 1.00 0.00

ATOM 2391 N SER 34 21.235 40.833 68.405 1.00 0.00

ATOM 2392 H SER 34 21.391 40.840 69.403 1.00 0.00

ATOM 2393 CA SER 34 22.336 40.734 67.466 1.00 0.00

ATOM 2394 HA SER 34 22.198 41.468 66.673 1.00 0.00

ATOM 2395 CB SER 34 23.603 41.115 68.226 1.00 0.00

ATOM 2396 HB1 SER 34 24.360 41.219 67.450 1.00 0.00

ATOM 2397 HB2 SER 34 24.009 40.365 68.906 1.00 0.00

ATOM 2398 OG SER 34 23.623 42.416 68.770 1.00 0.00

ATOM 2399 HG SER 34 24.312 42.341 69.433 1.00 0.00

ATOM 2400 C SER 34 22.638 39.387 66.826 1.00 0.00

ATOM 2401 O SER 34 23.392 39.381 65.856 1.00 0.00

ATOM 2402 N ALA 35 22.037 38.302 67.320 1.00 0.00

ATOM 2403 H ALA 35 21.541 38.292 68.200 1.00 0.00

ATOM 2404 CA ALA 35 22.105 37.035 66.618 1.00 0.00

ATOM 2405 HA ALA 35 22.950 37.074 65.932 1.00 0.00

ATOM 2406 CB ALA 35 22.387 35.896 67.593 1.00 0.00

ATOM 2407 HB1 ALA 35 21.483 35.526 68.077 1.00 0.00

ATOM 2408 HB2 ALA 35 22.894 35.051 67.128 1.00 0.00

ATOM 2409 HB3 ALA 35 23.110 36.297 68.304 1.00 0.00

ATOM 2410 C ALA 35 20.904 36.651 65.765 1.00 0.00

ATOM 2411 O ALA 35 21.083 36.146 64.659 1.00 0.00

ATOM 2412 N ALA 36 19.671 36.827 66.246 1.00 0.00

ATOM 2413 H ALA 36 19.575 37.236 67.164 1.00 0.00

ATOM 2414 CA ALA 36 18.445 36.527 65.534 1.00 0.00

ATOM 2415 HA ALA 36 18.539 35.810 64.719 1.00 0.00

ATOM 2416 CB ALA 36 17.532 35.780 66.502 1.00 0.00

ATOM 2417 HB1 ALA 36 17.440 36.385 67.403 1.00 0.00

ATOM 2418 HB2 ALA 36 18.063 34.852 66.714 1.00 0.00

ATOM 2419 HB3 ALA 36 16.531 35.472 66.210 1.00 0.00

ATOM 2420 C ALA 36 17.933 37.784 64.846 1.00 0.00

ATOM 2421 O ALA 36 17.797 37.682 63.629 1.00 0.00

ATOM 2422 N SER 37 17.590 38.863 65.554 1.00 0.00

ATOM 2423 H SER 37 17.793 39.002 66.534 1.00 0.00

ATOM 2424 CA SER 37 16.877 39.962 64.935 1.00 0.00

ATOM 2425 HA SER 37 15.934 39.637 64.497 1.00 0.00

ATOM 2426 CB SER 37 16.554 40.903 66.093 1.00 0.00

ATOM 2427 HB1 SER 37 17.493 41.279 66.499 1.00 0.00

ATOM 2428 HB2 SER 37 15.863 41.705 65.849 1.00 0.00

ATOM 2429 OG SER 37 16.037 40.059 67.097 1.00 0.00

ATOM 2430 HG SER 37 15.681 39.280 66.663 1.00 0.00

ATOM 2431 C SER 37 17.669 40.708 63.871 1.00 0.00

ATOM 2432 O SER 37 17.028 41.386 63.070 1.00 0.00

ATOM 2433 N SER 38 18.992 40.532 63.840 1.00 0.00

ATOM 2434 H SER 38 19.419 39.943 64.540 1.00 0.00

ATOM 2435 CA SER 38 19.994 41.098 62.959 1.00 0.00

ATOM 2436 HA SER 38 19.839 42.174 62.899 1.00 0.00

ATOM 2437 CB SER 38 21.386 40.870 63.543 1.00 0.00

ATOM 2438 HB1 SER 38 21.521 41.351 64.512 1.00 0.00

ATOM 2439 HB2 SER 38 22.206 41.220 62.917 1.00 0.00

ATOM 2440 OG SER 38 21.582 39.486 63.719 1.00 0.00

ATOM 2441 HG SER 38 22.386 39.411 64.238 1.00 0.00

ATOM 2442 C SER 38 19.907 40.513 61.556 1.00 0.00

ATOM 2443 O SER 38 20.298 41.131 60.569 1.00 0.00

ATOM 2444 N ASP 39 19.393 39.284 61.473 1.00 0.00

ATOM 2445 H ASP 39 19.318 38.687 62.285 1.00 0.00

ATOM 2446 CA ASP 39 19.116 38.663 60.194 1.00 0.00

ATOM 2447 HA ASP 39 19.989 38.973 59.621 1.00 0.00

ATOM 2448 CB ASP 39 18.969 37.160 60.409 1.00 0.00

ATOM 2449 HB1 ASP 39 19.854 36.872 60.977 1.00 0.00

ATOM 2450 HB2 ASP 39 18.109 37.096 61.074 1.00 0.00

ATOM 2451 CG ASP 39 18.780 36.439 59.081 1.00 0.00

ATOM 2452 OD1 ASP 39 19.762 36.181 58.352 1.00 0.00

ATOM 2453 OD2 ASP 39 17.626 36.064 58.785 1.00 0.00

ATOM 2454 C ASP 39 17.888 39.241 59.505 1.00 0.00

ATOM 2455 O ASP 39 17.913 39.484 58.300 1.00 0.00

ATOM 2456 N LEU 40 16.835 39.555 60.263 1.00 0.00

ATOM 2457 H LEU 40 16.836 39.253 61.227 1.00 0.00

ATOM 2458 CA LEU 40 15.673 40.186 59.668 1.00 0.00

ATOM 2459 HA LEU 40 15.530 39.653 58.728 1.00 0.00

ATOM 2460 CB LEU 40 14.521 40.087 60.663 1.00 0.00

ATOM 2461 HB1 LEU 40 14.752 40.587 61.604 1.00 0.00

ATOM 2462 HB2 LEU 40 14.466 39.047 60.987 1.00 0.00

ATOM 2463 CG LEU 40 13.185 40.610 60.143 1.00 0.00

ATOM 2464 HG LEU 40 13.272 41.695 60.080 1.00 0.00

ATOM 2465 CD1 LEU 40 12.615 40.029 58.852 1.00 0.00

ATOM 2466 1HD1 LEU 40 13.218 40.444 58.045 1.00 0.00

ATOM 2467 2HD1 LEU 40 11.628 40.447 58.657 1.00 0.00

ATOM 2468 3HD1 LEU 40 12.533 38.943 58.901 1.00 0.00

ATOM 2469 CD2 LEU 40 12.104 40.347 61.188 1.00 0.00

ATOM 2470 1HD2 LEU 40 11.113 40.688 60.892 1.00 0.00

ATOM 2471 2HD2 LEU 40 12.436 40.740 62.149 1.00 0.00

ATOM 2472 3HD2 LEU 40 11.930 39.301 61.438 1.00 0.00

ATOM 2473 C LEU 40 16.063 41.596 59.246 1.00 0.00

ATOM 2474 O LEU 40 15.611 42.184 58.267 1.00 0.00

ATOM 2475 N ARG 41 16.960 42.225 60.009 1.00 0.00

ATOM 2476 H ARG 41 17.282 41.729 60.827 1.00 0.00

ATOM 2477 CA ARG 41 17.527 43.525 59.707 1.00 0.00

ATOM 2478 HA ARG 41 16.707 44.238 59.630 1.00 0.00

ATOM 2479 CB ARG 41 18.545 44.018 60.733 1.00 0.00

ATOM 2480 HB1 ARG 41 19.538 43.607 60.551 1.00 0.00

ATOM 2481 HB2 ARG 41 18.188 43.673 61.703 1.00 0.00

ATOM 2482 CG ARG 41 18.568 45.531 60.929 1.00 0.00

ATOM 2483 HG1 ARG 41 17.589 45.920 60.648 1.00 0.00

ATOM 2484 HG2 ARG 41 18.708 45.745 61.986 1.00 0.00

ATOM 2485 CD ARG 41 19.611 46.271 60.099 1.00 0.00

ATOM 2486 HD1 ARG 41 20.607 45.842 60.212 1.00 0.00

ATOM 2487 HD2 ARG 41 19.428 46.136 59.033 1.00 0.00

ATOM 2488 NE ARG 41 19.647 47.685 60.474 1.00 0.00

ATOM 2489 HE ARG 41 19.141 47.979 61.297 1.00 0.00

ATOM 2490 CZ ARG 41 20.287 48.639 59.783 1.00 0.00

ATOM 2491 NH1 ARG 41 21.229 48.418 58.856 1.00 0.00

ATOM 2492 1HH1 ARG 41 21.422 47.470 58.568 1.00 0.00

ATOM 2493 2HH1 ARG 41 21.939 49.095 58.616 1.00 0.00

ATOM 2494 NH2 ARG 41 20.075 49.919 60.085 1.00 0.00

ATOM 2495 1HH2 ARG 41 19.310 50.129 60.714 1.00 0.00

ATOM 2496 2HH2 ARG 41 20.413 50.642 59.462 1.00 0.00

ATOM 2497 C ARG 41 18.148 43.462 58.320 1.00 0.00

ATOM 2498 O ARG 41 17.806 44.240 57.433 1.00 0.00

ATOM 2499 N ASN 42 19.029 42.474 58.145 1.00 0.00

ATOM 2500 H ASN 42 19.262 41.914 58.953 1.00 0.00

ATOM 2501 CA ASN 42 19.695 42.163 56.897 1.00 0.00

ATOM 2502 HA ASN 42 20.223 43.078 56.632 1.00 0.00

ATOM 2503 CB ASN 42 20.774 41.143 57.252 1.00 0.00

ATOM 2504 HB1 ASN 42 21.405 41.560 58.037 1.00 0.00

ATOM 2505 HB2 ASN 42 20.246 40.278 57.654 1.00 0.00

ATOM 2506 CG ASN 42 21.621 40.777 56.040 1.00 0.00

ATOM 2507 OD1 ASN 42 22.361 41.590 55.492 1.00 0.00

ATOM 2508 ND2 ASN 42 21.371 39.598 55.467 1.00 0.00

ATOM 2509 1HD2 ASN 42 20.690 39.060 55.982 1.00 0.00

ATOM 2510 2HD2 ASN 42 21.787 39.264 54.609 1.00 0.00

ATOM 2511 C ASN 42 18.742 41.850 55.750 1.00 0.00

ATOM 2512 O ASN 42 18.985 42.293 54.631 1.00 0.00

ATOM 2513 N LEU 43 17.641 41.131 55.975 1.00 0.00

ATOM 2514 H LEU 43 17.632 40.777 56.920 1.00 0.00

ATOM 2515 CA LEU 43 16.529 40.936 55.063 1.00 0.00

ATOM 2516 HA LEU 43 16.909 40.419 54.183 1.00 0.00

ATOM 2517 CB LEU 43 15.455 40.050 55.689 1.00 0.00

ATOM 2518 HB1 LEU 43 14.954 40.657 56.443 1.00 0.00

ATOM 2519 HB2 LEU 43 16.072 39.228 56.050 1.00 0.00

ATOM 2520 CG LEU 43 14.359 39.563 54.745 1.00 0.00

ATOM 2521 HG LEU 43 14.259 40.256 53.910 1.00 0.00

ATOM 2522 CD1 LEU 43 14.703 38.209 54.133 1.00 0.00

ATOM 2523 1HD1 LEU 43 13.873 37.890 53.502 1.00 0.00

ATOM 2524 2HD1 LEU 43 14.698 37.488 54.950 1.00 0.00

ATOM 2525 3HD1 LEU 43 15.633 38.258 53.566 1.00 0.00

ATOM 2526 CD2 LEU 43 13.006 39.484 55.448 1.00 0.00

ATOM 2527 1HD2 LEU 43 13.149 38.943 56.362 1.00 0.00

ATOM 2528 2HD2 LEU 43 12.250 39.047 54.797 1.00 0.00

ATOM 2529 3HD2 LEU 43 12.616 40.467 55.710 1.00 0.00

ATOM 2530 C LEU 43 15.984 42.253 54.531 1.00 0.00

ATOM 2531 O LEU 43 15.948 42.419 53.314 1.00 0.00

ATOM 2532 N LYS 44 15.685 43.168 55.456 1.00 0.00

ATOM 2533 H LYS 44 15.905 42.910 56.407 1.00 0.00

ATOM 2534 CA LYS 44 15.120 44.489 55.268 1.00 0.00

ATOM 2535 HA LYS 44 14.358 44.397 54.496 1.00 0.00

ATOM 2536 CB LYS 44 14.482 44.970 56.569 1.00 0.00

ATOM 2537 HB1 LYS 44 14.228 46.030 56.550 1.00 0.00

ATOM 2538 HB2 LYS 44 15.214 44.843 57.367 1.00 0.00

ATOM 2539 CG LYS 44 13.224 44.216 56.992 1.00 0.00

ATOM 2540 HG1 LYS 44 12.423 44.559 56.337 1.00 0.00

ATOM 2541 HG2 LYS 44 13.255 43.133 56.877 1.00 0.00

ATOM 2542 CD LYS 44 12.949 44.509 58.464 1.00 0.00

ATOM 2543 HD1 LYS 44 13.768 44.095 59.052 1.00 0.00

ATOM 2544 HD2 LYS 44 12.804 45.581 58.596 1.00 0.00

ATOM 2545 CE LYS 44 11.707 43.824 59.027 1.00 0.00

ATOM 2546 HE1 LYS 44 10.796 44.132 58.512 1.00 0.00

ATOM 2547 HE2 LYS 44 11.822 42.754 58.858 1.00 0.00

ATOM 2548 NZ LYS 44 11.581 44.191 60.445 1.00 0.00

ATOM 2549 HZ1 LYS 44 12.354 43.835 60.990 1.00 0.00

ATOM 2550 HZ2 LYS 44 10.703 43.856 60.819 1.00 0.00

ATOM 2551 HZ3 LYS 44 11.518 45.198 60.480 1.00 0.00

ATOM 2552 C LYS 44 16.056 45.490 54.603 1.00 0.00

ATOM 2553 O LYS 44 15.714 46.259 53.708 1.00 0.00

ATOM 2554 N THR 45 17.335 45.527 54.981 1.00 0.00

ATOM 2555 H THR 45 17.537 45.079 55.862 1.00 0.00

ATOM 2556 CA THR 45 18.368 46.440 54.535 1.00 0.00

ATOM 2557 HA THR 45 17.997 47.458 54.413 1.00 0.00

ATOM 2558 CB THR 45 19.480 46.424 55.580 1.00 0.00

ATOM 2559 HB THR 45 19.840 45.400 55.678 1.00 0.00

ATOM 2560 CG2 THR 45 20.661 47.344 55.284 1.00 0.00

ATOM 2561 1HG2 THR 45 21.190 47.490 56.225 1.00 0.00

ATOM 2562 2HG2 THR 45 20.394 48.267 54.768 1.00 0.00

ATOM 2563 3HG2 THR 45 21.323 46.750 54.655 1.00 0.00

ATOM 2564 OG1 THR 45 18.822 46.822 56.761 1.00 0.00

ATOM 2565 HG1 THR 45 18.939 47.771 56.688 1.00 0.00

ATOM 2566 C THR 45 18.944 46.001 53.196 1.00 0.00

ATOM 2567 O THR 45 19.396 46.855 52.436 1.00 0.00

ATOM 2568 N ALA 46 18.930 44.695 52.919 1.00 0.00

ATOM 2569 H ALA 46 18.717 43.999 53.618 1.00 0.00

ATOM 2570 CA ALA 46 19.559 44.144 51.735 1.00 0.00

ATOM 2571 HA ALA 46 19.688 44.979 51.047 1.00 0.00

ATOM 2572 CB ALA 46 20.932 43.777 52.288 1.00 0.00

ATOM 2573 HB1 ALA 46 20.820 42.907 52.935 1.00 0.00

ATOM 2574 HB2 ALA 46 21.389 44.564 52.888 1.00 0.00

ATOM 2575 HB3 ALA 46 21.568 43.458 51.502 1.00 0.00

ATOM 2576 C ALA 46 18.926 43.032 50.907 1.00 0.00

ATOM 2577 O ALA 46 19.182 43.050 49.706 1.00 0.00

ATOM 2578 N LEU 47 17.993 42.173 51.325 1.00 0.00

ATOM 2579 H LEU 47 17.768 42.219 52.307 1.00 0.00

ATOM 2580 CA LEU 47 17.301 41.189 50.515 1.00 0.00

ATOM 2581 HA LEU 47 17.962 40.891 49.702 1.00 0.00

ATOM 2582 CB LEU 47 16.868 39.914 51.234 1.00 0.00

ATOM 2583 HB1 LEU 47 16.223 39.353 50.558 1.00 0.00

ATOM 2584 HB2 LEU 47 16.201 40.106 52.074 1.00 0.00

ATOM 2585 CG LEU 47 17.903 38.885 51.680 1.00 0.00

ATOM 2586 HG LEU 47 17.341 38.166 52.276 1.00 0.00

ATOM 2587 CD1 LEU 47 18.484 38.140 50.482 1.00 0.00

ATOM 2588 1HD1 LEU 47 17.779 37.888 49.692 1.00 0.00

ATOM 2589 2HD1 LEU 47 18.987 37.225 50.794 1.00 0.00

ATOM 2590 3HD1 LEU 47 19.110 38.834 49.921 1.00 0.00

ATOM 2591 CD2 LEU 47 19.077 39.306 52.560 1.00 0.00

ATOM 2592 1HD2 LEU 47 18.656 39.591 53.525 1.00 0.00

ATOM 2593 2HD2 LEU 47 19.687 40.115 52.159 1.00 0.00

ATOM 2594 3HD2 LEU 47 19.746 38.494 52.751 1.00 0.00

ATOM 2595 C LEU 47 16.090 41.797 49.823 1.00 0.00

ATOM 2596 O LEU 47 15.903 41.546 48.635 1.00 0.00

ATOM 2597 N GLU 48 15.390 42.748 50.447 1.00 0.00

ATOM 2598 H GLU 48 15.416 42.823 51.454 1.00 0.00

ATOM 2599 CA GLU 48 14.355 43.590 49.880 1.00 0.00

ATOM 2600 HA GLU 48 13.645 43.057 49.249 1.00 0.00

ATOM 2601 CB GLU 48 13.589 44.330 50.974 1.00 0.00

ATOM 2602 HB1 GLU 48 12.918 44.995 50.431 1.00 0.00

ATOM 2603 HB2 GLU 48 14.287 44.974 51.510 1.00 0.00

ATOM 2604 CG GLU 48 12.827 43.458 51.969 1.00 0.00

ATOM 2605 HG1 GLU 48 12.386 42.627 51.421 1.00 0.00

ATOM 2606 HG2 GLU 48 13.652 43.082 52.574 1.00 0.00

ATOM 2607 CD GLU 48 11.728 44.150 52.763 1.00 0.00

ATOM 2608 OE1 GLU 48 12.014 45.297 53.167 1.00 0.00

ATOM 2609 OE2 GLU 48 10.534 43.803 52.636 1.00 0.00

ATOM 2610 C GLU 48 14.873 44.621 48.888 1.00 0.00

ATOM 2611 O GLU 48 14.154 44.934 47.942 1.00 0.00

ATOM 2612 N SER 49 16.076 45.170 49.066 1.00 0.00

ATOM 2613 H SER 49 16.662 44.849 49.824 1.00 0.00

ATOM 2614 CA SER 49 16.553 46.228 48.199 1.00 0.00

ATOM 2615 HA SER 49 15.743 46.957 48.165 1.00 0.00

ATOM 2616 CB SER 49 17.881 46.805 48.679 1.00 0.00

ATOM 2617 HB1 SER 49 18.702 46.268 48.204 1.00 0.00

ATOM 2618 HB2 SER 49 17.858 46.854 49.768 1.00 0.00

ATOM 2619 OG SER 49 17.983 48.107 48.150 1.00 0.00

ATOM 2620 HG SER 49 18.092 48.750 48.855 1.00 0.00

ATOM 2621 C SER 49 16.644 45.743 46.758 1.00 0.00

ATOM 2622 O SER 49 16.298 46.340 45.741 1.00 0.00

ATOM 2623 N ALA 50 16.972 44.465 46.555 1.00 0.00

ATOM 2624 H ALA 50 17.354 43.955 47.337 1.00 0.00

ATOM 2625 CA ALA 50 17.022 43.699 45.326 1.00 0.00

ATOM 2626 HA ALA 50 17.728 44.256 44.712 1.00 0.00

ATOM 2627 CB ALA 50 17.645 42.340 45.634 1.00 0.00

ATOM 2628 HB1 ALA 50 17.313 41.828 46.536 1.00 0.00

ATOM 2629 HB2 ALA 50 17.463 41.714 44.760 1.00 0.00

ATOM 2630 HB3 ALA 50 18.731 42.395 45.709 1.00 0.00

ATOM 2631 C ALA 50 15.701 43.591 44.579 1.00 0.00

ATOM 2632 O ALA 50 15.798 43.455 43.361 1.00 0.00

ATOM 2633 N PHE 51 14.570 43.654 45.287 1.00 0.00

ATOM 2634 H PHE 51 14.546 43.754 46.292 1.00 0.00

ATOM 2635 CA PHE 51 13.295 43.606 44.600 1.00 0.00

ATOM 2636 HA PHE 51 13.325 42.911 43.761 1.00 0.00

ATOM 2637 CB PHE 51 12.161 43.096 45.484 1.00 0.00

ATOM 2638 HB1 PHE 51 11.285 42.967 44.847 1.00 0.00

ATOM 2639 HB2 PHE 51 11.877 43.923 46.134 1.00 0.00

ATOM 2640 CG PHE 51 12.354 41.780 46.198 1.00 0.00

ATOM 2641 CD1 PHE 51 12.788 40.665 45.471 1.00 0.00

ATOM 2642 HD1 PHE 51 13.018 40.789 44.422 1.00 0.00

ATOM 2643 CE1 PHE 51 12.950 39.442 46.132 1.00 0.00

ATOM 2644 HE1 PHE 51 13.304 38.577 45.590 1.00 0.00

ATOM 2645 CZ PHE 51 12.757 39.407 47.519 1.00 0.00

ATOM 2646 HZ PHE 51 12.893 38.480 48.054 1.00 0.00

ATOM 2647 CE2 PHE 51 12.278 40.481 48.279 1.00 0.00

ATOM 2648 HE2 PHE 51 12.192 40.421 49.354 1.00 0.00

ATOM 2649 CD2 PHE 51 12.053 41.662 47.560 1.00 0.00

ATOM 2650 HD2 PHE 51 11.549 42.509 47.999 1.00 0.00

ATOM 2651 C PHE 51 12.886 44.936 43.984 1.00 0.00

ATOM 2652 O PHE 51 12.329 44.986 42.891 1.00 0.00

ATOM 2653 N ALA 52 13.251 46.024 44.666 1.00 0.00

ATOM 2654 H ALA 52 13.778 46.009 45.528 1.00 0.00

ATOM 2655 CA ALA 52 12.775 47.354 44.341 1.00 0.00

ATOM 2656 HA ALA 52 11.715 47.318 44.093 1.00 0.00

ATOM 2657 CB ALA 52 13.086 48.248 45.538 1.00 0.00

ATOM 2658 HB1 ALA 52 14.165 48.288 45.687 1.00 0.00

ATOM 2659 HB2 ALA 52 12.728 49.259 45.342 1.00 0.00

ATOM 2660 HB3 ALA 52 12.485 47.886 46.372 1.00 0.00

ATOM 2661 C ALA 52 13.494 47.793 43.072 1.00 0.00

ATOM 2662 O ALA 52 12.922 48.433 42.194 1.00 0.00

ATOM 2663 N ASP 53 14.691 47.259 42.819 1.00 0.00

ATOM 2664 H ASP 53 15.053 46.646 43.536 1.00 0.00

ATOM 2665 CA ASP 53 15.555 47.587 41.703 1.00 0.00

ATOM 2666 HA ASP 53 15.675 48.670 41.692 1.00 0.00

ATOM 2667 CB ASP 53 16.888 46.882 41.932 1.00 0.00

ATOM 2668 HB1 ASP 53 17.241 47.197 42.914 1.00 0.00

ATOM 2669 HB2 ASP 53 16.615 45.848 42.140 1.00 0.00

ATOM 2670 CG ASP 53 17.828 47.153 40.763 1.00 0.00

ATOM 2671 OD1 ASP 53 18.531 48.183 40.688 1.00 0.00

ATOM 2672 OD2 ASP 53 17.992 46.292 39.872 1.00 0.00

ATOM 2673 C ASP 53 14.957 47.183 40.361 1.00 0.00

ATOM 2674 O ASP 53 14.808 47.892 39.368 1.00 0.00

ATOM 2675 N ASP 54 14.259 46.050 40.286 1.00 0.00

ATOM 2676 H ASP 54 14.350 45.474 41.110 1.00 0.00

ATOM 2677 CA ASP 54 13.553 45.414 39.190 1.00 0.00

ATOM 2678 HA ASP 54 14.165 45.389 38.289 1.00 0.00

ATOM 2679 CB ASP 54 13.326 43.941 39.519 1.00 0.00

ATOM 2680 HB1 ASP 54 14.109 43.569 40.179 1.00 0.00

ATOM 2681 HB2 ASP 54 12.489 43.955 40.216 1.00 0.00

ATOM 2682 CG ASP 54 13.077 43.026 38.328 1.00 0.00

ATOM 2683 OD1 ASP 54 12.042 42.326 38.289 1.00 0.00

ATOM 2684 OD2 ASP 54 13.992 42.953 37.480 1.00 0.00

ATOM 2685 C ASP 54 12.316 46.138 38.676 1.00 0.00

ATOM 2686 O ASP 54 11.821 45.818 37.599 1.00 0.00

ATOM 2687 N GLN 55 11.779 47.083 39.452 1.00 0.00

ATOM 2688 H GLN 55 12.238 47.176 40.346 1.00 0.00

ATOM 2689 CA GLN 55 10.781 48.019 38.973 1.00 0.00

ATOM 2690 HA GLN 55 10.678 47.850 37.901 1.00 0.00

ATOM 2691 CB GLN 55 9.427 47.686 39.593 1.00 0.00

ATOM 2692 HB1 GLN 55 9.176 46.639 39.427 1.00 0.00

ATOM 2693 HB2 GLN 55 8.754 48.387 39.099 1.00 0.00

ATOM 2694 CG GLN 55 9.328 48.086 41.062 1.00 0.00

ATOM 2695 HG1 GLN 55 10.180 47.719 41.634 1.00 0.00

ATOM 2696 HG2 GLN 55 9.383 49.175 41.055 1.00 0.00

ATOM 2697 CD GLN 55 8.050 47.643 41.760 1.00 0.00

ATOM 2698 OE1 GLN 55 7.325 46.798 41.240 1.00 0.00

ATOM 2699 NE2 GLN 55 7.774 48.127 42.972 1.00 0.00

ATOM 2700 1HE2 GLN 55 8.536 48.678 43.344 1.00 0.00

ATOM 2701 2HE2 GLN 55 6.867 48.007 43.401 1.00 0.00

ATOM 2702 C GLN 55 11.231 49.447 39.244 1.00 0.00

ATOM 2703 O GLN 55 10.403 50.353 39.214 1.00 0.00

ATOM 2704 N THR 56 12.501 49.642 39.603 1.00 0.00

ATOM 2705 H THR 56 13.017 48.790 39.429 1.00 0.00

ATOM 2706 CA THR 56 13.056 50.878 40.118 1.00 0.00

ATOM 2707 HA THR 56 14.004 50.558 40.549 1.00 0.00

ATOM 2708 CB THR 56 13.445 51.811 38.975 1.00 0.00

ATOM 2709 HB THR 56 13.893 52.736 39.336 1.00 0.00

ATOM 2710 CG2 THR 56 14.413 51.137 38.005 1.00 0.00

ATOM 2711 1HG2 THR 56 14.956 51.875 37.415 1.00 0.00

ATOM 2712 2HG2 THR 56 13.987 50.376 37.350 1.00 0.00

ATOM 2713 3HG2 THR 56 15.167 50.583 38.562 1.00 0.00

ATOM 2714 OG1 THR 56 12.342 52.066 38.134 1.00 0.00

ATOM 2715 HG1 THR 56 11.535 51.710 38.513 1.00 0.00

ATOM 2716 C THR 56 12.304 51.503 41.286 1.00 0.00

ATOM 2717 O THR 56 12.536 52.670 41.590 1.00 0.00

ATOM 2718 N TYR 57 11.367 50.863 41.991 1.00 0.00

ATOM 2719 H TYR 57 11.293 49.857 41.946 1.00 0.00

ATOM 2720 CA TYR 57 10.533 51.431 43.031 1.00 0.00

ATOM 2721 HA TYR 57 10.983 52.381 43.317 1.00 0.00

ATOM 2722 CB TYR 57 9.183 51.845 42.453 1.00 0.00

ATOM 2723 HB1 TYR 57 8.605 50.930 42.336 1.00 0.00

ATOM 2724 HB2 TYR 57 9.365 52.285 41.481 1.00 0.00

ATOM 2725 CG TYR 57 8.371 52.791 43.306 1.00 0.00

ATOM 2726 CD1 TYR 57 8.900 54.041 43.646 1.00 0.00

ATOM 2727 HD1 TYR 57 9.876 54.324 43.282 1.00 0.00

ATOM 2728 CE1 TYR 57 8.226 54.898 44.524 1.00 0.00

ATOM 2729 HE1 TYR 57 8.740 55.784 44.870 1.00 0.00

ATOM 2730 CZ TYR 57 6.937 54.580 44.995 1.00 0.00

ATOM 2731 OH TYR 57 6.215 55.439 45.769 1.00 0.00

ATOM 2732 HH TYR 57 5.391 55.030 46.047 1.00 0.00

ATOM 2733 CE2 TYR 57 6.325 53.413 44.496 1.00 0.00

ATOM 2734 HE2 TYR 57 5.310 53.246 44.826 1.00 0.00

ATOM 2735 CD2 TYR 57 7.067 52.505 43.731 1.00 0.00

ATOM 2736 HD2 TYR 57 6.707 51.490 43.665 1.00 0.00

ATOM 2737 C TYR 57 10.303 50.519 44.229 1.00 0.00

ATOM 2738 O TYR 57 10.149 49.317 44.026 1.00 0.00

ATOM 2739 N PRO 58 10.335 51.013 45.469 1.00 0.00

ATOM 2740 CD PRO 58 10.643 52.358 45.912 1.00 0.00

ATOM 2741 HD1 PRO 58 9.775 53.013 46.001 1.00 0.00

ATOM 2742 HD2 PRO 58 11.363 52.864 45.271 1.00 0.00

ATOM 2743 CG PRO 58 11.258 52.107 47.286 1.00 0.00

ATOM 2744 HG1 PRO 58 11.229 52.968 47.952 1.00 0.00

ATOM 2745 HG2 PRO 58 12.301 51.791 47.288 1.00 0.00

ATOM 2746 CB PRO 58 10.269 51.075 47.818 1.00 0.00

ATOM 2747 HB1 PRO 58 9.340 51.609 48.019 1.00 0.00

ATOM 2748 HB2 PRO 58 10.584 50.424 48.631 1.00 0.00

ATOM 2749 CA PRO 58 9.986 50.189 46.608 1.00 0.00

ATOM 2750 HA PRO 58 10.705 49.376 46.704 1.00 0.00

ATOM 2751 C PRO 58 8.612 49.532 46.584 1.00 0.00

ATOM 2752 O PRO 58 7.691 49.938 45.878 1.00 0.00

ATOM 2753 N PRO 59 8.453 48.496 47.411 1.00 0.00

ATOM 2754 CD PRO 59 9.404 47.917 48.339 1.00 0.00

ATOM 2755 HD1 PRO 59 9.751 48.649 49.068 1.00 0.00

ATOM 2756 HD2 PRO 59 10.261 47.506 47.806 1.00 0.00

ATOM 2757 CG PRO 59 8.686 46.817 49.116 1.00 0.00

ATOM 2758 HG1 PRO 59 8.304 47.284 50.023 1.00 0.00

ATOM 2759 HG2 PRO 59 9.306 45.985 49.449 1.00 0.00

ATOM 2760 CB PRO 59 7.528 46.466 48.185 1.00 0.00

ATOM 2761 HB1 PRO 59 6.650 46.001 48.633 1.00 0.00

ATOM 2762 HB2 PRO 59 7.894 45.803 47.401 1.00 0.00

ATOM 2763 CA PRO 59 7.180 47.829 47.595 1.00 0.00

ATOM 2764 HA PRO 59 6.584 47.613 46.709 1.00 0.00

ATOM 2765 C PRO 59 6.239 48.631 48.483 1.00 0.00

ATOM 2766 O PRO 59 6.658 49.540 49.197 1.00 0.00

ATOM 2767 N GLU 60 4.936 48.346 48.447 1.00 0.00

ATOM 2768 H GLU 60 4.701 47.465 48.012 1.00 0.00

ATOM 2769 CA GLU 60 3.914 48.988 49.250 1.00 0.00

ATOM 2770 HA GLU 60 3.036 48.898 48.611 1.00 0.00

ATOM 2771 CB GLU 60 3.689 48.314 50.600 1.00 0.00

ATOM 2772 HB1 GLU 60 2.647 48.550 50.812 1.00 0.00

ATOM 2773 HB2 GLU 60 4.336 48.799 51.332 1.00 0.00

ATOM 2774 CG GLU 60 3.794 46.794 50.686 1.00 0.00

ATOM 2775 HG1 GLU 60 4.863 46.615 50.682 1.00 0.00

ATOM 2776 HG2 GLU 60 3.387 46.394 51.615 1.00 0.00

ATOM 2777 CD GLU 60 3.108 46.130 49.499 1.00 0.00

ATOM 2778 OE1 GLU 60 3.651 45.818 48.418 1.00 0.00

ATOM 2779 OE2 GLU 60 1.877 45.922 49.531 1.00 0.00

ATOM 2780 C GLU 60 4.036 50.502 49.340 1.00 0.00

ATOM 2781 O GLU 60 3.943 51.101 50.410 1.00 0.00

ATOM 2782 N SER 61 4.280 51.081 48.162 1.00 0.00

ATOM 2783 H SER 61 4.205 50.528 47.321 1.00 0.00

ATOM 2784 CA SER 61 4.578 52.489 47.986 1.00 0.00

ATOM 2785 HA SER 61 4.142 53.018 48.833 1.00 0.00

ATOM 2786 CB SER 61 6.075 52.780 47.948 1.00 0.00

ATOM 2787 HB1 SER 61 6.427 52.375 46.999 1.00 0.00

ATOM 2788 HB2 SER 61 6.157 53.866 47.892 1.00 0.00

ATOM 2789 OG SER 61 6.725 52.303 49.105 1.00 0.00

ATOM 2790 HG SER 61 6.947 51.406 48.846 1.00 0.00

ATOM 2791 C SER 61 3.811 53.125 46.836 1.00 0.00

ATOM 2792 OC1 SER 61 2.911 52.438 46.306 1.00 0.00

ATOM 2793 OC2 SER 61 4.066 54.316 46.556 1.00 0.00

ATOM 2794 N PHE 1 37.967 40.601 121.667 1.00 0.00

ATOM 2795 H1 PHE 1 38.170 41.183 122.468 1.00 0.00

ATOM 2796 H2 PHE 1 37.895 41.191 120.850 1.00 0.00

ATOM 2797 H3 PHE 1 38.692 39.900 121.664 1.00 0.00

ATOM 2798 CA PHE 1 36.662 39.926 121.721 1.00 0.00

ATOM 2799 HA PHE 1 35.955 40.653 122.119 1.00 0.00

ATOM 2800 CB PHE 1 36.272 39.447 120.324 1.00 0.00

ATOM 2801 HB1 PHE 1 35.343 38.892 120.457 1.00 0.00

ATOM 2802 HB2 PHE 1 37.082 38.808 119.974 1.00 0.00

ATOM 2803 CG PHE 1 36.114 40.544 119.298 1.00 0.00

ATOM 2804 CD1 PHE 1 35.125 41.524 119.447 1.00 0.00

ATOM 2805 HD1 PHE 1 34.482 41.523 120.314 1.00 0.00

ATOM 2806 CE1 PHE 1 34.960 42.433 118.395 1.00 0.00

ATOM 2807 HE1 PHE 1 34.135 43.121 118.359 1.00 0.00

ATOM 2808 CZ PHE 1 35.784 42.415 117.263 1.00 0.00

ATOM 2809 HZ PHE 1 35.759 43.234 116.559 1.00 0.00

ATOM 2810 CE2 PHE 1 36.883 41.549 117.238 1.00 0.00

ATOM 2811 HE2 PHE 1 37.531 41.637 116.378 1.00 0.00

ATOM 2812 CD2 PHE 1 36.972 40.528 118.192 1.00 0.00

ATOM 2813 HD2 PHE 1 37.712 39.751 118.073 1.00 0.00

ATOM 2814 C PHE 1 36.703 38.851 122.798 1.00 0.00

ATOM 2815 O PHE 1 37.754 38.261 123.036 1.00 0.00

ATOM 2816 N THR 2 35.594 38.491 123.447 1.00 0.00

ATOM 2817 H THR 2 34.731 38.972 123.238 1.00 0.00

ATOM 2818 CA THR 2 35.459 37.427 124.422 1.00 0.00

ATOM 2819 HA THR 2 36.451 37.366 124.870 1.00 0.00

ATOM 2820 CB THR 2 34.534 37.848 125.562 1.00 0.00

ATOM 2821 HB THR 2 34.873 38.778 126.016 1.00 0.00

ATOM 2822 CG2 THR 2 33.070 38.064 125.188 1.00 0.00

ATOM 2823 1HG2 THR 2 33.004 39.041 124.715 1.00 0.00

ATOM 2824 2HG2 THR 2 32.478 38.276 126.078 1.00 0.00

ATOM 2825 3HG2 THR 2 32.671 37.202 124.652 1.00 0.00

ATOM 2826 OG1 THR 2 34.638 36.935 126.630 1.00 0.00

ATOM 2827 HG1 THR 2 33.769 36.750 126.995 1.00 0.00

ATOM 2828 C THR 2 35.110 36.101 123.762 1.00 0.00

ATOM 2829 O THR 2 34.749 36.044 122.588 1.00 0.00

ATOM 2830 N LEU 3 35.237 34.982 124.480 1.00 0.00

ATOM 2831 H LEU 3 35.440 35.056 125.466 1.00 0.00

ATOM 2832 CA LEU 3 35.016 33.633 123.998 1.00 0.00

ATOM 2833 HA LEU 3 35.783 33.477 123.242 1.00 0.00

ATOM 2834 CB LEU 3 35.435 32.526 124.962 1.00 0.00

ATOM 2835 HB1 LEU 3 36.462 32.663 125.300 1.00 0.00

ATOM 2836 HB2 LEU 3 35.388 31.628 124.346 1.00 0.00

ATOM 2837 CG LEU 3 34.529 32.202 126.147 1.00 0.00

ATOM 2838 HG LEU 3 33.540 31.873 125.828 1.00 0.00

ATOM 2839 CD1 LEU 3 35.102 31.028 126.936 1.00 0.00

ATOM 2840 1HD1 LEU 3 34.977 30.085 126.404 1.00 0.00

ATOM 2841 2HD1 LEU 3 34.491 30.962 127.836 1.00 0.00

ATOM 2842 3HD1 LEU 3 36.138 31.173 127.243 1.00 0.00

ATOM 2843 CD2 LEU 3 34.400 33.451 127.016 1.00 0.00

ATOM 2844 1HD2 LEU 3 35.350 33.785 127.433 1.00 0.00

ATOM 2845 2HD2 LEU 3 33.619 33.373 127.773 1.00 0.00

ATOM 2846 3HD2 LEU 3 34.052 34.304 126.436 1.00 0.00

ATOM 2847 C LEU 3 33.662 33.384 123.347 1.00 0.00

ATOM 2848 O LEU 3 33.515 32.806 122.273 1.00 0.00

ATOM 2849 N ILE 4 32.641 34.053 123.888 1.00 0.00

ATOM 2850 H ILE 4 32.818 34.712 124.632 1.00 0.00

ATOM 2851 CA ILE 4 31.292 34.052 123.358 1.00 0.00

ATOM 2852 HA ILE 4 31.036 33.045 123.027 1.00 0.00

ATOM 2853 CB ILE 4 30.209 34.236 124.417 1.00 0.00

ATOM 2854 HB ILE 4 29.312 33.840 123.942 1.00 0.00

ATOM 2855 CG2 ILE 4 30.416 33.236 125.550 1.00 0.00

ATOM 2856 1HG2 ILE 4 29.572 33.255 126.240 1.00 0.00

ATOM 2857 2HG2 ILE 4 31.261 33.577 126.148 1.00 0.00

ATOM 2858 3HG2 ILE 4 30.632 32.256 125.133 1.00 0.00

ATOM 2859 CG1 ILE 4 30.023 35.669 124.908 1.00 0.00

ATOM 2860 1HG1 ILE 4 30.804 35.841 125.649 1.00 0.00

ATOM 2861 2HG1 ILE 4 30.050 36.380 124.085 1.00 0.00

ATOM 2862 CD ILE 4 28.762 35.988 125.706 1.00 0.00

ATOM 2863 HD1 ILE 4 28.880 36.995 126.108 1.00 0.00

ATOM 2864 HD2 ILE 4 28.768 35.301 126.552 1.00 0.00

ATOM 2865 HD3 ILE 4 27.855 36.010 125.101 1.00 0.00

ATOM 2866 C ILE 4 31.062 34.935 122.140 1.00 0.00

ATOM 2867 O ILE 4 30.042 34.753 121.479 1.00 0.00

ATOM 2868 N GLU 5 31.981 35.810 121.728 1.00 0.00

ATOM 2869 H GLU 5 32.867 35.759 122.208 1.00 0.00

ATOM 2870 CA GLU 5 31.913 36.425 120.415 1.00 0.00

ATOM 2871 HA GLU 5 30.955 36.174 119.960 1.00 0.00

ATOM 2872 CB GLU 5 31.969 37.950 120.412 1.00 0.00

ATOM 2873 HB1 GLU 5 31.910 38.268 119.371 1.00 0.00

ATOM 2874 HB2 GLU 5 32.896 38.299 120.867 1.00 0.00

ATOM 2875 CG GLU 5 30.720 38.410 121.159 1.00 0.00

ATOM 2876 HG1 GLU 5 29.804 38.176 120.634 1.00 0.00

ATOM 2877 HG2 GLU 5 30.597 37.888 122.107 1.00 0.00

ATOM 2878 CD GLU 5 30.721 39.919 121.365 1.00 0.00

ATOM 2879 OE1 GLU 5 31.017 40.418 122.471 1.00 0.00

ATOM 2880 OE2 GLU 5 30.259 40.643 120.457 1.00 0.00

ATOM 2881 C GLU 5 32.978 35.869 119.482 1.00 0.00

ATOM 2882 O GLU 5 33.358 36.422 118.452 1.00 0.00

ATOM 2883 N LEU 6 33.397 34.642 119.801 1.00 0.00

ATOM 2884 H LEU 6 32.983 34.157 120.585 1.00 0.00

ATOM 2885 CA LEU 6 34.359 33.924 118.989 1.00 0.00

ATOM 2886 HA LEU 6 34.381 34.144 117.921 1.00 0.00

ATOM 2887 CB LEU 6 35.708 34.150 119.666 1.00 0.00

ATOM 2888 HB1 LEU 6 36.514 33.572 119.213 1.00 0.00

ATOM 2889 HB2 LEU 6 35.624 33.738 120.672 1.00 0.00

ATOM 2890 CG LEU 6 36.155 35.608 119.705 1.00 0.00

ATOM 2891 HG LEU 6 35.403 36.255 120.157 1.00 0.00

ATOM 2892 CD1 LEU 6 37.475 35.760 120.456 1.00 0.00

ATOM 2893 1HD1 LEU 6 37.726 36.812 120.324 1.00 0.00

ATOM 2894 2HD1 LEU 6 38.224 35.154 119.946 1.00 0.00

ATOM 2895 3HD1 LEU 6 37.418 35.486 121.509 1.00 0.00

ATOM 2896 CD2 LEU 6 36.394 36.140 118.295 1.00 0.00

ATOM 2897 1HD2 LEU 6 35.501 36.049 117.677 1.00 0.00

ATOM 2898 2HD2 LEU 6 37.232 35.610 117.842 1.00 0.00

ATOM 2899 3HD2 LEU 6 36.594 37.211 118.334 1.00 0.00

ATOM 2900 C LEU 6 33.989 32.462 118.786 1.00 0.00

ATOM 2901 O LEU 6 34.099 31.968 117.666 1.00 0.00

ATOM 2902 N LEU 7 33.473 31.738 119.780 1.00 0.00

ATOM 2903 H LEU 7 33.531 32.261 120.642 1.00 0.00

ATOM 2904 CA LEU 7 33.295 30.301 119.708 1.00 0.00

ATOM 2905 HA LEU 7 33.718 29.871 118.799 1.00 0.00

ATOM 2906 CB LEU 7 33.877 29.672 120.971 1.00 0.00

ATOM 2907 HB1 LEU 7 33.472 28.678 121.157 1.00 0.00

ATOM 2908 HB2 LEU 7 33.713 30.270 121.867 1.00 0.00

ATOM 2909 CG LEU 7 35.384 29.439 120.924 1.00 0.00

ATOM 2910 HG LEU 7 35.532 28.947 121.887 1.00 0.00

ATOM 2911 CD1 LEU 7 35.925 28.772 119.662 1.00 0.00

ATOM 2912 1HD1 LEU 7 36.897 28.314 119.830 1.00 0.00

ATOM 2913 2HD1 LEU 7 35.961 29.508 118.860 1.00 0.00

ATOM 2914 3HD1 LEU 7 35.133 28.057 119.444 1.00 0.00

ATOM 2915 CD2 LEU 7 36.248 30.683 121.096 1.00 0.00

ATOM 2916 1HD2 LEU 7 36.178 31.424 120.300 1.00 0.00

ATOM 2917 2HD2 LEU 7 37.244 30.301 120.870 1.00 0.00

ATOM 2918 3HD2 LEU 7 36.165 31.118 122.092 1.00 0.00

ATOM 2919 C LEU 7 31.812 29.971 119.623 1.00 0.00

ATOM 2920 O LEU 7 31.493 28.785 119.593 1.00 0.00

ATOM 2921 N ILE 8 30.930 30.963 119.482 1.00 0.00

ATOM 2922 H ILE 8 31.275 31.905 119.602 1.00 0.00

ATOM 2923 CA ILE 8 29.520 30.785 119.200 1.00 0.00

ATOM 2924 HA ILE 8 29.253 29.956 119.854 1.00 0.00

ATOM 2925 CB ILE 8 28.778 32.009 119.726 1.00 0.00

ATOM 2926 HB ILE 8 29.256 32.381 120.632 1.00 0.00

ATOM 2927 CG2 ILE 8 28.890 33.209 118.790 1.00 0.00

ATOM 2928 1HG2 ILE 8 28.205 33.114 117.948 1.00 0.00

ATOM 2929 2HG2 ILE 8 29.924 33.419 118.517 1.00 0.00

ATOM 2930 3HG2 ILE 8 28.658 34.145 119.297 1.00 0.00

ATOM 2931 CG1 ILE 8 27.330 31.624 120.023 1.00 0.00

ATOM 2932 1HG1 ILE 8 26.724 31.252 119.198 1.00 0.00

ATOM 2933 2HG1 ILE 8 27.340 30.740 120.659 1.00 0.00

ATOM 2934 CD ILE 8 26.489 32.601 120.839 1.00 0.00

ATOM 2935 HD1 ILE 8 26.979 32.616 121.812 1.00 0.00

ATOM 2936 HD2 ILE 8 25.456 32.275 120.953 1.00 0.00

ATOM 2937 HD3 ILE 8 26.474 33.601 120.405 1.00 0.00

ATOM 2938 C ILE 8 29.193 30.452 117.750 1.00 0.00

ATOM 2939 O ILE 8 28.158 29.864 117.445 1.00 0.00

ATOM 2940 N VAL 9 30.133 30.711 116.838 1.00 0.00

ATOM 2941 H VAL 9 30.912 31.270 117.154 1.00 0.00

ATOM 2942 CA VAL 9 30.044 30.425 115.420 1.00 0.00

ATOM 2943 HA VAL 9 28.990 30.613 115.226 1.00 0.00

ATOM 2944 CB VAL 9 30.840 31.481 114.658 1.00 0.00

ATOM 2945 HB VAL 9 30.646 32.466 115.083 1.00 0.00

ATOM 2946 CG1 VAL 9 32.341 31.239 114.787 1.00 0.00

ATOM 2947 1HG1 VAL 9 32.688 31.176 115.818 1.00 0.00

ATOM 2948 2HG1 VAL 9 32.824 32.078 114.287 1.00 0.00

ATOM 2949 3HG1 VAL 9 32.694 30.333 114.296 1.00 0.00

ATOM 2950 CG2 VAL 9 30.503 31.687 113.185 1.00 0.00

ATOM 2951 1HG2 VAL 9 31.005 32.609 112.888 1.00 0.00

ATOM 2952 2HG2 VAL 9 29.436 31.907 113.216 1.00 0.00

ATOM 2953 3HG2 VAL 9 30.650 30.803 112.564 1.00 0.00

ATOM 2954 C VAL 9 30.250 28.994 114.942 1.00 0.00

ATOM 2955 O VAL 9 30.054 28.715 113.762 1.00 0.00

ATOM 2956 N VAL 10 30.828 28.059 115.700 1.00 0.00

ATOM 2957 H VAL 10 30.954 28.279 116.677 1.00 0.00

ATOM 2958 CA VAL 10 31.446 26.835 115.229 1.00 0.00

ATOM 2959 HA VAL 10 32.159 27.177 114.479 1.00 0.00

ATOM 2960 CB VAL 10 32.196 26.180 116.386 1.00 0.00

ATOM 2961 HB VAL 10 32.554 25.233 115.985 1.00 0.00

ATOM 2962 CG1 VAL 10 33.528 26.898 116.583 1.00 0.00

ATOM 2963 1HG1 VAL 10 33.359 27.953 116.796 1.00 0.00

ATOM 2964 2HG1 VAL 10 34.164 26.842 115.699 1.00 0.00

ATOM 2965 3HG1 VAL 10 34.126 26.513 117.409 1.00 0.00

ATOM 2966 CG2 VAL 10 31.422 25.817 117.651 1.00 0.00

ATOM 2967 1HG2 VAL 10 32.017 25.175 118.300 1.00 0.00

ATOM 2968 2HG2 VAL 10 30.550 25.252 117.321 1.00 0.00

ATOM 2969 3HG2 VAL 10 31.218 26.787 118.103 1.00 0.00

ATOM 2970 C VAL 10 30.581 25.823 114.491 1.00 0.00

ATOM 2971 O VAL 10 31.041 25.133 113.584 1.00 0.00

ATOM 2972 N ALA 11 29.287 25.660 114.774 1.00 0.00

ATOM 2973 H ALA 11 28.862 26.393 115.323 1.00 0.00

ATOM 2974 CA ALA 11 28.350 24.958 113.919 1.00 0.00

ATOM 2975 HA ALA 11 28.856 24.076 113.527 1.00 0.00

ATOM 2976 CB ALA 11 27.168 24.491 114.763 1.00 0.00

ATOM 2977 HB1 ALA 11 26.539 23.800 114.202 1.00 0.00

ATOM 2978 HB2 ALA 11 26.615 25.412 114.938 1.00 0.00

ATOM 2979 HB3 ALA 11 27.591 24.117 115.695 1.00 0.00

ATOM 2980 C ALA 11 27.986 25.732 112.660 1.00 0.00

ATOM 2981 O ALA 11 27.825 25.073 111.636 1.00 0.00

ATOM 2982 N ILE 12 27.958 27.067 112.678 1.00 0.00

ATOM 2983 H ILE 12 28.355 27.497 113.500 1.00 0.00

ATOM 2984 CA ILE 12 27.628 27.969 111.592 1.00 0.00

ATOM 2985 HA ILE 12 26.708 27.566 111.171 1.00 0.00

ATOM 2986 CB ILE 12 27.337 29.404 112.024 1.00 0.00

ATOM 2987 HB ILE 12 28.307 29.861 112.220 1.00 0.00

ATOM 2988 CG2 ILE 12 26.626 30.177 110.917 1.00 0.00

ATOM 2989 1HG2 ILE 12 25.647 29.842 110.574 1.00 0.00

ATOM 2990 2HG2 ILE 12 27.238 30.349 110.031 1.00 0.00

ATOM 2991 3HG2 ILE 12 26.440 31.133 111.405 1.00 0.00

ATOM 2992 CG1 ILE 12 26.470 29.585 113.268 1.00 0.00

ATOM 2993 1HG1 ILE 12 25.430 29.333 113.063 1.00 0.00

ATOM 2994 2HG1 ILE 12 26.819 28.903 114.042 1.00 0.00

ATOM 2995 CD ILE 12 26.397 30.973 113.896 1.00 0.00

ATOM 2996 HD1 ILE 12 26.059 30.833 114.923 1.00 0.00

ATOM 2997 HD2 ILE 12 25.559 31.487 113.424 1.00 0.00

ATOM 2998 HD3 ILE 12 27.325 31.532 113.775 1.00 0.00

ATOM 2999 C ILE 12 28.681 27.889 110.496 1.00 0.00

ATOM 3000 O ILE 12 28.358 27.578 109.352 1.00 0.00

ATOM 3001 N ILE 13 29.945 28.082 110.880 1.00 0.00

ATOM 3002 H ILE 13 30.145 28.428 111.808 1.00 0.00

ATOM 3003 CA ILE 13 31.047 27.752 109.999 1.00 0.00

ATOM 3004 HA ILE 13 30.843 28.324 109.094 1.00 0.00

ATOM 3005 CB ILE 13 32.374 28.368 110.433 1.00 0.00

ATOM 3006 HB ILE 13 32.151 29.352 110.840 1.00 0.00

ATOM 3007 CG2 ILE 13 33.024 27.575 111.564 1.00 0.00

ATOM 3008 1HG2 ILE 13 32.318 27.384 112.372 1.00 0.00

ATOM 3009 2HG2 ILE 13 33.897 28.150 111.871 1.00 0.00

ATOM 3010 3HG2 ILE 13 33.423 26.642 111.165 1.00 0.00

ATOM 3011 CG1 ILE 13 33.361 28.572 109.287 1.00 0.00

ATOM 3012 1HG1 ILE 13 34.295 29.007 109.642 1.00 0.00

ATOM 3013 2HG1 ILE 13 33.719 27.610 108.920 1.00 0.00

ATOM 3014 CD ILE 13 32.943 29.406 108.080 1.00 0.00

ATOM 3015 HD1 ILE 13 33.826 29.483 107.445 1.00 0.00

ATOM 3016 HD2 ILE 13 32.715 30.402 108.459 1.00 0.00

ATOM 3017 HD3 ILE 13 32.101 29.011 107.512 1.00 0.00

ATOM 3018 C ILE 13 31.151 26.267 109.680 1.00 0.00

ATOM 3019 O ILE 13 31.464 25.940 108.539 1.00 0.00

ATOM 3020 N GLY 14 30.735 25.368 110.574 1.00 0.00

ATOM 3021 H GLY 14 30.614 25.713 111.515 1.00 0.00

ATOM 3022 CA GLY 14 30.795 23.951 110.273 1.00 0.00

ATOM 3023 HA1 GLY 14 31.827 23.701 110.024 1.00 0.00

ATOM 3024 HA2 GLY 14 30.437 23.370 111.123 1.00 0.00

ATOM 3025 C GLY 14 30.101 23.525 108.986 1.00 0.00

ATOM 3026 O GLY 14 30.521 22.654 108.227 1.00 0.00

ATOM 3027 N ILE 15 28.927 24.092 108.708 1.00 0.00

ATOM 3028 H ILE 15 28.669 24.867 109.303 1.00 0.00

ATOM 3029 CA ILE 15 28.176 23.725 107.524 1.00 0.00

ATOM 3030 HA ILE 15 28.492 22.709 107.289 1.00 0.00

ATOM 3031 CB ILE 15 26.680 23.700 107.822 1.00 0.00

ATOM 3032 HB ILE 15 26.139 23.567 106.886 1.00 0.00

ATOM 3033 CG2 ILE 15 26.389 22.411 108.586 1.00 0.00

ATOM 3034 1HG2 ILE 15 26.327 21.544 107.928 1.00 0.00

ATOM 3035 2HG2 ILE 15 25.433 22.611 109.069 1.00 0.00

ATOM 3036 3HG2 ILE 15 27.129 22.261 109.372 1.00 0.00

ATOM 3037 CG1 ILE 15 26.226 25.025 108.429 1.00 0.00

ATOM 3038 1HG1 ILE 15 26.646 25.047 109.435 1.00 0.00

ATOM 3039 2HG1 ILE 15 26.512 25.820 107.740 1.00 0.00

ATOM 3040 CD ILE 15 24.710 25.203 108.427 1.00 0.00

ATOM 3041 HD1 ILE 15 24.309 25.412 107.435 1.00 0.00

ATOM 3042 HD2 ILE 15 24.446 26.074 109.027 1.00 0.00

ATOM 3043 HD3 ILE 15 24.285 24.305 108.875 1.00 0.00

ATOM 3044 C ILE 15 28.618 24.536 106.314 1.00 0.00

ATOM 3045 O ILE 15 28.410 24.088 105.189 1.00 0.00

ATOM 3046 N LEU 16 29.194 25.722 106.527 1.00 0.00

ATOM 3047 H LEU 16 29.431 25.971 107.477 1.00 0.00

ATOM 3048 CA LEU 16 29.508 26.657 105.466 1.00 0.00

ATOM 3049 HA LEU 16 28.776 26.448 104.686 1.00 0.00

ATOM 3050 CB LEU 16 29.241 28.073 105.973 1.00 0.00

ATOM 3051 HB1 LEU 16 29.618 28.950 105.449 1.00 0.00

ATOM 3052 HB2 LEU 16 29.837 28.150 106.881 1.00 0.00

ATOM 3053 CG LEU 16 27.785 28.409 106.282 1.00 0.00

ATOM 3054 HG LEU 16 27.371 27.610 106.897 1.00 0.00

ATOM 3055 CD1 LEU 16 27.718 29.730 107.045 1.00 0.00

ATOM 3056 1HD1 LEU 16 28.127 30.559 106.468 1.00 0.00

ATOM 3057 2HD1 LEU 16 28.262 29.721 107.989 1.00 0.00

ATOM 3058 3HD1 LEU 16 26.689 29.909 107.357 1.00 0.00

ATOM 3059 CD2 LEU 16 26.987 28.640 105.001 1.00 0.00

ATOM 3060 1HD2 LEU 16 27.027 27.752 104.371 1.00 0.00

ATOM 3061 2HD2 LEU 16 27.442 29.411 104.380 1.00 0.00

ATOM 3062 3HD2 LEU 16 25.927 28.852 105.136 1.00 0.00

ATOM 3063 C LEU 16 30.906 26.377 104.934 1.00 0.00

ATOM 3064 O LEU 16 31.322 27.138 104.064 1.00 0.00

ATOM 3065 N ALA 17 31.613 25.351 105.415 1.00 0.00

ATOM 3066 H ALA 17 31.187 24.753 106.108 1.00 0.00

ATOM 3067 CA ALA 17 32.848 24.896 104.809 1.00 0.00

ATOM 3068 HA ALA 17 33.028 25.404 103.861 1.00 0.00

ATOM 3069 CB ALA 17 33.904 25.464 105.752 1.00 0.00

ATOM 3070 HB1 ALA 17 33.657 26.519 105.873 1.00 0.00

ATOM 3071 HB2 ALA 17 34.928 25.396 105.385 1.00 0.00

ATOM 3072 HB3 ALA 17 33.918 24.873 106.667 1.00 0.00

ATOM 3073 C ALA 17 32.972 23.379 104.728 1.00 0.00

ATOM 3074 O ALA 17 33.895 22.864 104.103 1.00 0.00

ATOM 3075 N ALA 18 32.004 22.612 105.234 1.00 0.00

ATOM 3076 H ALA 18 31.276 23.074 105.759 1.00 0.00

ATOM 3077 CA ALA 18 31.851 21.194 104.972 1.00 0.00

ATOM 3078 HA ALA 18 32.814 20.799 105.293 1.00 0.00

ATOM 3079 CB ALA 18 30.762 20.718 105.928 1.00 0.00

ATOM 3080 HB1 ALA 18 29.830 21.216 105.659 1.00 0.00

ATOM 3081 HB2 ALA 18 30.687 19.667 105.651 1.00 0.00

ATOM 3082 HB3 ALA 18 31.021 20.706 106.987 1.00 0.00

ATOM 3083 C ALA 18 31.509 20.761 103.551 1.00 0.00

ATOM 3084 O ALA 18 32.024 19.766 103.048 1.00 0.00

ATOM 3085 N ILE 19 30.751 21.575 102.813 1.00 0.00

ATOM 3086 H ILE 19 30.428 22.452 103.193 1.00 0.00

ATOM 3087 CA ILE 19 30.236 21.280 101.491 1.00 0.00

ATOM 3088 HA ILE 19 30.858 20.460 101.138 1.00 0.00

ATOM 3089 CB ILE 19 28.813 20.748 101.636 1.00 0.00

ATOM 3090 HB ILE 19 28.529 20.549 100.603 1.00 0.00

ATOM 3091 CG2 ILE 19 28.667 19.519 102.529 1.00 0.00

ATOM 3092 1HG2 ILE 19 27.844 18.865 102.250 1.00 0.00

ATOM 3093 2HG2 ILE 19 28.407 19.897 103.518 1.00 0.00

ATOM 3094 3HG2 ILE 19 29.555 18.887 102.547 1.00 0.00

ATOM 3095 CG1 ILE 19 27.907 21.863 102.151 1.00 0.00

ATOM 3096 1HG1 ILE 19 28.246 22.118 103.155 1.00 0.00

ATOM 3097 2HG1 ILE 19 28.021 22.736 101.507 1.00 0.00

ATOM 3098 CD ILE 19 26.434 21.480 102.260 1.00 0.00

ATOM 3099 HD1 ILE 19 26.269 20.613 102.900 1.00 0.00

ATOM 3100 HD2 ILE 19 26.069 21.281 101.252 1.00 0.00

ATOM 3101 HD3 ILE 19 25.949 22.300 102.788 1.00 0.00

ATOM 3102 C ILE 19 30.496 22.278 100.370 1.00 0.00

ATOM 3103 O ILE 19 29.809 22.263 99.351 1.00 0.00

ATOM 3104 N ALA 20 31.496 23.152 100.507 1.00 0.00

ATOM 3105 H ALA 20 32.043 23.022 101.345 1.00 0.00

ATOM 3106 CA ALA 20 31.807 24.192 99.547 1.00 0.00

ATOM 3107 HA ALA 20 30.800 24.515 99.286 1.00 0.00

ATOM 3108 CB ALA 20 32.670 25.279 100.184 1.00 0.00

ATOM 3109 HB1 ALA 20 32.319 25.513 101.189 1.00 0.00

ATOM 3110 HB2 ALA 20 33.725 25.011 100.238 1.00 0.00

ATOM 3111 HB3 ALA 20 32.576 26.175 99.571 1.00 0.00

ATOM 3112 C ALA 20 32.283 23.620 98.219 1.00 0.00

ATOM 3113 O ALA 20 31.762 24.007 97.176 1.00 0.00

ATOM 3114 N ILE 21 33.260 22.713 98.252 1.00 0.00

ATOM 3115 H ILE 21 33.663 22.623 99.175 1.00 0.00

ATOM 3116 CA ILE 21 33.762 21.994 97.098 1.00 0.00

ATOM 3117 HA ILE 21 34.046 22.807 96.430 1.00 0.00

ATOM 3118 CB ILE 21 35.069 21.274 97.415 1.00 0.00

ATOM 3119 HB ILE 21 34.792 20.481 98.110 1.00 0.00

ATOM 3120 CG2 ILE 21 35.633 20.666 96.134 1.00 0.00

ATOM 3121 1HG2 ILE 21 34.966 19.883 95.773 1.00 0.00

ATOM 3122 2HG2 ILE 21 36.618 20.262 96.367 1.00 0.00

ATOM 3123 3HG2 ILE 21 35.669 21.365 95.298 1.00 0.00

ATOM 3124 CG1 ILE 21 36.062 22.226 98.079 1.00 0.00

ATOM 3125 1HG1 ILE 21 35.651 22.724 98.950 1.00 0.00

ATOM 3126 2HG1 ILE 21 36.874 21.580 98.409 1.00 0.00

ATOM 3127 CD ILE 21 36.661 23.328 97.211 1.00 0.00

ATOM 3128 HD1 ILE 21 37.385 23.890 97.802 1.00 0.00

ATOM 3129 HD2 ILE 21 35.917 24.052 96.884 1.00 0.00

ATOM 3130 HD3 ILE 21 37.144 22.957 96.307 1.00 0.00

ATOM 3131 C ILE 21 32.791 21.179 96.257 1.00 0.00

ATOM 3132 O ILE 21 32.941 21.420 95.061 1.00 0.00

ATOM 3133 N PRO 22 31.796 20.418 96.719 1.00 0.00

ATOM 3134 CD PRO 22 31.647 19.892 98.061 1.00 0.00

ATOM 3135 HD1 PRO 22 31.657 20.661 98.834 1.00 0.00

ATOM 3136 HD2 PRO 22 32.404 19.125 98.224 1.00 0.00

ATOM 3137 CG PRO 22 30.276 19.221 98.051 1.00 0.00

ATOM 3138 HG1 PRO 22 29.483 19.871 98.413 1.00 0.00

ATOM 3139 HG2 PRO 22 30.267 18.312 98.652 1.00 0.00

ATOM 3140 CB PRO 22 29.960 18.888 96.596 1.00 0.00

ATOM 3141 HB1 PRO 22 28.883 19.019 96.489 1.00 0.00

ATOM 3142 HB2 PRO 22 30.316 17.880 96.381 1.00 0.00

ATOM 3143 CA PRO 22 30.741 19.959 95.839 1.00 0.00

ATOM 3144 HA PRO 22 31.141 19.493 94.938 1.00 0.00

ATOM 3145 C PRO 22 29.802 21.038 95.321 1.00 0.00

ATOM 3146 O PRO 22 29.270 20.857 94.229 1.00 0.00

ATOM 3147 N GLN 23 29.680 22.141 96.064 1.00 0.00

ATOM 3148 H GLN 23 30.051 22.190 97.002 1.00 0.00

ATOM 3149 CA GLN 23 28.937 23.282 95.569 1.00 0.00

ATOM 3150 HA GLN 23 28.038 22.822 95.161 1.00 0.00

ATOM 3151 CB GLN 23 28.489 24.272 96.641 1.00 0.00

ATOM 3152 HB1 GLN 23 27.890 25.005 96.101 1.00 0.00

ATOM 3153 HB2 GLN 23 29.290 24.590 97.310 1.00 0.00

ATOM 3154 CG GLN 23 27.447 23.676 97.582 1.00 0.00

ATOM 3155 HG1 GLN 23 26.494 23.426 97.116 1.00 0.00

ATOM 3156 HG2 GLN 23 27.873 22.770 97.990 1.00 0.00

ATOM 3157 CD GLN 23 27.149 24.538 98.803 1.00 0.00

ATOM 3158 OE1 GLN 23 27.787 25.494 99.237 1.00 0.00

ATOM 3159 NE2 GLN 23 26.025 24.270 99.466 1.00 0.00

ATOM 3160 1HE2 GLN 23 25.525 23.418 99.260 1.00 0.00

ATOM 3161 2HE2 GLN 23 25.690 24.899 100.185 1.00 0.00

ATOM 3162 C GLN 23 29.608 23.980 94.395 1.00 0.00

ATOM 3163 O GLN 23 28.944 24.121 93.370 1.00 0.00

ATOM 3164 N PHE 24 30.910 24.230 94.555 1.00 0.00

ATOM 3165 H PHE 24 31.260 24.006 95.475 1.00 0.00

ATOM 3166 CA PHE 24 31.798 24.765 93.541 1.00 0.00

ATOM 3167 HA PHE 24 31.445 25.702 93.111 1.00 0.00

ATOM 3168 CB PHE 24 33.185 25.027 94.122 1.00 0.00

ATOM 3169 HB1 PHE 24 33.467 24.174 94.739 1.00 0.00

ATOM 3170 HB2 PHE 24 33.089 25.929 94.725 1.00 0.00

ATOM 3171 CG PHE 24 34.348 25.269 93.191 1.00 0.00

ATOM 3172 CD1 PHE 24 34.286 26.135 92.092 1.00 0.00

ATOM 3173 HD1 PHE 24 33.411 26.741 91.910 1.00 0.00

ATOM 3174 CE1 PHE 24 35.425 26.431 91.333 1.00 0.00

ATOM 3175 HE1 PHE 24 35.367 27.077 90.470 1.00 0.00

ATOM 3176 CZ PHE 24 36.631 25.818 91.695 1.00 0.00

ATOM 3177 HZ PHE 24 37.529 26.089 91.160 1.00 0.00

ATOM 3178 CE2 PHE 24 36.712 24.950 92.791 1.00 0.00

ATOM 3179 HE2 PHE 24 37.652 24.538 93.126 1.00 0.00

ATOM 3180 CD2 PHE 24 35.560 24.655 93.530 1.00 0.00

ATOM 3181 HD2 PHE 24 35.609 24.041 94.417 1.00 0.00

ATOM 3182 C PHE 24 31.755 23.810 92.358 1.00 0.00

ATOM 3183 O PHE 24 31.673 24.240 91.210 1.00 0.00

ATOM 3184 N SER 25 31.933 22.517 92.635 1.00 0.00

ATOM 3185 H SER 25 31.925 22.309 93.623 1.00 0.00

ATOM 3186 CA SER 25 31.935 21.520 91.582 1.00 0.00

ATOM 3187 HA SER 25 32.731 21.683 90.855 1.00 0.00

ATOM 3188 CB SER 25 32.255 20.124 92.106 1.00 0.00

ATOM 3189 HB1 SER 25 32.386 19.439 91.268 1.00 0.00

ATOM 3190 HB2 SER 25 31.422 19.849 92.754 1.00 0.00

ATOM 3191 OG SER 25 33.500 20.202 92.764 1.00 0.00

ATOM 3192 HG SER 25 33.450 20.734 93.562 1.00 0.00

ATOM 3193 C SER 25 30.660 21.410 90.759 1.00 0.00

ATOM 3194 O SER 25 30.808 21.200 89.558 1.00 0.00

ATOM 3195 N ALA 26 29.491 21.483 91.400 1.00 0.00

ATOM 3196 H ALA 26 29.470 21.507 92.410 1.00 0.00

ATOM 3197 CA ALA 26 28.243 21.667 90.688 1.00 0.00

ATOM 3198 HA ALA 26 28.119 20.747 90.116 1.00 0.00

ATOM 3199 CB ALA 26 27.043 21.823 91.617 1.00 0.00

ATOM 3200 HB1 ALA 26 26.193 21.434 91.060 1.00 0.00

ATOM 3201 HB2 ALA 26 27.132 21.199 92.507 1.00 0.00

ATOM 3202 HB3 ALA 26 26.956 22.873 91.894 1.00 0.00

ATOM 3203 C ALA 26 28.282 22.830 89.706 1.00 0.00

ATOM 3204 O ALA 26 27.879 22.643 88.561 1.00 0.00

ATOM 3205 N ALA 27 28.741 24.014 90.119 1.00 0.00

ATOM 3206 H ALA 27 29.002 24.118 91.089 1.00 0.00

ATOM 3207 CA ALA 27 28.880 25.144 89.222 1.00 0.00

ATOM 3208 HA ALA 27 27.866 25.306 88.861 1.00 0.00

ATOM 3209 CB ALA 27 29.169 26.407 90.029 1.00 0.00

ATOM 3210 HB1 ALA 27 28.349 26.402 90.746 1.00 0.00

ATOM 3211 HB2 ALA 27 29.081 27.399 89.585 1.00 0.00

ATOM 3212 HB3 ALA 27 30.139 26.296 90.514 1.00 0.00

ATOM 3213 C ALA 27 29.721 24.984 87.963 1.00 0.00

ATOM 3214 O ALA 27 29.346 25.486 86.905 1.00 0.00

ATOM 3215 N ARG 28 30.779 24.169 87.935 1.00 0.00

ATOM 3216 H ARG 28 31.083 23.617 88.724 1.00 0.00

ATOM 3217 CA ARG 28 31.608 23.793 86.808 1.00 0.00

ATOM 3218 HA ARG 28 31.767 24.708 86.238 1.00 0.00

ATOM 3219 CB ARG 28 32.923 23.226 87.334 1.00 0.00

ATOM 3220 HB1 ARG 28 33.622 22.858 86.583 1.00 0.00

ATOM 3221 HB2 ARG 28 32.550 22.405 87.947 1.00 0.00

ATOM 3222 CG ARG 28 33.710 24.233 88.168 1.00 0.00

ATOM 3223 HG1 ARG 28 33.709 25.148 87.576 1.00 0.00

ATOM 3224 HG2 ARG 28 33.176 24.451 89.093 1.00 0.00

ATOM 3225 CD ARG 28 35.122 23.795 88.545 1.00 0.00

ATOM 3226 HD1 ARG 28 35.628 23.557 87.609 1.00 0.00

ATOM 3227 HD2 ARG 28 35.538 24.633 89.104 1.00 0.00

ATOM 3228 NE ARG 28 35.040 22.579 89.355 1.00 0.00

ATOM 3229 HE ARG 28 34.155 22.092 89.359 1.00 0.00

ATOM 3230 CZ ARG 28 36.000 22.242 90.229 1.00 0.00

ATOM 3231 NH1 ARG 28 37.150 22.914 90.334 1.00 0.00

ATOM 3232 1HH1 ARG 28 37.321 23.763 89.810 1.00 0.00

ATOM 3233 2HH1 ARG 28 37.817 22.604 91.027 1.00 0.00

ATOM 3234 NH2 ARG 28 35.885 21.248 91.117 1.00 0.00

ATOM 3235 1HH2 ARG 28 35.004 20.758 91.126 1.00 0.00

ATOM 3236 2HH2 ARG 28 36.710 21.104 91.686 1.00 0.00

ATOM 3237 C ARG 28 30.894 22.753 85.955 1.00 0.00

ATOM 3238 O ARG 28 31.001 22.782 84.732 1.00 0.00

ATOM 3239 N VAL 29 30.141 21.799 86.508 1.00 0.00

ATOM 3240 H VAL 29 30.045 21.689 87.507 1.00 0.00

ATOM 3241 CA VAL 29 29.443 20.797 85.728 1.00 0.00

ATOM 3242 HA VAL 29 30.102 20.509 84.908 1.00 0.00

ATOM 3243 CB VAL 29 29.187 19.613 86.657 1.00 0.00

ATOM 3244 HB VAL 29 28.968 20.007 87.648 1.00 0.00

ATOM 3245 CG1 VAL 29 28.015 18.675 86.380 1.00 0.00

ATOM 3246 1HG1 VAL 29 28.155 18.227 85.396 1.00 0.00

ATOM 3247 2HG1 VAL 29 27.092 19.256 86.377 1.00 0.00

ATOM 3248 3HG1 VAL 29 27.949 17.845 87.082 1.00 0.00

ATOM 3249 CG2 VAL 29 30.429 18.746 86.836 1.00 0.00

ATOM 3250 1HG2 VAL 29 30.459 18.201 85.893 1.00 0.00

ATOM 3251 2HG2 VAL 29 30.200 17.983 87.579 1.00 0.00

ATOM 3252 3HG2 VAL 29 31.362 19.284 87.007 1.00 0.00

ATOM 3253 C VAL 29 28.199 21.284 85.001 1.00 0.00

ATOM 3254 O VAL 29 28.125 21.015 83.804 1.00 0.00

ATOM 3255 N LYS 30 27.408 22.162 85.623 1.00 0.00

ATOM 3256 H LYS 30 27.517 22.380 86.604 1.00 0.00

ATOM 3257 CA LYS 30 26.402 22.884 84.871 1.00 0.00

ATOM 3258 HA LYS 30 25.910 22.141 84.243 1.00 0.00

ATOM 3259 CB LYS 30 25.479 23.615 85.841 1.00 0.00

ATOM 3260 HB1 LYS 30 24.813 24.255 85.261 1.00 0.00

ATOM 3261 HB2 LYS 30 26.135 24.165 86.515 1.00 0.00

ATOM 3262 CG LYS 30 24.702 22.596 86.669 1.00 0.00

ATOM 3263 HG1 LYS 30 24.406 21.809 85.975 1.00 0.00

ATOM 3264 HG2 LYS 30 25.304 22.092 87.426 1.00 0.00

ATOM 3265 CD LYS 30 23.522 23.311 87.320 1.00 0.00

ATOM 3266 HD1 LYS 30 23.884 24.291 87.633 1.00 0.00

ATOM 3267 HD2 LYS 30 22.711 23.319 86.593 1.00 0.00

ATOM 3268 CE LYS 30 22.896 22.491 88.444 1.00 0.00

ATOM 3269 HE1 LYS 30 22.466 21.548 88.110 1.00 0.00

ATOM 3270 HE2 LYS 30 23.729 22.432 89.144 1.00 0.00

ATOM 3271 NZ LYS 30 21.739 23.164 89.051 1.00 0.00

ATOM 3272 HZ1 LYS 30 20.974 23.157 88.391 1.00 0.00

ATOM 3273 HZ2 LYS 30 21.846 24.147 89.259 1.00 0.00

ATOM 3274 HZ3 LYS 30 21.393 22.664 89.858 1.00 0.00

ATOM 3275 C LYS 30 27.004 23.850 83.861 1.00 0.00

ATOM 3276 O LYS 30 26.274 24.147 82.918 1.00 0.00

ATOM 3277 N ALA 31 28.224 24.350 84.077 1.00 0.00

ATOM 3278 H ALA 31 28.713 24.295 84.958 1.00 0.00

ATOM 3279 CA ALA 31 28.867 25.237 83.129 1.00 0.00

ATOM 3280 HA ALA 31 28.113 25.924 82.747 1.00 0.00

ATOM 3281 CB ALA 31 29.931 26.095 83.808 1.00 0.00

ATOM 3282 HB1 ALA 31 29.367 26.778 84.444 1.00 0.00

ATOM 3283 HB2 ALA 31 30.639 25.474 84.357 1.00 0.00

ATOM 3284 HB3 ALA 31 30.463 26.690 83.066 1.00 0.00

ATOM 3285 C ALA 31 29.291 24.500 81.866 1.00 0.00

ATOM 3286 O ALA 31 28.904 24.902 80.771 1.00 0.00

ATOM 3287 N TYR 32 30.003 23.387 82.058 1.00 0.00

ATOM 3288 H TYR 32 30.095 23.161 83.038 1.00 0.00

ATOM 3289 CA TYR 32 30.261 22.361 81.067 1.00 0.00

ATOM 3290 HA TYR 32 31.007 22.711 80.354 1.00 0.00

ATOM 3291 CB TYR 32 30.953 21.189 81.758 1.00 0.00

ATOM 3292 HB1 TYR 32 30.537 21.007 82.749 1.00 0.00

ATOM 3293 HB2 TYR 32 31.980 21.523 81.898 1.00 0.00

ATOM 3294 CG TYR 32 31.134 19.938 80.933 1.00 0.00

ATOM 3295 CD1 TYR 32 30.292 18.831 81.092 1.00 0.00

ATOM 3296 HD1 TYR 32 29.645 18.836 81.957 1.00 0.00

ATOM 3297 CE1 TYR 32 30.199 17.823 80.125 1.00 0.00

ATOM 3298 HE1 TYR 32 29.487 17.026 80.277 1.00 0.00

ATOM 3299 CZ TYR 32 31.036 17.840 78.992 1.00 0.00

ATOM 3300 OH TYR 32 30.990 16.831 78.074 1.00 0.00

ATOM 3301 HH TYR 32 30.376 16.101 78.176 1.00 0.00

ATOM 3302 CE2 TYR 32 31.940 18.913 78.869 1.00 0.00

ATOM 3303 HE2 TYR 32 32.446 18.931 77.922 1.00 0.00

ATOM 3304 CD2 TYR 32 32.012 19.918 79.843 1.00 0.00

ATOM 3305 HD2 TYR 32 32.668 20.760 79.677 1.00 0.00

ATOM 3306 C TYR 32 29.061 21.932 80.234 1.00 0.00

ATOM 3307 O TYR 32 29.129 21.937 79.007 1.00 0.00

ATOM 3308 N ASN 33 27.929 21.759 80.921 1.00 0.00

ATOM 3309 H ASN 33 27.947 21.830 81.928 1.00 0.00

ATOM 3310 CA ASN 33 26.726 21.294 80.261 1.00 0.00

ATOM 3311 HA ASN 33 27.064 20.451 79.664 1.00 0.00

ATOM 3312 CB ASN 33 25.718 20.638 81.200 1.00 0.00

ATOM 3313 HB1 ASN 33 26.155 19.746 81.650 1.00 0.00

ATOM 3314 HB2 ASN 33 25.510 21.373 81.975 1.00 0.00

ATOM 3315 CG ASN 33 24.410 20.349 80.475 1.00 0.00

ATOM 3316 OD1 ASN 33 24.425 19.424 79.667 1.00 0.00

ATOM 3317 ND2 ASN 33 23.353 21.159 80.538 1.00 0.00

ATOM 3318 1HD2 ASN 33 23.276 21.796 81.319 1.00 0.00

ATOM 3319 2HD2 ASN 33 22.623 21.024 79.850 1.00 0.00

ATOM 3320 C ASN 33 26.158 22.383 79.361 1.00 0.00

ATOM 3321 O ASN 33 25.791 22.239 78.196 1.00 0.00

ATOM 3322 N SER 34 26.192 23.627 79.840 1.00 0.00

ATOM 3323 H SER 34 26.473 23.754 80.801 1.00 0.00

ATOM 3324 CA SER 34 25.842 24.794 79.055 1.00 0.00

ATOM 3325 HA SER 34 24.818 24.707 78.691 1.00 0.00

ATOM 3326 CB SER 34 25.944 26.052 79.915 1.00 0.00

ATOM 3327 HB1 SER 34 25.410 26.870 79.432 1.00 0.00

ATOM 3328 HB2 SER 34 26.937 26.478 80.052 1.00 0.00

ATOM 3329 OG SER 34 25.332 25.965 81.183 1.00 0.00

ATOM 3330 HG SER 34 25.749 25.274 81.703 1.00 0.00

ATOM 3331 C SER 34 26.740 24.974 77.840 1.00 0.00

ATOM 3332 O SER 34 26.220 25.292 76.772 1.00 0.00

ATOM 3333 N ALA 35 28.065 24.836 77.926 1.00 0.00

ATOM 3334 H ALA 35 28.430 24.601 78.838 1.00 0.00

ATOM 3335 CA ALA 35 28.979 24.846 76.802 1.00 0.00

ATOM 3336 HA ALA 35 28.844 25.675 76.107 1.00 0.00

ATOM 3337 CB ALA 35 30.400 24.784 77.355 1.00 0.00

ATOM 3338 HB1 ALA 35 31.109 24.831 76.529 1.00 0.00

ATOM 3339 HB2 ALA 35 30.487 25.737 77.875 1.00 0.00

ATOM 3340 HB3 ALA 35 30.507 23.989 78.093 1.00 0.00

ATOM 3341 C ALA 35 28.642 23.701 75.858 1.00 0.00

ATOM 3342 O ALA 35 28.517 23.928 74.656 1.00 0.00

ATOM 3343 N ALA 36 28.501 22.467 76.347 1.00 0.00

ATOM 3344 H ALA 36 28.437 22.292 77.340 1.00 0.00

ATOM 3345 CA ALA 36 28.489 21.323 75.457 1.00 0.00

ATOM 3346 HA ALA 36 29.395 21.421 74.861 1.00 0.00

ATOM 3347 CB ALA 36 28.623 20.035 76.263 1.00 0.00

ATOM 3348 HB1 ALA 36 29.572 20.106 76.795 1.00 0.00

ATOM 3349 HB2 ALA 36 28.769 19.176 75.608 1.00 0.00

ATOM 3350 HB3 ALA 36 27.805 19.900 76.971 1.00 0.00

ATOM 3351 C ALA 36 27.314 21.313 74.488 1.00 0.00

ATOM 3352 O ALA 36 27.401 21.173 73.270 1.00 0.00

ATOM 3353 N SER 37 26.145 21.487 75.108 1.00 0.00

ATOM 3354 H SER 37 26.094 21.655 76.103 1.00 0.00

ATOM 3355 CA SER 37 24.856 21.668 74.471 1.00 0.00

ATOM 3356 HA SER 37 24.588 20.791 73.886 1.00 0.00

ATOM 3357 CB SER 37 23.755 21.864 75.510 1.00 0.00

ATOM 3358 HB1 SER 37 22.743 21.964 75.130 1.00 0.00

ATOM 3359 HB2 SER 37 23.782 21.114 76.299 1.00 0.00

ATOM 3360 OG SER 37 24.056 22.996 76.296 1.00 0.00

ATOM 3361 HG SER 37 24.577 22.804 77.080 1.00 0.00

ATOM 3362 C SER 37 24.811 22.811 73.467 1.00 0.00

ATOM 3363 O SER 37 24.291 22.628 72.369 1.00 0.00

ATOM 3364 N SER 38 25.342 23.992 73.795 1.00 0.00

ATOM 3365 H SER 38 25.667 24.204 74.727 1.00 0.00

ATOM 3366 CA SER 38 25.285 25.097 72.860 1.00 0.00

ATOM 3367 HA SER 38 24.349 25.134 72.303 1.00 0.00

ATOM 3368 CB SER 38 25.530 26.353 73.691 1.00 0.00

ATOM 3369 HB1 SER 38 24.742 26.460 74.436 1.00 0.00

ATOM 3370 HB2 SER 38 25.479 27.203 73.010 1.00 0.00

ATOM 3371 OG SER 38 26.709 26.221 74.451 1.00 0.00

ATOM 3372 HG SER 38 26.407 25.994 75.335 1.00 0.00

ATOM 3373 C SER 38 26.310 25.048 71.736 1.00 0.00

ATOM 3374 O SER 38 26.000 25.408 70.603 1.00 0.00

ATOM 3375 N ASP 39 27.496 24.447 71.858 1.00 0.00

ATOM 3376 H ASP 39 27.858 24.184 72.763 1.00 0.00

ATOM 3377 CA ASP 39 28.346 24.116 70.731 1.00 0.00

ATOM 3378 HA ASP 39 28.545 25.042 70.191 1.00 0.00

ATOM 3379 CB ASP 39 29.724 23.775 71.290 1.00 0.00

ATOM 3380 HB1 ASP 39 30.213 24.675 71.663 1.00 0.00

ATOM 3381 HB2 ASP 39 29.721 23.187 72.207 1.00 0.00

ATOM 3382 CG ASP 39 30.588 23.084 70.243 1.00 0.00

ATOM 3383 OD1 ASP 39 31.243 23.767 69.428 1.00 0.00

ATOM 3384 OD2 ASP 39 30.600 21.839 70.133 1.00 0.00

ATOM 3385 C ASP 39 27.811 23.014 69.828 1.00 0.00

ATOM 3386 O ASP 39 27.825 23.148 68.606 1.00 0.00

ATOM 3387 N LEU 40 27.096 22.035 70.385 1.00 0.00

ATOM 3388 H LEU 40 27.116 21.904 71.387 1.00 0.00

ATOM 3389 CA LEU 40 26.478 20.942 69.662 1.00 0.00

ATOM 3390 HA LEU 40 27.218 20.482 69.008 1.00 0.00

ATOM 3391 CB LEU 40 26.107 19.883 70.696 1.00 0.00

ATOM 3392 HB1 LEU 40 25.574 20.320 71.541 1.00 0.00

ATOM 3393 HB2 LEU 40 27.045 19.472 71.070 1.00 0.00

ATOM 3394 CG LEU 40 25.275 18.741 70.119 1.00 0.00

ATOM 3395 HG LEU 40 24.325 19.128 69.754 1.00 0.00

ATOM 3396 CD1 LEU 40 26.006 18.028 68.984 1.00 0.00

ATOM 3397 1HD1 LEU 40 27.039 17.814 69.243 1.00 0.00

ATOM 3398 2HD1 LEU 40 26.013 18.677 68.108 1.00 0.00

ATOM 3399 3HD1 LEU 40 25.616 17.094 68.704 1.00 0.00

ATOM 3400 CD2 LEU 40 24.831 17.872 71.291 1.00 0.00

ATOM 3401 1HD2 LEU 40 24.236 17.024 70.954 1.00 0.00

ATOM 3402 2HD2 LEU 40 24.217 18.359 71.961 1.00 0.00

ATOM 3403 3HD2 LEU 40 25.737 17.392 71.658 1.00 0.00

ATOM 3404 C LEU 40 25.276 21.433 68.867 1.00 0.00

ATOM 3405 O LEU 40 25.043 20.911 67.780 1.00 0.00

ATOM 3406 N ARG 41 24.482 22.425 69.279 1.00 0.00

ATOM 3407 H ARG 41 24.643 22.749 70.221 1.00 0.00

ATOM 3408 CA ARG 41 23.458 23.017 68.441 1.00 0.00

ATOM 3409 HA ARG 41 22.801 22.246 68.047 1.00 0.00

ATOM 3410 CB ARG 41 22.712 24.085 69.233 1.00 0.00

ATOM 3411 HB1 ARG 41 23.450 24.860 69.443 1.00 0.00

ATOM 3412 HB2 ARG 41 22.381 23.593 70.147 1.00 0.00

ATOM 3413 CG ARG 41 21.489 24.658 68.521 1.00 0.00

ATOM 3414 HG1 ARG 41 21.038 25.388 69.192 1.00 0.00

ATOM 3415 HG2 ARG 41 21.873 25.170 67.640 1.00 0.00

ATOM 3416 CD ARG 41 20.347 23.722 68.136 1.00 0.00

ATOM 3417 HD1 ARG 41 20.826 22.927 67.566 1.00 0.00

ATOM 3418 HD2 ARG 41 19.912 23.300 69.043 1.00 0.00

ATOM 3419 NE ARG 41 19.322 24.296 67.264 1.00 0.00

ATOM 3420 HE ARG 41 19.282 25.303 67.196 1.00 0.00

ATOM 3421 CZ ARG 41 18.442 23.524 66.612 1.00 0.00

ATOM 3422 NH1 ARG 41 18.462 22.189 66.540 1.00 0.00

ATOM 3423 1HH1 ARG 41 19.257 21.622 66.807 1.00 0.00

ATOM 3424 2HH1 ARG 41 17.833 21.732 65.897 1.00 0.00

ATOM 3425 NH2 ARG 41 17.509 24.156 65.889 1.00 0.00

ATOM 3426 1HH2 ARG 41 17.501 25.162 65.973 1.00 0.00

ATOM 3427 2HH2 ARG 41 16.787 23.560 65.507 1.00 0.00

ATOM 3428 C ARG 41 24.021 23.679 67.191 1.00 0.00

ATOM 3429 O ARG 41 23.272 23.772 66.222 1.00 0.00

ATOM 3430 N ASN 42 25.230 24.244 67.162 1.00 0.00

ATOM 3431 H ASN 42 25.821 24.120 67.971 1.00 0.00

ATOM 3432 CA ASN 42 25.903 24.680 65.956 1.00 0.00

ATOM 3433 HA ASN 42 25.117 25.278 65.496 1.00 0.00

ATOM 3434 CB ASN 42 27.032 25.594 66.426 1.00 0.00

ATOM 3435 HB1 ASN 42 26.569 26.476 66.868 1.00 0.00

ATOM 3436 HB2 ASN 42 27.610 25.225 67.271 1.00 0.00

ATOM 3437 CG ASN 42 27.913 26.002 65.254 1.00 0.00

ATOM 3438 OD1 ASN 42 27.539 26.835 64.431 1.00 0.00

ATOM 3439 ND2 ASN 42 29.124 25.467 65.082 1.00 0.00

ATOM 3440 1HD2 ASN 42 29.603 24.997 65.837 1.00 0.00

ATOM 3441 2HD2 ASN 42 29.687 25.691 64.275 1.00 0.00

ATOM 3442 C ASN 42 26.355 23.526 65.070 1.00 0.00

ATOM 3443 O ASN 42 26.448 23.696 63.857 1.00 0.00

ATOM 3444 N LEU 43 26.714 22.382 65.657 1.00 0.00

ATOM 3445 H LEU 43 26.566 22.387 66.655 1.00 0.00

ATOM 3446 CA LEU 43 27.292 21.198 65.053 1.00 0.00

ATOM 3447 HA LEU 43 27.992 21.605 64.325 1.00 0.00

ATOM 3448 CB LEU 43 27.925 20.289 66.105 1.00 0.00

ATOM 3449 HB1 LEU 43 27.086 19.807 66.607 1.00 0.00

ATOM 3450 HB2 LEU 43 28.449 20.958 66.787 1.00 0.00

ATOM 3451 CG LEU 43 28.829 19.129 65.697 1.00 0.00

ATOM 3452 HG LEU 43 28.236 18.484 65.049 1.00 0.00

ATOM 3453 CD1 LEU 43 29.986 19.633 64.839 1.00 0.00

ATOM 3454 1HD1 LEU 43 30.777 20.126 65.403 1.00 0.00

ATOM 3455 2HD1 LEU 43 29.585 20.237 64.025 1.00 0.00

ATOM 3456 3HD1 LEU 43 30.533 18.800 64.398 1.00 0.00

ATOM 3457 CD2 LEU 43 29.300 18.361 66.929 1.00 0.00

ATOM 3458 1HD2 LEU 43 29.955 17.517 66.711 1.00 0.00

ATOM 3459 2HD2 LEU 43 28.504 17.917 67.526 1.00 0.00

ATOM 3460 3HD2 LEU 43 29.912 18.967 67.596 1.00 0.00

ATOM 3461 C LEU 43 26.182 20.557 64.232 1.00 0.00

ATOM 3462 O LEU 43 26.425 20.132 63.105 1.00 0.00

ATOM 3463 N LYS 44 24.980 20.375 64.782 1.00 0.00

ATOM 3464 H LYS 44 24.811 20.862 65.650 1.00 0.00

ATOM 3465 CA LYS 44 23.841 19.725 64.165 1.00 0.00

ATOM 3466 HA LYS 44 23.996 19.392 63.138 1.00 0.00

ATOM 3467 CB LYS 44 23.629 18.408 64.906 1.00 0.00

ATOM 3468 HB1 LYS 44 23.368 18.474 65.962 1.00 0.00

ATOM 3469 HB2 LYS 44 24.587 17.888 64.880 1.00 0.00

ATOM 3470 CG LYS 44 22.618 17.535 64.168 1.00 0.00

ATOM 3471 HG1 LYS 44 21.640 18.005 64.280 1.00 0.00

ATOM 3472 HG2 LYS 44 23.018 17.325 63.176 1.00 0.00

ATOM 3473 CD LYS 44 22.713 16.139 64.778 1.00 0.00

ATOM 3474 HD1 LYS 44 23.737 15.777 64.672 1.00 0.00

ATOM 3475 HD2 LYS 44 22.444 16.176 65.833 1.00 0.00

ATOM 3476 CE LYS 44 21.695 15.210 64.127 1.00 0.00

ATOM 3477 HE1 LYS 44 21.899 15.039 63.070 1.00 0.00

ATOM 3478 HE2 LYS 44 21.921 14.239 64.566 1.00 0.00

ATOM 3479 NZ LYS 44 20.313 15.689 64.295 1.00 0.00

ATOM 3480 HZ1 LYS 44 20.252 16.693 64.202 1.00 0.00

ATOM 3481 HZ2 LYS 44 20.067 15.555 65.265 1.00 0.00

ATOM 3482 HZ3 LYS 44 19.671 15.243 63.655 1.00 0.00

ATOM 3483 C LYS 44 22.623 20.639 64.158 1.00 0.00

ATOM 3484 O LYS 44 21.882 20.823 65.119 1.00 0.00

ATOM 3485 N THR 45 22.404 21.320 63.030 1.00 0.00

ATOM 3486 H THR 45 23.088 21.248 62.291 1.00 0.00

ATOM 3487 CA THR 45 21.261 22.164 62.745 1.00 0.00

ATOM 3488 HA THR 45 20.429 21.654 63.231 1.00 0.00

ATOM 3489 CB THR 45 21.553 23.505 63.413 1.00 0.00

ATOM 3490 HB THR 45 21.589 23.447 64.501 1.00 0.00

ATOM 3491 CG2 THR 45 22.816 24.235 62.965 1.00 0.00

ATOM 3492 1HG2 THR 45 22.762 24.503 61.910 1.00 0.00

ATOM 3493 2HG2 THR 45 23.788 23.766 63.114 1.00 0.00

ATOM 3494 3HG2 THR 45 22.849 25.185 63.499 1.00 0.00

ATOM 3495 OG1 THR 45 20.603 24.488 63.070 1.00 0.00

ATOM 3496 HG1 THR 45 20.580 25.145 63.770 1.00 0.00

ATOM 3497 C THR 45 20.865 22.367 61.290 1.00 0.00

ATOM 3498 O THR 45 19.681 22.553 61.015 1.00 0.00

ATOM 3499 N ALA 46 21.819 22.363 60.356 1.00 0.00

ATOM 3500 H ALA 46 22.763 22.094 60.592 1.00 0.00

ATOM 3501 CA ALA 46 21.582 22.862 59.015 1.00 0.00

ATOM 3502 HA ALA 46 20.559 23.162 58.793 1.00 0.00

ATOM 3503 CB ALA 46 22.304 24.200 58.904 1.00 0.00

ATOM 3504 HB1 ALA 46 22.055 24.843 59.749 1.00 0.00

ATOM 3505 HB2 ALA 46 22.068 24.587 57.912 1.00 0.00

ATOM 3506 HB3 ALA 46 23.354 23.909 58.887 1.00 0.00

ATOM 3507 C ALA 46 21.889 21.991 57.804 1.00 0.00

ATOM 3508 O ALA 46 21.094 21.944 56.868 1.00 0.00

ATOM 3509 N LEU 47 22.963 21.202 57.870 1.00 0.00

ATOM 3510 H LEU 47 23.578 21.360 58.656 1.00 0.00

ATOM 3511 CA LEU 47 23.451 20.348 56.806 1.00 0.00

ATOM 3512 HA LEU 47 23.543 20.968 55.917 1.00 0.00

ATOM 3513 CB LEU 47 24.834 19.908 57.282 1.00 0.00

ATOM 3514 HB1 LEU 47 25.270 19.227 56.552 1.00 0.00

ATOM 3515 HB2 LEU 47 24.847 19.254 58.153 1.00 0.00

ATOM 3516 CG LEU 47 25.784 21.078 57.531 1.00 0.00

ATOM 3517 HG LEU 47 25.274 21.857 58.098 1.00 0.00

ATOM 3518 CD1 LEU 47 26.979 20.734 58.414 1.00 0.00

ATOM 3519 1HD1 LEU 47 26.545 20.087 59.176 1.00 0.00

ATOM 3520 2HD1 LEU 47 27.507 21.637 58.719 1.00 0.00

ATOM 3521 3HD1 LEU 47 27.717 20.139 57.875 1.00 0.00

ATOM 3522 CD2 LEU 47 26.424 21.501 56.213 1.00 0.00

ATOM 3523 1HD2 LEU 47 25.607 21.773 55.549 1.00 0.00

ATOM 3524 2HD2 LEU 47 26.950 20.690 55.708 1.00 0.00

ATOM 3525 3HD2 LEU 47 26.987 22.430 56.304 1.00 0.00

ATOM 3526 C LEU 47 22.618 19.164 56.333 1.00 0.00

ATOM 3527 O LEU 47 22.783 18.690 55.211 1.00 0.00

ATOM 3528 N GLU 48 21.686 18.655 57.140 1.00 0.00

ATOM 3529 H GLU 48 21.515 19.166 57.993 1.00 0.00

ATOM 3530 CA GLU 48 20.869 17.486 56.873 1.00 0.00

ATOM 3531 HA GLU 48 20.730 17.318 55.805 1.00 0.00

ATOM 3532 CB GLU 48 21.559 16.263 57.470 1.00 0.00

ATOM 3533 HB1 GLU 48 20.884 15.442 57.701 1.00 0.00

ATOM 3534 HB2 GLU 48 21.851 16.527 58.479 1.00 0.00

ATOM 3535 CG GLU 48 22.678 15.608 56.667 1.00 0.00

ATOM 3536 HG1 GLU 48 22.284 15.299 55.699 1.00 0.00

ATOM 3537 HG2 GLU 48 23.476 16.322 56.464 1.00 0.00

ATOM 3538 CD GLU 48 23.157 14.383 57.434 1.00 0.00

ATOM 3539 OE1 GLU 48 23.928 14.545 58.403 1.00 0.00

ATOM 3540 OE2 GLU 48 22.914 13.245 56.979 1.00 0.00

ATOM 3541 C GLU 48 19.430 17.498 57.370 1.00 0.00

ATOM 3542 O GLU 48 18.550 16.950 56.711 1.00 0.00

ATOM 3543 N SER 49 19.125 18.337 58.363 1.00 0.00

ATOM 3544 H SER 49 19.885 18.900 58.714 1.00 0.00

ATOM 3545 CA SER 49 17.808 18.791 58.764 1.00 0.00

ATOM 3546 HA SER 49 17.183 17.953 59.073 1.00 0.00

ATOM 3547 CB SER 49 17.959 19.792 59.905 1.00 0.00

ATOM 3548 HB1 SER 49 18.659 19.450 60.667 1.00 0.00

ATOM 3549 HB2 SER 49 16.971 20.080 60.264 1.00 0.00

ATOM 3550 OG SER 49 18.551 20.944 59.346 1.00 0.00

ATOM 3551 HG SER 49 18.816 21.532 60.057 1.00 0.00

ATOM 3552 C SER 49 17.006 19.390 57.618 1.00 0.00

ATOM 3553 O SER 49 15.788 19.251 57.602 1.00 0.00

ATOM 3554 N ALA 50 17.658 19.872 56.556 1.00 0.00

ATOM 3555 H ALA 50 18.662 19.938 56.461 1.00 0.00

ATOM 3556 CA ALA 50 16.998 20.342 55.355 1.00 0.00

ATOM 3557 HA ALA 50 16.250 21.116 55.508 1.00 0.00

ATOM 3558 CB ALA 50 18.068 20.890 54.415 1.00 0.00

ATOM 3559 HB1 ALA 50 18.766 20.073 54.229 1.00 0.00

ATOM 3560 HB2 ALA 50 17.623 21.232 53.481 1.00 0.00

ATOM 3561 HB3 ALA 50 18.637 21.681 54.903 1.00 0.00

ATOM 3562 C ALA 50 16.230 19.210 54.688 1.00 0.00

ATOM 3563 O ALA 50 15.082 19.450 54.321 1.00 0.00

ATOM 3564 N PHE 51 16.720 17.973 54.793 1.00 0.00

ATOM 3565 H PHE 51 17.617 17.833 55.235 1.00 0.00

ATOM 3566 CA PHE 51 16.111 16.840 54.125 1.00 0.00

ATOM 3567 HA PHE 51 15.480 17.274 53.349 1.00 0.00

ATOM 3568 CB PHE 51 17.170 16.179 53.248 1.00 0.00

ATOM 3569 HB1 PHE 51 16.764 15.481 52.516 1.00 0.00

ATOM 3570 HB2 PHE 51 17.778 15.635 53.971 1.00 0.00

ATOM 3571 CG PHE 51 18.089 17.187 52.599 1.00 0.00

ATOM 3572 CD1 PHE 51 19.470 17.122 52.821 1.00 0.00

ATOM 3573 HD1 PHE 51 19.830 16.297 53.416 1.00 0.00

ATOM 3574 CE1 PHE 51 20.339 18.176 52.514 1.00 0.00

ATOM 3575 HE1 PHE 51 21.400 18.113 52.707 1.00 0.00

ATOM 3576 CZ PHE 51 19.812 19.242 51.775 1.00 0.00

ATOM 3577 HZ PHE 51 20.475 20.043 51.481 1.00 0.00

ATOM 3578 CE2 PHE 51 18.459 19.293 51.419 1.00 0.00

ATOM 3579 HE2 PHE 51 18.108 20.134 50.840 1.00 0.00

ATOM 3580 CD2 PHE 51 17.612 18.260 51.836 1.00 0.00

ATOM 3581 HD2 PHE 51 16.557 18.356 51.625 1.00 0.00

ATOM 3582 C PHE 51 15.348 15.853 54.997 1.00 0.00

ATOM 3583 O PHE 51 15.258 14.695 54.597 1.00 0.00

ATOM 3584 N ALA 52 15.227 16.114 56.300 1.00 0.00

ATOM 3585 H ALA 52 15.444 17.035 56.655 1.00 0.00

ATOM 3586 CA ALA 52 15.018 15.046 57.257 1.00 0.00

ATOM 3587 HA ALA 52 15.664 14.213 56.979 1.00 0.00

ATOM 3588 CB ALA 52 15.368 15.612 58.630 1.00 0.00

ATOM 3589 HB1 ALA 52 16.428 15.866 58.640 1.00 0.00

ATOM 3590 HB2 ALA 52 14.621 16.326 58.981 1.00 0.00

ATOM 3591 HB3 ALA 52 15.246 14.764 59.302 1.00 0.00

ATOM 3592 C ALA 52 13.605 14.482 57.218 1.00 0.00

ATOM 3593 O ALA 52 13.400 13.310 57.525 1.00 0.00

ATOM 3594 N ASP 53 12.656 15.367 56.903 1.00 0.00

ATOM 3595 H ASP 53 12.885 16.350 56.903 1.00 0.00

ATOM 3596 CA ASP 53 11.268 14.990 56.731 1.00 0.00

ATOM 3597 HA ASP 53 10.980 14.481 57.651 1.00 0.00

ATOM 3598 CB ASP 53 10.549 16.302 56.432 1.00 0.00

ATOM 3599 HB1 ASP 53 10.709 17.002 57.252 1.00 0.00

ATOM 3600 HB2 ASP 53 10.927 16.682 55.483 1.00 0.00

ATOM 3601 CG ASP 53 9.057 16.022 56.318 1.00 0.00

ATOM 3602 OD1 ASP 53 8.267 16.156 57.277 1.00 0.00

ATOM 3603 OD2 ASP 53 8.585 15.783 55.187 1.00 0.00

ATOM 3604 C ASP 53 11.029 13.962 55.633 1.00 0.00

ATOM 3605 O ASP 53 10.363 12.993 55.983 1.00 0.00

ATOM 3606 N ASP 54 11.551 14.074 54.408 1.00 0.00

ATOM 3607 H ASP 54 12.059 14.925 54.217 1.00 0.00

ATOM 3608 CA ASP 54 11.477 13.057 53.378 1.00 0.00

ATOM 3609 HA ASP 54 10.432 12.786 53.225 1.00 0.00

ATOM 3610 CB ASP 54 11.958 13.618 52.043 1.00 0.00

ATOM 3611 HB1 ASP 54 11.531 14.559 51.697 1.00 0.00

ATOM 3612 HB2 ASP 54 13.013 13.886 52.110 1.00 0.00

ATOM 3613 CG ASP 54 11.714 12.576 50.959 1.00 0.00

ATOM 3614 OD1 ASP 54 12.657 11.842 50.595 1.00 0.00

ATOM 3615 OD2 ASP 54 10.692 12.633 50.243 1.00 0.00

ATOM 3616 C ASP 54 12.278 11.821 53.761 1.00 0.00

ATOM 3617 O ASP 54 11.870 10.694 53.492 1.00 0.00

ATOM 3618 N GLN 55 13.343 11.966 54.553 1.00 0.00

ATOM 3619 H GLN 55 13.737 12.897 54.547 1.00 0.00

ATOM 3620 CA GLN 55 14.052 10.825 55.098 1.00 0.00

ATOM 3621 HA GLN 55 14.171 10.088 54.305 1.00 0.00

ATOM 3622 CB GLN 55 15.496 11.187 55.428 1.00 0.00

ATOM 3623 HB1 GLN 55 16.015 10.355 55.905 1.00 0.00

ATOM 3624 HB2 GLN 55 15.439 12.052 56.088 1.00 0.00

ATOM 3625 CG GLN 55 16.263 11.411 54.128 1.00 0.00

ATOM 3626 HG1 GLN 55 16.409 10.407 53.737 1.00 0.00

ATOM 3627 HG2 GLN 55 15.810 12.108 53.422 1.00 0.00

ATOM 3628 CD GLN 55 17.652 11.970 54.410 1.00 0.00

ATOM 3629 OE1 GLN 55 18.110 12.330 55.491 1.00 0.00

ATOM 3630 NE2 GLN 55 18.541 12.063 53.423 1.00 0.00

ATOM 3631 1HE2 GLN 55 18.317 11.620 52.542 1.00 0.00

ATOM 3632 2HE2 GLN 55 19.429 12.496 53.639 1.00 0.00

ATOM 3633 C GLN 55 13.417 10.096 56.274 1.00 0.00

ATOM 3634 O GLN 55 13.947 9.063 56.678 1.00 0.00

ATOM 3635 N THR 56 12.304 10.629 56.782 1.00 0.00

ATOM 3636 H THR 56 11.905 11.346 56.195 1.00 0.00

ATOM 3637 CA THR 56 11.516 10.207 57.924 1.00 0.00

ATOM 3638 HA THR 56 10.596 10.786 58.014 1.00 0.00

ATOM 3639 CB THR 56 10.852 8.832 57.891 1.00 0.00

ATOM 3640 HB THR 56 10.219 8.620 58.753 1.00 0.00

ATOM 3641 CG2 THR 56 10.066 8.739 56.588 1.00 0.00

ATOM 3642 1HG2 THR 56 9.489 7.823 56.456 1.00 0.00

ATOM 3643 2HG2 THR 56 10.696 8.755 55.698 1.00 0.00

ATOM 3644 3HG2 THR 56 9.356 9.566 56.580 1.00 0.00

ATOM 3645 OG1 THR 56 11.806 7.796 57.840 1.00 0.00

ATOM 3646 HG1 THR 56 12.536 8.169 57.341 1.00 0.00

ATOM 3647 C THR 56 12.196 10.438 59.265 1.00 0.00

ATOM 3648 O THR 56 11.867 9.680 60.174 1.00 0.00

ATOM 3649 N TYR 57 13.156 11.353 59.411 1.00 0.00

ATOM 3650 H TYR 57 13.266 11.982 58.628 1.00 0.00

ATOM 3651 CA TYR 57 13.737 11.726 60.684 1.00 0.00

ATOM 3652 HA TYR 57 13.521 10.938 61.405 1.00 0.00

ATOM 3653 CB TYR 57 15.228 11.879 60.395 1.00 0.00

ATOM 3654 HB1 TYR 57 15.604 12.123 61.350 1.00 0.00

ATOM 3655 HB2 TYR 57 15.439 12.642 59.646 1.00 0.00

ATOM 3656 CG TYR 57 15.955 10.634 59.946 1.00 0.00

ATOM 3657 CD1 TYR 57 16.485 10.506 58.657 1.00 0.00

ATOM 3658 HD1 TYR 57 16.425 11.349 57.985 1.00 0.00

ATOM 3659 CE1 TYR 57 17.132 9.326 58.269 1.00 0.00

ATOM 3660 HE1 TYR 57 17.609 9.186 57.310 1.00 0.00

ATOM 3661 CZ TYR 57 17.168 8.200 59.115 1.00 0.00

ATOM 3662 OH TYR 57 17.866 7.101 58.708 1.00 0.00

ATOM 3663 HH TYR 57 17.967 6.436 59.392 1.00 0.00

ATOM 3664 CE2 TYR 57 16.606 8.357 60.398 1.00 0.00

ATOM 3665 HE2 TYR 57 16.746 7.618 61.172 1.00 0.00

ATOM 3666 CD2 TYR 57 16.004 9.546 60.826 1.00 0.00

ATOM 3667 HD2 TYR 57 15.689 9.576 61.859 1.00 0.00

ATOM 3668 C TYR 57 13.145 13.068 61.092 1.00 0.00

ATOM 3669 O TYR 57 12.671 13.805 60.232 1.00 0.00

ATOM 3670 N PRO 58 13.084 13.406 62.382 1.00 0.00

ATOM 3671 CD PRO 58 13.015 12.413 63.436 1.00 0.00

ATOM 3672 HD1 PRO 58 13.999 12.224 63.864 1.00 0.00

ATOM 3673 HD2 PRO 58 12.521 11.457 63.260 1.00 0.00

ATOM 3674 CG PRO 58 12.214 13.088 64.544 1.00 0.00

ATOM 3675 HG1 PRO 58 12.304 12.623 65.523 1.00 0.00

ATOM 3676 HG2 PRO 58 11.146 13.102 64.326 1.00 0.00

ATOM 3677 CB PRO 58 12.735 14.518 64.423 1.00 0.00

ATOM 3678 HB1 PRO 58 13.751 14.525 64.815 1.00 0.00

ATOM 3679 HB2 PRO 58 12.190 15.289 64.968 1.00 0.00

ATOM 3680 CA PRO 58 12.867 14.736 62.919 1.00 0.00

ATOM 3681 HA PRO 58 11.906 15.131 62.595 1.00 0.00

ATOM 3682 C PRO 58 13.900 15.811 62.605 1.00 0.00

ATOM 3683 O PRO 58 15.084 15.594 62.849 1.00 0.00

ATOM 3684 N PRO 59 13.573 16.927 61.952 1.00 0.00

ATOM 3685 CD PRO 59 12.291 17.250 61.358 1.00 0.00

ATOM 3686 HD1 PRO 59 11.446 17.107 62.031 1.00 0.00

ATOM 3687 HD2 PRO 59 12.132 16.596 60.500 1.00 0.00

ATOM 3688 CG PRO 59 12.381 18.707 60.912 1.00 0.00

ATOM 3689 HG1 PRO 59 12.089 19.439 61.666 1.00 0.00

ATOM 3690 HG2 PRO 59 11.903 18.895 59.952 1.00 0.00

ATOM 3691 CB PRO 59 13.861 18.873 60.580 1.00 0.00

ATOM 3692 HB1 PRO 59 14.080 19.925 60.766 1.00 0.00

ATOM 3693 HB2 PRO 59 14.064 18.648 59.535 1.00 0.00

ATOM 3694 CA PRO 59 14.573 17.858 61.470 1.00 0.00

ATOM 3695 HA PRO 59 15.395 17.320 60.997 1.00 0.00

ATOM 3696 C PRO 59 15.154 18.564 62.687 1.00 0.00

ATOM 3697 O PRO 59 16.269 19.054 62.533 1.00 0.00

ATOM 3698 N GLU 60 14.462 18.701 63.820 1.00 0.00

ATOM 3699 H GLU 60 13.571 18.233 63.753 1.00 0.00

ATOM 3700 CA GLU 60 14.702 19.608 64.926 1.00 0.00

ATOM 3701 HA GLU 60 13.799 19.705 65.529 1.00 0.00

ATOM 3702 CB GLU 60 15.830 19.053 65.791 1.00 0.00

ATOM 3703 HB1 GLU 60 15.987 19.780 66.587 1.00 0.00

ATOM 3704 HB2 GLU 60 16.788 19.015 65.272 1.00 0.00

ATOM 3705 CG GLU 60 15.643 17.703 66.477 1.00 0.00

ATOM 3706 HG1 GLU 60 14.869 17.759 67.242 1.00 0.00

ATOM 3707 HG2 GLU 60 15.274 16.948 65.782 1.00 0.00

ATOM 3708 CD GLU 60 16.904 17.336 67.246 1.00 0.00

ATOM 3709 OE1 GLU 60 17.410 16.225 66.977 1.00 0.00

ATOM 3710 OE2 GLU 60 17.533 18.196 67.898 1.00 0.00

ATOM 3711 C GLU 60 14.945 21.036 64.460 1.00 0.00

ATOM 3712 O GLU 60 15.877 21.753 64.819 1.00 0.00

ATOM 3713 N SER 61 14.007 21.418 63.592 1.00 0.00

ATOM 3714 H SER 61 13.320 20.746 63.279 1.00 0.00

ATOM 3715 CA SER 61 14.053 22.675 62.871 1.00 0.00

ATOM 3716 HA SER 61 14.275 23.498 63.548 1.00 0.00

ATOM 3717 CB SER 61 14.968 22.588 61.654 1.00 0.00

ATOM 3718 HB1 SER 61 14.512 21.880 60.961 1.00 0.00

ATOM 3719 HB2 SER 61 15.009 23.617 61.315 1.00 0.00

ATOM 3720 OG SER 61 16.265 22.221 62.067 1.00 0.00

ATOM 3721 HG SER 61 16.256 21.457 62.649 1.00 0.00

ATOM 3722 C SER 61 12.727 23.196 62.335 1.00 0.00

ATOM 3723 OC1 SER 61 12.261 24.240 62.841 1.00 0.00

ATOM 3724 OC2 SER 61 12.042 22.526 61.532 1.00 0.00

ATOM 3725 N PHE 1 28.708 36.141 133.649 1.00 0.00

ATOM 3726 H1 PHE 1 28.337 35.373 134.190 1.00 0.00

ATOM 3727 H2 PHE 1 29.440 35.832 133.025 1.00 0.00

ATOM 3728 H3 PHE 1 27.927 36.521 133.134 1.00 0.00

ATOM 3729 CA PHE 1 29.152 37.129 134.645 1.00 0.00

ATOM 3730 HA PHE 1 29.675 37.879 134.052 1.00 0.00

ATOM 3731 CB PHE 1 27.944 37.818 135.271 1.00 0.00

ATOM 3732 HB1 PHE 1 27.269 37.076 135.698 1.00 0.00

ATOM 3733 HB2 PHE 1 27.437 38.342 134.461 1.00 0.00

ATOM 3734 CG PHE 1 28.245 38.885 136.296 1.00 0.00

ATOM 3735 CD1 PHE 1 28.312 38.663 137.677 1.00 0.00

ATOM 3736 HD1 PHE 1 28.236 37.651 138.045 1.00 0.00

ATOM 3737 CE1 PHE 1 28.440 39.741 138.561 1.00 0.00

ATOM 3738 HE1 PHE 1 28.428 39.606 139.633 1.00 0.00

ATOM 3739 CZ PHE 1 28.452 41.048 138.054 1.00 0.00

ATOM 3740 HZ PHE 1 28.463 41.998 138.568 1.00 0.00

ATOM 3741 CE2 PHE 1 28.434 41.290 136.676 1.00 0.00

ATOM 3742 HE2 PHE 1 28.582 42.232 136.169 1.00 0.00

ATOM 3743 CD2 PHE 1 28.267 40.200 135.815 1.00 0.00

ATOM 3744 HD2 PHE 1 28.220 40.306 134.742 1.00 0.00

ATOM 3745 C PHE 1 30.155 36.594 135.658 1.00 0.00

ATOM 3746 O PHE 1 29.803 35.989 136.667 1.00 0.00

ATOM 3747 N THR 2 31.428 36.815 135.325 1.00 0.00

ATOM 3748 H THR 2 31.601 37.325 134.470 1.00 0.00

ATOM 3749 CA THR 2 32.578 36.382 136.094 1.00 0.00

ATOM 3750 HA THR 2 32.311 36.573 137.132 1.00 0.00

ATOM 3751 CB THR 2 32.738 34.866 136.010 1.00 0.00

ATOM 3752 HB THR 2 31.728 34.470 136.110 1.00 0.00

ATOM 3753 CG2 THR 2 33.156 34.467 134.598 1.00 0.00

ATOM 3754 1HG2 THR 2 34.134 34.918 134.428 1.00 0.00

ATOM 3755 2HG2 THR 2 32.401 34.856 133.916 1.00 0.00

ATOM 3756 3HG2 THR 2 33.059 33.400 134.401 1.00 0.00

ATOM 3757 OG1 THR 2 33.583 34.337 137.008 1.00 0.00

ATOM 3758 HG1 THR 2 33.055 33.829 137.627 1.00 0.00

ATOM 3759 C THR 2 33.850 37.097 135.657 1.00 0.00

ATOM 3760 O THR 2 33.917 37.645 134.559 1.00 0.00

ATOM 3761 N LEU 3 34.935 37.101 136.434 1.00 0.00

ATOM 3762 H LEU 3 34.937 36.656 137.340 1.00 0.00

ATOM 3763 CA LEU 3 36.224 37.692 136.133 1.00 0.00

ATOM 3764 HA LEU 3 36.159 38.328 135.250 1.00 0.00

ATOM 3765 CB LEU 3 36.675 38.475 137.362 1.00 0.00

ATOM 3766 HB1 LEU 3 36.740 37.783 138.202 1.00 0.00

ATOM 3767 HB2 LEU 3 35.841 39.147 137.559 1.00 0.00

ATOM 3768 CG LEU 3 37.950 39.314 137.394 1.00 0.00

ATOM 3769 HG LEU 3 38.726 38.615 137.103 1.00 0.00

ATOM 3770 CD1 LEU 3 37.780 40.588 136.571 1.00 0.00

ATOM 3771 1HD1 LEU 3 37.587 40.354 135.524 1.00 0.00

ATOM 3772 2HD1 LEU 3 38.711 41.151 136.641 1.00 0.00

ATOM 3773 3HD1 LEU 3 36.934 41.193 136.897 1.00 0.00

ATOM 3774 CD2 LEU 3 38.186 39.847 138.804 1.00 0.00

ATOM 3775 1HD2 LEU 3 39.119 40.393 138.944 1.00 0.00

ATOM 3776 2HD2 LEU 3 38.102 38.999 139.484 1.00 0.00

ATOM 3777 3HD2 LEU 3 37.361 40.495 139.100 1.00 0.00

ATOM 3778 C LEU 3 37.236 36.605 135.801 1.00 0.00

ATOM 3779 O LEU 3 38.221 36.881 135.121 1.00 0.00

ATOM 3780 N ILE 4 37.148 35.389 136.346 1.00 0.00

ATOM 3781 H ILE 4 36.432 35.175 137.024 1.00 0.00

ATOM 3782 CA ILE 4 38.027 34.288 136.004 1.00 0.00

ATOM 3783 HA ILE 4 39.051 34.641 136.120 1.00 0.00

ATOM 3784 CB ILE 4 37.793 33.116 136.953 1.00 0.00

ATOM 3785 HB ILE 4 37.735 33.543 137.954 1.00 0.00

ATOM 3786 CG2 ILE 4 36.535 32.298 136.677 1.00 0.00

ATOM 3787 1HG2 ILE 4 35.632 32.854 136.930 1.00 0.00

ATOM 3788 2HG2 ILE 4 36.687 31.412 137.293 1.00 0.00

ATOM 3789 3HG2 ILE 4 36.519 32.027 135.621 1.00 0.00

ATOM 3790 CG1 ILE 4 39.075 32.290 137.018 1.00 0.00

ATOM 3791 1HG1 ILE 4 39.210 31.667 136.135 1.00 0.00

ATOM 3792 2HG1 ILE 4 39.857 33.037 136.882 1.00 0.00

ATOM 3793 CD ILE 4 39.301 31.396 138.234 1.00 0.00

ATOM 3794 HD1 ILE 4 38.502 30.656 138.187 1.00 0.00

ATOM 3795 HD2 ILE 4 39.361 32.061 139.095 1.00 0.00

ATOM 3796 HD3 ILE 4 40.257 30.897 138.168 1.00 0.00

ATOM 3797 C ILE 4 38.027 33.890 134.535 1.00 0.00

ATOM 3798 O ILE 4 38.960 33.228 134.086 1.00 0.00

ATOM 3799 N GLU 5 36.962 34.223 133.801 1.00 0.00

ATOM 3800 H GLU 5 36.195 34.708 134.243 1.00 0.00

ATOM 3801 CA GLU 5 36.914 33.892 132.391 1.00 0.00

ATOM 3802 HA GLU 5 36.999 32.807 132.325 1.00 0.00

ATOM 3803 CB GLU 5 35.541 34.169 131.784 1.00 0.00

ATOM 3804 HB1 GLU 5 34.783 33.590 132.312 1.00 0.00

ATOM 3805 HB2 GLU 5 35.583 33.775 130.769 1.00 0.00

ATOM 3806 CG GLU 5 35.170 35.644 131.655 1.00 0.00

ATOM 3807 HG1 GLU 5 35.138 36.071 132.656 1.00 0.00

ATOM 3808 HG2 GLU 5 35.997 36.221 131.239 1.00 0.00

ATOM 3809 CD GLU 5 33.872 35.989 130.939 1.00 0.00

ATOM 3810 OE1 GLU 5 33.839 37.022 130.236 1.00 0.00

ATOM 3811 OE2 GLU 5 32.847 35.300 131.121 1.00 0.00

ATOM 3812 C GLU 5 38.102 34.462 131.630 1.00 0.00

ATOM 3813 O GLU 5 38.290 33.936 130.536 1.00 0.00

ATOM 3814 N LEU 6 38.806 35.504 132.082 1.00 0.00

ATOM 3815 H LEU 6 38.682 35.823 133.032 1.00 0.00

ATOM 3816 CA LEU 6 39.977 36.001 131.389 1.00 0.00

ATOM 3817 HA LEU 6 39.831 36.035 130.309 1.00 0.00

ATOM 3818 CB LEU 6 40.287 37.395 131.926 1.00 0.00

ATOM 3819 HB1 LEU 6 41.321 37.649 131.694 1.00 0.00

ATOM 3820 HB2 LEU 6 40.185 37.369 133.011 1.00 0.00

ATOM 3821 CG LEU 6 39.475 38.563 131.373 1.00 0.00

ATOM 3822 HG LEU 6 39.751 39.407 132.006 1.00 0.00

ATOM 3823 CD1 LEU 6 39.954 38.858 129.954 1.00 0.00

ATOM 3824 1HD1 LEU 6 39.597 38.055 129.310 1.00 0.00

ATOM 3825 2HD1 LEU 6 41.004 39.151 129.905 1.00 0.00

ATOM 3826 3HD1 LEU 6 39.423 39.735 129.584 1.00 0.00

ATOM 3827 CD2 LEU 6 37.956 38.450 131.449 1.00 0.00

ATOM 3828 1HD2 LEU 6 37.486 39.428 131.336 1.00 0.00

ATOM 3829 2HD2 LEU 6 37.600 37.937 132.343 1.00 0.00

ATOM 3830 3HD2 LEU 6 37.640 37.795 130.638 1.00 0.00

ATOM 3831 C LEU 6 41.112 34.986 131.443 1.00 0.00

ATOM 3832 O LEU 6 41.969 34.983 130.564 1.00 0.00

ATOM 3833 N LEU 7 41.104 33.913 132.237 1.00 0.00

ATOM 3834 H LEU 7 40.387 33.822 132.941 1.00 0.00

ATOM 3835 CA LEU 7 42.001 32.776 132.297 1.00 0.00

ATOM 3836 HA LEU 7 43.011 33.183 132.330 1.00 0.00

ATOM 3837 CB LEU 7 41.708 31.921 133.528 1.00 0.00

ATOM 3838 HB1 LEU 7 40.806 31.322 133.411 1.00 0.00

ATOM 3839 HB2 LEU 7 41.464 32.630 134.319 1.00 0.00

ATOM 3840 CG LEU 7 42.831 30.989 133.977 1.00 0.00

ATOM 3841 HG LEU 7 43.354 30.415 133.213 1.00 0.00

ATOM 3842 CD1 LEU 7 43.943 31.771 134.668 1.00 0.00

ATOM 3843 1HD1 LEU 7 43.526 32.252 135.551 1.00 0.00

ATOM 3844 2HD1 LEU 7 44.439 32.477 134.002 1.00 0.00

ATOM 3845 3HD1 LEU 7 44.725 31.061 134.939 1.00 0.00

ATOM 3846 CD2 LEU 7 42.256 29.943 134.930 1.00 0.00

ATOM 3847 1HD2 LEU 7 41.380 29.448 134.513 1.00 0.00

ATOM 3848 2HD2 LEU 7 41.840 30.425 135.812 1.00 0.00

ATOM 3849 3HD2 LEU 7 42.969 29.180 135.242 1.00 0.00

ATOM 3850 C LEU 7 41.836 31.970 131.017 1.00 0.00

ATOM 3851 O LEU 7 42.779 31.343 130.539 1.00 0.00

ATOM 3852 N ILE 8 40.605 31.949 130.501 1.00 0.00

ATOM 3853 H ILE 8 39.852 32.382 131.016 1.00 0.00

ATOM 3854 CA ILE 8 40.267 31.187 129.314 1.00 0.00

ATOM 3855 HA ILE 8 40.870 30.282 129.246 1.00 0.00

ATOM 3856 CB ILE 8 38.783 30.862 129.161 1.00 0.00

ATOM 3857 HB ILE 8 38.133 31.714 128.963 1.00 0.00

ATOM 3858 CG2 ILE 8 38.496 29.859 128.048 1.00 0.00

ATOM 3859 1HG2 ILE 8 37.466 29.519 128.160 1.00 0.00

ATOM 3860 2HG2 ILE 8 39.213 29.039 128.090 1.00 0.00

ATOM 3861 3HG2 ILE 8 38.576 30.320 127.064 1.00 0.00

ATOM 3862 CG1 ILE 8 38.241 30.301 130.473 1.00 0.00

ATOM 3863 1HG1 ILE 8 38.687 29.327 130.671 1.00 0.00

ATOM 3864 2HG1 ILE 8 38.619 30.900 131.300 1.00 0.00

ATOM 3865 CD ILE 8 36.742 30.430 130.725 1.00 0.00

ATOM 3866 HD1 ILE 8 36.344 31.367 130.336 1.00 0.00

ATOM 3867 HD2 ILE 8 36.396 30.338 131.756 1.00 0.00

ATOM 3868 HD3 ILE 8 36.400 29.661 130.034 1.00 0.00

ATOM 3869 C ILE 8 40.612 32.069 128.124 1.00 0.00

ATOM 3870 O ILE 8 41.183 31.558 127.164 1.00 0.00

ATOM 3871 N VAL 9 40.098 33.301 128.191 1.00 0.00

ATOM 3872 H VAL 9 39.523 33.452 129.008 1.00 0.00

ATOM 3873 CA VAL 9 40.207 34.253 127.104 1.00 0.00

ATOM 3874 HA VAL 9 39.793 33.720 126.249 1.00 0.00

ATOM 3875 CB VAL 9 39.391 35.533 127.264 1.00 0.00

ATOM 3876 HB VAL 9 39.862 36.167 128.016 1.00 0.00

ATOM 3877 CG1 VAL 9 39.496 36.237 125.915 1.00 0.00

ATOM 3878 1HG1 VAL 9 39.229 35.633 125.050 1.00 0.00

ATOM 3879 2HG1 VAL 9 40.498 36.589 125.670 1.00 0.00

ATOM 3880 3HG1 VAL 9 38.904 37.149 125.976 1.00 0.00

ATOM 3881 CG2 VAL 9 37.902 35.248 127.447 1.00 0.00

ATOM 3882 1HG2 VAL 9 37.374 34.522 126.831 1.00 0.00

ATOM 3883 2HG2 VAL 9 37.472 36.243 127.338 1.00 0.00

ATOM 3884 3HG2 VAL 9 37.755 35.029 128.503 1.00 0.00

ATOM 3885 C VAL 9 41.680 34.386 126.743 1.00 0.00

ATOM 3886 O VAL 9 42.111 34.231 125.603 1.00 0.00

ATOM 3887 N VAL 10 42.533 34.499 127.763 1.00 0.00

ATOM 3888 H VAL 10 42.102 34.487 128.676 1.00 0.00

ATOM 3889 CA VAL 10 43.964 34.688 127.620 1.00 0.00

ATOM 3890 HA VAL 10 44.167 35.451 126.868 1.00 0.00

ATOM 3891 CB VAL 10 44.628 35.184 128.901 1.00 0.00

ATOM 3892 HB VAL 10 43.922 35.884 129.346 1.00 0.00

ATOM 3893 CG1 VAL 10 44.828 34.072 129.927 1.00 0.00

ATOM 3894 1HG1 VAL 10 45.797 33.572 129.893 1.00 0.00

ATOM 3895 2HG1 VAL 10 43.983 33.386 129.974 1.00 0.00

ATOM 3896 3HG1 VAL 10 44.831 34.476 130.938 1.00 0.00

ATOM 3897 CG2 VAL 10 45.983 35.866 128.731 1.00 0.00

ATOM 3898 1HG2 VAL 10 46.351 36.221 129.693 1.00 0.00

ATOM 3899 2HG2 VAL 10 45.897 36.817 128.206 1.00 0.00

ATOM 3900 3HG2 VAL 10 46.681 35.111 128.371 1.00 0.00

ATOM 3901 C VAL 10 44.664 33.474 127.028 1.00 0.00

ATOM 3902 O VAL 10 45.775 33.617 126.522 1.00 0.00

ATOM 3903 N ALA 11 44.056 32.285 127.016 1.00 0.00

ATOM 3904 H ALA 11 43.086 32.179 127.274 1.00 0.00

ATOM 3905 CA ALA 11 44.656 31.096 126.446 1.00 0.00

ATOM 3906 HA ALA 11 45.739 31.205 126.385 1.00 0.00

ATOM 3907 CB ALA 11 44.236 29.910 127.310 1.00 0.00

ATOM 3908 HB1 ALA 11 43.223 29.813 127.704 1.00 0.00

ATOM 3909 HB2 ALA 11 44.894 29.908 128.179 1.00 0.00

ATOM 3910 HB3 ALA 11 44.523 29.007 126.773 1.00 0.00

ATOM 3911 C ALA 11 44.155 30.964 125.014 1.00 0.00

ATOM 3912 O ALA 11 44.951 30.668 124.126 1.00 0.00

ATOM 3913 N ILE 12 42.892 31.241 124.685 1.00 0.00

ATOM 3914 H ILE 12 42.364 31.638 125.447 1.00 0.00

ATOM 3915 CA ILE 12 42.390 31.236 123.324 1.00 0.00

ATOM 3916 HA ILE 12 42.629 30.278 122.862 1.00 0.00

ATOM 3917 CB ILE 12 40.871 31.256 123.186 1.00 0.00

ATOM 3918 HB ILE 12 40.771 31.058 122.119 1.00 0.00

ATOM 3919 CG2 ILE 12 40.244 30.132 124.007 1.00 0.00

ATOM 3920 1HG2 ILE 12 40.818 29.212 123.892 1.00 0.00

ATOM 3921 2HG2 ILE 12 39.247 29.922 123.625 1.00 0.00

ATOM 3922 3HG2 ILE 12 40.045 30.304 125.065 1.00 0.00

ATOM 3923 CG1 ILE 12 40.209 32.570 123.595 1.00 0.00

ATOM 3924 1HG1 ILE 12 40.065 32.631 124.674 1.00 0.00

ATOM 3925 2HG1 ILE 12 40.762 33.491 123.416 1.00 0.00

ATOM 3926 CD ILE 12 38.898 32.726 122.830 1.00 0.00

ATOM 3927 HD1 ILE 12 38.238 33.540 123.127 1.00 0.00

ATOM 3928 HD2 ILE 12 38.427 31.745 122.902 1.00 0.00

ATOM 3929 HD3 ILE 12 39.127 32.939 121.786 1.00 0.00

ATOM 3930 C ILE 12 42.978 32.322 122.433 1.00 0.00

ATOM 3931 O ILE 12 43.065 32.153 121.220 1.00 0.00

ATOM 3932 N ILE 13 43.518 33.417 122.972 1.00 0.00

ATOM 3933 H ILE 13 43.371 33.467 123.968 1.00 0.00

ATOM 3934 CA ILE 13 44.005 34.590 122.270 1.00 0.00

ATOM 3935 HA ILE 13 43.585 34.669 121.268 1.00 0.00

ATOM 3936 CB ILE 13 43.780 35.918 122.990 1.00 0.00

ATOM 3937 HB ILE 13 42.738 35.874 123.303 1.00 0.00

ATOM 3938 CG2 ILE 13 44.692 35.998 124.213 1.00 0.00

ATOM 3939 1HG2 ILE 13 44.730 35.034 124.718 1.00 0.00

ATOM 3940 2HG2 ILE 13 44.252 36.748 124.871 1.00 0.00

ATOM 3941 3HG2 ILE 13 45.689 36.279 123.876 1.00 0.00

ATOM 3942 CG1 ILE 13 43.784 37.117 122.046 1.00 0.00

ATOM 3943 1HG1 ILE 13 44.706 37.099 121.465 1.00 0.00

ATOM 3944 2HG1 ILE 13 42.969 36.993 121.333 1.00 0.00

ATOM 3945 CD ILE 13 43.661 38.437 122.801 1.00 0.00

ATOM 3946 HD1 ILE 13 43.199 39.111 122.081 1.00 0.00

ATOM 3947 HD2 ILE 13 44.658 38.845 122.961 1.00 0.00

ATOM 3948 HD3 ILE 13 42.951 38.449 123.629 1.00 0.00

ATOM 3949 C ILE 13 45.475 34.388 121.934 1.00 0.00

ATOM 3950 O ILE 13 45.919 34.744 120.845 1.00 0.00

ATOM 3951 N GLY 14 46.217 33.650 122.764 1.00 0.00

ATOM 3952 H GLY 14 45.885 33.395 123.682 1.00 0.00

ATOM 3953 CA GLY 14 47.581 33.211 122.547 1.00 0.00

ATOM 3954 HA1 GLY 14 48.203 34.052 122.245 1.00 0.00

ATOM 3955 HA2 GLY 14 48.090 32.932 123.467 1.00 0.00

ATOM 3956 C GLY 14 47.697 32.176 121.438 1.00 0.00

ATOM 3957 O GLY 14 48.586 32.259 120.594 1.00 0.00

ATOM 3958 N ILE 15 46.822 31.169 121.395 1.00 0.00

ATOM 3959 H ILE 15 45.981 31.384 121.910 1.00 0.00

ATOM 3960 CA ILE 15 46.913 30.014 120.524 1.00 0.00

ATOM 3961 HA ILE 15 47.930 29.633 120.604 1.00 0.00

ATOM 3962 CB ILE 15 46.038 28.821 120.900 1.00 0.00

ATOM 3963 HB ILE 15 46.186 28.035 120.159 1.00 0.00

ATOM 3964 CG2 ILE 15 46.475 28.236 122.240 1.00 0.00

ATOM 3965 1HG2 ILE 15 45.943 27.319 122.498 1.00 0.00

ATOM 3966 2HG2 ILE 15 46.370 28.973 123.036 1.00 0.00

ATOM 3967 3HG2 ILE 15 47.538 27.995 122.217 1.00 0.00

ATOM 3968 CG1 ILE 15 44.578 29.267 120.866 1.00 0.00

ATOM 3969 1HG1 ILE 15 44.329 30.040 121.592 1.00 0.00

ATOM 3970 2HG1 ILE 15 44.195 29.574 119.894 1.00 0.00

ATOM 3971 CD ILE 15 43.604 28.132 121.172 1.00 0.00

ATOM 3972 HD1 ILE 15 43.811 27.270 120.539 1.00 0.00

ATOM 3973 HD2 ILE 15 42.584 28.479 121.008 1.00 0.00

ATOM 3974 HD3 ILE 15 43.667 27.697 122.169 1.00 0.00

ATOM 3975 C ILE 15 46.817 30.416 119.058 1.00 0.00

ATOM 3976 O ILE 15 47.160 29.677 118.137 1.00 0.00

ATOM 3977 N LEU 16 46.321 31.614 118.745 1.00 0.00

ATOM 3978 H LEU 16 46.129 32.250 119.506 1.00 0.00

ATOM 3979 CA LEU 16 46.167 32.137 117.402 1.00 0.00

ATOM 3980 HA LEU 16 45.534 31.497 116.788 1.00 0.00

ATOM 3981 CB LEU 16 45.565 33.534 117.529 1.00 0.00

ATOM 3982 HB1 LEU 16 45.529 34.042 116.566 1.00 0.00

ATOM 3983 HB2 LEU 16 46.231 34.181 118.099 1.00 0.00

ATOM 3984 CG LEU 16 44.202 33.662 118.202 1.00 0.00

ATOM 3985 HG LEU 16 44.338 33.292 119.218 1.00 0.00

ATOM 3986 CD1 LEU 16 43.664 35.090 118.238 1.00 0.00

ATOM 3987 1HD1 LEU 16 42.814 35.295 118.888 1.00 0.00

ATOM 3988 2HD1 LEU 16 43.427 35.488 117.251 1.00 0.00

ATOM 3989 3HD1 LEU 16 44.549 35.594 118.627 1.00 0.00

ATOM 3990 CD2 LEU 16 43.164 32.845 117.437 1.00 0.00

ATOM 3991 1HD2 LEU 16 42.226 32.986 117.973 1.00 0.00

ATOM 3992 2HD2 LEU 16 43.443 31.793 117.496 1.00 0.00

ATOM 3993 3HD2 LEU 16 43.060 33.170 116.402 1.00 0.00

ATOM 3994 C LEU 16 47.490 32.123 116.650 1.00 0.00

ATOM 3995 O LEU 16 47.485 32.328 115.438 1.00 0.00

ATOM 3996 N ALA 17 48.599 32.039 117.389 1.00 0.00

ATOM 3997 H ALA 17 48.486 31.768 118.355 1.00 0.00

ATOM 3998 CA ALA 17 49.933 31.942 116.830 1.00 0.00

ATOM 3999 HA ALA 17 50.216 32.858 116.327 1.00 0.00

ATOM 4000 CB ALA 17 50.896 31.917 118.013 1.00 0.00

ATOM 4001 HB1 ALA 17 51.930 31.805 117.688 1.00 0.00

ATOM 4002 HB2 ALA 17 50.630 31.130 118.717 1.00 0.00

ATOM 4003 HB3 ALA 17 50.661 32.862 118.501 1.00 0.00

ATOM 4004 C ALA 17 50.122 30.757 115.894 1.00 0.00

ATOM 4005 O ALA 17 51.081 30.779 115.125 1.00 0.00

ATOM 4006 N ALA 18 49.244 29.753 115.950 1.00 0.00

ATOM 4007 H ALA 18 48.631 29.796 116.752 1.00 0.00

ATOM 4008 CA ALA 18 49.196 28.610 115.062 1.00 0.00

ATOM 4009 HA ALA 18 49.918 28.691 114.249 1.00 0.00

ATOM 4010 CB ALA 18 49.619 27.376 115.854 1.00 0.00

ATOM 4011 HB1 ALA 18 49.047 27.234 116.772 1.00 0.00

ATOM 4012 HB2 ALA 18 49.540 26.494 115.219 1.00 0.00

ATOM 4013 HB3 ALA 18 50.636 27.425 116.240 1.00 0.00

ATOM 4014 C ALA 18 47.852 28.374 114.386 1.00 0.00

ATOM 4015 O ALA 18 47.872 27.573 113.455 1.00 0.00

ATOM 4016 N ILE 19 46.741 29.047 114.691 1.00 0.00

ATOM 4017 H ILE 19 46.895 29.649 115.487 1.00 0.00

ATOM 4018 CA ILE 19 45.435 28.813 114.105 1.00 0.00

ATOM 4019 HA ILE 19 45.472 28.152 113.239 1.00 0.00

ATOM 4020 CB ILE 19 44.590 28.054 115.124 1.00 0.00

ATOM 4021 HB ILE 19 43.615 27.940 114.652 1.00 0.00

ATOM 4022 CG2 ILE 19 45.091 26.638 115.396 1.00 0.00

ATOM 4023 1HG2 ILE 19 46.038 26.640 115.929 1.00 0.00

ATOM 4024 2HG2 ILE 19 45.204 26.215 114.398 1.00 0.00

ATOM 4025 3HG2 ILE 19 44.404 26.079 116.032 1.00 0.00

ATOM 4026 CG1 ILE 19 44.313 28.911 116.357 1.00 0.00

ATOM 4027 1HG1 ILE 19 45.196 29.156 116.946 1.00 0.00

ATOM 4028 2HG1 ILE 19 43.761 29.836 116.187 1.00 0.00

ATOM 4029 CD ILE 19 43.382 28.261 117.376 1.00 0.00

ATOM 4030 HD1 ILE 19 43.945 27.672 118.101 1.00 0.00

ATOM 4031 HD2 ILE 19 42.683 27.665 116.788 1.00 0.00

ATOM 4032 HD3 ILE 19 42.952 29.058 117.975 1.00 0.00

ATOM 4033 C ILE 19 44.758 30.104 113.666 1.00 0.00

ATOM 4034 O ILE 19 43.547 30.141 113.462 1.00 0.00

ATOM 4035 N ALA 20 45.481 31.210 113.482 1.00 0.00

ATOM 4036 H ALA 20 46.471 31.011 113.485 1.00 0.00

ATOM 4037 CA ALA 20 44.928 32.328 112.744 1.00 0.00

ATOM 4038 HA ALA 20 44.351 31.947 111.904 1.00 0.00

ATOM 4039 CB ALA 20 44.015 33.199 113.604 1.00 0.00

ATOM 4040 HB1 ALA 20 43.225 32.603 114.059 1.00 0.00

ATOM 4041 HB2 ALA 20 44.591 33.609 114.433 1.00 0.00

ATOM 4042 HB3 ALA 20 43.624 34.051 113.049 1.00 0.00

ATOM 4043 C ALA 20 45.994 33.210 112.108 1.00 0.00

ATOM 4044 O ALA 20 45.812 33.731 111.011 1.00 0.00

ATOM 4045 N ILE 21 47.119 33.488 112.771 1.00 0.00

ATOM 4046 H ILE 21 47.239 33.008 113.650 1.00 0.00

ATOM 4047 CA ILE 21 48.071 34.541 112.478 1.00 0.00

ATOM 4048 HA ILE 21 47.508 35.421 112.166 1.00 0.00

ATOM 4049 CB ILE 21 48.846 35.000 113.709 1.00 0.00

ATOM 4050 HB ILE 21 49.480 34.202 114.097 1.00 0.00

ATOM 4051 CG2 ILE 21 49.742 36.134 113.221 1.00 0.00

ATOM 4052 1HG2 ILE 21 49.275 36.861 112.556 1.00 0.00

ATOM 4053 2HG2 ILE 21 50.517 35.651 112.628 1.00 0.00

ATOM 4054 3HG2 ILE 21 50.100 36.689 114.089 1.00 0.00

ATOM 4055 CG1 ILE 21 47.892 35.421 114.823 1.00 0.00

ATOM 4056 1HG1 ILE 21 47.284 36.251 114.461 1.00 0.00

ATOM 4057 2HG1 ILE 21 47.252 34.563 115.023 1.00 0.00

ATOM 4058 CD ILE 21 48.616 35.817 116.107 1.00 0.00

ATOM 4059 HD1 ILE 21 49.439 35.122 116.232 1.00 0.00

ATOM 4060 HD2 ILE 21 48.042 35.587 117.004 1.00 0.00

ATOM 4061 HD3 ILE 21 48.985 36.840 116.183 1.00 0.00

ATOM 4062 C ILE 21 48.937 34.069 111.318 1.00 0.00

ATOM 4063 O ILE 21 49.062 34.857 110.383 1.00 0.00

ATOM 4064 N PRO 22 49.415 32.823 111.284 1.00 0.00

ATOM 4065 CD PRO 22 49.586 31.815 112.311 1.00 0.00

ATOM 4066 HD1 PRO 22 48.774 31.089 112.344 1.00 0.00

ATOM 4067 HD2 PRO 22 49.739 32.271 113.290 1.00 0.00

ATOM 4068 CG PRO 22 50.830 31.067 111.841 1.00 0.00

ATOM 4069 HG1 PRO 22 50.906 30.049 112.223 1.00 0.00

ATOM 4070 HG2 PRO 22 51.702 31.671 112.086 1.00 0.00

ATOM 4071 CB PRO 22 50.579 31.010 110.336 1.00 0.00

ATOM 4072 HB1 PRO 22 49.895 30.263 109.934 1.00 0.00

ATOM 4073 HB2 PRO 22 51.471 30.854 109.729 1.00 0.00

ATOM 4074 CA PRO 22 50.020 32.396 110.038 1.00 0.00

ATOM 4075 HA PRO 22 50.845 33.092 109.888 1.00 0.00

ATOM 4076 C PRO 22 49.147 32.244 108.800 1.00 0.00

ATOM 4077 O PRO 22 49.513 32.780 107.757 1.00 0.00

ATOM 4078 N GLN 23 47.925 31.731 108.968 1.00 0.00

ATOM 4079 H GLN 23 47.684 31.471 109.913 1.00 0.00

ATOM 4080 CA GLN 23 46.964 31.467 107.916 1.00 0.00

ATOM 4081 HA GLN 23 47.507 30.961 107.117 1.00 0.00

ATOM 4082 CB GLN 23 45.834 30.551 108.378 1.00 0.00

ATOM 4083 HB1 GLN 23 45.309 30.291 107.460 1.00 0.00

ATOM 4084 HB2 GLN 23 45.091 31.037 109.003 1.00 0.00

ATOM 4085 CG GLN 23 46.112 29.172 108.971 1.00 0.00

ATOM 4086 HG1 GLN 23 45.202 28.573 109.002 1.00 0.00

ATOM 4087 HG2 GLN 23 46.713 28.669 108.213 1.00 0.00

ATOM 4088 CD GLN 23 46.659 29.186 110.391 1.00 0.00

ATOM 4089 OE1 GLN 23 46.686 30.198 111.091 1.00 0.00

ATOM 4090 NE2 GLN 23 47.054 28.005 110.868 1.00 0.00

ATOM 4091 1HE2 GLN 23 47.071 27.201 110.258 1.00 0.00

ATOM 4092 2HE2 GLN 23 47.534 27.967 111.757 1.00 0.00

ATOM 4093 C GLN 23 46.454 32.728 107.234 1.00 0.00

ATOM 4094 O GLN 23 46.172 32.721 106.038 1.00 0.00

ATOM 4095 N PHE 24 46.425 33.858 107.945 1.00 0.00

ATOM 4096 H PHE 24 46.580 33.827 108.943 1.00 0.00

ATOM 4097 CA PHE 24 45.983 35.152 107.463 1.00 0.00

ATOM 4098 HA PHE 24 45.090 35.025 106.849 1.00 0.00

ATOM 4099 CB PHE 24 45.487 35.969 108.651 1.00 0.00

ATOM 4100 HB1 PHE 24 46.264 35.992 109.415 1.00 0.00

ATOM 4101 HB2 PHE 24 44.674 35.336 109.006 1.00 0.00

ATOM 4102 CG PHE 24 44.941 37.345 108.349 1.00 0.00

ATOM 4103 CD1 PHE 24 43.576 37.511 108.095 1.00 0.00

ATOM 4104 HD1 PHE 24 43.010 36.593 108.053 1.00 0.00

ATOM 4105 CE1 PHE 24 43.057 38.791 107.867 1.00 0.00

ATOM 4106 HE1 PHE 24 41.993 38.903 107.717 1.00 0.00

ATOM 4107 CZ PHE 24 43.957 39.861 107.800 1.00 0.00

ATOM 4108 HZ PHE 24 43.622 40.859 107.566 1.00 0.00

ATOM 4109 CE2 PHE 24 45.322 39.724 108.077 1.00 0.00

ATOM 4110 HE2 PHE 24 45.934 40.588 107.864 1.00 0.00

ATOM 4111 CD2 PHE 24 45.817 38.439 108.328 1.00 0.00

ATOM 4112 HD2 PHE 24 46.890 38.386 108.438 1.00 0.00

ATOM 4113 C PHE 24 47.019 35.991 106.731 1.00 0.00

ATOM 4114 O PHE 24 46.700 36.414 105.621 1.00 0.00

ATOM 4115 N SER 25 48.265 35.950 107.207 1.00 0.00

ATOM 4116 H SER 25 48.455 35.518 108.100 1.00 0.00

ATOM 4117 CA SER 25 49.409 36.339 106.408 1.00 0.00

ATOM 4118 HA SER 25 49.325 37.385 106.114 1.00 0.00

ATOM 4119 CB SER 25 50.667 36.073 107.230 1.00 0.00

ATOM 4120 HB1 SER 25 51.502 36.087 106.529 1.00 0.00

ATOM 4121 HB2 SER 25 50.640 35.088 107.695 1.00 0.00

ATOM 4122 OG SER 25 50.874 37.078 108.190 1.00 0.00

ATOM 4123 HG SER 25 51.315 37.766 107.687 1.00 0.00

ATOM 4124 C SER 25 49.489 35.520 105.128 1.00 0.00

ATOM 4125 O SER 25 49.667 36.055 104.036 1.00 0.00

ATOM 4126 N ALA 26 49.589 34.192 105.220 1.00 0.00

ATOM 4127 H ALA 26 49.622 33.702 106.102 1.00 0.00

ATOM 4128 CA ALA 26 49.441 33.334 104.062 1.00 0.00

ATOM 4129 HA ALA 26 50.403 33.461 103.573 1.00 0.00

ATOM 4130 CB ALA 26 49.155 31.922 104.567 1.00 0.00

ATOM 4131 HB1 ALA 26 49.054 31.225 103.735 1.00 0.00

ATOM 4132 HB2 ALA 26 50.032 31.480 105.040 1.00 0.00

ATOM 4133 HB3 ALA 26 48.289 31.899 105.228 1.00 0.00

ATOM 4134 C ALA 26 48.356 33.785 103.093 1.00 0.00

ATOM 4135 O ALA 26 48.563 33.818 101.881 1.00 0.00

ATOM 4136 N ALA 27 47.141 34.063 103.568 1.00 0.00

ATOM 4137 H ALA 27 47.029 33.897 104.558 1.00 0.00

ATOM 4138 CA ALA 27 46.004 34.572 102.827 1.00 0.00

ATOM 4139 HA ALA 27 45.733 33.847 102.059 1.00 0.00

ATOM 4140 CB ALA 27 44.759 34.595 103.709 1.00 0.00

ATOM 4141 HB1 ALA 27 43.931 34.914 103.076 1.00 0.00

ATOM 4142 HB2 ALA 27 44.930 35.271 104.547 1.00 0.00

ATOM 4143 HB3 ALA 27 44.600 33.567 104.035 1.00 0.00

ATOM 4144 C ALA 27 46.249 35.869 102.069 1.00 0.00

ATOM 4145 O ALA 27 45.525 36.085 101.100 1.00 0.00

ATOM 4146 N ARG 28 47.323 36.606 102.355 1.00 0.00

ATOM 4147 H ARG 28 47.910 36.344 103.135 1.00 0.00

ATOM 4148 CA ARG 28 47.672 37.763 101.554 1.00 0.00

ATOM 4149 HA ARG 28 46.780 38.218 101.125 1.00 0.00

ATOM 4150 CB ARG 28 48.380 38.797 102.424 1.00 0.00

ATOM 4151 HB1 ARG 28 48.841 39.521 101.751 1.00 0.00

ATOM 4152 HB2 ARG 28 49.170 38.183 102.856 1.00 0.00

ATOM 4153 CG ARG 28 47.613 39.422 103.587 1.00 0.00

ATOM 4154 HG1 ARG 28 47.218 38.599 104.182 1.00 0.00

ATOM 4155 HG2 ARG 28 48.190 40.145 104.165 1.00 0.00

ATOM 4156 CD ARG 28 46.304 40.056 103.129 1.00 0.00

ATOM 4157 HD1 ARG 28 45.574 39.257 102.998 1.00 0.00

ATOM 4158 HD2 ARG 28 45.953 40.630 103.986 1.00 0.00

ATOM 4159 NE ARG 28 46.516 40.908 101.958 1.00 0.00

ATOM 4160 HE ARG 28 47.443 41.239 101.727 1.00 0.00

ATOM 4161 CZ ARG 28 45.522 41.338 101.171 1.00 0.00

ATOM 4162 NH1 ARG 28 44.235 41.069 101.422 1.00 0.00

ATOM 4163 1HH1 ARG 28 44.060 40.422 102.179 1.00 0.00

ATOM 4164 2HH1 ARG 28 43.629 41.839 101.181 1.00 0.00

ATOM 4165 NH2 ARG 28 45.797 42.246 100.227 1.00 0.00

ATOM 4166 1HH2 ARG 28 46.758 42.406 99.956 1.00 0.00

ATOM 4167 2HH2 ARG 28 45.083 42.533 99.573 1.00 0.00

ATOM 4168 C ARG 28 48.598 37.423 100.395 1.00 0.00

ATOM 4169 O ARG 28 48.974 38.341 99.669 1.00 0.00

ATOM 4170 N VAL 29 48.934 36.153 100.158 1.00 0.00

ATOM 4171 H VAL 29 48.587 35.421 100.760 1.00 0.00

ATOM 4172 CA VAL 29 49.823 35.722 99.097 1.00 0.00

ATOM 4173 HA VAL 29 50.044 36.556 98.431 1.00 0.00

ATOM 4174 CB VAL 29 51.156 35.473 99.794 1.00 0.00

ATOM 4175 HB VAL 29 51.037 34.549 100.361 1.00 0.00

ATOM 4176 CG1 VAL 29 52.259 35.306 98.752 1.00 0.00

ATOM 4177 1HG1 VAL 29 53.178 35.046 99.276 1.00 0.00

ATOM 4178 2HG1 VAL 29 52.356 36.154 98.073 1.00 0.00

ATOM 4179 3HG1 VAL 29 52.011 34.427 98.159 1.00 0.00

ATOM 4180 CG2 VAL 29 51.505 36.550 100.817 1.00 0.00

ATOM 4181 1HG2 VAL 29 50.766 36.554 101.618 1.00 0.00

ATOM 4182 2HG2 VAL 29 51.573 37.536 100.357 1.00 0.00

ATOM 4183 3HG2 VAL 29 52.482 36.348 101.255 1.00 0.00

ATOM 4184 C VAL 29 49.473 34.451 98.335 1.00 0.00

ATOM 4185 O VAL 29 49.471 34.498 97.107 1.00 0.00

ATOM 4186 N LYS 30 49.130 33.345 98.998 1.00 0.00

ATOM 4187 H LYS 30 49.001 33.370 100.000 1.00 0.00

ATOM 4188 CA LYS 30 49.293 32.034 98.403 1.00 0.00

ATOM 4189 HA LYS 30 50.268 31.910 97.932 1.00 0.00

ATOM 4190 CB LYS 30 49.087 30.943 99.450 1.00 0.00

ATOM 4191 HB1 LYS 30 49.118 30.043 98.837 1.00 0.00

ATOM 4192 HB2 LYS 30 48.168 31.153 99.998 1.00 0.00

ATOM 4193 CG LYS 30 50.204 30.844 100.484 1.00 0.00

ATOM 4194 HG1 LYS 30 51.145 30.698 99.955 1.00 0.00

ATOM 4195 HG2 LYS 30 50.355 31.812 100.963 1.00 0.00

ATOM 4196 CD LYS 30 49.968 29.616 101.361 1.00 0.00

ATOM 4197 HD1 LYS 30 48.999 29.574 101.857 1.00 0.00

ATOM 4198 HD2 LYS 30 49.939 28.748 100.703 1.00 0.00

ATOM 4199 CE LYS 30 51.110 29.557 102.371 1.00 0.00

ATOM 4200 HE1 LYS 30 51.996 29.774 101.775 1.00 0.00

ATOM 4201 HE2 LYS 30 50.926 30.260 103.184 1.00 0.00

ATOM 4202 NZ LYS 30 51.309 28.241 102.998 1.00 0.00

ATOM 4203 HZ1 LYS 30 50.452 27.991 103.472 1.00 0.00

ATOM 4204 HZ2 LYS 30 52.081 28.218 103.648 1.00 0.00

ATOM 4205 HZ3 LYS 30 51.556 27.503 102.355 1.00 0.00

ATOM 4206 C LYS 30 48.432 31.850 97.162 1.00 0.00

ATOM 4207 O LYS 30 48.975 31.725 96.067 1.00 0.00

ATOM 4208 N ALA 31 47.109 31.898 97.339 1.00 0.00

ATOM 4209 H ALA 31 46.791 32.023 98.288 1.00 0.00

ATOM 4210 CA ALA 31 46.114 31.957 96.287 1.00 0.00

ATOM 4211 HA ALA 31 46.310 31.139 95.593 1.00 0.00

ATOM 4212 CB ALA 31 44.685 31.783 96.790 1.00 0.00

ATOM 4213 HB1 ALA 31 44.605 30.855 97.347 1.00 0.00

ATOM 4214 HB2 ALA 31 44.490 32.587 97.500 1.00 0.00

ATOM 4215 HB3 ALA 31 44.071 31.638 95.900 1.00 0.00

ATOM 4216 C ALA 31 46.232 33.211 95.433 1.00 0.00

ATOM 4217 O ALA 31 46.185 33.073 94.213 1.00 0.00

ATOM 4218 N TYR 32 46.483 34.395 95.994 1.00 0.00

ATOM 4219 H TYR 32 46.377 34.400 96.998 1.00 0.00

ATOM 4220 CA TYR 32 46.579 35.601 95.194 1.00 0.00

ATOM 4221 HA TYR 32 45.624 35.713 94.682 1.00 0.00

ATOM 4222 CB TYR 32 46.640 36.778 96.162 1.00 0.00

ATOM 4223 HB1 TYR 32 47.211 36.448 97.030 1.00 0.00

ATOM 4224 HB2 TYR 32 45.626 37.069 96.436 1.00 0.00

ATOM 4225 CG TYR 32 47.232 38.047 95.596 1.00 0.00

ATOM 4226 CD1 TYR 32 48.609 38.261 95.740 1.00 0.00

ATOM 4227 HD1 TYR 32 49.262 37.528 96.189 1.00 0.00

ATOM 4228 CE1 TYR 32 49.203 39.418 95.221 1.00 0.00

ATOM 4229 HE1 TYR 32 50.252 39.595 95.407 1.00 0.00

ATOM 4230 CZ TYR 32 48.435 40.398 94.561 1.00 0.00

ATOM 4231 OH TYR 32 48.989 41.555 94.099 1.00 0.00

ATOM 4232 HH TYR 32 49.904 41.563 94.392 1.00 0.00

ATOM 4233 CE2 TYR 32 47.061 40.139 94.392 1.00 0.00

ATOM 4234 HE2 TYR 32 46.576 40.823 93.712 1.00 0.00

ATOM 4235 CD2 TYR 32 46.448 39.012 94.954 1.00 0.00

ATOM 4236 HD2 TYR 32 45.379 38.862 94.913 1.00 0.00

ATOM 4237 C TYR 32 47.676 35.694 94.142 1.00 0.00

ATOM 4238 O TYR 32 47.361 35.996 92.993 1.00 0.00

ATOM 4239 N ASN 33 48.897 35.333 94.541 1.00 0.00

ATOM 4240 H ASN 33 49.060 35.165 95.524 1.00 0.00

ATOM 4241 CA ASN 33 49.999 35.208 93.609 1.00 0.00

ATOM 4242 HA ASN 33 49.981 36.099 92.984 1.00 0.00

ATOM 4243 CB ASN 33 51.327 35.276 94.358 1.00 0.00

ATOM 4244 HB1 ASN 33 51.487 36.161 94.975 1.00 0.00

ATOM 4245 HB2 ASN 33 51.279 34.419 95.028 1.00 0.00

ATOM 4246 CG ASN 33 52.593 35.021 93.551 1.00 0.00

ATOM 4247 OD1 ASN 33 53.424 35.872 93.243 1.00 0.00

ATOM 4248 ND2 ASN 33 52.748 33.748 93.181 1.00 0.00

ATOM 4249 1HD2 ASN 33 52.107 33.105 93.622 1.00 0.00

ATOM 4250 2HD2 ASN 33 53.543 33.379 92.679 1.00 0.00

ATOM 4251 C ASN 33 49.770 34.040 92.660 1.00 0.00

ATOM 4252 O ASN 33 50.052 34.221 91.477 1.00 0.00

ATOM 4253 N SER 34 49.041 33.000 93.075 1.00 0.00

ATOM 4254 H SER 34 48.798 32.814 94.037 1.00 0.00

ATOM 4255 CA SER 34 48.667 31.959 92.140 1.00 0.00

ATOM 4256 HA SER 34 49.579 31.651 91.628 1.00 0.00

ATOM 4257 CB SER 34 48.173 30.674 92.797 1.00 0.00

ATOM 4258 HB1 SER 34 47.806 29.991 92.030 1.00 0.00

ATOM 4259 HB2 SER 34 47.331 30.907 93.449 1.00 0.00

ATOM 4260 OG SER 34 49.253 30.031 93.432 1.00 0.00

ATOM 4261 HG SER 34 48.828 29.552 94.146 1.00 0.00

ATOM 4262 C SER 34 47.658 32.499 91.136 1.00 0.00

ATOM 4263 O SER 34 47.789 32.195 89.952 1.00 0.00

ATOM 4264 N ALA 35 46.662 33.280 91.560 1.00 0.00

ATOM 4265 H ALA 35 46.516 33.517 92.531 1.00 0.00

ATOM 4266 CA ALA 35 45.733 33.889 90.629 1.00 0.00

ATOM 4267 HA ALA 35 45.145 33.136 90.106 1.00 0.00

ATOM 4268 CB ALA 35 44.691 34.688 91.407 1.00 0.00

ATOM 4269 HB1 ALA 35 44.066 35.148 90.642 1.00 0.00

ATOM 4270 HB2 ALA 35 44.135 34.056 92.100 1.00 0.00

ATOM 4271 HB3 ALA 35 45.098 35.417 92.108 1.00 0.00

ATOM 4272 C ALA 35 46.420 34.698 89.538 1.00 0.00

ATOM 4273 O ALA 35 46.158 34.572 88.344 1.00 0.00

ATOM 4274 N ALA 36 47.474 35.433 89.897 1.00 0.00

ATOM 4275 H ALA 36 47.689 35.432 90.884 1.00 0.00

ATOM 4276 CA ALA 36 48.278 36.151 88.929 1.00 0.00

ATOM 4277 HA ALA 36 47.635 36.639 88.195 1.00 0.00

ATOM 4278 CB ALA 36 49.058 37.198 89.720 1.00 0.00

ATOM 4279 HB1 ALA 36 49.619 37.861 89.062 1.00 0.00

ATOM 4280 HB2 ALA 36 49.651 36.911 90.587 1.00 0.00

ATOM 4281 HB3 ALA 36 48.268 37.824 90.136 1.00 0.00

ATOM 4282 C ALA 36 49.203 35.277 88.095 1.00 0.00

ATOM 4283 O ALA 36 49.334 35.543 86.902 1.00 0.00

ATOM 4284 N SER 37 49.847 34.230 88.614 1.00 0.00

ATOM 4285 H SER 37 49.865 34.181 89.623 1.00 0.00

ATOM 4286 CA SER 37 50.646 33.309 87.829 1.00 0.00

ATOM 4287 HA SER 37 51.539 33.770 87.406 1.00 0.00

ATOM 4288 CB SER 37 51.263 32.235 88.718 1.00 0.00

ATOM 4289 HB1 SER 37 50.486 31.549 89.054 1.00 0.00

ATOM 4290 HB2 SER 37 52.003 31.590 88.243 1.00 0.00

ATOM 4291 OG SER 37 51.891 32.942 89.764 1.00 0.00

ATOM 4292 HG SER 37 51.283 33.600 90.110 1.00 0.00

ATOM 4293 C SER 37 49.897 32.556 86.739 1.00 0.00

ATOM 4294 O SER 37 50.448 32.324 85.666 1.00 0.00

ATOM 4295 N SER 38 48.649 32.208 87.063 1.00 0.00

ATOM 4296 H SER 38 48.333 32.564 87.954 1.00 0.00

ATOM 4297 CA SER 38 47.702 31.486 86.238 1.00 0.00

ATOM 4298 HA SER 38 48.123 30.540 85.899 1.00 0.00

ATOM 4299 CB SER 38 46.510 31.150 87.128 1.00 0.00

ATOM 4300 HB1 SER 38 45.707 30.722 86.529 1.00 0.00

ATOM 4301 HB2 SER 38 46.144 32.015 87.681 1.00 0.00

ATOM 4302 OG SER 38 46.824 30.185 88.108 1.00 0.00

ATOM 4303 HG SER 38 47.621 30.471 88.560 1.00 0.00

ATOM 4304 C SER 38 47.175 32.264 85.039 1.00 0.00

ATOM 4305 O SER 38 46.548 31.730 84.128 1.00 0.00

ATOM 4306 N ASP 39 47.538 33.535 84.854 1.00 0.00

ATOM 4307 H ASP 39 48.101 33.950 85.581 1.00 0.00

ATOM 4308 CA ASP 39 47.112 34.419 83.786 1.00 0.00

ATOM 4309 HA ASP 39 46.197 34.176 83.244 1.00 0.00

ATOM 4310 CB ASP 39 46.915 35.861 84.241 1.00 0.00

ATOM 4311 HB1 ASP 39 46.511 35.941 85.250 1.00 0.00

ATOM 4312 HB2 ASP 39 47.893 36.342 84.257 1.00 0.00

ATOM 4313 CG ASP 39 45.980 36.634 83.320 1.00 0.00

ATOM 4314 OD1 ASP 39 44.746 36.558 83.507 1.00 0.00

ATOM 4315 OD2 ASP 39 46.467 37.391 82.454 1.00 0.00

ATOM 4316 C ASP 39 48.254 34.396 82.779 1.00 0.00

ATOM 4317 O ASP 39 47.986 34.118 81.613 1.00 0.00

ATOM 4318 N LEU 40 49.522 34.459 83.191 1.00 0.00

ATOM 4319 H LEU 40 49.703 34.622 84.171 1.00 0.00

ATOM 4320 CA LEU 40 50.666 34.150 82.357 1.00 0.00

ATOM 4321 HA LEU 40 50.693 34.839 81.512 1.00 0.00

ATOM 4322 CB LEU 40 51.966 34.354 83.130 1.00 0.00

ATOM 4323 HB1 LEU 40 52.718 34.059 82.399 1.00 0.00

ATOM 4324 HB2 LEU 40 51.967 33.640 83.954 1.00 0.00

ATOM 4325 CG LEU 40 52.282 35.689 83.798 1.00 0.00

ATOM 4326 HG LEU 40 51.501 36.080 84.450 1.00 0.00

ATOM 4327 CD1 LEU 40 53.574 35.536 84.597 1.00 0.00

ATOM 4328 1HD1 LEU 40 54.352 35.180 83.925 1.00 0.00

ATOM 4329 2HD1 LEU 40 53.528 34.692 85.285 1.00 0.00

ATOM 4330 3HD1 LEU 40 53.890 36.470 85.061 1.00 0.00

ATOM 4331 CD2 LEU 40 52.456 36.784 82.750 1.00 0.00

ATOM 4332 1HD2 LEU 40 53.113 36.530 81.920 1.00 0.00

ATOM 4333 2HD2 LEU 40 52.861 37.594 83.351 1.00 0.00

ATOM 4334 3HD2 LEU 40 51.489 37.001 82.297 1.00 0.00

ATOM 4335 C LEU 40 50.725 32.735 81.800 1.00 0.00

ATOM 4336 O LEU 40 51.281 32.602 80.712 1.00 0.00

ATOM 4337 N ARG 41 50.192 31.727 82.494 1.00 0.00

ATOM 4338 H ARG 41 49.801 32.015 83.379 1.00 0.00

ATOM 4339 CA ARG 41 49.942 30.383 82.011 1.00 0.00

ATOM 4340 HA ARG 41 50.824 30.008 81.492 1.00 0.00

ATOM 4341 CB ARG 41 49.539 29.438 83.140 1.00 0.00

ATOM 4342 HB1 ARG 41 48.556 29.668 83.552 1.00 0.00

ATOM 4343 HB2 ARG 41 50.239 29.576 83.964 1.00 0.00

ATOM 4344 CG ARG 41 49.508 27.973 82.715 1.00 0.00

ATOM 4345 HG1 ARG 41 48.986 27.977 81.759 1.00 0.00

ATOM 4346 HG2 ARG 41 50.489 27.521 82.567 1.00 0.00

ATOM 4347 CD ARG 41 48.800 27.106 83.752 1.00 0.00

ATOM 4348 HD1 ARG 41 49.148 27.381 84.748 1.00 0.00

ATOM 4349 HD2 ARG 41 47.725 27.289 83.762 1.00 0.00

ATOM 4350 NE ARG 41 49.020 25.669 83.585 1.00 0.00

ATOM 4351 HE ARG 41 49.719 25.372 82.920 1.00 0.00

ATOM 4352 CZ ARG 41 48.408 24.751 84.347 1.00 0.00

ATOM 4353 NH1 ARG 41 47.498 25.090 85.268 1.00 0.00

ATOM 4354 1HH1 ARG 41 47.452 26.047 85.589 1.00 0.00

ATOM 4355 2HH1 ARG 41 46.842 24.400 85.608 1.00 0.00

ATOM 4356 NH2 ARG 41 48.525 23.440 84.105 1.00 0.00

ATOM 4357 1HH2 ARG 41 49.243 23.160 83.452 1.00 0.00

ATOM 4358 2HH2 ARG 41 48.105 22.724 84.683 1.00 0.00

ATOM 4359 C ARG 41 48.904 30.450 80.900 1.00 0.00

ATOM 4360 O ARG 41 49.018 29.811 79.857 1.00 0.00

ATOM 4361 N ASN 42 47.863 31.279 81.004 1.00 0.00

ATOM 4362 H ASN 42 47.652 31.527 81.959 1.00 0.00

ATOM 4363 CA ASN 42 46.811 31.362 80.009 1.00 0.00

ATOM 4364 HA ASN 42 46.630 30.322 79.738 1.00 0.00

ATOM 4365 CB ASN 42 45.579 32.067 80.567 1.00 0.00

ATOM 4366 HB1 ASN 42 45.394 31.763 81.598 1.00 0.00

ATOM 4367 HB2 ASN 42 45.850 33.116 80.686 1.00 0.00

ATOM 4368 CG ASN 42 44.408 32.069 79.593 1.00 0.00

ATOM 4369 OD1 ASN 42 43.888 31.048 79.159 1.00 0.00

ATOM 4370 ND2 ASN 42 43.795 33.210 79.272 1.00 0.00

ATOM 4371 1HD2 ASN 42 43.980 34.125 79.656 1.00 0.00

ATOM 4372 2HD2 ASN 42 42.970 33.086 78.703 1.00 0.00

ATOM 4373 C ASN 42 47.220 32.114 78.751 1.00 0.00

ATOM 4374 O ASN 42 46.922 31.717 77.626 1.00 0.00

ATOM 4375 N LEU 43 47.973 33.191 78.987 1.00 0.00

ATOM 4376 H LEU 43 48.133 33.436 79.954 1.00 0.00

ATOM 4377 CA LEU 43 48.601 34.085 78.035 1.00 0.00

ATOM 4378 HA LEU 43 47.840 34.637 77.483 1.00 0.00

ATOM 4379 CB LEU 43 49.362 35.091 78.895 1.00 0.00

ATOM 4380 HB1 LEU 43 50.140 34.627 79.499 1.00 0.00

ATOM 4381 HB2 LEU 43 48.729 35.579 79.638 1.00 0.00

ATOM 4382 CG LEU 43 50.130 36.191 78.167 1.00 0.00

ATOM 4383 HG LEU 43 50.452 36.982 78.843 1.00 0.00

ATOM 4384 CD1 LEU 43 51.439 35.701 77.555 1.00 0.00

ATOM 4385 1HD1 LEU 43 51.047 35.047 76.777 1.00 0.00

ATOM 4386 2HD1 LEU 43 52.001 35.080 78.254 1.00 0.00

ATOM 4387 3HD1 LEU 43 52.017 36.469 77.040 1.00 0.00

ATOM 4388 CD2 LEU 43 49.283 36.944 77.147 1.00 0.00

ATOM 4389 1HD2 LEU 43 49.806 37.821 76.765 1.00 0.00

ATOM 4390 2HD2 LEU 43 48.372 37.257 77.657 1.00 0.00

ATOM 4391 3HD2 LEU 43 49.015 36.249 76.351 1.00 0.00

ATOM 4392 C LEU 43 49.405 33.290 77.016 1.00 0.00

ATOM 4393 O LEU 43 49.015 33.196 75.855 1.00 0.00

ATOM 4394 N LYS 44 50.486 32.590 77.372 1.00 0.00

ATOM 4395 H LYS 44 50.878 32.667 78.298 1.00 0.00

ATOM 4396 CA LYS 44 51.340 31.730 76.578 1.00 0.00

ATOM 4397 HA LYS 44 51.665 32.180 75.640 1.00 0.00

ATOM 4398 CB LYS 44 52.553 31.329 77.414 1.00 0.00

ATOM 4399 HB1 LYS 44 52.333 30.358 77.858 1.00 0.00

ATOM 4400 HB2 LYS 44 52.624 32.096 78.184 1.00 0.00

ATOM 4401 CG LYS 44 53.880 31.261 76.661 1.00 0.00

ATOM 4402 HG1 LYS 44 54.608 30.927 77.399 1.00 0.00

ATOM 4403 HG2 LYS 44 53.906 30.545 75.840 1.00 0.00

ATOM 4404 CD LYS 44 54.400 32.585 76.111 1.00 0.00

ATOM 4405 HD1 LYS 44 53.598 33.024 75.518 1.00 0.00

ATOM 4406 HD2 LYS 44 54.519 33.170 77.023 1.00 0.00

ATOM 4407 CE LYS 44 55.723 32.507 75.354 1.00 0.00

ATOM 4408 HE1 LYS 44 56.498 32.155 76.035 1.00 0.00

ATOM 4409 HE2 LYS 44 55.751 31.800 74.524 1.00 0.00

ATOM 4410 NZ LYS 44 56.111 33.840 74.870 1.00 0.00

ATOM 4411 HZ1 LYS 44 55.285 34.247 74.454 1.00 0.00

ATOM 4412 HZ2 LYS 44 56.835 33.875 74.166 1.00 0.00

ATOM 4413 HZ3 LYS 44 56.470 34.342 75.668 1.00 0.00

ATOM 4414 C LYS 44 50.512 30.553 76.083 1.00 0.00

ATOM 4415 O LYS 44 50.889 30.063 75.022 1.00 0.00

ATOM 4416 N THR 45 49.393 30.145 76.686 1.00 0.00

ATOM 4417 H THR 45 49.130 30.532 77.582 1.00 0.00

ATOM 4418 CA THR 45 48.634 29.010 76.201 1.00 0.00

ATOM 4419 HA THR 45 49.350 28.298 75.802 1.00 0.00

ATOM 4420 CB THR 45 47.952 28.267 77.347 1.00 0.00

ATOM 4421 HB THR 45 47.436 28.946 78.027 1.00 0.00

ATOM 4422 CG2 THR 45 47.010 27.142 76.928 1.00 0.00

ATOM 4423 1HG2 THR 45 46.547 26.730 77.825 1.00 0.00

ATOM 4424 2HG2 THR 45 47.552 26.405 76.335 1.00 0.00

ATOM 4425 3HG2 THR 45 46.275 27.537 76.227 1.00 0.00

ATOM 4426 OG1 THR 45 48.962 27.662 78.122 1.00 0.00

ATOM 4427 HG1 THR 45 49.243 28.292 78.790 1.00 0.00

ATOM 4428 C THR 45 47.732 29.383 75.033 1.00 0.00

ATOM 4429 O THR 45 47.614 28.675 74.036 1.00 0.00

ATOM 4430 N ALA 46 46.998 30.489 75.178 1.00 0.00

ATOM 4431 H ALA 46 47.078 30.907 76.094 1.00 0.00

ATOM 4432 CA ALA 46 45.956 30.939 74.278 1.00 0.00

ATOM 4433 HA ALA 46 45.855 30.259 73.433 1.00 0.00

ATOM 4434 CB ALA 46 44.686 30.867 75.124 1.00 0.00

ATOM 4435 HB1 ALA 46 43.770 30.693 74.562 1.00 0.00

ATOM 4436 HB2 ALA 46 44.514 31.749 75.740 1.00 0.00

ATOM 4437 HB3 ALA 46 44.674 30.040 75.834 1.00 0.00

ATOM 4438 C ALA 46 46.224 32.343 73.755 1.00 0.00

ATOM 4439 O ALA 46 45.977 32.572 72.574 1.00 0.00

ATOM 4440 N LEU 47 46.571 33.329 74.588 1.00 0.00

ATOM 4441 H LEU 47 46.927 33.055 75.492 1.00 0.00

ATOM 4442 CA LEU 47 46.335 34.737 74.335 1.00 0.00

ATOM 4443 HA LEU 47 45.509 34.715 73.625 1.00 0.00

ATOM 4444 CB LEU 47 46.055 35.510 75.622 1.00 0.00

ATOM 4445 HB1 LEU 47 46.829 36.274 75.679 1.00 0.00

ATOM 4446 HB2 LEU 47 46.224 34.956 76.546 1.00 0.00

ATOM 4447 CG LEU 47 44.703 36.218 75.643 1.00 0.00

ATOM 4448 HG LEU 47 44.798 36.924 76.468 1.00 0.00

ATOM 4449 CD1 LEU 47 44.520 37.055 74.381 1.00 0.00

ATOM 4450 1HD1 LEU 47 45.444 37.594 74.177 1.00 0.00

ATOM 4451 2HD1 LEU 47 43.748 37.801 74.564 1.00 0.00

ATOM 4452 3HD1 LEU 47 44.271 36.381 73.560 1.00 0.00

ATOM 4453 CD2 LEU 47 43.491 35.309 75.822 1.00 0.00

ATOM 4454 1HD2 LEU 47 42.572 35.876 75.673 1.00 0.00

ATOM 4455 2HD2 LEU 47 43.337 34.943 76.836 1.00 0.00

ATOM 4456 3HD2 LEU 47 43.579 34.365 75.283 1.00 0.00

ATOM 4457 C LEU 47 47.409 35.356 73.452 1.00 0.00

ATOM 4458 O LEU 47 47.288 36.377 72.779 1.00 0.00

ATOM 4459 N GLU 48 48.529 34.643 73.328 1.00 0.00

ATOM 4460 H GLU 48 48.591 33.902 74.011 1.00 0.00

ATOM 4461 CA GLU 48 49.591 34.944 72.388 1.00 0.00

ATOM 4462 HA GLU 48 49.615 36.006 72.143 1.00 0.00

ATOM 4463 CB GLU 48 50.912 34.737 73.122 1.00 0.00

ATOM 4464 HB1 GLU 48 51.222 33.698 73.010 1.00 0.00

ATOM 4465 HB2 GLU 48 50.719 34.890 74.184 1.00 0.00

ATOM 4466 CG GLU 48 52.146 35.495 72.643 1.00 0.00

ATOM 4467 HG1 GLU 48 52.330 35.322 71.582 1.00 0.00

ATOM 4468 HG2 GLU 48 52.163 36.577 72.774 1.00 0.00

ATOM 4469 CD GLU 48 53.353 34.985 73.418 1.00 0.00

ATOM 4470 OE1 GLU 48 53.893 33.895 73.134 1.00 0.00

ATOM 4471 OE2 GLU 48 53.812 35.708 74.328 1.00 0.00

ATOM 4472 C GLU 48 49.520 34.051 71.156 1.00 0.00

ATOM 4473 O GLU 48 50.181 34.354 70.166 1.00 0.00

ATOM 4474 N SER 49 48.726 32.979 71.130 1.00 0.00

ATOM 4475 H SER 49 48.211 32.923 71.997 1.00 0.00

ATOM 4476 CA SER 49 48.777 31.897 70.167 1.00 0.00

ATOM 4477 HA SER 49 49.830 31.713 69.955 1.00 0.00

ATOM 4478 CB SER 49 48.146 30.711 70.892 1.00 0.00

ATOM 4479 HB1 SER 49 48.361 29.836 70.279 1.00 0.00

ATOM 4480 HB2 SER 49 47.061 30.789 70.961 1.00 0.00

ATOM 4481 OG SER 49 48.690 30.646 72.191 1.00 0.00

ATOM 4482 HG SER 49 48.249 29.950 72.684 1.00 0.00

ATOM 4483 C SER 49 48.174 32.162 68.795 1.00 0.00

ATOM 4484 O SER 49 47.846 31.257 68.030 1.00 0.00

ATOM 4485 N ALA 50 48.154 33.444 68.423 1.00 0.00

ATOM 4486 H ALA 50 48.620 34.113 69.019 1.00 0.00

ATOM 4487 CA ALA 50 47.662 33.945 67.154 1.00 0.00

ATOM 4488 HA ALA 50 46.582 33.808 67.131 1.00 0.00

ATOM 4489 CB ALA 50 47.952 35.429 66.950 1.00 0.00

ATOM 4490 HB1 ALA 50 47.492 35.998 67.758 1.00 0.00

ATOM 4491 HB2 ALA 50 49.021 35.634 66.889 1.00 0.00

ATOM 4492 HB3 ALA 50 47.500 35.823 66.039 1.00 0.00

ATOM 4493 C ALA 50 48.218 33.279 65.904 1.00 0.00

ATOM 4494 O ALA 50 47.525 33.012 64.924 1.00 0.00

ATOM 4495 N PHE 51 49.511 32.948 65.890 1.00 0.00

ATOM 4496 H PHE 51 50.025 33.042 66.753 1.00 0.00

ATOM 4497 CA PHE 51 50.312 32.520 64.761 1.00 0.00

ATOM 4498 HA PHE 51 49.639 32.481 63.903 1.00 0.00

ATOM 4499 CB PHE 51 51.518 33.443 64.613 1.00 0.00

ATOM 4500 HB1 PHE 51 52.004 33.164 63.677 1.00 0.00

ATOM 4501 HB2 PHE 51 52.221 33.291 65.431 1.00 0.00

ATOM 4502 CG PHE 51 51.232 34.924 64.540 1.00 0.00

ATOM 4503 CD1 PHE 51 51.278 35.595 63.312 1.00 0.00

ATOM 4504 HD1 PHE 51 51.276 34.966 62.438 1.00 0.00

ATOM 4505 CE1 PHE 51 51.048 36.972 63.205 1.00 0.00

ATOM 4506 HE1 PHE 51 51.026 37.394 62.211 1.00 0.00

ATOM 4507 CZ PHE 51 51.011 37.728 64.383 1.00 0.00

ATOM 4508 HZ PHE 51 50.805 38.788 64.380 1.00 0.00

ATOM 4509 CE2 PHE 51 51.160 37.097 65.624 1.00 0.00

ATOM 4510 HE2 PHE 51 51.025 37.650 66.537 1.00 0.00

ATOM 4511 CD2 PHE 51 51.225 35.700 65.705 1.00 0.00

ATOM 4512 HD2 PHE 51 51.247 35.210 66.667 1.00 0.00

ATOM 4513 C PHE 51 50.844 31.107 64.957 1.00 0.00

ATOM 4514 O PHE 51 51.544 30.641 64.062 1.00 0.00

ATOM 4515 N ALA 52 50.348 30.277 65.877 1.00 0.00

ATOM 4516 H ALA 52 49.770 30.635 66.624 1.00 0.00

ATOM 4517 CA ALA 52 50.766 28.907 66.095 1.00 0.00

ATOM 4518 HA ALA 52 51.822 29.044 66.325 1.00 0.00

ATOM 4519 CB ALA 52 50.073 28.429 67.368 1.00 0.00

ATOM 4520 HB1 ALA 52 50.337 29.138 68.153 1.00 0.00

ATOM 4521 HB2 ALA 52 48.986 28.406 67.291 1.00 0.00

ATOM 4522 HB3 ALA 52 50.502 27.465 67.643 1.00 0.00

ATOM 4523 C ALA 52 50.568 27.921 64.953 1.00 0.00

ATOM 4524 O ALA 52 51.353 26.989 64.794 1.00 0.00

ATOM 4525 N ASP 53 49.584 28.214 64.099 1.00 0.00

ATOM 4526 H ASP 53 48.986 28.995 64.331 1.00 0.00

ATOM 4527 CA ASP 53 49.254 27.379 62.962 1.00 0.00

ATOM 4528 HA ASP 53 49.359 26.361 63.338 1.00 0.00

ATOM 4529 CB ASP 53 47.863 27.697 62.421 1.00 0.00

ATOM 4530 HB1 ASP 53 47.057 27.639 63.147 1.00 0.00

ATOM 4531 HB2 ASP 53 47.899 28.686 61.964 1.00 0.00

ATOM 4532 CG ASP 53 47.607 26.700 61.300 1.00 0.00

ATOM 4533 OD1 ASP 53 47.261 25.536 61.595 1.00 0.00

ATOM 4534 OD2 ASP 53 47.650 27.143 60.132 1.00 0.00

ATOM 4535 C ASP 53 50.319 27.545 61.888 1.00 0.00

ATOM 4536 O ASP 53 50.606 26.601 61.157 1.00 0.00

ATOM 4537 N ASP 54 50.946 28.718 61.780 1.00 0.00

ATOM 4538 H ASP 54 50.703 29.481 62.396 1.00 0.00

ATOM 4539 CA ASP 54 52.020 28.974 60.841 1.00 0.00

ATOM 4540 HA ASP 54 51.953 28.338 59.959 1.00 0.00

ATOM 4541 CB ASP 54 51.884 30.423 60.383 1.00 0.00

ATOM 4542 HB1 ASP 54 50.818 30.571 60.226 1.00 0.00

ATOM 4543 HB2 ASP 54 52.137 31.076 61.219 1.00 0.00

ATOM 4544 CG ASP 54 52.717 30.793 59.163 1.00 0.00

ATOM 4545 OD1 ASP 54 52.465 30.299 58.043 1.00 0.00

ATOM 4546 OD2 ASP 54 53.735 31.492 59.353 1.00 0.00

ATOM 4547 C ASP 54 53.383 28.698 61.461 1.00 0.00

ATOM 4548 O ASP 54 54.309 28.344 60.740 1.00 0.00

ATOM 4549 N GLN 55 53.636 28.902 62.756 1.00 0.00

ATOM 4550 H GLN 55 52.814 29.225 63.245 1.00 0.00

ATOM 4551 CA GLN 55 54.771 28.560 63.592 1.00 0.00

ATOM 4552 HA GLN 55 55.681 28.831 63.058 1.00 0.00

ATOM 4553 CB GLN 55 54.753 29.485 64.805 1.00 0.00

ATOM 4554 HB1 GLN 55 55.416 29.126 65.593 1.00 0.00

ATOM 4555 HB2 GLN 55 53.715 29.481 65.136 1.00 0.00

ATOM 4556 CG GLN 55 54.948 30.952 64.431 1.00 0.00

ATOM 4557 HG1 GLN 55 55.855 31.067 63.876 1.00 0.00

ATOM 4558 HG2 GLN 55 54.231 31.165 63.638 1.00 0.00

ATOM 4559 CD GLN 55 54.720 31.984 65.526 1.00 0.00

ATOM 4560 OE1 GLN 55 54.343 31.639 66.640 1.00 0.00

ATOM 4561 NE2 GLN 55 54.914 33.279 65.262 1.00 0.00

ATOM 4562 1HE2 GLN 55 55.141 33.631 64.344 1.00 0.00

ATOM 4563 2HE2 GLN 55 54.875 33.943 66.020 1.00 0.00

ATOM 4564 C GLN 55 54.826 27.077 63.934 1.00 0.00

ATOM 4565 O GLN 55 55.606 26.577 64.742 1.00 0.00

ATOM 4566 N THR 56 53.905 26.266 63.414 1.00 0.00

ATOM 4567 H THR 56 53.221 26.873 62.987 1.00 0.00

ATOM 4568 CA THR 56 53.693 24.847 63.630 1.00 0.00

ATOM 4569 HA THR 56 52.805 24.595 63.051 1.00 0.00

ATOM 4570 CB THR 56 54.780 23.893 63.141 1.00 0.00

ATOM 4571 HB THR 56 54.326 22.909 63.261 1.00 0.00

ATOM 4572 CG2 THR 56 55.345 24.076 61.736 1.00 0.00

ATOM 4573 1HG2 THR 56 55.756 25.082 61.652 1.00 0.00

ATOM 4574 2HG2 THR 56 54.552 24.004 60.997 1.00 0.00

ATOM 4575 3HG2 THR 56 56.008 23.236 61.534 1.00 0.00

ATOM 4576 OG1 THR 56 55.859 24.032 64.038 1.00 0.00

ATOM 4577 HG1 THR 56 55.895 24.883 64.484 1.00 0.00

ATOM 4578 C THR 56 53.249 24.428 65.023 1.00 0.00

ATOM 4579 O THR 56 52.722 23.321 65.110 1.00 0.00

ATOM 4580 N TYR 57 53.540 25.164 66.099 1.00 0.00

ATOM 4581 H TYR 57 54.047 26.031 66.000 1.00 0.00

ATOM 4582 CA TYR 57 53.207 24.790 67.458 1.00 0.00

ATOM 4583 HA TYR 57 52.371 24.092 67.429 1.00 0.00

ATOM 4584 CB TYR 57 54.380 24.074 68.126 1.00 0.00

ATOM 4585 HB1 TYR 57 54.676 23.223 67.515 1.00 0.00

ATOM 4586 HB2 TYR 57 54.142 23.600 69.078 1.00 0.00

ATOM 4587 CG TYR 57 55.714 24.777 68.194 1.00 0.00

ATOM 4588 CD1 TYR 57 56.019 25.817 69.079 1.00 0.00

ATOM 4589 HD1 TYR 57 55.283 26.194 69.774 1.00 0.00

ATOM 4590 CE1 TYR 57 57.290 26.403 69.100 1.00 0.00

ATOM 4591 HE1 TYR 57 57.439 27.167 69.849 1.00 0.00

ATOM 4592 CZ TYR 57 58.280 26.002 68.182 1.00 0.00

ATOM 4593 OH TYR 57 59.405 26.754 68.009 1.00 0.00

ATOM 4594 HH TYR 57 60.030 26.424 67.359 1.00 0.00

ATOM 4595 CE2 TYR 57 57.966 24.991 67.251 1.00 0.00

ATOM 4596 HE2 TYR 57 58.746 24.669 66.577 1.00 0.00

ATOM 4597 CD2 TYR 57 56.725 24.348 67.325 1.00 0.00

ATOM 4598 HD2 TYR 57 56.568 23.382 66.869 1.00 0.00

ATOM 4599 C TYR 57 52.830 26.033 68.251 1.00 0.00

ATOM 4600 O TYR 57 53.220 27.101 67.786 1.00 0.00

ATOM 4601 N PRO 58 52.098 25.881 69.358 1.00 0.00

ATOM 4602 CD PRO 58 51.593 24.684 70.000 1.00 0.00

ATOM 4603 HD1 PRO 58 52.444 24.133 70.399 1.00 0.00

ATOM 4604 HD2 PRO 58 51.050 24.041 69.308 1.00 0.00

ATOM 4605 CG PRO 58 50.749 25.086 71.206 1.00 0.00

ATOM 4606 HG1 PRO 58 51.450 25.009 72.038 1.00 0.00

ATOM 4607 HG2 PRO 58 49.845 24.480 71.259 1.00 0.00

ATOM 4608 CB PRO 58 50.420 26.544 70.897 1.00 0.00

ATOM 4609 HB1 PRO 58 50.068 27.192 71.699 1.00 0.00

ATOM 4610 HB2 PRO 58 49.619 26.493 70.161 1.00 0.00

ATOM 4611 CA PRO 58 51.702 26.993 70.200 1.00 0.00

ATOM 4612 HA PRO 58 51.510 27.850 69.555 1.00 0.00

ATOM 4613 C PRO 58 52.827 27.431 71.126 1.00 0.00

ATOM 4614 O PRO 58 53.729 26.635 71.373 1.00 0.00

ATOM 4615 N PRO 59 52.868 28.666 71.633 1.00 0.00

ATOM 4616 CD PRO 59 52.071 29.802 71.216 1.00 0.00

ATOM 4617 HD1 PRO 59 51.089 29.766 71.687 1.00 0.00

ATOM 4618 HD2 PRO 59 51.945 29.890 70.137 1.00 0.00

ATOM 4619 CG PRO 59 52.783 31.063 71.696 1.00 0.00

ATOM 4620 HG1 PRO 59 52.044 31.848 71.858 1.00 0.00

ATOM 4621 HG2 PRO 59 53.545 31.331 70.963 1.00 0.00

ATOM 4622 CB PRO 59 53.613 30.595 72.887 1.00 0.00

ATOM 4623 HB1 PRO 59 53.018 30.772 73.783 1.00 0.00

ATOM 4624 HB2 PRO 59 54.518 31.199 72.946 1.00 0.00

ATOM 4625 CA PRO 59 53.871 29.123 72.573 1.00 0.00

ATOM 4626 HA PRO 59 54.791 29.063 71.993 1.00 0.00

ATOM 4627 C PRO 59 54.031 28.225 73.792 1.00 0.00

ATOM 4628 O PRO 59 55.093 27.632 73.968 1.00 0.00

ATOM 4629 N GLU 60 53.011 28.093 74.642 1.00 0.00

ATOM 4630 H GLU 60 52.173 28.583 74.363 1.00 0.00

ATOM 4631 CA GLU 60 52.958 27.345 75.883 1.00 0.00

ATOM 4632 HA GLU 60 51.926 27.433 76.221 1.00 0.00

ATOM 4633 CB GLU 60 53.291 25.893 75.552 1.00 0.00

ATOM 4634 HB1 GLU 60 54.350 25.687 75.703 1.00 0.00

ATOM 4635 HB2 GLU 60 53.160 25.535 74.530 1.00 0.00

ATOM 4636 CG GLU 60 52.478 25.089 76.559 1.00 0.00

ATOM 4637 HG1 GLU 60 52.655 25.416 77.570 1.00 0.00

ATOM 4638 HG2 GLU 60 51.435 25.276 76.304 1.00 0.00

ATOM 4639 CD GLU 60 52.664 23.591 76.366 1.00 0.00

ATOM 4640 OE1 GLU 60 53.626 23.018 76.923 1.00 0.00

ATOM 4641 OE2 GLU 60 51.901 22.993 75.577 1.00 0.00

ATOM 4642 C GLU 60 53.769 27.858 77.066 1.00 0.00

ATOM 4643 O GLU 60 53.150 27.941 78.123 1.00 0.00

ATOM 4644 N SER 61 55.067 28.159 76.975 1.00 0.00

ATOM 4645 H SER 61 55.468 28.101 76.051 1.00 0.00

ATOM 4646 CA SER 61 56.087 28.379 77.981 1.00 0.00

ATOM 4647 HA SER 61 55.547 28.702 78.870 1.00 0.00

ATOM 4648 CB SER 61 56.758 27.064 78.367 1.00 0.00

ATOM 4649 HB1 SER 61 57.176 26.570 77.489 1.00 0.00

ATOM 4650 HB2 SER 61 57.513 27.169 79.145 1.00 0.00

ATOM 4651 OG SER 61 55.885 26.180 79.034 1.00 0.00

ATOM 4652 HG SER 61 55.984 25.297 78.671 1.00 0.00

ATOM 4653 C SER 61 57.056 29.516 77.682 1.00 0.00

ATOM 4654 OC1 SER 61 57.306 29.801 76.492 1.00 0.00

ATOM 4655 OC2 SER 61 57.586 30.114 78.639 1.00 0.00

ATOM 4656 N PHE 1 33.224 34.014 144.307 1.00 0.00

ATOM 4657 H1 PHE 1 33.371 33.329 145.033 1.00 0.00

ATOM 4658 H2 PHE 1 34.085 34.520 144.155 1.00 0.00

ATOM 4659 H3 PHE 1 33.082 33.365 143.548 1.00 0.00

ATOM 4660 CA PHE 1 32.045 34.862 144.552 1.00 0.00

ATOM 4661 HA PHE 1 31.881 35.475 143.666 1.00 0.00

ATOM 4662 CB PHE 1 30.750 34.110 144.842 1.00 0.00

ATOM 4663 HB1 PHE 1 29.857 34.730 144.761 1.00 0.00

ATOM 4664 HB2 PHE 1 30.793 33.646 145.829 1.00 0.00

ATOM 4665 CG PHE 1 30.556 33.113 143.726 1.00 0.00

ATOM 4666 CD1 PHE 1 30.396 33.549 142.405 1.00 0.00

ATOM 4667 HD1 PHE 1 30.251 34.599 142.197 1.00 0.00

ATOM 4668 CE1 PHE 1 30.172 32.636 141.368 1.00 0.00

ATOM 4669 HE1 PHE 1 30.072 33.018 140.363 1.00 0.00

ATOM 4670 CZ PHE 1 30.135 31.256 141.600 1.00 0.00

ATOM 4671 HZ PHE 1 30.211 30.467 140.866 1.00 0.00

ATOM 4672 CE2 PHE 1 30.211 30.850 142.937 1.00 0.00

ATOM 4673 HE2 PHE 1 30.263 29.801 143.187 1.00 0.00

ATOM 4674 CD2 PHE 1 30.527 31.735 143.975 1.00 0.00

ATOM 4675 HD2 PHE 1 30.693 31.345 144.968 1.00 0.00

ATOM 4676 C PHE 1 32.366 35.832 145.681 1.00 0.00

ATOM 4677 O PHE 1 31.926 35.700 146.819 1.00 0.00

ATOM 4678 N THR 2 33.275 36.774 145.417 1.00 0.00

ATOM 4679 H THR 2 33.499 36.833 144.435 1.00 0.00

ATOM 4680 CA THR 2 33.833 37.793 146.283 1.00 0.00

ATOM 4681 HA THR 2 33.235 37.778 147.194 1.00 0.00

ATOM 4682 CB THR 2 35.303 37.621 146.658 1.00 0.00

ATOM 4683 HB THR 2 35.569 38.421 147.349 1.00 0.00

ATOM 4684 CG2 THR 2 35.564 36.264 147.305 1.00 0.00

ATOM 4685 1HG2 THR 2 36.580 36.341 147.689 1.00 0.00

ATOM 4686 2HG2 THR 2 35.601 35.417 146.620 1.00 0.00

ATOM 4687 3HG2 THR 2 34.976 36.120 148.212 1.00 0.00

ATOM 4688 OG1 THR 2 36.065 37.708 145.475 1.00 0.00

ATOM 4689 HG1 THR 2 35.756 37.011 144.892 1.00 0.00

ATOM 4690 C THR 2 33.595 39.189 145.726 1.00 0.00

ATOM 4691 O THR 2 33.436 39.358 144.519 1.00 0.00

ATOM 4692 N LEU 3 33.598 40.132 146.671 1.00 0.00

ATOM 4693 H LEU 3 33.573 39.832 147.635 1.00 0.00

ATOM 4694 CA LEU 3 33.156 41.486 146.405 1.00 0.00

ATOM 4695 HA LEU 3 32.198 41.405 145.892 1.00 0.00

ATOM 4696 CB LEU 3 32.929 42.245 147.710 1.00 0.00

ATOM 4697 HB1 LEU 3 33.842 42.263 148.302 1.00 0.00

ATOM 4698 HB2 LEU 3 32.202 41.814 148.399 1.00 0.00

ATOM 4699 CG LEU 3 32.538 43.681 147.375 1.00 0.00

ATOM 4700 HG LEU 3 33.262 44.263 146.805 1.00 0.00

ATOM 4701 CD1 LEU 3 31.204 43.741 146.636 1.00 0.00

ATOM 4702 1HD1 LEU 3 31.070 44.821 146.614 1.00 0.00

ATOM 4703 2HD1 LEU 3 30.400 43.279 147.209 1.00 0.00

ATOM 4704 3HD1 LEU 3 31.333 43.463 145.589 1.00 0.00

ATOM 4705 CD2 LEU 3 32.322 44.330 148.739 1.00 0.00

ATOM 4706 1HD2 LEU 3 31.534 43.787 149.261 1.00 0.00

ATOM 4707 2HD2 LEU 3 32.088 45.391 148.644 1.00 0.00

ATOM 4708 3HD2 LEU 3 33.173 44.210 149.409 1.00 0.00

ATOM 4709 C LEU 3 34.051 42.163 145.376 1.00 0.00

ATOM 4710 O LEU 3 33.518 42.844 144.503 1.00 0.00

ATOM 4711 N ILE 4 35.350 41.858 145.323 1.00 0.00

ATOM 4712 H ILE 4 35.692 41.306 146.096 1.00 0.00

ATOM 4713 CA ILE 4 36.311 42.212 144.298 1.00 0.00

ATOM 4714 HA ILE 4 36.257 43.300 144.317 1.00 0.00

ATOM 4715 CB ILE 4 37.711 42.003 144.867 1.00 0.00

ATOM 4716 HB ILE 4 37.764 42.582 145.790 1.00 0.00

ATOM 4717 CG2 ILE 4 37.974 40.513 145.070 1.00 0.00

ATOM 4718 1HG2 ILE 4 38.791 40.440 145.787 1.00 0.00

ATOM 4719 2HG2 ILE 4 38.298 39.976 144.177 1.00 0.00

ATOM 4720 3HG2 ILE 4 37.109 40.073 145.564 1.00 0.00

ATOM 4721 CG1 ILE 4 38.800 42.583 143.971 1.00 0.00

ATOM 4722 1HG1 ILE 4 39.755 42.356 144.445 1.00 0.00

ATOM 4723 2HG1 ILE 4 38.804 42.069 143.008 1.00 0.00

ATOM 4724 CD ILE 4 38.634 44.098 143.912 1.00 0.00

ATOM 4725 HD1 ILE 4 38.467 44.602 144.865 1.00 0.00

ATOM 4726 HD2 ILE 4 37.768 44.264 143.272 1.00 0.00

ATOM 4727 HD3 ILE 4 39.527 44.431 143.385 1.00 0.00

ATOM 4728 C ILE 4 36.102 41.692 142.882 1.00 0.00

ATOM 4729 O ILE 4 36.424 42.363 141.904 1.00 0.00

ATOM 4730 N GLU 5 35.567 40.472 142.800 1.00 0.00

ATOM 4731 H GLU 5 35.360 39.928 143.625 1.00 0.00

ATOM 4732 CA GLU 5 35.269 39.894 141.505 1.00 0.00

ATOM 4733 HA GLU 5 36.099 40.092 140.826 1.00 0.00

ATOM 4734 CB GLU 5 35.067 38.391 141.667 1.00 0.00

ATOM 4735 HB1 GLU 5 34.648 37.976 140.751 1.00 0.00

ATOM 4736 HB2 GLU 5 34.355 38.286 142.486 1.00 0.00

ATOM 4737 CG GLU 5 36.310 37.561 141.974 1.00 0.00

ATOM 4738 HG1 GLU 5 36.998 37.429 141.140 1.00 0.00

ATOM 4739 HG2 GLU 5 36.902 38.074 142.732 1.00 0.00

ATOM 4740 CD GLU 5 35.933 36.168 142.455 1.00 0.00

ATOM 4741 OE1 GLU 5 35.285 35.987 143.510 1.00 0.00

ATOM 4742 OE2 GLU 5 36.043 35.220 141.650 1.00 0.00

ATOM 4743 C GLU 5 34.004 40.522 140.938 1.00 0.00

ATOM 4744 O GLU 5 34.193 40.920 139.791 1.00 0.00

ATOM 4745 N LEU 6 32.959 40.837 141.707 1.00 0.00

ATOM 4746 H LEU 6 33.072 40.518 142.658 1.00 0.00

ATOM 4747 CA LEU 6 31.845 41.671 141.296 1.00 0.00

ATOM 4748 HA LEU 6 31.408 41.327 140.359 1.00 0.00

ATOM 4749 CB LEU 6 30.866 41.606 142.464 1.00 0.00

ATOM 4750 HB1 LEU 6 31.436 41.376 143.364 1.00 0.00

ATOM 4751 HB2 LEU 6 30.234 40.721 142.392 1.00 0.00

ATOM 4752 CG LEU 6 29.897 42.780 142.591 1.00 0.00

ATOM 4753 HG LEU 6 30.383 43.723 142.837 1.00 0.00

ATOM 4754 CD1 LEU 6 28.963 42.883 141.388 1.00 0.00

ATOM 4755 1HD1 LEU 6 28.316 42.007 141.437 1.00 0.00

ATOM 4756 2HD1 LEU 6 29.493 42.849 140.436 1.00 0.00

ATOM 4757 3HD1 LEU 6 28.274 43.728 141.407 1.00 0.00

ATOM 4758 CD2 LEU 6 29.013 42.614 143.823 1.00 0.00

ATOM 4759 1HD2 LEU 6 28.207 43.348 143.783 1.00 0.00

ATOM 4760 2HD2 LEU 6 29.557 42.592 144.767 1.00 0.00

ATOM 4761 3HD2 LEU 6 28.524 41.648 143.700 1.00 0.00

ATOM 4762 C LEU 6 32.181 43.112 140.938 1.00 0.00

ATOM 4763 O LEU 6 31.667 43.769 140.035 1.00 0.00

ATOM 4764 N LEU 7 33.315 43.541 141.494 1.00 0.00

ATOM 4765 H LEU 7 33.782 42.980 142.192 1.00 0.00

ATOM 4766 CA LEU 7 33.838 44.878 141.291 1.00 0.00

ATOM 4767 HA LEU 7 33.015 45.591 141.320 1.00 0.00

ATOM 4768 CB LEU 7 34.689 45.234 142.506 1.00 0.00

ATOM 4769 HB1 LEU 7 35.447 44.464 142.653 1.00 0.00

ATOM 4770 HB2 LEU 7 33.919 45.205 143.276 1.00 0.00

ATOM 4771 CG LEU 7 35.274 46.644 142.503 1.00 0.00

ATOM 4772 HG LEU 7 36.018 46.735 141.712 1.00 0.00

ATOM 4773 CD1 LEU 7 34.226 47.744 142.367 1.00 0.00

ATOM 4774 1HD1 LEU 7 33.600 47.627 141.483 1.00 0.00

ATOM 4775 2HD1 LEU 7 34.789 48.665 142.346 1.00 0.00

ATOM 4776 3HD1 LEU 7 33.642 47.869 143.280 1.00 0.00

ATOM 4777 CD2 LEU 7 35.920 46.755 143.881 1.00 0.00

ATOM 4778 1HD2 LEU 7 36.686 45.986 143.982 1.00 0.00

ATOM 4779 2HD2 LEU 7 35.115 46.675 144.610 1.00 0.00

ATOM 4780 3HD2 LEU 7 36.385 47.725 144.058 1.00 0.00

ATOM 4781 C LEU 7 34.560 45.141 139.978 1.00 0.00

ATOM 4782 O LEU 7 34.429 46.247 139.458 1.00 0.00

ATOM 4783 N ILE 8 35.146 44.094 139.391 1.00 0.00

ATOM 4784 H ILE 8 35.248 43.240 139.919 1.00 0.00

ATOM 4785 CA ILE 8 36.029 44.248 138.253 1.00 0.00

ATOM 4786 HA ILE 8 35.990 45.292 137.941 1.00 0.00

ATOM 4787 CB ILE 8 37.434 43.870 138.711 1.00 0.00

ATOM 4788 HB ILE 8 37.426 42.798 138.912 1.00 0.00

ATOM 4789 CG2 ILE 8 38.361 43.927 137.502 1.00 0.00

ATOM 4790 1HG2 ILE 8 37.971 43.302 136.699 1.00 0.00

ATOM 4791 2HG2 ILE 8 39.367 43.573 137.728 1.00 0.00

ATOM 4792 3HG2 ILE 8 38.358 44.976 137.204 1.00 0.00

ATOM 4793 CG1 ILE 8 37.962 44.805 139.796 1.00 0.00

ATOM 4794 1HG1 ILE 8 37.375 44.717 140.709 1.00 0.00

ATOM 4795 2HG1 ILE 8 38.896 44.309 140.057 1.00 0.00

ATOM 4796 CD ILE 8 38.160 46.307 139.601 1.00 0.00

ATOM 4797 HD1 ILE 8 37.254 46.909 139.521 1.00 0.00

ATOM 4798 HD2 ILE 8 38.721 46.429 138.675 1.00 0.00

ATOM 4799 HD3 ILE 8 38.717 46.690 140.456 1.00 0.00

ATOM 4800 C ILE 8 35.469 43.584 137.003 1.00 0.00

ATOM 4801 O ILE 8 35.851 44.042 135.928 1.00 0.00

ATOM 4802 N VAL 9 34.397 42.800 137.141 1.00 0.00

ATOM 4803 H VAL 9 34.051 42.626 138.076 1.00 0.00

ATOM 4804 CA VAL 9 33.711 42.173 136.030 1.00 0.00

ATOM 4805 HA VAL 9 34.492 41.590 135.543 1.00 0.00

ATOM 4806 CB VAL 9 32.613 41.202 136.456 1.00 0.00

ATOM 4807 HB VAL 9 33.133 40.521 137.129 1.00 0.00

ATOM 4808 CG1 VAL 9 31.632 42.049 137.260 1.00 0.00

ATOM 4809 1HG1 VAL 9 31.196 41.385 138.007 1.00 0.00

ATOM 4810 2HG1 VAL 9 30.944 42.563 136.589 1.00 0.00

ATOM 4811 3HG1 VAL 9 32.073 42.825 137.884 1.00 0.00

ATOM 4812 CG2 VAL 9 31.920 40.337 135.406 1.00 0.00

ATOM 4813 1HG2 VAL 9 31.065 40.852 134.969 1.00 0.00

ATOM 4814 2HG2 VAL 9 31.511 39.412 135.814 1.00 0.00

ATOM 4815 3HG2 VAL 9 32.701 39.974 134.738 1.00 0.00

ATOM 4816 C VAL 9 33.290 43.167 134.957 1.00 0.00

ATOM 4817 O VAL 9 33.529 42.870 133.789 1.00 0.00

ATOM 4818 N VAL 10 32.700 44.308 135.318 1.00 0.00

ATOM 4819 H VAL 10 32.481 44.492 136.287 1.00 0.00

ATOM 4820 CA VAL 10 32.432 45.407 134.413 1.00 0.00

ATOM 4821 HA VAL 10 32.297 44.886 133.466 1.00 0.00

ATOM 4822 CB VAL 10 31.117 46.106 134.748 1.00 0.00

ATOM 4823 HB VAL 10 31.240 46.846 135.537 1.00 0.00

ATOM 4824 CG1 VAL 10 30.595 46.788 133.485 1.00 0.00

ATOM 4825 1HG1 VAL 10 30.454 46.050 132.697 1.00 0.00

ATOM 4826 2HG1 VAL 10 31.301 47.426 132.953 1.00 0.00

ATOM 4827 3HG1 VAL 10 29.773 47.476 133.685 1.00 0.00

ATOM 4828 CG2 VAL 10 30.050 45.094 135.160 1.00 0.00

ATOM 4829 1HG2 VAL 10 30.027 44.215 134.517 1.00 0.00

ATOM 4830 2HG2 VAL 10 29.027 45.466 135.119 1.00 0.00

ATOM 4831 3HG2 VAL 10 30.176 44.732 136.180 1.00 0.00

ATOM 4832 C VAL 10 33.584 46.385 134.238 1.00 0.00

ATOM 4833 O VAL 10 33.791 47.076 133.242 1.00 0.00

ATOM 4834 N ALA 11 34.423 46.505 135.268 1.00 0.00

ATOM 4835 H ALA 11 34.223 46.128 136.184 1.00 0.00

ATOM 4836 CA ALA 11 35.551 47.407 135.137 1.00 0.00

ATOM 4837 HA ALA 11 35.183 48.400 134.883 1.00 0.00

ATOM 4838 CB ALA 11 36.259 47.580 136.477 1.00 0.00

ATOM 4839 HB1 ALA 11 35.514 47.722 137.259 1.00 0.00

ATOM 4840 HB2 ALA 11 36.713 48.557 136.398 1.00 0.00

ATOM 4841 HB3 ALA 11 37.047 46.841 136.624 1.00 0.00

ATOM 4842 C ALA 11 36.592 47.167 134.053 1.00 0.00

ATOM 4843 O ALA 11 37.300 48.057 133.587 1.00 0.00

ATOM 4844 N ILE 12 36.645 45.922 133.577 1.00 0.00

ATOM 4845 H ILE 12 36.124 45.241 134.109 1.00 0.00

ATOM 4846 CA ILE 12 37.450 45.427 132.476 1.00 0.00

ATOM 4847 HA ILE 12 38.418 45.919 132.550 1.00 0.00

ATOM 4848 CB ILE 12 37.734 43.929 132.403 1.00 0.00

ATOM 4849 HB ILE 12 38.205 43.765 131.434 1.00 0.00

ATOM 4850 CG2 ILE 12 38.684 43.642 133.562 1.00 0.00

ATOM 4851 1HG2 ILE 12 38.146 43.841 134.488 1.00 0.00

ATOM 4852 2HG2 ILE 12 39.606 44.216 133.464 1.00 0.00

ATOM 4853 3HG2 ILE 12 39.034 42.610 133.600 1.00 0.00

ATOM 4854 CG1 ILE 12 36.406 43.187 132.521 1.00 0.00

ATOM 4855 1HG1 ILE 12 36.030 43.211 133.544 1.00 0.00

ATOM 4856 2HG1 ILE 12 35.648 43.621 131.868 1.00 0.00

ATOM 4857 CD ILE 12 36.587 41.729 132.107 1.00 0.00

ATOM 4858 HD1 ILE 12 35.592 41.308 131.971 1.00 0.00

ATOM 4859 HD2 ILE 12 36.998 41.088 132.887 1.00 0.00

ATOM 4860 HD3 ILE 12 37.157 41.637 131.182 1.00 0.00

ATOM 4861 C ILE 12 36.968 45.915 131.118 1.00 0.00

ATOM 4862 O ILE 12 37.663 45.857 130.107 1.00 0.00

ATOM 4863 N ILE 13 35.679 46.239 130.998 1.00 0.00

ATOM 4864 H ILE 13 35.185 46.311 131.876 1.00 0.00

ATOM 4865 CA ILE 13 34.971 46.200 129.734 1.00 0.00

ATOM 4866 HA ILE 13 35.297 45.272 129.266 1.00 0.00

ATOM 4867 CB ILE 13 33.494 46.092 130.101 1.00 0.00

ATOM 4868 HB ILE 13 33.287 46.919 130.778 1.00 0.00

ATOM 4869 CG2 ILE 13 32.480 46.418 129.006 1.00 0.00

ATOM 4870 1HG2 ILE 13 31.436 46.391 129.320 1.00 0.00

ATOM 4871 2HG2 ILE 13 32.463 45.666 128.218 1.00 0.00

ATOM 4872 3HG2 ILE 13 32.648 47.355 128.478 1.00 0.00

ATOM 4873 CG1 ILE 13 33.166 44.794 130.834 1.00 0.00

ATOM 4874 1HG1 ILE 13 33.712 44.762 131.777 1.00 0.00

ATOM 4875 2HG1 ILE 13 32.121 44.784 131.143 1.00 0.00

ATOM 4876 CD ILE 13 33.414 43.508 130.048 1.00 0.00

ATOM 4877 HD1 ILE 13 33.263 43.527 128.968 1.00 0.00

ATOM 4878 HD2 ILE 13 32.898 42.705 130.573 1.00 0.00

ATOM 4879 HD3 ILE 13 34.453 43.181 130.077 1.00 0.00

ATOM 4880 C ILE 13 35.329 47.338 128.789 1.00 0.00

ATOM 4881 O ILE 13 35.459 47.088 127.593 1.00 0.00

ATOM 4882 N GLY 14 35.422 48.560 129.319 1.00 0.00

ATOM 4883 H GLY 14 35.196 48.693 130.295 1.00 0.00

ATOM 4884 CA GLY 14 35.863 49.737 128.598 1.00 0.00

ATOM 4885 HA1 GLY 14 35.072 49.919 127.871 1.00 0.00

ATOM 4886 HA2 GLY 14 35.967 50.571 129.292 1.00 0.00

ATOM 4887 C GLY 14 37.129 49.577 127.769 1.00 0.00

ATOM 4888 O GLY 14 37.086 49.764 126.555 1.00 0.00

ATOM 4889 N ILE 15 38.203 49.107 128.408 1.00 0.00

ATOM 4890 H ILE 15 38.133 48.825 129.375 1.00 0.00

ATOM 4891 CA ILE 15 39.439 48.715 127.761 1.00 0.00

ATOM 4892 HA ILE 15 39.738 49.566 127.150 1.00 0.00

ATOM 4893 CB ILE 15 40.546 48.439 128.775 1.00 0.00

ATOM 4894 HB ILE 15 41.309 48.019 128.120 1.00 0.00

ATOM 4895 CG2 ILE 15 41.006 49.681 129.535 1.00 0.00

ATOM 4896 1HG2 ILE 15 40.149 50.197 129.966 1.00 0.00

ATOM 4897 2HG2 ILE 15 41.453 50.299 128.756 1.00 0.00

ATOM 4898 3HG2 ILE 15 41.690 49.503 130.365 1.00 0.00

ATOM 4899 CG1 ILE 15 40.228 47.305 129.745 1.00 0.00

ATOM 4900 1HG1 ILE 15 39.482 47.751 130.404 1.00 0.00

ATOM 4901 2HG1 ILE 15 39.819 46.442 129.220 1.00 0.00

ATOM 4902 CD ILE 15 41.387 46.812 130.606 1.00 0.00

ATOM 4903 HD1 ILE 15 41.108 46.002 131.280 1.00 0.00

ATOM 4904 HD2 ILE 15 41.652 47.657 131.241 1.00 0.00

ATOM 4905 HD3 ILE 15 42.247 46.516 130.004 1.00 0.00

ATOM 4906 C ILE 15 39.385 47.577 126.751 1.00 0.00

ATOM 4907 O ILE 15 40.238 47.375 125.889 1.00 0.00

ATOM 4908 N LEU 16 38.358 46.741 126.912 1.00 0.00

ATOM 4909 H LEU 16 37.615 46.907 127.576 1.00 0.00

ATOM 4910 CA LEU 16 38.052 45.666 125.990 1.00 0.00

ATOM 4911 HA LEU 16 38.989 45.381 125.510 1.00 0.00

ATOM 4912 CB LEU 16 37.430 44.478 126.718 1.00 0.00

ATOM 4913 HB1 LEU 16 36.840 43.853 126.049 1.00 0.00

ATOM 4914 HB2 LEU 16 36.599 44.793 127.349 1.00 0.00

ATOM 4915 CG LEU 16 38.338 43.675 127.646 1.00 0.00

ATOM 4916 HG LEU 16 38.873 44.343 128.321 1.00 0.00

ATOM 4917 CD1 LEU 16 37.513 42.739 128.524 1.00 0.00

ATOM 4918 1HD1 LEU 16 36.690 43.238 129.037 1.00 0.00

ATOM 4919 2HD1 LEU 16 38.133 42.256 129.278 1.00 0.00

ATOM 4920 3HD1 LEU 16 37.101 41.922 127.932 1.00 0.00

ATOM 4921 CD2 LEU 16 39.303 42.838 126.811 1.00 0.00

ATOM 4922 1HD2 LEU 16 39.595 43.297 125.866 1.00 0.00

ATOM 4923 2HD2 LEU 16 38.941 41.836 126.634 1.00 0.00

ATOM 4924 3HD2 LEU 16 40.175 42.591 127.415 1.00 0.00

ATOM 4925 C LEU 16 37.149 46.112 124.849 1.00 0.00

ATOM 4926 O LEU 16 37.098 45.468 123.803 1.00 0.00

ATOM 4927 N ALA 17 36.507 47.277 124.961 1.00 0.00

ATOM 4928 H ALA 17 36.618 47.829 125.800 1.00 0.00

ATOM 4929 CA ALA 17 35.614 47.716 123.907 1.00 0.00

ATOM 4930 HA ALA 17 35.261 46.885 123.296 1.00 0.00

ATOM 4931 CB ALA 17 34.309 48.322 124.415 1.00 0.00

ATOM 4932 HB1 ALA 17 33.841 47.801 125.250 1.00 0.00

ATOM 4933 HB2 ALA 17 33.665 48.377 123.538 1.00 0.00

ATOM 4934 HB3 ALA 17 34.576 49.301 124.813 1.00 0.00

ATOM 4935 C ALA 17 36.365 48.614 122.933 1.00 0.00

ATOM 4936 O ALA 17 36.239 48.536 121.713 1.00 0.00

ATOM 4937 N ALA 18 37.208 49.534 123.403 1.00 0.00

ATOM 4938 H ALA 18 37.243 49.591 124.411 1.00 0.00

ATOM 4939 CA ALA 18 38.040 50.375 122.566 1.00 0.00

ATOM 4940 HA ALA 18 37.428 51.005 121.920 1.00 0.00

ATOM 4941 CB ALA 18 38.988 51.168 123.459 1.00 0.00

ATOM 4942 HB1 ALA 18 38.404 51.666 124.133 1.00 0.00

ATOM 4943 HB2 ALA 18 39.671 50.515 124.001 1.00 0.00

ATOM 4944 HB3 ALA 18 39.617 51.776 122.808 1.00 0.00

ATOM 4945 C ALA 18 38.894 49.639 121.543 1.00 0.00

ATOM 4946 O ALA 18 39.111 50.087 120.418 1.00 0.00

ATOM 4947 N ILE 19 39.336 48.428 121.887 1.00 0.00

ATOM 4948 H ILE 19 39.215 48.028 122.807 1.00 0.00

ATOM 4949 CA ILE 19 40.212 47.693 120.998 1.00 0.00

ATOM 4950 HA ILE 19 40.718 48.401 120.343 1.00 0.00

ATOM 4951 CB ILE 19 41.272 46.906 121.763 1.00 0.00

ATOM 4952 HB ILE 19 41.739 46.198 121.079 1.00 0.00

ATOM 4953 CG2 ILE 19 42.233 47.829 122.507 1.00 0.00

ATOM 4954 1HG2 ILE 19 42.796 48.512 121.870 1.00 0.00

ATOM 4955 2HG2 ILE 19 42.983 47.305 123.099 1.00 0.00

ATOM 4956 3HG2 ILE 19 41.591 48.428 123.153 1.00 0.00

ATOM 4957 CG1 ILE 19 40.546 46.138 122.865 1.00 0.00

ATOM 4958 1HG1 ILE 19 40.074 46.868 123.522 1.00 0.00

ATOM 4959 2HG1 ILE 19 39.747 45.523 122.453 1.00 0.00

ATOM 4960 CD ILE 19 41.341 45.208 123.778 1.00 0.00

ATOM 4961 HD1 ILE 19 41.880 44.536 123.111 1.00 0.00

ATOM 4962 HD2 ILE 19 40.689 44.583 124.389 1.00 0.00

ATOM 4963 HD3 ILE 19 41.991 45.866 124.354 1.00 0.00

ATOM 4964 C ILE 19 39.418 46.853 120.008 1.00 0.00

ATOM 4965 O ILE 19 39.973 46.461 118.984 1.00 0.00

ATOM 4966 N ALA 20 38.115 46.682 120.234 1.00 0.00

ATOM 4967 H ALA 20 37.802 47.068 121.114 1.00 0.00

ATOM 4968 CA ALA 20 37.229 45.917 119.379 1.00 0.00

ATOM 4969 HA ALA 20 37.816 45.266 118.731 1.00 0.00

ATOM 4970 CB ALA 20 36.420 45.159 120.426 1.00 0.00

ATOM 4971 HB1 ALA 20 35.719 44.521 119.888 1.00 0.00

ATOM 4972 HB2 ALA 20 37.118 44.591 121.041 1.00 0.00

ATOM 4973 HB3 ALA 20 35.766 45.822 120.994 1.00 0.00

ATOM 4974 C ALA 20 36.312 46.769 118.511 1.00 0.00

ATOM 4975 O ALA 20 36.002 46.344 117.401 1.00 0.00

ATOM 4976 N ILE 21 35.822 47.944 118.913 1.00 0.00

ATOM 4977 H ILE 21 36.003 48.244 119.860 1.00 0.00

ATOM 4978 CA ILE 21 34.946 48.804 118.142 1.00 0.00

ATOM 4979 HA ILE 21 34.058 48.239 117.861 1.00 0.00

ATOM 4980 CB ILE 21 34.399 49.856 119.104 1.00 0.00

ATOM 4981 HB ILE 21 35.155 50.261 119.775 1.00 0.00

ATOM 4982 CG2 ILE 21 33.878 50.992 118.229 1.00 0.00

ATOM 4983 1HG2 ILE 21 33.545 51.810 118.867 1.00 0.00

ATOM 4984 2HG2 ILE 21 33.009 50.604 117.697 1.00 0.00

ATOM 4985 3HG2 ILE 21 34.607 51.449 117.559 1.00 0.00

ATOM 4986 CG1 ILE 21 33.365 49.301 120.081 1.00 0.00

ATOM 4987 1HG1 ILE 21 32.592 48.817 119.484 1.00 0.00

ATOM 4988 2HG1 ILE 21 33.725 48.507 120.736 1.00 0.00

ATOM 4989 CD ILE 21 32.736 50.378 120.960 1.00 0.00

ATOM 4990 HD1 ILE 21 32.145 49.869 121.721 1.00 0.00

ATOM 4991 HD2 ILE 21 32.039 51.038 120.444 1.00 0.00

ATOM 4992 HD3 ILE 21 33.525 50.933 121.468 1.00 0.00

ATOM 4993 C ILE 21 35.534 49.211 116.799 1.00 0.00

ATOM 4994 O ILE 21 34.746 49.002 115.880 1.00 0.00

ATOM 4995 N PRO 22 36.773 49.691 116.662 1.00 0.00

ATOM 4996 CD PRO 22 37.578 50.167 117.768 1.00 0.00

ATOM 4997 HD1 PRO 22 38.038 49.289 118.221 1.00 0.00

ATOM 4998 HD2 PRO 22 36.931 50.708 118.459 1.00 0.00

ATOM 4999 CG PRO 22 38.654 51.037 117.125 1.00 0.00

ATOM 5000 HG1 PRO 22 39.603 51.149 117.651 1.00 0.00

ATOM 5001 HG2 PRO 22 38.202 52.024 117.030 1.00 0.00

ATOM 5002 CB PRO 22 38.759 50.411 115.737 1.00 0.00

ATOM 5003 HB1 PRO 22 39.423 49.553 115.629 1.00 0.00

ATOM 5004 HB2 PRO 22 39.124 51.228 115.114 1.00 0.00

ATOM 5005 CA PRO 22 37.330 50.024 115.366 1.00 0.00

ATOM 5006 HA PRO 22 36.801 50.887 114.964 1.00 0.00

ATOM 5007 C PRO 22 37.222 48.970 114.274 1.00 0.00

ATOM 5008 O PRO 22 36.601 49.175 113.233 1.00 0.00

ATOM 5009 N GLN 23 37.534 47.731 114.661 1.00 0.00

ATOM 5010 H GLN 23 37.962 47.503 115.546 1.00 0.00

ATOM 5011 CA GLN 23 37.507 46.597 113.758 1.00 0.00

ATOM 5012 HA GLN 23 37.935 46.894 112.802 1.00 0.00

ATOM 5013 CB GLN 23 38.334 45.423 114.276 1.00 0.00

ATOM 5014 HB1 GLN 23 37.874 45.026 115.180 1.00 0.00

ATOM 5015 HB2 GLN 23 39.271 45.786 114.698 1.00 0.00

ATOM 5016 CG GLN 23 38.654 44.344 113.245 1.00 0.00

ATOM 5017 HG1 GLN 23 37.680 44.140 112.801 1.00 0.00

ATOM 5018 HG2 GLN 23 39.256 44.834 112.480 1.00 0.00

ATOM 5019 CD GLN 23 39.300 43.078 113.798 1.00 0.00

ATOM 5020 OE1 GLN 23 39.610 42.862 114.966 1.00 0.00

ATOM 5021 NE2 GLN 23 39.518 42.039 112.996 1.00 0.00

ATOM 5022 1HE2 GLN 23 38.811 41.873 112.292 1.00 0.00

ATOM 5023 2HE2 GLN 23 39.934 41.170 113.303 1.00 0.00

ATOM 5024 C GLN 23 36.078 46.161 113.467 1.00 0.00

ATOM 5025 O GLN 23 35.710 46.012 112.304 1.00 0.00

ATOM 5026 N PHE 24 35.254 46.019 114.506 1.00 0.00

ATOM 5027 H PHE 24 35.594 46.299 115.415 1.00 0.00

ATOM 5028 CA PHE 24 33.825 45.776 114.519 1.00 0.00

ATOM 5029 HA PHE 24 33.701 44.766 114.130 1.00 0.00

ATOM 5030 CB PHE 24 33.306 45.706 115.953 1.00 0.00

ATOM 5031 HB1 PHE 24 33.367 46.667 116.463 1.00 0.00

ATOM 5032 HB2 PHE 24 33.986 45.002 116.433 1.00 0.00

ATOM 5033 CG PHE 24 31.891 45.188 116.049 1.00 0.00

ATOM 5034 CD1 PHE 24 31.661 43.816 115.891 1.00 0.00

ATOM 5035 HD1 PHE 24 32.396 43.154 115.457 1.00 0.00

ATOM 5036 CE1 PHE 24 30.412 43.279 116.223 1.00 0.00

ATOM 5037 HE1 PHE 24 30.209 42.242 116.017 1.00 0.00

ATOM 5038 CZ PHE 24 29.376 44.155 116.569 1.00 0.00

ATOM 5039 HZ PHE 24 28.368 43.773 116.631 1.00 0.00

ATOM 5040 CE2 PHE 24 29.582 45.540 116.602 1.00 0.00

ATOM 5041 HE2 PHE 24 28.813 46.248 116.871 1.00 0.00

ATOM 5042 CD2 PHE 24 30.883 46.047 116.502 1.00 0.00

ATOM 5043 HD2 PHE 24 31.098 47.093 116.656 1.00 0.00

ATOM 5044 C PHE 24 32.993 46.667 113.606 1.00 0.00

ATOM 5045 O PHE 24 32.386 46.281 112.609 1.00 0.00

ATOM 5046 N SER 25 33.123 47.977 113.820 1.00 0.00

ATOM 5047 H SER 25 33.564 48.235 114.691 1.00 0.00

ATOM 5048 CA SER 25 32.614 48.970 112.895 1.00 0.00

ATOM 5049 HA SER 25 31.528 48.891 112.833 1.00 0.00

ATOM 5050 CB SER 25 32.894 50.412 113.307 1.00 0.00

ATOM 5051 HB1 SER 25 32.341 51.101 112.668 1.00 0.00

ATOM 5052 HB2 SER 25 33.956 50.622 113.182 1.00 0.00

ATOM 5053 OG SER 25 32.572 50.579 114.669 1.00 0.00

ATOM 5054 HG SER 25 33.311 50.195 115.146 1.00 0.00

ATOM 5055 C SER 25 33.125 48.780 111.474 1.00 0.00

ATOM 5056 O SER 25 32.344 48.799 110.526 1.00 0.00

ATOM 5057 N ALA 26 34.426 48.562 111.268 1.00 0.00

ATOM 5058 H ALA 26 35.041 48.425 112.058 1.00 0.00

ATOM 5059 CA ALA 26 34.966 48.436 109.929 1.00 0.00

ATOM 5060 HA ALA 26 34.608 49.237 109.282 1.00 0.00

ATOM 5061 CB ALA 26 36.484 48.554 110.037 1.00 0.00

ATOM 5062 HB1 ALA 26 36.830 48.454 109.009 1.00 0.00

ATOM 5063 HB2 ALA 26 36.767 49.543 110.399 1.00 0.00

ATOM 5064 HB3 ALA 26 36.904 47.757 110.651 1.00 0.00

ATOM 5065 C ALA 26 34.434 47.204 109.212 1.00 0.00

ATOM 5066 O ALA 26 34.253 47.222 107.996 1.00 0.00

ATOM 5067 N ALA 27 33.979 46.202 109.968 1.00 0.00

ATOM 5068 H ALA 27 34.243 46.239 110.942 1.00 0.00

ATOM 5069 CA ALA 27 33.331 44.981 109.535 1.00 0.00

ATOM 5070 HA ALA 27 33.798 44.726 108.584 1.00 0.00

ATOM 5071 CB ALA 27 33.663 43.902 110.563 1.00 0.00

ATOM 5072 HB1 ALA 27 33.369 42.917 110.202 1.00 0.00

ATOM 5073 HB2 ALA 27 33.085 44.012 111.480 1.00 0.00

ATOM 5074 HB3 ALA 27 34.737 43.975 110.738 1.00 0.00

ATOM 5075 C ALA 27 31.852 45.195 109.249 1.00 0.00

ATOM 5076 O ALA 27 31.342 44.535 108.346 1.00 0.00

ATOM 5077 N ARG 28 31.124 46.057 109.962 1.00 0.00

ATOM 5078 H ARG 28 31.481 46.341 110.863 1.00 0.00

ATOM 5079 CA ARG 28 29.736 46.345 109.654 1.00 0.00

ATOM 5080 HA ARG 28 29.201 45.396 109.625 1.00 0.00

ATOM 5081 CB ARG 28 29.064 47.162 110.754 1.00 0.00

ATOM 5082 HB1 ARG 28 29.361 48.207 110.665 1.00 0.00

ATOM 5083 HB2 ARG 28 29.407 46.773 111.712 1.00 0.00

ATOM 5084 CG ARG 28 27.545 47.048 110.831 1.00 0.00

ATOM 5085 HG1 ARG 28 27.234 47.692 111.653 1.00 0.00

ATOM 5086 HG2 ARG 28 27.169 47.551 109.940 1.00 0.00

ATOM 5087 CD ARG 28 26.840 45.696 110.915 1.00 0.00

ATOM 5088 HD1 ARG 28 25.774 45.925 110.919 1.00 0.00

ATOM 5089 HD2 ARG 28 26.823 45.049 110.038 1.00 0.00

ATOM 5090 NE ARG 28 27.261 44.863 112.042 1.00 0.00

ATOM 5091 HE ARG 28 28.214 44.901 112.375 1.00 0.00

ATOM 5092 CZ ARG 28 26.498 43.925 112.618 1.00 0.00

ATOM 5093 NH1 ARG 28 25.207 43.724 112.322 1.00 0.00

ATOM 5094 1HH1 ARG 28 24.761 44.322 111.641 1.00 0.00

ATOM 5095 2HH1 ARG 28 24.692 43.105 112.929 1.00 0.00

ATOM 5096 NH2 ARG 28 26.999 43.278 113.676 1.00 0.00

ATOM 5097 1HH2 ARG 28 28.002 43.268 113.799 1.00 0.00

ATOM 5098 2HH2 ARG 28 26.460 42.546 114.121 1.00 0.00

ATOM 5099 C ARG 28 29.550 47.086 108.338 1.00 0.00

ATOM 5100 O ARG 28 28.801 46.705 107.442 1.00 0.00

ATOM 5101 N VAL 29 30.435 48.067 108.147 1.00 0.00

ATOM 5102 H VAL 29 31.117 48.202 108.879 1.00 0.00

ATOM 5103 CA VAL 29 30.717 48.727 106.889 1.00 0.00

ATOM 5104 HA VAL 29 29.833 49.309 106.629 1.00 0.00

ATOM 5105 CB VAL 29 31.804 49.790 107.023 1.00 0.00

ATOM 5106 HB VAL 29 32.698 49.256 107.346 1.00 0.00

ATOM 5107 CG1 VAL 29 31.953 50.514 105.688 1.00 0.00

ATOM 5108 1HG1 VAL 29 32.271 49.830 104.901 1.00 0.00

ATOM 5109 2HG1 VAL 29 32.648 51.353 105.738 1.00 0.00

ATOM 5110 3HG1 VAL 29 30.981 50.891 105.369 1.00 0.00

ATOM 5111 CG2 VAL 29 31.479 50.793 108.127 1.00 0.00

ATOM 5112 1HG2 VAL 29 30.603 51.370 107.833 1.00 0.00

ATOM 5113 2HG2 VAL 29 32.285 51.526 108.110 1.00 0.00

ATOM 5114 3HG2 VAL 29 31.404 50.366 109.127 1.00 0.00

ATOM 5115 C VAL 29 30.924 47.701 105.784 1.00 0.00

ATOM 5116 O VAL 29 30.217 47.640 104.780 1.00 0.00

ATOM 5117 N LYS 30 31.907 46.821 105.983 1.00 0.00

ATOM 5118 H LYS 30 32.542 46.914 106.763 1.00 0.00

ATOM 5119 CA LYS 30 32.278 45.824 104.999 1.00 0.00

ATOM 5120 HA LYS 30 32.582 46.283 104.058 1.00 0.00

ATOM 5121 CB LYS 30 33.544 45.140 105.506 1.00 0.00

ATOM 5122 HB1 LYS 30 33.409 44.676 106.483 1.00 0.00

ATOM 5123 HB2 LYS 30 34.286 45.929 105.621 1.00 0.00

ATOM 5124 CG LYS 30 34.029 44.093 104.506 1.00 0.00

ATOM 5125 HG1 LYS 30 33.382 43.216 104.509 1.00 0.00

ATOM 5126 HG2 LYS 30 34.164 44.462 103.489 1.00 0.00

ATOM 5127 CD LYS 30 35.402 43.496 104.796 1.00 0.00

ATOM 5128 HD1 LYS 30 35.418 43.021 105.777 1.00 0.00

ATOM 5129 HD2 LYS 30 35.558 42.705 104.063 1.00 0.00

ATOM 5130 CE LYS 30 36.578 44.455 104.637 1.00 0.00

ATOM 5131 HE1 LYS 30 36.536 44.992 103.689 1.00 0.00

ATOM 5132 HE2 LYS 30 36.640 45.243 105.388 1.00 0.00

ATOM 5133 NZ LYS 30 37.864 43.759 104.803 1.00 0.00

ATOM 5134 HZ1 LYS 30 37.932 42.944 104.212 1.00 0.00

ATOM 5135 HZ2 LYS 30 38.034 43.490 105.762 1.00 0.00

ATOM 5136 HZ3 LYS 30 38.616 44.374 104.526 1.00 0.00

ATOM 5137 C LYS 30 31.105 44.880 104.774 1.00 0.00

ATOM 5138 O LYS 30 31.057 44.346 103.669 1.00 0.00

ATOM 5139 N ALA 31 30.153 44.799 105.707 1.00 0.00

ATOM 5140 H ALA 31 30.320 45.198 106.620 1.00 0.00

ATOM 5141 CA ALA 31 28.938 44.019 105.586 1.00 0.00

ATOM 5142 HA ALA 31 29.082 43.248 104.829 1.00 0.00

ATOM 5143 CB ALA 31 28.626 43.287 106.888 1.00 0.00

ATOM 5144 HB1 ALA 31 27.703 42.707 106.917 1.00 0.00

ATOM 5145 HB2 ALA 31 29.506 42.697 107.143 1.00 0.00

ATOM 5146 HB3 ALA 31 28.553 44.003 107.706 1.00 0.00

ATOM 5147 C ALA 31 27.724 44.820 105.136 1.00 0.00

ATOM 5148 O ALA 31 26.672 44.211 104.960 1.00 0.00

ATOM 5149 N TYR 32 27.724 46.142 104.949 1.00 0.00

ATOM 5150 H TYR 32 28.562 46.689 105.083 1.00 0.00

ATOM 5151 CA TYR 32 26.590 46.990 104.642 1.00 0.00

ATOM 5152 HA TYR 32 25.665 46.420 104.695 1.00 0.00

ATOM 5153 CB TYR 32 26.417 47.997 105.776 1.00 0.00

ATOM 5154 HB1 TYR 32 27.268 48.677 105.816 1.00 0.00

ATOM 5155 HB2 TYR 32 26.509 47.479 106.729 1.00 0.00

ATOM 5156 CG TYR 32 25.170 48.845 105.865 1.00 0.00

ATOM 5157 CD1 TYR 32 24.052 48.384 106.572 1.00 0.00

ATOM 5158 HD1 TYR 32 24.057 47.449 107.106 1.00 0.00

ATOM 5159 CE1 TYR 32 22.840 49.085 106.590 1.00 0.00

ATOM 5160 HE1 TYR 32 22.004 48.668 107.126 1.00 0.00

ATOM 5161 CZ TYR 32 22.689 50.274 105.849 1.00 0.00

ATOM 5162 OH TYR 32 21.531 50.991 105.885 1.00 0.00

ATOM 5163 HH TYR 32 20.897 50.588 106.483 1.00 0.00

ATOM 5164 CE2 TYR 32 23.834 50.779 105.202 1.00 0.00

ATOM 5165 HE2 TYR 32 23.746 51.754 104.746 1.00 0.00

ATOM 5166 CD2 TYR 32 25.037 50.063 105.188 1.00 0.00

ATOM 5167 HD2 TYR 32 25.834 50.425 104.556 1.00 0.00

ATOM 5168 C TYR 32 26.749 47.685 103.296 1.00 0.00

ATOM 5169 O TYR 32 25.908 47.479 102.427 1.00 0.00

ATOM 5170 N ASN 33 27.837 48.423 103.065 1.00 0.00

ATOM 5171 H ASN 33 28.480 48.562 103.831 1.00 0.00

ATOM 5172 CA ASN 33 28.173 49.123 101.841 1.00 0.00

ATOM 5173 HA ASN 33 27.437 48.814 101.100 1.00 0.00

ATOM 5174 CB ASN 33 28.041 50.626 102.074 1.00 0.00

ATOM 5175 HB1 ASN 33 26.982 50.826 102.238 1.00 0.00

ATOM 5176 HB2 ASN 33 28.635 50.935 102.935 1.00 0.00

ATOM 5177 CG ASN 33 28.470 51.453 100.870 1.00 0.00

ATOM 5178 OD1 ASN 33 28.047 51.189 99.746 1.00 0.00

ATOM 5179 ND2 ASN 33 29.350 52.430 101.091 1.00 0.00

ATOM 5180 1HD2 ASN 33 29.837 52.513 101.973 1.00 0.00

ATOM 5181 2HD2 ASN 33 29.545 53.062 100.328 1.00 0.00

ATOM 5182 C ASN 33 29.545 48.643 101.387 1.00 0.00

ATOM 5183 O ASN 33 30.555 49.220 101.779 1.00 0.00

ATOM 5184 N SER 34 29.721 47.648 100.513 1.00 0.00

ATOM 5185 H SER 34 28.923 47.111 100.211 1.00 0.00

ATOM 5186 CA SER 34 30.862 47.426 99.647 1.00 0.00

ATOM 5187 HA SER 34 31.159 48.342 99.135 1.00 0.00

ATOM 5188 CB SER 34 32.012 46.752 100.391 1.00 0.00

ATOM 5189 HB1 SER 34 32.730 46.452 99.628 1.00 0.00

ATOM 5190 HB2 SER 34 31.755 45.858 100.959 1.00 0.00

ATOM 5191 OG SER 34 32.667 47.696 101.208 1.00 0.00

ATOM 5192 HG SER 34 32.011 48.340 101.482 1.00 0.00

ATOM 5193 C SER 34 30.512 46.610 98.410 1.00 0.00

ATOM 5194 O SER 34 31.034 46.817 97.316 1.00 0.00

ATOM 5195 N ALA 35 29.749 45.526 98.515 1.00 0.00

ATOM 5196 H ALA 35 29.143 45.383 99.316 1.00 0.00

ATOM 5197 CA ALA 35 29.557 44.541 97.471 1.00 0.00

ATOM 5198 HA ALA 35 30.494 44.036 97.238 1.00 0.00

ATOM 5199 CB ALA 35 28.446 43.565 97.850 1.00 0.00

ATOM 5200 HB1 ALA 35 28.704 43.077 98.790 1.00 0.00

ATOM 5201 HB2 ALA 35 27.545 44.168 97.962 1.00 0.00

ATOM 5202 HB3 ALA 35 28.332 42.809 97.073 1.00 0.00

ATOM 5203 C ALA 35 29.161 45.154 96.135 1.00 0.00

ATOM 5204 O ALA 35 29.700 44.707 95.126 1.00 0.00

ATOM 5205 N ALA 36 28.346 46.208 96.048 1.00 0.00

ATOM 5206 H ALA 36 27.860 46.568 96.857 1.00 0.00

ATOM 5207 CA ALA 36 28.053 46.955 94.842 1.00 0.00

ATOM 5208 HA ALA 36 27.902 46.180 94.092 1.00 0.00

ATOM 5209 CB ALA 36 26.710 47.611 95.154 1.00 0.00

ATOM 5210 HB1 ALA 36 26.022 47.112 95.839 1.00 0.00

ATOM 5211 HB2 ALA 36 26.165 47.812 94.233 1.00 0.00

ATOM 5212 HB3 ALA 36 26.919 48.565 95.633 1.00 0.00

ATOM 5213 C ALA 36 29.153 47.858 94.302 1.00 0.00

ATOM 5214 O ALA 36 29.442 47.802 93.109 1.00 0.00

ATOM 5215 N SER 37 29.942 48.496 95.168 1.00 0.00

ATOM 5216 H SER 37 29.716 48.408 96.149 1.00 0.00

ATOM 5217 CA SER 37 31.130 49.224 94.765 1.00 0.00

ATOM 5218 HA SER 37 30.996 50.065 94.086 1.00 0.00

ATOM 5219 CB SER 37 31.740 50.011 95.921 1.00 0.00

ATOM 5220 HB1 SER 37 32.290 49.394 96.631 1.00 0.00

ATOM 5221 HB2 SER 37 32.505 50.682 95.530 1.00 0.00

ATOM 5222 OG SER 37 30.783 50.824 96.562 1.00 0.00

ATOM 5223 HG SER 37 31.177 51.211 97.347 1.00 0.00

ATOM 5224 C SER 37 32.174 48.294 94.164 1.00 0.00

ATOM 5225 O SER 37 32.937 48.655 93.270 1.00 0.00

ATOM 5226 N SER 38 32.277 47.052 94.642 1.00 0.00

ATOM 5227 H SER 38 31.624 46.643 95.296 1.00 0.00

ATOM 5228 CA SER 38 33.211 46.084 94.103 1.00 0.00

ATOM 5229 HA SER 38 34.226 46.475 94.146 1.00 0.00

ATOM 5230 CB SER 38 33.185 44.817 94.953 1.00 0.00

ATOM 5231 HB1 SER 38 33.919 44.202 94.433 1.00 0.00

ATOM 5232 HB2 SER 38 32.193 44.376 95.060 1.00 0.00

ATOM 5233 OG SER 38 33.777 45.082 96.204 1.00 0.00

ATOM 5234 HG SER 38 33.814 44.262 96.702 1.00 0.00

ATOM 5235 C SER 38 32.909 45.732 92.654 1.00 0.00

ATOM 5236 O SER 38 33.791 45.829 91.804 1.00 0.00

ATOM 5237 N ASP 39 31.643 45.521 92.292 1.00 0.00

ATOM 5238 H ASP 39 30.954 45.739 92.998 1.00 0.00

ATOM 5239 CA ASP 39 31.289 45.333 90.898 1.00 0.00

ATOM 5240 HA ASP 39 31.814 44.459 90.515 1.00 0.00

ATOM 5241 CB ASP 39 29.815 44.943 90.835 1.00 0.00

ATOM 5242 HB1 ASP 39 29.494 44.195 91.561 1.00 0.00

ATOM 5243 HB2 ASP 39 29.263 45.820 91.172 1.00 0.00

ATOM 5244 CG ASP 39 29.403 44.482 89.444 1.00 0.00

ATOM 5245 OD1 ASP 39 29.723 43.311 89.143 1.00 0.00

ATOM 5246 OD2 ASP 39 28.698 45.198 88.702 1.00 0.00

ATOM 5247 C ASP 39 31.573 46.562 90.046 1.00 0.00

ATOM 5248 O ASP 39 32.038 46.438 88.915 1.00 0.00

ATOM 5249 N LEU 40 31.304 47.750 90.594 1.00 0.00

ATOM 5250 H LEU 40 30.803 47.721 91.470 1.00 0.00

ATOM 5251 CA LEU 40 31.332 49.004 89.868 1.00 0.00

ATOM 5252 HA LEU 40 30.907 48.845 88.878 1.00 0.00

ATOM 5253 CB LEU 40 30.362 50.014 90.479 1.00 0.00

ATOM 5254 HB1 LEU 40 30.490 50.183 91.548 1.00 0.00

ATOM 5255 HB2 LEU 40 29.381 49.568 90.322 1.00 0.00

ATOM 5256 CG LEU 40 30.311 51.427 89.905 1.00 0.00

ATOM 5257 HG LEU 40 31.342 51.752 90.044 1.00 0.00

ATOM 5258 CD1 LEU 40 30.022 51.324 88.410 1.00 0.00

ATOM 5259 1HD1 LEU 40 30.854 51.143 87.729 1.00 0.00

ATOM 5260 2HD1 LEU 40 29.536 52.235 88.063 1.00 0.00

ATOM 5261 3HD1 LEU 40 29.257 50.573 88.223 1.00 0.00

ATOM 5262 CD2 LEU 40 29.323 52.403 90.538 1.00 0.00

ATOM 5263 1HD2 LEU 40 29.489 52.353 91.613 1.00 0.00

ATOM 5264 2HD2 LEU 40 28.270 52.143 90.422 1.00 0.00

ATOM 5265 3HD2 LEU 40 29.534 53.416 90.199 1.00 0.00

ATOM 5266 C LEU 40 32.746 49.505 89.611 1.00 0.00

ATOM 5267 O LEU 40 33.116 50.020 88.558 1.00 0.00

ATOM 5268 N ARG 41 33.678 49.148 90.498 1.00 0.00

ATOM 5269 H ARG 41 33.463 48.633 91.340 1.00 0.00

ATOM 5270 CA ARG 41 35.092 49.432 90.353 1.00 0.00

ATOM 5271 HA ARG 41 35.222 50.499 90.176 1.00 0.00

ATOM 5272 CB ARG 41 35.897 49.064 91.597 1.00 0.00

ATOM 5273 HB1 ARG 41 35.852 48.017 91.870 1.00 0.00

ATOM 5274 HB2 ARG 41 35.446 49.574 92.445 1.00 0.00

ATOM 5275 CG ARG 41 37.375 49.447 91.577 1.00 0.00

ATOM 5276 HG1 ARG 41 37.854 49.009 90.702 1.00 0.00

ATOM 5277 HG2 ARG 41 37.618 50.506 91.488 1.00 0.00

ATOM 5278 CD ARG 41 38.137 48.962 92.806 1.00 0.00

ATOM 5279 HD1 ARG 41 37.858 49.523 93.690 1.00 0.00

ATOM 5280 HD2 ARG 41 37.942 47.896 92.925 1.00 0.00

ATOM 5281 NE ARG 41 39.568 49.265 92.730 1.00 0.00

ATOM 5282 HE ARG 41 39.917 49.539 91.824 1.00 0.00

ATOM 5283 CZ ARG 41 40.620 48.916 93.483 1.00 0.00

ATOM 5284 NH1 ARG 41 40.500 48.488 94.746 1.00 0.00

ATOM 5285 1HH1 ARG 41 39.611 48.498 95.226 1.00 0.00

ATOM 5286 2HH1 ARG 41 41.294 48.044 95.183 1.00 0.00

ATOM 5287 NH2 ARG 41 41.860 48.951 92.984 1.00 0.00

ATOM 5288 1HH2 ARG 41 41.939 49.225 92.015 1.00 0.00

ATOM 5289 2HH2 ARG 41 42.736 48.860 93.486 1.00 0.00

ATOM 5290 C ARG 41 35.679 48.712 89.147 1.00 0.00

ATOM 5291 O ARG 41 36.531 49.306 88.489 1.00 0.00

ATOM 5292 N ASN 42 35.226 47.534 88.712 1.00 0.00

ATOM 5293 H ASN 42 34.651 46.924 89.274 1.00 0.00

ATOM 5294 CA ASN 42 35.771 46.966 87.495 1.00 0.00

ATOM 5295 HA ASN 42 36.856 47.073 87.504 1.00 0.00

ATOM 5296 CB ASN 42 35.361 45.501 87.615 1.00 0.00

ATOM 5297 HB1 ASN 42 35.442 45.215 88.664 1.00 0.00

ATOM 5298 HB2 ASN 42 34.327 45.318 87.323 1.00 0.00

ATOM 5299 CG ASN 42 36.225 44.738 86.622 1.00 0.00

ATOM 5300 OD1 ASN 42 37.433 44.955 86.566 1.00 0.00

ATOM 5301 ND2 ASN 42 35.774 43.655 85.983 1.00 0.00

ATOM 5302 1HD2 ASN 42 34.781 43.474 85.952 1.00 0.00

ATOM 5303 2HD2 ASN 42 36.323 43.340 85.196 1.00 0.00

ATOM 5304 C ASN 42 35.262 47.704 86.265 1.00 0.00

ATOM 5305 O ASN 42 35.987 47.791 85.277 1.00 0.00

ATOM 5306 N LEU 43 34.049 48.256 86.339 1.00 0.00

ATOM 5307 H LEU 43 33.552 48.253 87.218 1.00 0.00

ATOM 5308 CA LEU 43 33.512 49.172 85.353 1.00 0.00

ATOM 5309 HA LEU 43 33.562 48.681 84.382 1.00 0.00

ATOM 5310 CB LEU 43 32.010 49.340 85.562 1.00 0.00

ATOM 5311 HB1 LEU 43 31.955 49.949 86.465 1.00 0.00

ATOM 5312 HB2 LEU 43 31.650 48.326 85.735 1.00 0.00

ATOM 5313 CG LEU 43 31.167 50.014 84.483 1.00 0.00

ATOM 5314 HG LEU 43 30.181 50.053 84.947 1.00 0.00

ATOM 5315 CD1 LEU 43 31.568 51.398 83.976 1.00 0.00

ATOM 5316 1HD1 LEU 43 31.708 52.092 84.804 1.00 0.00

ATOM 5317 2HD1 LEU 43 30.819 51.830 83.313 1.00 0.00

ATOM 5318 3HD1 LEU 43 32.516 51.448 83.440 1.00 0.00

ATOM 5319 CD2 LEU 43 31.048 49.105 83.263 1.00 0.00

ATOM 5320 1HD2 LEU 43 30.779 48.135 83.682 1.00 0.00

ATOM 5321 2HD2 LEU 43 31.961 49.013 82.675 1.00 0.00

ATOM 5322 3HD2 LEU 43 30.210 49.455 82.661 1.00 0.00

ATOM 5323 C LEU 43 34.305 50.454 85.146 1.00 0.00

ATOM 5324 O LEU 43 34.641 50.824 84.023 1.00 0.00

ATOM 5325 N LYS 44 34.759 51.043 86.255 1.00 0.00

ATOM 5326 H LYS 44 34.323 50.847 87.145 1.00 0.00

ATOM 5327 CA LYS 44 35.621 52.207 86.236 1.00 0.00

ATOM 5328 HA LYS 44 35.252 52.949 85.528 1.00 0.00

ATOM 5329 CB LYS 44 35.699 52.800 87.639 1.00 0.00

ATOM 5330 HB1 LYS 44 36.513 53.525 87.617 1.00 0.00

ATOM 5331 HB2 LYS 44 35.778 52.002 88.377 1.00 0.00

ATOM 5332 CG LYS 44 34.582 53.780 87.988 1.00 0.00

ATOM 5333 HG1 LYS 44 34.781 54.249 88.951 1.00 0.00

ATOM 5334 HG2 LYS 44 34.693 54.528 87.204 1.00 0.00

ATOM 5335 CD LYS 44 33.131 53.323 88.112 1.00 0.00

ATOM 5336 HD1 LYS 44 32.778 52.810 87.218 1.00 0.00

ATOM 5337 HD2 LYS 44 33.158 52.459 88.774 1.00 0.00

ATOM 5338 CE LYS 44 32.109 54.394 88.486 1.00 0.00

ATOM 5339 HE1 LYS 44 31.077 54.046 88.428 1.00 0.00

ATOM 5340 HE2 LYS 44 32.200 54.751 89.512 1.00 0.00

ATOM 5341 NZ LYS 44 32.207 55.602 87.653 1.00 0.00

ATOM 5342 HZ1 LYS 44 32.407 55.363 86.692 1.00 0.00

ATOM 5343 HZ2 LYS 44 32.929 56.254 87.925 1.00 0.00

ATOM 5344 HZ3 LYS 44 31.346 56.118 87.766 1.00 0.00

ATOM 5345 C LYS 44 37.005 51.814 85.737 1.00 0.00

ATOM 5346 O LYS 44 37.543 52.459 84.840 1.00 0.00

ATOM 5347 N THR 45 37.582 50.744 86.287 1.00 0.00

ATOM 5348 H THR 45 37.059 50.259 87.003 1.00 0.00

ATOM 5349 CA THR 45 38.923 50.260 86.028 1.00 0.00

ATOM 5350 HA THR 45 39.547 51.148 86.129 1.00 0.00

ATOM 5351 CB THR 45 39.416 49.249 87.060 1.00 0.00

ATOM 5352 HB THR 45 38.983 48.275 86.832 1.00 0.00

ATOM 5353 CG2 THR 45 40.919 48.997 86.987 1.00 0.00

ATOM 5354 1HG2 THR 45 41.446 49.909 87.268 1.00 0.00

ATOM 5355 2HG2 THR 45 41.265 48.599 86.033 1.00 0.00

ATOM 5356 3HG2 THR 45 41.041 48.207 87.728 1.00 0.00

ATOM 5357 OG1 THR 45 39.174 49.639 88.393 1.00 0.00

ATOM 5358 HG1 THR 45 38.227 49.782 88.454 1.00 0.00

ATOM 5359 C THR 45 39.199 49.788 84.608 1.00 0.00

ATOM 5360 O THR 45 40.212 50.181 84.033 1.00 0.00

ATOM 5361 N ALA 46 38.285 49.073 83.950 1.00 0.00

ATOM 5362 H ALA 46 37.471 48.782 84.473 1.00 0.00

ATOM 5363 CA ALA 46 38.485 48.542 82.616 1.00 0.00

ATOM 5364 HA ALA 46 39.541 48.699 82.396 1.00 0.00

ATOM 5365 CB ALA 46 38.209 47.042 82.580 1.00 0.00

ATOM 5366 HB1 ALA 46 38.908 46.578 81.885 1.00 0.00

ATOM 5367 HB2 ALA 46 37.188 46.812 82.272 1.00 0.00

ATOM 5368 HB3 ALA 46 38.497 46.598 83.533 1.00 0.00

ATOM 5369 C ALA 46 37.682 49.160 81.481 1.00 0.00

ATOM 5370 O ALA 46 38.001 48.867 80.331 1.00 0.00

ATOM 5371 N LEU 47 36.547 49.820 81.725 1.00 0.00

ATOM 5372 H LEU 47 36.220 49.913 82.676 1.00 0.00

ATOM 5373 CA LEU 47 35.706 50.326 80.660 1.00 0.00

ATOM 5374 HA LEU 47 36.099 49.906 79.734 1.00 0.00

ATOM 5375 CB LEU 47 34.283 49.840 80.918 1.00 0.00

ATOM 5376 HB1 LEU 47 33.855 50.263 81.827 1.00 0.00

ATOM 5377 HB2 LEU 47 34.284 48.766 81.104 1.00 0.00

ATOM 5378 CG LEU 47 33.263 50.069 79.805 1.00 0.00

ATOM 5379 HG LEU 47 32.427 49.420 80.066 1.00 0.00

ATOM 5380 CD1 LEU 47 32.642 51.461 79.874 1.00 0.00

ATOM 5381 1HD1 LEU 47 32.255 51.603 80.883 1.00 0.00

ATOM 5382 2HD1 LEU 47 31.878 51.503 79.098 1.00 0.00

ATOM 5383 3HD1 LEU 47 33.432 52.181 79.658 1.00 0.00

ATOM 5384 CD2 LEU 47 33.724 49.826 78.371 1.00 0.00

ATOM 5385 1HD2 LEU 47 32.902 50.122 77.719 1.00 0.00

ATOM 5386 2HD2 LEU 47 33.885 48.770 78.156 1.00 0.00

ATOM 5387 3HD2 LEU 47 34.686 50.302 78.179 1.00 0.00

ATOM 5388 C LEU 47 35.825 51.814 80.363 1.00 0.00

ATOM 5389 O LEU 47 35.937 52.221 79.209 1.00 0.00

ATOM 5390 N GLU 48 35.793 52.661 81.394 1.00 0.00

ATOM 5391 H GLU 48 35.501 52.328 82.302 1.00 0.00

ATOM 5392 CA GLU 48 35.846 54.108 81.302 1.00 0.00

ATOM 5393 HA GLU 48 35.075 54.510 80.647 1.00 0.00

ATOM 5394 CB GLU 48 35.479 54.625 82.691 1.00 0.00

ATOM 5395 HB1 GLU 48 35.687 55.692 82.742 1.00 0.00

ATOM 5396 HB2 GLU 48 36.053 54.125 83.471 1.00 0.00

ATOM 5397 CG GLU 48 33.996 54.559 83.045 1.00 0.00

ATOM 5398 HG1 GLU 48 33.521 54.820 82.100 1.00 0.00

ATOM 5399 HG2 GLU 48 33.681 53.522 83.146 1.00 0.00

ATOM 5400 CD GLU 48 33.509 55.363 84.244 1.00 0.00

ATOM 5401 OE1 GLU 48 32.497 55.044 84.906 1.00 0.00

ATOM 5402 OE2 GLU 48 34.161 56.352 84.628 1.00 0.00

ATOM 5403 C GLU 48 37.149 54.717 80.806 1.00 0.00

ATOM 5404 O GLU 48 37.192 55.629 79.982 1.00 0.00

ATOM 5405 N SER 49 38.196 53.942 81.097 1.00 0.00

ATOM 5406 H SER 49 37.969 53.077 81.567 1.00 0.00

ATOM 5407 CA SER 49 39.558 53.986 80.601 1.00 0.00

ATOM 5408 HA SER 49 39.968 54.989 80.715 1.00 0.00

ATOM 5409 CB SER 49 40.450 53.106 81.472 1.00 0.00

ATOM 5410 HB1 SER 49 40.484 53.585 82.451 1.00 0.00

ATOM 5411 HB2 SER 49 41.504 53.102 81.197 1.00 0.00

ATOM 5412 OG SER 49 39.948 51.795 81.602 1.00 0.00

ATOM 5413 HG SER 49 40.084 51.451 82.488 1.00 0.00

ATOM 5414 C SER 49 39.702 53.652 79.124 1.00 0.00

ATOM 5415 O SER 49 40.436 54.314 78.394 1.00 0.00

ATOM 5416 N ALA 50 38.931 52.676 78.639 1.00 0.00

ATOM 5417 H ALA 50 38.348 52.171 79.292 1.00 0.00

ATOM 5418 CA ALA 50 38.996 52.247 77.257 1.00 0.00

ATOM 5419 HA ALA 50 40.019 52.377 76.904 1.00 0.00

ATOM 5420 CB ALA 50 38.641 50.763 77.233 1.00 0.00

ATOM 5421 HB1 ALA 50 37.594 50.613 77.495 1.00 0.00

ATOM 5422 HB2 ALA 50 38.833 50.396 76.225 1.00 0.00

ATOM 5423 HB3 ALA 50 39.256 50.278 77.991 1.00 0.00

ATOM 5424 C ALA 50 38.013 53.031 76.398 1.00 0.00

ATOM 5425 O ALA 50 38.168 53.108 75.182 1.00 0.00

ATOM 5426 N PHE 51 37.040 53.732 76.982 1.00 0.00

ATOM 5427 H PHE 51 36.773 53.414 77.903 1.00 0.00

ATOM 5428 CA PHE 51 36.228 54.682 76.248 1.00 0.00

ATOM 5429 HA PHE 51 36.034 54.234 75.273 1.00 0.00

ATOM 5430 CB PHE 51 34.902 54.768 76.998 1.00 0.00

ATOM 5431 HB1 PHE 51 35.089 55.104 78.018 1.00 0.00

ATOM 5432 HB2 PHE 51 34.582 53.727 76.948 1.00 0.00

ATOM 5433 CG PHE 51 33.875 55.662 76.346 1.00 0.00

ATOM 5434 CD1 PHE 51 32.933 55.170 75.436 1.00 0.00

ATOM 5435 HD1 PHE 51 33.017 54.144 75.108 1.00 0.00

ATOM 5436 CE1 PHE 51 32.040 56.056 74.822 1.00 0.00

ATOM 5437 HE1 PHE 51 31.358 55.716 74.056 1.00 0.00

ATOM 5438 CZ PHE 51 31.836 57.318 75.392 1.00 0.00

ATOM 5439 HZ PHE 51 31.164 57.985 74.872 1.00 0.00

ATOM 5440 CE2 PHE 51 32.714 57.798 76.372 1.00 0.00

ATOM 5441 HE2 PHE 51 32.744 58.844 76.638 1.00 0.00

ATOM 5442 CD2 PHE 51 33.801 57.001 76.749 1.00 0.00

ATOM 5443 HD2 PHE 51 34.629 57.405 77.312 1.00 0.00

ATOM 5444 C PHE 51 36.869 56.060 76.148 1.00 0.00

ATOM 5445 O PHE 51 36.699 56.776 75.164 1.00 0.00

ATOM 5446 N ALA 52 37.638 56.438 77.172 1.00 0.00

ATOM 5447 H ALA 52 37.753 55.857 77.990 1.00 0.00

ATOM 5448 CA ALA 52 38.408 57.660 77.284 1.00 0.00

ATOM 5449 HA ALA 52 37.681 58.460 77.145 1.00 0.00

ATOM 5450 CB ALA 52 39.003 57.750 78.686 1.00 0.00

ATOM 5451 HB1 ALA 52 39.429 56.771 78.905 1.00 0.00

ATOM 5452 HB2 ALA 52 39.696 58.591 78.709 1.00 0.00

ATOM 5453 HB3 ALA 52 38.226 57.902 79.436 1.00 0.00

ATOM 5454 C ALA 52 39.403 57.763 76.136 1.00 0.00

ATOM 5455 O ALA 52 39.608 58.856 75.613 1.00 0.00

ATOM 5456 N ASP 53 39.957 56.632 75.695 1.00 0.00

ATOM 5457 H ASP 53 39.767 55.756 76.161 1.00 0.00

ATOM 5458 CA ASP 53 40.858 56.560 74.561 1.00 0.00

ATOM 5459 HA ASP 53 41.637 57.308 74.699 1.00 0.00

ATOM 5460 CB ASP 53 41.525 55.187 74.582 1.00 0.00

ATOM 5461 HB1 ASP 53 41.899 54.852 75.549 1.00 0.00

ATOM 5462 HB2 ASP 53 40.701 54.552 74.258 1.00 0.00

ATOM 5463 CG ASP 53 42.756 55.132 73.688 1.00 0.00

ATOM 5464 OD1 ASP 53 43.690 55.906 73.992 1.00 0.00

ATOM 5465 OD2 ASP 53 42.866 54.241 72.820 1.00 0.00

ATOM 5466 C ASP 53 40.264 56.957 73.216 1.00 0.00

ATOM 5467 O ASP 53 40.869 57.528 72.312 1.00 0.00

ATOM 5468 N ASP 54 38.983 56.666 72.983 1.00 0.00

ATOM 5469 H ASP 54 38.405 56.226 73.685 1.00 0.00

ATOM 5470 CA ASP 54 38.254 56.974 71.768 1.00 0.00

ATOM 5471 HA ASP 54 38.969 56.854 70.955 1.00 0.00

ATOM 5472 CB ASP 54 37.213 55.897 71.478 1.00 0.00

ATOM 5473 HB1 ASP 54 37.733 54.990 71.782 1.00 0.00

ATOM 5474 HB2 ASP 54 36.276 56.154 71.975 1.00 0.00

ATOM 5475 CG ASP 54 37.011 55.904 69.969 1.00 0.00

ATOM 5476 OD1 ASP 54 37.813 55.366 69.186 1.00 0.00

ATOM 5477 OD2 ASP 54 35.984 56.474 69.542 1.00 0.00

ATOM 5478 C ASP 54 37.676 58.379 71.684 1.00 0.00

ATOM 5479 O ASP 54 37.373 58.880 70.604 1.00 0.00

ATOM 5480 N GLN 55 37.451 58.953 72.868 1.00 0.00

ATOM 5481 H GLN 55 37.524 58.389 73.703 1.00 0.00

ATOM 5482 CA GLN 55 37.131 60.348 73.101 1.00 0.00

ATOM 5483 HA GLN 55 36.569 60.778 72.273 1.00 0.00

ATOM 5484 CB GLN 55 36.211 60.493 74.309 1.00 0.00

ATOM 5485 HB1 GLN 55 36.331 61.443 74.830 1.00 0.00

ATOM 5486 HB2 GLN 55 36.567 59.686 74.947 1.00 0.00

ATOM 5487 CG GLN 55 34.720 60.299 74.048 1.00 0.00

ATOM 5488 HG1 GLN 55 34.203 60.246 75.006 1.00 0.00

ATOM 5489 HG2 GLN 55 34.343 61.234 73.633 1.00 0.00

ATOM 5490 CD GLN 55 34.288 59.116 73.193 1.00 0.00

ATOM 5491 OE1 GLN 55 33.566 59.345 72.225 1.00 0.00

ATOM 5492 NE2 GLN 55 34.600 57.857 73.509 1.00 0.00

ATOM 5493 1HE2 GLN 55 35.288 57.553 74.183 1.00 0.00

ATOM 5494 2HE2 GLN 55 34.103 57.110 73.045 1.00 0.00

ATOM 5495 C GLN 55 38.349 61.245 73.258 1.00 0.00

ATOM 5496 O GLN 55 38.196 62.464 73.302 1.00 0.00

ATOM 5497 N THR 56 39.566 60.705 73.156 1.00 0.00

ATOM 5498 H THR 56 39.572 59.712 72.978 1.00 0.00

ATOM 5499 CA THR 56 40.857 61.305 73.428 1.00 0.00

ATOM 5500 HA THR 56 41.536 60.464 73.571 1.00 0.00

ATOM 5501 CB THR 56 41.375 61.988 72.165 1.00 0.00

ATOM 5502 HB THR 56 40.734 62.845 71.957 1.00 0.00

ATOM 5503 CG2 THR 56 42.877 62.251 72.094 1.00 0.00

ATOM 5504 1HG2 THR 56 43.485 61.361 71.932 1.00 0.00

ATOM 5505 2HG2 THR 56 43.235 62.583 73.067 1.00 0.00

ATOM 5506 3HG2 THR 56 43.148 63.086 71.466 1.00 0.00

ATOM 5507 OG1 THR 56 41.311 61.056 71.110 1.00 0.00

ATOM 5508 HG1 THR 56 40.401 61.273 70.898 1.00 0.00

ATOM 5509 C THR 56 40.864 62.229 74.637 1.00 0.00

ATOM 5510 O THR 56 41.305 63.374 74.566 1.00 0.00

ATOM 5511 N TYR 57 40.336 61.734 75.759 1.00 0.00

ATOM 5512 H TYR 57 40.079 60.757 75.762 1.00 0.00

ATOM 5513 CA TYR 57 40.102 62.552 76.933 1.00 0.00

ATOM 5514 HA TYR 57 40.952 63.221 77.051 1.00 0.00

ATOM 5515 CB TYR 57 38.810 63.359 76.835 1.00 0.00

ATOM 5516 HB1 TYR 57 38.008 62.634 76.724 1.00 0.00

ATOM 5517 HB2 TYR 57 38.845 63.967 75.931 1.00 0.00

ATOM 5518 CG TYR 57 38.411 64.274 77.968 1.00 0.00

ATOM 5519 CD1 TYR 57 39.244 65.340 78.326 1.00 0.00

ATOM 5520 HD1 TYR 57 40.196 65.375 77.817 1.00 0.00

ATOM 5521 CE1 TYR 57 38.984 66.127 79.454 1.00 0.00

ATOM 5522 HE1 TYR 57 39.699 66.875 79.762 1.00 0.00

ATOM 5523 CZ TYR 57 37.806 65.890 80.189 1.00 0.00

ATOM 5524 OH TYR 57 37.597 66.813 81.170 1.00 0.00

ATOM 5525 HH TYR 57 36.766 66.570 81.583 1.00 0.00

ATOM 5526 CE2 TYR 57 36.923 64.853 79.825 1.00 0.00

ATOM 5527 HE2 TYR 57 36.093 64.555 80.450 1.00 0.00

ATOM 5528 CD2 TYR 57 37.266 64.039 78.739 1.00 0.00

ATOM 5529 HD2 TYR 57 36.590 63.217 78.550 1.00 0.00

ATOM 5530 C TYR 57 40.131 61.811 78.262 1.00 0.00

ATOM 5531 O TYR 57 39.373 60.847 78.311 1.00 0.00

ATOM 5532 N PRO 58 40.847 62.151 79.338 1.00 0.00

ATOM 5533 CD PRO 58 41.540 63.377 79.676 1.00 0.00

ATOM 5534 HD1 PRO 58 40.944 64.057 80.285 1.00 0.00

ATOM 5535 HD2 PRO 58 41.985 63.814 78.782 1.00 0.00

ATOM 5536 CG PRO 58 42.680 62.915 80.579 1.00 0.00

ATOM 5537 HG1 PRO 58 42.868 63.747 81.257 1.00 0.00

ATOM 5538 HG2 PRO 58 43.534 62.626 79.966 1.00 0.00

ATOM 5539 CB PRO 58 42.146 61.737 81.387 1.00 0.00

ATOM 5540 HB1 PRO 58 41.712 61.954 82.364 1.00 0.00

ATOM 5541 HB2 PRO 58 42.988 61.051 81.481 1.00 0.00

ATOM 5542 CA PRO 58 41.066 61.230 80.436 1.00 0.00

ATOM 5543 HA PRO 58 41.455 60.329 79.964 1.00 0.00

ATOM 5544 C PRO 58 39.859 60.799 81.257 1.00 0.00

ATOM 5545 O PRO 58 38.979 61.641 81.412 1.00 0.00

ATOM 5546 N PRO 59 39.842 59.631 81.905 1.00 0.00

ATOM 5547 CD PRO 59 40.905 58.647 81.922 1.00 0.00

ATOM 5548 HD1 PRO 59 41.856 59.003 82.319 1.00 0.00

ATOM 5549 HD2 PRO 59 41.162 58.264 80.934 1.00 0.00

ATOM 5550 CG PRO 59 40.329 57.541 82.801 1.00 0.00

ATOM 5551 HG1 PRO 59 40.594 57.687 83.848 1.00 0.00

ATOM 5552 HG2 PRO 59 40.673 56.542 82.534 1.00 0.00

ATOM 5553 CB PRO 59 38.821 57.753 82.903 1.00 0.00

ATOM 5554 HB1 PRO 59 38.545 57.469 83.919 1.00 0.00

ATOM 5555 HB2 PRO 59 38.306 57.179 82.134 1.00 0.00

ATOM 5556 CA PRO 59 38.602 59.214 82.527 1.00 0.00

ATOM 5557 HA PRO 59 37.831 59.261 81.756 1.00 0.00

ATOM 5558 C PRO 59 38.328 59.966 83.822 1.00 0.00

ATOM 5559 O PRO 59 39.204 60.647 84.350 1.00 0.00

ATOM 5560 N GLU 60 37.071 59.868 84.262 1.00 0.00

ATOM 5561 H GLU 60 36.541 59.106 83.862 1.00 0.00

ATOM 5562 CA GLU 60 36.452 60.473 85.424 1.00 0.00

ATOM 5563 HA GLU 60 35.394 60.602 85.193 1.00 0.00

ATOM 5564 CB GLU 60 36.499 59.483 86.583 1.00 0.00

ATOM 5565 HB1 GLU 60 35.910 58.600 86.335 1.00 0.00

ATOM 5566 HB2 GLU 60 36.022 59.923 87.459 1.00 0.00

ATOM 5567 CG GLU 60 37.885 59.049 87.049 1.00 0.00

ATOM 5568 HG1 GLU 60 38.529 58.754 86.222 1.00 0.00

ATOM 5569 HG2 GLU 60 38.332 59.958 87.420 1.00 0.00

ATOM 5570 CD GLU 60 37.764 58.066 88.206 1.00 0.00

ATOM 5571 OE1 GLU 60 38.291 58.296 89.317 1.00 0.00

ATOM 5572 OE2 GLU 60 37.211 56.957 88.050 1.00 0.00

ATOM 5573 C GLU 60 36.820 61.912 85.759 1.00 0.00

ATOM 5574 O GLU 60 36.710 62.298 86.917 1.00 0.00

ATOM 5575 N SER 61 37.090 62.690 84.707 1.00 0.00

ATOM 5576 H SER 61 37.120 62.329 83.765 1.00 0.00

ATOM 5577 CA SER 61 37.530 64.070 84.708 1.00 0.00

ATOM 5578 HA SER 61 37.803 64.204 85.753 1.00 0.00

ATOM 5579 CB SER 61 38.756 64.227 83.813 1.00 0.00

ATOM 5580 HB1 SER 61 38.472 64.124 82.767 1.00 0.00

ATOM 5581 HB2 SER 61 39.066 65.256 83.916 1.00 0.00

ATOM 5582 OG SER 61 39.723 63.288 84.229 1.00 0.00

ATOM 5583 HG SER 61 39.397 62.383 84.213 1.00 0.00

ATOM 5584 C SER 61 36.430 65.086 84.434 1.00 0.00

ATOM 5585 OC1 SER 61 36.110 65.899 85.328 1.00 0.00

ATOM 5586 OC2 SER 61 35.864 64.971 83.326 1.00 0.00

ATOM 5587 N PHE 1 40.288 40.664 154.473 1.00 0.00

ATOM 5588 H1 PHE 1 40.912 40.879 153.710 1.00 0.00

ATOM 5589 H2 PHE 1 40.901 40.487 155.257 1.00 0.00

ATOM 5590 H3 PHE 1 39.638 41.432 154.554 1.00 0.00

ATOM 5591 CA PHE 1 39.508 39.487 154.065 1.00 0.00

ATOM 5592 HA PHE 1 38.865 39.910 153.293 1.00 0.00

ATOM 5593 CB PHE 1 40.395 38.437 153.399 1.00 0.00

ATOM 5594 HB1 PHE 1 41.160 37.984 154.029 1.00 0.00

ATOM 5595 HB2 PHE 1 40.880 38.945 152.566 1.00 0.00

ATOM 5596 CG PHE 1 39.549 37.346 152.787 1.00 0.00

ATOM 5597 CD1 PHE 1 39.603 36.040 153.288 1.00 0.00

ATOM 5598 HD1 PHE 1 40.145 35.875 154.208 1.00 0.00

ATOM 5599 CE1 PHE 1 38.816 35.037 152.712 1.00 0.00

ATOM 5600 HE1 PHE 1 38.932 34.075 153.161 1.00 0.00

ATOM 5601 CZ PHE 1 38.112 35.313 151.533 1.00 0.00

ATOM 5602 HZ PHE 1 37.702 34.494 150.961 1.00 0.00

ATOM 5603 CE2 PHE 1 38.003 36.615 151.030 1.00 0.00

ATOM 5604 HE2 PHE 1 37.530 36.816 150.080 1.00 0.00

ATOM 5605 CD2 PHE 1 38.745 37.614 151.673 1.00 0.00

ATOM 5606 HD2 PHE 1 38.752 38.640 151.338 1.00 0.00

ATOM 5607 C PHE 1 38.653 39.045 155.244 1.00 0.00

ATOM 5608 O PHE 1 39.138 38.373 156.153 1.00 0.00

ATOM 5609 N THR 2 37.380 39.440 155.173 1.00 0.00

ATOM 5610 H THR 2 37.040 40.102 154.491 1.00 0.00

ATOM 5611 CA THR 2 36.321 39.180 156.128 1.00 0.00

ATOM 5612 HA THR 2 36.661 38.685 157.016 1.00 0.00

ATOM 5613 CB THR 2 35.650 40.487 156.542 1.00 0.00

ATOM 5614 HB THR 2 34.858 40.338 157.276 1.00 0.00

ATOM 5615 CG2 THR 2 36.667 41.501 157.055 1.00 0.00

ATOM 5616 1HG2 THR 2 37.255 41.121 157.892 1.00 0.00

ATOM 5617 2HG2 THR 2 36.117 42.379 157.391 1.00 0.00

ATOM 5618 3HG2 THR 2 37.468 41.670 156.335 1.00 0.00

ATOM 5619 OG1 THR 2 35.159 41.110 155.376 1.00 0.00

ATOM 5620 HG1 THR 2 35.828 41.214 154.695 1.00 0.00

ATOM 5621 C THR 2 35.186 38.341 155.560 1.00 0.00

ATOM 5622 O THR 2 35.236 38.181 154.343 1.00 0.00

ATOM 5623 N LEU 3 34.179 37.911 156.326 1.00 0.00

ATOM 5624 H LEU 3 34.229 38.054 157.324 1.00 0.00

ATOM 5625 CA LEU 3 32.989 37.251 155.826 1.00 0.00

ATOM 5626 HA LEU 3 33.338 36.456 155.169 1.00 0.00

ATOM 5627 CB LEU 3 32.148 36.627 156.937 1.00 0.00

ATOM 5628 HB1 LEU 3 31.851 37.392 157.654 1.00 0.00

ATOM 5629 HB2 LEU 3 32.712 35.833 157.428 1.00 0.00

ATOM 5630 CG LEU 3 30.796 36.041 156.542 1.00 0.00

ATOM 5631 HG LEU 3 30.132 36.776 156.260 1.00 0.00

ATOM 5632 CD1 LEU 3 30.918 34.993 155.439 1.00 0.00

ATOM 5633 1HD1 LEU 3 31.175 35.426 154.472 1.00 0.00

ATOM 5634 2HD1 LEU 3 29.985 34.467 155.240 1.00 0.00

ATOM 5635 3HD1 LEU 3 31.738 34.333 155.721 1.00 0.00

ATOM 5636 CD2 LEU 3 30.077 35.480 157.766 1.00 0.00

ATOM 5637 1HD2 LEU 3 29.209 34.982 157.335 1.00 0.00

ATOM 5638 2HD2 LEU 3 29.735 36.204 158.506 1.00 0.00

ATOM 5639 3HD2 LEU 3 30.703 34.724 158.240 1.00 0.00

ATOM 5640 C LEU 3 32.157 38.193 154.969 1.00 0.00

ATOM 5641 O LEU 3 31.458 37.769 154.051 1.00 0.00

ATOM 5642 N ILE 4 32.222 39.504 155.213 1.00 0.00

ATOM 5643 H ILE 4 32.751 39.826 156.010 1.00 0.00

ATOM 5644 CA ILE 4 31.389 40.485 154.547 1.00 0.00

ATOM 5645 HA ILE 4 30.330 40.290 154.716 1.00 0.00

ATOM 5646 CB ILE 4 31.563 41.908 155.071 1.00 0.00

ATOM 5647 HB ILE 4 32.619 42.110 155.251 1.00 0.00

ATOM 5648 CG2 ILE 4 31.001 42.985 154.146 1.00 0.00

ATOM 5649 1HG2 ILE 4 31.474 43.001 153.166 1.00 0.00

ATOM 5650 2HG2 ILE 4 31.094 43.986 154.568 1.00 0.00

ATOM 5651 3HG2 ILE 4 29.966 42.815 153.849 1.00 0.00

ATOM 5652 CG1 ILE 4 30.807 42.097 156.382 1.00 0.00

ATOM 5653 1HG1 ILE 4 30.896 43.159 156.610 1.00 0.00

ATOM 5654 2HG1 ILE 4 29.789 41.855 156.074 1.00 0.00

ATOM 5655 CD ILE 4 31.310 41.260 157.555 1.00 0.00

ATOM 5656 HD1 ILE 4 32.372 41.422 157.742 1.00 0.00

ATOM 5657 HD2 ILE 4 31.106 40.208 157.357 1.00 0.00

ATOM 5658 HD3 ILE 4 30.760 41.505 158.464 1.00 0.00

ATOM 5659 C ILE 4 31.830 40.463 153.090 1.00 0.00

ATOM 5660 O ILE 4 30.957 40.391 152.230 1.00 0.00

ATOM 5661 N GLU 5 33.107 40.381 152.704 1.00 0.00

ATOM 5662 H GLU 5 33.872 40.323 153.360 1.00 0.00

ATOM 5663 CA GLU 5 33.517 40.250 151.320 1.00 0.00

ATOM 5664 HA GLU 5 33.087 41.020 150.679 1.00 0.00

ATOM 5665 CB GLU 5 35.037 40.375 151.351 1.00 0.00

ATOM 5666 HB1 GLU 5 35.518 40.054 150.428 1.00 0.00

ATOM 5667 HB2 GLU 5 35.505 39.603 151.963 1.00 0.00

ATOM 5668 CG GLU 5 35.474 41.823 151.554 1.00 0.00

ATOM 5669 HG1 GLU 5 35.328 42.488 150.702 1.00 0.00

ATOM 5670 HG2 GLU 5 34.878 42.322 152.316 1.00 0.00

ATOM 5671 CD GLU 5 36.896 41.860 152.094 1.00 0.00

ATOM 5672 OE1 GLU 5 37.048 41.494 153.281 1.00 0.00

ATOM 5673 OE2 GLU 5 37.848 42.296 151.414 1.00 0.00

ATOM 5674 C GLU 5 33.140 38.964 150.597 1.00 0.00

ATOM 5675 O GLU 5 33.342 38.938 149.386 1.00 0.00

ATOM 5676 N LEU 6 32.418 38.010 151.188 1.00 0.00

ATOM 5677 H LEU 6 32.358 38.184 152.180 1.00 0.00

ATOM 5678 CA LEU 6 31.920 36.763 150.641 1.00 0.00

ATOM 5679 HA LEU 6 32.226 36.655 149.600 1.00 0.00

ATOM 5680 CB LEU 6 32.489 35.600 151.449 1.00 0.00

ATOM 5681 HB1 LEU 6 32.302 35.685 152.519 1.00 0.00

ATOM 5682 HB2 LEU 6 33.576 35.665 151.445 1.00 0.00

ATOM 5683 CG LEU 6 32.135 34.173 151.040 1.00 0.00

ATOM 5684 HG LEU 6 32.871 33.463 151.416 1.00 0.00

ATOM 5685 CD1 LEU 6 30.830 33.700 151.674 1.00 0.00

ATOM 5686 1HD1 LEU 6 30.823 33.885 152.749 1.00 0.00

ATOM 5687 2HD1 LEU 6 30.893 32.616 151.581 1.00 0.00

ATOM 5688 3HD1 LEU 6 29.984 34.004 151.057 1.00 0.00

ATOM 5689 CD2 LEU 6 32.042 33.994 149.527 1.00 0.00

ATOM 5690 1HD2 LEU 6 32.897 34.381 148.974 1.00 0.00

ATOM 5691 2HD2 LEU 6 31.102 34.393 149.147 1.00 0.00

ATOM 5692 3HD2 LEU 6 31.993 32.931 149.288 1.00 0.00

ATOM 5693 C LEU 6 30.399 36.812 150.689 1.00 0.00

ATOM 5694 O LEU 6 29.701 36.557 149.710 1.00 0.00

ATOM 5695 N LEU 7 29.757 36.975 151.848 1.00 0.00

ATOM 5696 H LEU 7 30.230 37.163 152.721 1.00 0.00

ATOM 5697 CA LEU 7 28.322 36.792 151.938 1.00 0.00

ATOM 5698 HA LEU 7 28.136 35.880 151.371 1.00 0.00

ATOM 5699 CB LEU 7 27.988 36.539 153.405 1.00 0.00

ATOM 5700 HB1 LEU 7 27.991 37.490 153.935 1.00 0.00

ATOM 5701 HB2 LEU 7 28.877 35.999 153.730 1.00 0.00

ATOM 5702 CG LEU 7 26.794 35.724 153.899 1.00 0.00

ATOM 5703 HG LEU 7 26.911 35.445 154.946 1.00 0.00

ATOM 5704 CD1 LEU 7 25.520 36.560 153.972 1.00 0.00

ATOM 5705 1HD1 LEU 7 24.722 35.921 154.350 1.00 0.00

ATOM 5706 2HD1 LEU 7 25.267 36.964 152.992 1.00 0.00

ATOM 5707 3HD1 LEU 7 25.810 37.419 154.577 1.00 0.00

ATOM 5708 CD2 LEU 7 26.579 34.422 153.134 1.00 0.00

ATOM 5709 1HD2 LEU 7 26.099 34.625 152.177 1.00 0.00

ATOM 5710 2HD2 LEU 7 25.865 33.774 153.642 1.00 0.00

ATOM 5711 3HD2 LEU 7 27.538 33.927 152.981 1.00 0.00

ATOM 5712 C LEU 7 27.600 37.913 151.203 1.00 0.00

ATOM 5713 O LEU 7 26.440 37.668 150.883 1.00 0.00

ATOM 5714 N ILE 8 28.082 39.118 150.893 1.00 0.00

ATOM 5715 H ILE 8 29.042 39.308 151.142 1.00 0.00

ATOM 5716 CA ILE 8 27.345 40.184 150.242 1.00 0.00

ATOM 5717 HA ILE 8 26.380 40.309 150.734 1.00 0.00

ATOM 5718 CB ILE 8 28.140 41.462 150.492 1.00 0.00

ATOM 5719 HB ILE 8 28.260 41.385 151.573 1.00 0.00

ATOM 5720 CG2 ILE 8 29.521 41.428 149.843 1.00 0.00

ATOM 5721 1HG2 ILE 8 29.983 40.443 149.912 1.00 0.00

ATOM 5722 2HG2 ILE 8 30.164 42.128 150.377 1.00 0.00

ATOM 5723 3HG2 ILE 8 29.465 41.596 148.768 1.00 0.00

ATOM 5724 CG1 ILE 8 27.280 42.690 150.206 1.00 0.00

ATOM 5725 1HG1 ILE 8 26.826 42.642 149.216 1.00 0.00

ATOM 5726 2HG1 ILE 8 26.471 42.565 150.925 1.00 0.00

ATOM 5727 CD ILE 8 27.969 44.030 150.446 1.00 0.00

ATOM 5728 HD1 ILE 8 27.281 44.855 150.263 1.00 0.00

ATOM 5729 HD2 ILE 8 28.904 44.228 149.921 1.00 0.00

ATOM 5730 HD3 ILE 8 28.150 44.107 151.517 1.00 0.00

ATOM 5731 C ILE 8 27.127 39.843 148.775 1.00 0.00

ATOM 5732 O ILE 8 26.115 40.257 148.214 1.00 0.00

ATOM 5733 N VAL 9 27.931 38.995 148.128 1.00 0.00

ATOM 5734 H VAL 9 28.838 38.783 148.520 1.00 0.00

ATOM 5735 CA VAL 9 27.636 38.510 146.794 1.00 0.00

ATOM 5736 HA VAL 9 27.142 39.320 146.258 1.00 0.00

ATOM 5737 CB VAL 9 28.883 38.232 145.962 1.00 0.00

ATOM 5738 HB VAL 9 29.325 37.283 146.266 1.00 0.00

ATOM 5739 CG1 VAL 9 28.544 38.127 144.479 1.00 0.00

ATOM 5740 1HG1 VAL 9 27.799 37.348 144.308 1.00 0.00

ATOM 5741 2HG1 VAL 9 29.499 37.779 144.087 1.00 0.00

ATOM 5742 3HG1 VAL 9 28.327 39.120 144.082 1.00 0.00

ATOM 5743 CG2 VAL 9 29.910 39.356 146.059 1.00 0.00

ATOM 5744 1HG2 VAL 9 30.259 39.306 147.090 1.00 0.00

ATOM 5745 2HG2 VAL 9 29.489 40.316 145.758 1.00 0.00

ATOM 5746 3HG2 VAL 9 30.789 39.228 145.426 1.00 0.00

ATOM 5747 C VAL 9 26.670 37.333 146.771 1.00 0.00

ATOM 5748 O VAL 9 25.844 37.179 145.875 1.00 0.00

ATOM 5749 N VAL 10 26.733 36.448 147.767 1.00 0.00

ATOM 5750 H VAL 10 27.329 36.792 148.506 1.00 0.00

ATOM 5751 CA VAL 10 25.727 35.430 147.997 1.00 0.00

ATOM 5752 HA VAL 10 25.593 34.772 147.138 1.00 0.00

ATOM 5753 CB VAL 10 26.254 34.589 149.156 1.00 0.00

ATOM 5754 HB VAL 10 26.327 35.212 150.048 1.00 0.00

ATOM 5755 CG1 VAL 10 25.369 33.369 149.397 1.00 0.00

ATOM 5756 1HG1 VAL 10 25.774 32.749 150.194 1.00 0.00

ATOM 5757 2HG1 VAL 10 25.318 32.846 148.447 1.00 0.00

ATOM 5758 3HG1 VAL 10 24.354 33.727 149.568 1.00 0.00

ATOM 5759 CG2 VAL 10 27.651 34.027 148.901 1.00 0.00

ATOM 5760 1HG2 VAL 10 28.497 34.706 148.998 1.00 0.00

ATOM 5761 2HG2 VAL 10 27.681 33.504 147.945 1.00 0.00

ATOM 5762 3HG2 VAL 10 27.978 33.332 149.674 1.00 0.00

ATOM 5763 C VAL 10 24.334 35.951 148.320 1.00 0.00

ATOM 5764 O VAL 10 23.374 35.330 147.872 1.00 0.00

ATOM 5765 N ALA 11 24.196 37.063 149.046 1.00 0.00

ATOM 5766 H ALA 11 24.998 37.387 149.567 1.00 0.00

ATOM 5767 CA ALA 11 23.009 37.851 149.304 1.00 0.00

ATOM 5768 HA ALA 11 22.364 37.337 150.018 1.00 0.00

ATOM 5769 CB ALA 11 23.474 39.087 150.070 1.00 0.00

ATOM 5770 HB1 ALA 11 23.865 39.885 149.438 1.00 0.00

ATOM 5771 HB2 ALA 11 24.278 38.856 150.768 1.00 0.00

ATOM 5772 HB3 ALA 11 22.622 39.564 150.555 1.00 0.00

ATOM 5773 C ALA 11 22.235 38.249 148.056 1.00 0.00

ATOM 5774 O ALA 11 21.018 38.237 148.186 1.00 0.00

ATOM 5775 N ILE 12 22.984 38.537 146.987 1.00 0.00

ATOM 5776 H ILE 12 23.993 38.586 147.010 1.00 0.00

ATOM 5777 CA ILE 12 22.370 38.924 145.735 1.00 0.00

ATOM 5778 HA ILE 12 21.291 38.801 145.834 1.00 0.00

ATOM 5779 CB ILE 12 22.547 40.418 145.474 1.00 0.00

ATOM 5780 HB ILE 12 21.796 40.747 144.756 1.00 0.00

ATOM 5781 CG2 ILE 12 22.207 41.267 146.696 1.00 0.00

ATOM 5782 1HG2 ILE 12 22.263 42.326 146.449 1.00 0.00

ATOM 5783 2HG2 ILE 12 22.995 41.288 147.448 1.00 0.00

ATOM 5784 3HG2 ILE 12 21.230 41.046 147.126 1.00 0.00

ATOM 5785 CG1 ILE 12 23.854 40.917 144.863 1.00 0.00

ATOM 5786 1HG1 ILE 12 24.584 41.194 145.623 1.00 0.00

ATOM 5787 2HG1 ILE 12 24.316 40.135 144.261 1.00 0.00

ATOM 5788 CD ILE 12 23.705 42.060 143.862 1.00 0.00

ATOM 5789 HD1 ILE 12 23.015 42.824 144.220 1.00 0.00

ATOM 5790 HD2 ILE 12 23.218 41.662 142.972 1.00 0.00

ATOM 5791 HD3 ILE 12 24.636 42.568 143.611 1.00 0.00

ATOM 5792 C ILE 12 22.649 38.057 144.515 1.00 0.00

ATOM 5793 O ILE 12 22.549 38.426 143.346 1.00 0.00

ATOM 5794 N ILE 13 23.011 36.788 144.703 1.00 0.00

ATOM 5795 H ILE 13 23.145 36.588 145.683 1.00 0.00

ATOM 5796 CA ILE 13 23.516 35.949 143.632 1.00 0.00

ATOM 5797 HA ILE 13 24.263 36.503 143.065 1.00 0.00

ATOM 5798 CB ILE 13 24.189 34.723 144.242 1.00 0.00

ATOM 5799 HB ILE 13 24.972 35.035 144.932 1.00 0.00

ATOM 5800 CG2 ILE 13 23.261 33.779 145.004 1.00 0.00

ATOM 5801 1HG2 ILE 13 22.530 33.318 144.341 1.00 0.00

ATOM 5802 2HG2 ILE 13 22.694 34.271 145.794 1.00 0.00

ATOM 5803 3HG2 ILE 13 23.729 32.982 145.578 1.00 0.00

ATOM 5804 CG1 ILE 13 25.076 34.012 143.225 1.00 0.00

ATOM 5805 1HG1 ILE 13 25.297 33.047 143.682 1.00 0.00

ATOM 5806 2HG1 ILE 13 24.589 33.925 142.254 1.00 0.00

ATOM 5807 CD ILE 13 26.414 34.722 143.046 1.00 0.00

ATOM 5808 HD1 ILE 13 26.268 35.687 142.562 1.00 0.00

ATOM 5809 HD2 ILE 13 27.010 34.086 142.391 1.00 0.00

ATOM 5810 HD3 ILE 13 26.857 34.796 144.040 1.00 0.00

ATOM 5811 C ILE 13 22.516 35.648 142.525 1.00 0.00

ATOM 5812 O ILE 13 22.828 35.604 141.338 1.00 0.00

ATOM 5813 N GLY 14 21.250 35.698 142.949 1.00 0.00

ATOM 5814 H GLY 14 21.092 35.965 143.910 1.00 0.00

ATOM 5815 CA GLY 14 20.158 35.397 142.045 1.00 0.00

ATOM 5816 HA1 GLY 14 20.404 34.491 141.492 1.00 0.00

ATOM 5817 HA2 GLY 14 19.216 35.112 142.513 1.00 0.00

ATOM 5818 C GLY 14 19.850 36.511 141.054 1.00 0.00

ATOM 5819 O GLY 14 19.403 36.221 139.949 1.00 0.00

ATOM 5820 N ILE 15 20.114 37.797 141.299 1.00 0.00

ATOM 5821 H ILE 15 20.396 38.155 142.200 1.00 0.00

ATOM 5822 CA ILE 15 19.952 38.836 140.301 1.00 0.00

ATOM 5823 HA ILE 15 19.101 38.607 139.660 1.00 0.00

ATOM 5824 CB ILE 15 19.619 40.194 140.914 1.00 0.00

ATOM 5825 HB ILE 15 18.678 40.106 141.457 1.00 0.00

ATOM 5826 CG2 ILE 15 20.608 40.662 141.977 1.00 0.00

ATOM 5827 1HG2 ILE 15 20.445 41.721 142.174 1.00 0.00

ATOM 5828 2HG2 ILE 15 21.631 40.553 141.615 1.00 0.00

ATOM 5829 3HG2 ILE 15 20.460 40.108 142.904 1.00 0.00

ATOM 5830 CG1 ILE 15 19.353 41.296 139.890 1.00 0.00

ATOM 5831 1HG1 ILE 15 20.235 41.612 139.334 1.00 0.00

ATOM 5832 2HG1 ILE 15 18.838 40.904 139.014 1.00 0.00

ATOM 5833 CD ILE 15 18.671 42.573 140.373 1.00 0.00

ATOM 5834 HD1 ILE 15 19.301 43.201 141.003 1.00 0.00

ATOM 5835 HD2 ILE 15 17.801 42.377 140.997 1.00 0.00

ATOM 5836 HD3 ILE 15 18.426 43.139 139.474 1.00 0.00

ATOM 5837 C ILE 15 21.158 38.917 139.377 1.00 0.00

ATOM 5838 O ILE 15 20.998 39.086 138.170 1.00 0.00

ATOM 5839 N LEU 16 22.347 38.601 139.897 1.00 0.00

ATOM 5840 H LEU 16 22.339 38.340 140.872 1.00 0.00

ATOM 5841 CA LEU 16 23.572 38.372 139.158 1.00 0.00

ATOM 5842 HA LEU 16 23.738 39.298 138.609 1.00 0.00

ATOM 5843 CB LEU 16 24.764 38.377 140.111 1.00 0.00

ATOM 5844 HB1 LEU 16 25.668 38.125 139.557 1.00 0.00

ATOM 5845 HB2 LEU 16 24.650 37.567 140.830 1.00 0.00

ATOM 5846 CG LEU 16 24.915 39.621 140.983 1.00 0.00

ATOM 5847 HG LEU 16 24.084 39.612 141.688 1.00 0.00

ATOM 5848 CD1 LEU 16 26.133 39.497 141.894 1.00 0.00

ATOM 5849 1HD1 LEU 16 25.825 38.959 142.791 1.00 0.00

ATOM 5850 2HD1 LEU 16 26.554 40.468 142.157 1.00 0.00

ATOM 5851 3HD1 LEU 16 26.973 38.991 141.417 1.00 0.00

ATOM 5852 CD2 LEU 16 25.120 40.977 140.314 1.00 0.00

ATOM 5853 1HD2 LEU 16 26.049 40.998 139.745 1.00 0.00

ATOM 5854 2HD2 LEU 16 25.090 41.764 141.069 1.00 0.00

ATOM 5855 3HD2 LEU 16 24.368 41.133 139.542 1.00 0.00

ATOM 5856 C LEU 16 23.481 37.160 138.239 1.00 0.00

ATOM 5857 O LEU 16 24.297 36.902 137.360 1.00 0.00

ATOM 5858 N ALA 17 22.487 36.313 138.512 1.00 0.00

ATOM 5859 H ALA 17 21.914 36.515 139.319 1.00 0.00

ATOM 5860 CA ALA 17 21.964 35.274 137.648 1.00 0.00

ATOM 5861 HA ALA 17 22.743 35.154 136.896 1.00 0.00

ATOM 5862 CB ALA 17 21.822 34.012 138.494 1.00 0.00

ATOM 5863 HB1 ALA 17 21.028 34.209 139.214 1.00 0.00

ATOM 5864 HB2 ALA 17 22.796 33.769 138.918 1.00 0.00

ATOM 5865 HB3 ALA 17 21.452 33.167 137.913 1.00 0.00

ATOM 5866 C ALA 17 20.746 35.683 136.832 1.00 0.00

ATOM 5867 O ALA 17 19.975 34.795 136.474 1.00 0.00

ATOM 5868 N ALA 18 20.583 36.979 136.558 1.00 0.00

ATOM 5869 H ALA 18 21.137 37.684 137.023 1.00 0.00

ATOM 5870 CA ALA 18 19.485 37.423 135.722 1.00 0.00

ATOM 5871 HA ALA 18 19.216 36.605 135.054 1.00 0.00

ATOM 5872 CB ALA 18 18.305 37.856 136.588 1.00 0.00

ATOM 5873 HB1 ALA 18 18.074 37.119 137.357 1.00 0.00

ATOM 5874 HB2 ALA 18 18.452 38.864 136.974 1.00 0.00

ATOM 5875 HB3 ALA 18 17.450 37.755 135.920 1.00 0.00

ATOM 5876 C ALA 18 19.888 38.571 134.807 1.00 0.00

ATOM 5877 O ALA 18 19.447 38.638 133.662 1.00 0.00

ATOM 5878 N ILE 19 20.631 39.591 135.241 1.00 0.00

ATOM 5879 H ILE 19 20.879 39.704 136.213 1.00 0.00

ATOM 5880 CA ILE 19 20.845 40.814 134.493 1.00 0.00

ATOM 5881 HA ILE 19 19.898 41.029 133.997 1.00 0.00

ATOM 5882 CB ILE 19 21.080 41.947 135.489 1.00 0.00

ATOM 5883 HB ILE 19 21.427 42.819 134.938 1.00 0.00

ATOM 5884 CG2 ILE 19 19.740 42.285 136.135 1.00 0.00

ATOM 5885 1HG2 ILE 19 19.501 41.528 136.882 1.00 0.00

ATOM 5886 2HG2 ILE 19 18.992 42.329 135.342 1.00 0.00

ATOM 5887 3HG2 ILE 19 19.676 43.239 136.658 1.00 0.00

ATOM 5888 CG1 ILE 19 22.185 41.811 136.534 1.00 0.00

ATOM 5889 1HG1 ILE 19 22.300 42.767 137.045 1.00 0.00

ATOM 5890 2HG1 ILE 19 21.847 41.155 137.336 1.00 0.00

ATOM 5891 CD ILE 19 23.532 41.301 136.033 1.00 0.00

ATOM 5892 HD1 ILE 19 24.066 41.940 135.328 1.00 0.00

ATOM 5893 HD2 ILE 19 23.450 40.253 135.742 1.00 0.00

ATOM 5894 HD3 ILE 19 24.209 41.373 136.884 1.00 0.00

ATOM 5895 C ILE 19 21.921 40.835 133.417 1.00 0.00

ATOM 5896 O ILE 19 22.151 41.816 132.717 1.00 0.00

ATOM 5897 N ALA 20 22.750 39.798 133.274 1.00 0.00

ATOM 5898 H ALA 20 22.663 38.999 133.885 1.00 0.00

ATOM 5899 CA ALA 20 23.775 39.716 132.253 1.00 0.00

ATOM 5900 HA ALA 20 23.558 40.416 131.446 1.00 0.00

ATOM 5901 CB ALA 20 25.086 40.133 132.913 1.00 0.00

ATOM 5902 HB1 ALA 20 25.341 39.316 133.588 1.00 0.00

ATOM 5903 HB2 ALA 20 25.861 40.206 132.150 1.00 0.00

ATOM 5904 HB3 ALA 20 25.088 41.079 133.453 1.00 0.00

ATOM 5905 C ALA 20 23.910 38.352 131.591 1.00 0.00

ATOM 5906 O ALA 20 24.944 37.971 131.048 1.00 0.00

ATOM 5907 N ILE 21 22.804 37.604 131.590 1.00 0.00

ATOM 5908 H ILE 21 22.120 37.952 132.247 1.00 0.00

ATOM 5909 CA ILE 21 22.683 36.332 130.907 1.00 0.00

ATOM 5910 HA ILE 21 23.684 36.103 130.540 1.00 0.00

ATOM 5911 CB ILE 21 22.356 35.174 131.847 1.00 0.00

ATOM 5912 HB ILE 21 23.113 35.393 132.600 1.00 0.00

ATOM 5913 CG2 ILE 21 21.005 35.251 132.552 1.00 0.00

ATOM 5914 1HG2 ILE 21 20.268 35.394 131.762 1.00 0.00

ATOM 5915 2HG2 ILE 21 21.019 36.069 133.271 1.00 0.00

ATOM 5916 3HG2 ILE 21 20.778 34.317 133.066 1.00 0.00

ATOM 5917 CG1 ILE 21 22.479 33.755 131.297 1.00 0.00

ATOM 5918 1HG1 ILE 21 22.394 32.969 132.048 1.00 0.00

ATOM 5919 2HG1 ILE 21 21.702 33.699 130.536 1.00 0.00

ATOM 5920 CD ILE 21 23.723 33.422 130.478 1.00 0.00

ATOM 5921 HD1 ILE 21 23.988 34.256 129.829 1.00 0.00

ATOM 5922 HD2 ILE 21 23.690 32.457 129.972 1.00 0.00

ATOM 5923 HD3 ILE 21 24.575 33.393 131.157 1.00 0.00

ATOM 5924 C ILE 21 21.693 36.343 129.750 1.00 0.00

ATOM 5925 O ILE 21 21.942 35.636 128.778 1.00 0.00

ATOM 5926 N PRO 22 20.705 37.237 129.666 1.00 0.00

ATOM 5927 CD PRO 22 20.219 38.082 130.738 1.00 0.00

ATOM 5928 HD1 PRO 22 20.799 38.996 130.863 1.00 0.00

ATOM 5929 HD2 PRO 22 20.388 37.665 131.730 1.00 0.00

ATOM 5930 CG PRO 22 18.774 38.387 130.350 1.00 0.00

ATOM 5931 HG1 PRO 22 18.494 39.365 130.741 1.00 0.00

ATOM 5932 HG2 PRO 22 18.109 37.641 130.786 1.00 0.00

ATOM 5933 CB PRO 22 18.799 38.415 128.825 1.00 0.00

ATOM 5934 HB1 PRO 22 19.154 39.393 128.503 1.00 0.00

ATOM 5935 HB2 PRO 22 17.853 38.065 128.413 1.00 0.00

ATOM 5936 CA PRO 22 19.823 37.326 128.520 1.00 0.00

ATOM 5937 HA PRO 22 19.299 36.370 128.510 1.00 0.00

ATOM 5938 C PRO 22 20.443 37.545 127.147 1.00 0.00

ATOM 5939 O PRO 22 19.986 37.113 126.091 1.00 0.00

ATOM 5940 N GLN 23 21.519 38.332 127.084 1.00 0.00

ATOM 5941 H GLN 23 21.863 38.722 127.950 1.00 0.00

ATOM 5942 CA GLN 23 22.187 38.683 125.847 1.00 0.00

ATOM 5943 HA GLN 23 21.381 39.018 125.195 1.00 0.00

ATOM 5944 CB GLN 23 23.217 39.786 126.072 1.00 0.00

ATOM 5945 HB1 GLN 23 23.767 39.974 125.151 1.00 0.00

ATOM 5946 HB2 GLN 23 23.954 39.465 126.808 1.00 0.00

ATOM 5947 CG GLN 23 22.613 41.124 126.488 1.00 0.00

ATOM 5948 HG1 GLN 23 23.388 41.886 126.409 1.00 0.00

ATOM 5949 HG2 GLN 23 21.871 41.507 125.788 1.00 0.00

ATOM 5950 CD GLN 23 22.014 41.149 127.889 1.00 0.00

ATOM 5951 OE1 GLN 23 22.412 40.490 128.846 1.00 0.00

ATOM 5952 NE2 GLN 23 20.968 41.941 128.126 1.00 0.00

ATOM 5953 1HE2 GLN 23 20.599 42.533 127.395 1.00 0.00

ATOM 5954 2HE2 GLN 23 20.722 42.086 129.095 1.00 0.00

ATOM 5955 C GLN 23 22.904 37.548 125.129 1.00 0.00

ATOM 5956 O GLN 23 22.712 37.377 123.927 1.00 0.00

ATOM 5957 N PHE 24 23.618 36.705 125.878 1.00 0.00

ATOM 5958 H PHE 24 23.793 36.887 126.856 1.00 0.00

ATOM 5959 CA PHE 24 24.162 35.431 125.450 1.00 0.00

ATOM 5960 HA PHE 24 24.721 35.652 124.542 1.00 0.00

ATOM 5961 CB PHE 24 25.034 34.827 126.548 1.00 0.00

ATOM 5962 HB1 PHE 24 24.441 34.642 127.444 1.00 0.00

ATOM 5963 HB2 PHE 24 25.760 35.606 126.782 1.00 0.00

ATOM 5964 CG PHE 24 25.757 33.550 126.191 1.00 0.00

ATOM 5965 CD1 PHE 24 26.541 33.390 125.042 1.00 0.00

ATOM 5966 HD1 PHE 24 26.684 34.109 124.249 1.00 0.00

ATOM 5967 CE1 PHE 24 27.210 32.182 124.811 1.00 0.00

ATOM 5968 HE1 PHE 24 27.847 32.193 123.939 1.00 0.00

ATOM 5969 CZ PHE 24 27.097 31.114 125.709 1.00 0.00

ATOM 5970 HZ PHE 24 27.619 30.172 125.627 1.00 0.00

ATOM 5971 CE2 PHE 24 26.338 31.271 126.875 1.00 0.00

ATOM 5972 HE2 PHE 24 26.153 30.469 127.574 1.00 0.00

ATOM 5973 CD2 PHE 24 25.746 32.514 127.132 1.00 0.00

ATOM 5974 HD2 PHE 24 25.172 32.667 128.034 1.00 0.00

ATOM 5975 C PHE 24 23.087 34.417 125.084 1.00 0.00

ATOM 5976 O PHE 24 23.204 33.609 124.165 1.00 0.00

ATOM 5977 N SER 25 21.949 34.524 125.770 1.00 0.00

ATOM 5978 H SER 25 22.008 35.176 126.540 1.00 0.00

ATOM 5979 CA SER 25 20.820 33.624 125.645 1.00 0.00

ATOM 5980 HA SER 25 21.204 32.610 125.533 1.00 0.00

ATOM 5981 CB SER 25 19.806 33.751 126.779 1.00 0.00

ATOM 5982 HB1 SER 25 18.960 33.067 126.717 1.00 0.00

ATOM 5983 HB2 SER 25 19.498 34.793 126.865 1.00 0.00

ATOM 5984 OG SER 25 20.521 33.469 127.961 1.00 0.00

ATOM 5985 HG SER 25 21.031 34.238 128.225 1.00 0.00

ATOM 5986 C SER 25 20.124 33.854 124.312 1.00 0.00

ATOM 5987 O SER 25 20.016 32.905 123.538 1.00 0.00

ATOM 5988 N ALA 26 19.828 35.092 123.912 1.00 0.00

ATOM 5989 H ALA 26 19.932 35.816 124.608 1.00 0.00

ATOM 5990 CA ALA 26 19.437 35.457 122.563 1.00 0.00

ATOM 5991 HA ALA 26 18.616 34.817 122.241 1.00 0.00

ATOM 5992 CB ALA 26 18.993 36.916 122.493 1.00 0.00

ATOM 5993 HB1 ALA 26 18.694 37.265 121.505 1.00 0.00

ATOM 5994 HB2 ALA 26 18.033 36.933 123.009 1.00 0.00

ATOM 5995 HB3 ALA 26 19.743 37.575 122.931 1.00 0.00

ATOM 5996 C ALA 26 20.428 35.116 121.460 1.00 0.00

ATOM 5997 O ALA 26 20.092 34.657 120.370 1.00 0.00

ATOM 5998 N ALA 27 21.712 35.344 121.740 1.00 0.00

ATOM 5999 H ALA 27 21.948 35.631 122.679 1.00 0.00

ATOM 6000 CA ALA 27 22.812 35.081 120.833 1.00 0.00

ATOM 6001 HA ALA 27 22.654 35.599 119.887 1.00 0.00

ATOM 6002 CB ALA 27 24.106 35.527 121.509 1.00 0.00

ATOM 6003 HB1 ALA 27 24.341 34.916 122.380 1.00 0.00

ATOM 6004 HB2 ALA 27 24.148 36.571 121.816 1.00 0.00

ATOM 6005 HB3 ALA 27 24.953 35.422 120.832 1.00 0.00

ATOM 6006 C ALA 27 22.908 33.600 120.497 1.00 0.00

ATOM 6007 O ALA 27 23.247 33.251 119.369 1.00 0.00

ATOM 6008 N ARG 28 22.522 32.688 121.393 1.00 0.00

ATOM 6009 H ARG 28 22.309 32.930 122.350 1.00 0.00

ATOM 6010 CA ARG 28 22.486 31.290 121.016 1.00 0.00

ATOM 6011 HA ARG 28 23.403 31.053 120.475 1.00 0.00

ATOM 6012 CB ARG 28 22.530 30.408 122.260 1.00 0.00

ATOM 6013 HB1 ARG 28 22.086 29.450 121.990 1.00 0.00

ATOM 6014 HB2 ARG 28 21.833 30.816 122.992 1.00 0.00

ATOM 6015 CG ARG 28 23.928 30.355 122.871 1.00 0.00

ATOM 6016 HG1 ARG 28 24.640 30.266 122.051 1.00 0.00

ATOM 6017 HG2 ARG 28 24.123 31.316 123.347 1.00 0.00

ATOM 6018 CD ARG 28 23.982 29.186 123.851 1.00 0.00

ATOM 6019 HD1 ARG 28 24.837 29.443 124.475 1.00 0.00

ATOM 6020 HD2 ARG 28 23.069 29.109 124.442 1.00 0.00

ATOM 6021 NE ARG 28 24.216 27.927 123.141 1.00 0.00

ATOM 6022 HE ARG 28 24.968 28.003 122.473 1.00 0.00

ATOM 6023 CZ ARG 28 23.597 26.744 123.265 1.00 0.00

ATOM 6024 NH1 ARG 28 22.686 26.610 124.234 1.00 0.00

ATOM 6025 1HH1 ARG 28 22.404 27.440 124.737 1.00 0.00

ATOM 6026 2HH1 ARG 28 22.188 25.738 124.345 1.00 0.00

ATOM 6027 NH2 ARG 28 23.725 25.704 122.431 1.00 0.00

ATOM 6028 1HH2 ARG 28 24.245 25.830 121.573 1.00 0.00

ATOM 6029 2HH2 ARG 28 23.160 24.875 122.556 1.00 0.00

ATOM 6030 C ARG 28 21.311 30.950 120.109 1.00 0.00

ATOM 6031 O ARG 28 21.525 30.142 119.209 1.00 0.00

ATOM 6032 N VAL 29 20.253 31.764 120.103 1.00 0.00

ATOM 6033 H VAL 29 20.120 32.442 120.840 1.00 0.00

ATOM 6034 CA VAL 29 19.142 31.652 119.180 1.00 0.00

ATOM 6035 HA VAL 29 18.961 30.585 119.052 1.00 0.00

ATOM 6036 CB VAL 29 17.809 32.212 119.668 1.00 0.00

ATOM 6037 HB VAL 29 17.733 33.268 119.407 1.00 0.00

ATOM 6038 CG1 VAL 29 16.645 31.453 119.037 1.00 0.00

ATOM 6039 1HG1 VAL 29 16.806 31.419 117.960 1.00 0.00

ATOM 6040 2HG1 VAL 29 15.709 31.934 119.323 1.00 0.00

ATOM 6041 3HG1 VAL 29 16.568 30.400 119.307 1.00 0.00

ATOM 6042 CG2 VAL 29 17.681 32.125 121.186 1.00 0.00

ATOM 6043 1HG2 VAL 29 18.462 32.637 121.748 1.00 0.00

ATOM 6044 2HG2 VAL 29 17.825 31.105 121.541 1.00 0.00

ATOM 6045 3HG2 VAL 29 16.735 32.577 121.484 1.00 0.00

ATOM 6046 C VAL 29 19.506 32.303 117.853 1.00 0.00

ATOM 6047 O VAL 29 19.250 31.720 116.801 1.00 0.00

ATOM 6048 N LYS 30 20.247 33.413 117.864 1.00 0.00

ATOM 6049 H LYS 30 20.424 33.924 118.717 1.00 0.00

ATOM 6050 CA LYS 30 20.939 33.890 116.683 1.00 0.00

ATOM 6051 HA LYS 30 20.145 34.050 115.954 1.00 0.00

ATOM 6052 CB LYS 30 21.737 35.161 116.958 1.00 0.00

ATOM 6053 HB1 LYS 30 22.309 35.274 116.072 1.00 0.00

ATOM 6054 HB2 LYS 30 22.304 35.019 117.879 1.00 0.00

ATOM 6055 CG LYS 30 20.726 36.296 117.087 1.00 0.00

ATOM 6056 HG1 LYS 30 20.229 36.330 116.119 1.00 0.00

ATOM 6057 HG2 LYS 30 19.974 36.107 117.853 1.00 0.00

ATOM 6058 CD LYS 30 21.468 37.607 117.329 1.00 0.00

ATOM 6059 HD1 LYS 30 22.039 37.489 118.250 1.00 0.00

ATOM 6060 HD2 LYS 30 22.188 37.760 116.525 1.00 0.00

ATOM 6061 CE LYS 30 20.471 38.755 117.456 1.00 0.00

ATOM 6062 HE1 LYS 30 19.970 38.684 116.494 1.00 0.00

ATOM 6063 HE2 LYS 30 19.774 38.527 118.261 1.00 0.00

ATOM 6064 NZ LYS 30 21.133 40.047 117.698 1.00 0.00

ATOM 6065 HZ1 LYS 30 21.881 40.007 118.375 1.00 0.00

ATOM 6066 HZ2 LYS 30 20.513 40.763 118.050 1.00 0.00

ATOM 6067 HZ3 LYS 30 21.510 40.372 116.819 1.00 0.00

ATOM 6068 C LYS 30 21.904 32.936 115.996 1.00 0.00

ATOM 6069 O LYS 30 22.030 32.876 114.774 1.00 0.00

ATOM 6070 N ALA 31 22.524 32.066 116.796 1.00 0.00

ATOM 6071 H ALA 31 22.517 32.191 117.798 1.00 0.00

ATOM 6072 CA ALA 31 23.441 31.061 116.297 1.00 0.00

ATOM 6073 HA ALA 31 23.983 31.554 115.492 1.00 0.00

ATOM 6074 CB ALA 31 24.581 30.661 117.231 1.00 0.00

ATOM 6075 HB1 ALA 31 25.056 29.754 116.857 1.00 0.00

ATOM 6076 HB2 ALA 31 25.316 31.465 117.207 1.00 0.00

ATOM 6077 HB3 ALA 31 24.201 30.481 118.237 1.00 0.00

ATOM 6078 C ALA 31 22.680 29.908 115.660 1.00 0.00

ATOM 6079 O ALA 31 22.986 29.541 114.527 1.00 0.00

ATOM 6080 N TYR 32 21.648 29.430 116.359 1.00 0.00

ATOM 6081 H TYR 32 21.343 29.929 117.182 1.00 0.00

ATOM 6082 CA TYR 32 20.861 28.300 115.907 1.00 0.00

ATOM 6083 HA TYR 32 21.549 27.473 115.725 1.00 0.00

ATOM 6084 CB TYR 32 19.861 27.818 116.953 1.00 0.00

ATOM 6085 HB1 TYR 32 19.291 28.664 117.337 1.00 0.00

ATOM 6086 HB2 TYR 32 20.535 27.336 117.661 1.00 0.00

ATOM 6087 CG TYR 32 18.905 26.753 116.473 1.00 0.00

ATOM 6088 CD1 TYR 32 19.416 25.462 116.288 1.00 0.00

ATOM 6089 HD1 TYR 32 20.433 25.250 116.573 1.00 0.00

ATOM 6090 CE1 TYR 32 18.611 24.439 115.774 1.00 0.00

ATOM 6091 HE1 TYR 32 19.036 23.449 115.693 1.00 0.00

ATOM 6092 CZ TYR 32 17.317 24.742 115.304 1.00 0.00

ATOM 6093 OH TYR 32 16.440 23.788 114.879 1.00 0.00

ATOM 6094 HH TYR 32 16.635 22.873 115.095 1.00 0.00

ATOM 6095 CE2 TYR 32 16.844 26.066 115.385 1.00 0.00

ATOM 6096 HE2 TYR 32 15.843 26.399 115.154 1.00 0.00

ATOM 6097 CD2 TYR 32 17.644 27.067 115.951 1.00 0.00

ATOM 6098 HD2 TYR 32 17.208 28.053 116.000 1.00 0.00

ATOM 6099 C TYR 32 20.022 28.625 114.679 1.00 0.00

ATOM 6100 O TYR 32 19.973 27.848 113.729 1.00 0.00

ATOM 6101 N ASN 33 19.412 29.812 114.704 1.00 0.00

ATOM 6102 H ASN 33 19.531 30.347 115.552 1.00 0.00

ATOM 6103 CA ASN 33 18.696 30.466 113.626 1.00 0.00

ATOM 6104 HA ASN 33 17.859 29.862 113.275 1.00 0.00

ATOM 6105 CB ASN 33 18.267 31.869 114.049 1.00 0.00

ATOM 6106 HB1 ASN 33 18.974 32.415 114.673 1.00 0.00

ATOM 6107 HB2 ASN 33 18.268 32.595 113.238 1.00 0.00

ATOM 6108 CG ASN 33 16.816 31.838 114.511 1.00 0.00

ATOM 6109 OD1 ASN 33 16.306 30.787 114.892 1.00 0.00

ATOM 6110 ND2 ASN 33 16.098 32.957 114.436 1.00 0.00

ATOM 6111 1HD2 ASN 33 16.553 33.833 114.212 1.00 0.00

ATOM 6112 2HD2 ASN 33 15.218 32.928 114.932 1.00 0.00

ATOM 6113 C ASN 33 19.524 30.616 112.359 1.00 0.00

ATOM 6114 O ASN 33 19.050 30.598 111.226 1.00 0.00

ATOM 6115 N SER 34 20.811 30.937 112.514 1.00 0.00

ATOM 6116 H SER 34 21.171 31.141 113.435 1.00 0.00

ATOM 6117 CA SER 34 21.836 31.051 111.495 1.00 0.00

ATOM 6118 HA SER 34 21.467 31.536 110.592 1.00 0.00

ATOM 6119 CB SER 34 23.086 31.699 112.086 1.00 0.00

ATOM 6120 HB1 SER 34 23.929 31.715 111.394 1.00 0.00

ATOM 6121 HB2 SER 34 23.545 31.232 112.957 1.00 0.00

ATOM 6122 OG SER 34 22.643 33.007 112.369 1.00 0.00

ATOM 6123 HG SER 34 22.555 33.043 113.324 1.00 0.00

ATOM 6124 C SER 34 22.187 29.710 110.871 1.00 0.00

ATOM 6125 O SER 34 22.136 29.582 109.650 1.00 0.00

ATOM 6126 N ALA 35 22.514 28.689 111.667 1.00 0.00

ATOM 6127 H ALA 35 22.258 28.738 112.643 1.00 0.00

ATOM 6128 CA ALA 35 22.706 27.369 111.100 1.00 0.00

ATOM 6129 HA ALA 35 23.534 27.399 110.394 1.00 0.00

ATOM 6130 CB ALA 35 23.168 26.528 112.285 1.00 0.00

ATOM 6131 HB1 ALA 35 23.254 25.510 111.909 1.00 0.00

ATOM 6132 HB2 ALA 35 24.110 26.888 112.699 1.00 0.00

ATOM 6133 HB3 ALA 35 22.411 26.499 113.071 1.00 0.00

ATOM 6134 C ALA 35 21.542 26.752 110.336 1.00 0.00

ATOM 6135 O ALA 35 21.801 26.221 109.258 1.00 0.00

ATOM 6136 N ALA 36 20.328 27.072 110.788 1.00 0.00

ATOM 6137 H ALA 36 20.234 27.359 111.753 1.00 0.00

ATOM 6138 CA ALA 36 19.134 26.727 110.043 1.00 0.00

ATOM 6139 HA ALA 36 19.248 25.731 109.617 1.00 0.00

ATOM 6140 CB ALA 36 18.035 26.575 111.089 1.00 0.00

ATOM 6141 HB1 ALA 36 18.396 25.936 111.895 1.00 0.00

ATOM 6142 HB2 ALA 36 17.090 26.261 110.645 1.00 0.00

ATOM 6143 HB3 ALA 36 17.921 27.567 111.525 1.00 0.00

ATOM 6144 C ALA 36 18.742 27.596 108.856 1.00 0.00

ATOM 6145 O ALA 36 17.600 27.598 108.401 1.00 0.00

ATOM 6146 N SER 37 19.688 28.394 108.356 1.00 0.00

ATOM 6147 H SER 37 20.668 28.336 108.595 1.00 0.00

ATOM 6148 CA SER 37 19.366 29.236 107.222 1.00 0.00

ATOM 6149 HA SER 37 18.672 28.750 106.535 1.00 0.00

ATOM 6150 CB SER 37 18.705 30.444 107.879 1.00 0.00

ATOM 6151 HB1 SER 37 18.215 31.084 107.146 1.00 0.00

ATOM 6152 HB2 SER 37 17.888 30.096 108.510 1.00 0.00

ATOM 6153 OG SER 37 19.528 31.248 108.694 1.00 0.00

ATOM 6154 HG SER 37 19.172 31.087 109.572 1.00 0.00

ATOM 6155 C SER 37 20.458 29.765 106.304 1.00 0.00

ATOM 6156 O SER 37 20.062 30.117 105.195 1.00 0.00

ATOM 6157 N SER 38 21.745 29.737 106.660 1.00 0.00

ATOM 6158 H SER 38 21.903 29.365 107.584 1.00 0.00

ATOM 6159 CA SER 38 22.818 30.527 106.086 1.00 0.00

ATOM 6160 HA SER 38 22.483 31.527 105.818 1.00 0.00

ATOM 6161 CB SER 38 23.994 30.823 107.013 1.00 0.00

ATOM 6162 HB1 SER 38 24.723 31.405 106.448 1.00 0.00

ATOM 6163 HB2 SER 38 24.331 29.886 107.455 1.00 0.00

ATOM 6164 OG SER 38 23.586 31.622 108.099 1.00 0.00

ATOM 6165 HG SER 38 22.883 31.197 108.594 1.00 0.00

ATOM 6166 C SER 38 23.271 29.966 104.745 1.00 0.00

ATOM 6167 O SER 38 23.393 30.705 103.772 1.00 0.00

ATOM 6168 N ASP 39 23.297 28.642 104.577 1.00 0.00

ATOM 6169 H ASP 39 23.131 28.071 105.393 1.00 0.00

ATOM 6170 CA ASP 39 23.470 27.937 103.322 1.00 0.00

ATOM 6171 HA ASP 39 24.338 28.433 102.889 1.00 0.00

ATOM 6172 CB ASP 39 23.803 26.464 103.544 1.00 0.00

ATOM 6173 HB1 ASP 39 24.822 26.329 103.881 1.00 0.00

ATOM 6174 HB2 ASP 39 23.143 26.046 104.304 1.00 0.00

ATOM 6175 CG ASP 39 23.855 25.710 102.222 1.00 0.00

ATOM 6176 OD1 ASP 39 24.878 25.807 101.510 1.00 0.00

ATOM 6177 OD2 ASP 39 22.880 25.005 101.887 1.00 0.00

ATOM 6178 C ASP 39 22.302 28.113 102.362 1.00 0.00

ATOM 6179 O ASP 39 22.468 28.468 101.197 1.00 0.00

ATOM 6180 N LEU 40 21.073 27.950 102.855 1.00 0.00

ATOM 6181 H LEU 40 21.022 27.672 103.825 1.00 0.00

ATOM 6182 CA LEU 40 19.874 28.036 102.046 1.00 0.00

ATOM 6183 HA LEU 40 19.963 27.363 101.193 1.00 0.00

ATOM 6184 CB LEU 40 18.717 27.516 102.894 1.00 0.00

ATOM 6185 HB1 LEU 40 18.489 28.164 103.740 1.00 0.00

ATOM 6186 HB2 LEU 40 18.924 26.497 103.220 1.00 0.00

ATOM 6187 CG LEU 40 17.526 27.368 101.950 1.00 0.00

ATOM 6188 HG LEU 40 17.219 28.311 101.498 1.00 0.00

ATOM 6189 CD1 LEU 40 17.621 26.375 100.794 1.00 0.00

ATOM 6190 1HD1 LEU 40 18.449 26.565 100.113 1.00 0.00

ATOM 6191 2HD1 LEU 40 16.828 26.567 100.072 1.00 0.00

ATOM 6192 3HD1 LEU 40 17.590 25.333 101.112 1.00 0.00

ATOM 6193 CD2 LEU 40 16.314 26.797 102.678 1.00 0.00

ATOM 6194 1HD2 LEU 40 16.625 25.878 103.173 1.00 0.00

ATOM 6195 2HD2 LEU 40 15.562 26.677 101.901 1.00 0.00

ATOM 6196 3HD2 LEU 40 16.061 27.450 103.512 1.00 0.00

ATOM 6197 C LEU 40 19.709 29.425 101.444 1.00 0.00

ATOM 6198 O LEU 40 19.321 29.596 100.291 1.00 0.00

ATOM 6199 N ARG 41 20.160 30.446 102.176 1.00 0.00

ATOM 6200 H ARG 41 20.492 30.232 103.106 1.00 0.00

ATOM 6201 CA ARG 41 20.194 31.841 101.784 1.00 0.00

ATOM 6202 HA ARG 41 19.195 32.094 101.429 1.00 0.00

ATOM 6203 CB ARG 41 20.589 32.784 102.916 1.00 0.00

ATOM 6204 HB1 ARG 41 21.670 32.661 102.973 1.00 0.00

ATOM 6205 HB2 ARG 41 20.109 32.403 103.817 1.00 0.00

ATOM 6206 CG ARG 41 20.167 34.244 102.763 1.00 0.00

ATOM 6207 HG1 ARG 41 20.574 34.735 103.647 1.00 0.00

ATOM 6208 HG2 ARG 41 20.505 34.641 101.806 1.00 0.00

ATOM 6209 CD ARG 41 18.669 34.434 102.983 1.00 0.00

ATOM 6210 HD1 ARG 41 18.137 33.486 102.911 1.00 0.00

ATOM 6211 HD2 ARG 41 18.583 34.718 104.028 1.00 0.00

ATOM 6212 NE ARG 41 18.059 35.506 102.193 1.00 0.00

ATOM 6213 HE ARG 41 18.428 36.424 102.393 1.00 0.00

ATOM 6214 CZ ARG 41 17.063 35.508 101.297 1.00 0.00

ATOM 6215 NH1 ARG 41 16.414 34.391 100.947 1.00 0.00

ATOM 6216 1HH1 ARG 41 16.262 33.723 101.689 1.00 0.00

ATOM 6217 2HH1 ARG 41 15.849 34.390 100.108 1.00 0.00

ATOM 6218 NH2 ARG 41 16.766 36.650 100.666 1.00 0.00

ATOM 6219 1HH2 ARG 41 17.167 37.552 100.884 1.00 0.00

ATOM 6220 2HH2 ARG 41 15.999 36.592 100.011 1.00 0.00

ATOM 6221 C ARG 41 21.079 32.055 100.565 1.00 0.00

ATOM 6222 O ARG 41 20.787 32.864 99.687 1.00 0.00

ATOM 6223 N ASN 42 22.185 31.318 100.438 1.00 0.00

ATOM 6224 H ASN 42 22.326 30.525 101.048 1.00 0.00

ATOM 6225 CA ASN 42 23.094 31.451 99.317 1.00 0.00

ATOM 6226 HA ASN 42 23.363 32.492 99.141 1.00 0.00

ATOM 6227 CB ASN 42 24.375 30.752 99.759 1.00 0.00

ATOM 6228 HB1 ASN 42 24.822 31.376 100.534 1.00 0.00

ATOM 6229 HB2 ASN 42 24.213 29.767 100.195 1.00 0.00

ATOM 6230 CG ASN 42 25.418 30.524 98.673 1.00 0.00

ATOM 6231 OD1 ASN 42 26.199 31.366 98.245 1.00 0.00

ATOM 6232 ND2 ASN 42 25.611 29.294 98.197 1.00 0.00

ATOM 6233 1HD2 ASN 42 25.029 28.550 98.557 1.00 0.00

ATOM 6234 2HD2 ASN 42 26.315 29.230 97.475 1.00 0.00

ATOM 6235 C ASN 42 22.580 30.852 98.015 1.00 0.00

ATOM 6236 O ASN 42 22.962 31.303 96.939 1.00 0.00

ATOM 6237 N LEU 43 21.551 30.020 98.184 1.00 0.00

ATOM 6238 H LEU 43 21.556 29.614 99.108 1.00 0.00

ATOM 6239 CA LEU 43 20.795 29.514 97.054 1.00 0.00

ATOM 6240 HA LEU 43 21.498 29.437 96.226 1.00 0.00

ATOM 6241 CB LEU 43 20.307 28.086 97.282 1.00 0.00

ATOM 6242 HB1 LEU 43 19.569 28.132 98.082 1.00 0.00

ATOM 6243 HB2 LEU 43 21.221 27.559 97.557 1.00 0.00

ATOM 6244 CG LEU 43 19.684 27.297 96.133 1.00 0.00

ATOM 6245 HG LEU 43 19.010 27.982 95.618 1.00 0.00

ATOM 6246 CD1 LEU 43 20.702 26.943 95.054 1.00 0.00

ATOM 6247 1HD1 LEU 43 21.457 26.331 95.547 1.00 0.00

ATOM 6248 2HD1 LEU 43 21.035 27.910 94.677 1.00 0.00

ATOM 6249 3HD1 LEU 43 20.184 26.434 94.241 1.00 0.00

ATOM 6250 CD2 LEU 43 19.095 25.979 96.626 1.00 0.00

ATOM 6251 1HD2 LEU 43 18.612 25.333 95.893 1.00 0.00

ATOM 6252 2HD2 LEU 43 18.419 26.295 97.420 1.00 0.00

ATOM 6253 3HD2 LEU 43 19.978 25.488 97.034 1.00 0.00

ATOM 6254 C LEU 43 19.674 30.444 96.614 1.00 0.00

ATOM 6255 O LEU 43 19.715 30.941 95.490 1.00 0.00

ATOM 6256 N LYS 44 18.754 30.705 97.546 1.00 0.00

ATOM 6257 H LYS 44 18.971 30.393 98.482 1.00 0.00

ATOM 6258 CA LYS 44 17.483 31.354 97.288 1.00 0.00

ATOM 6259 HA LYS 44 16.960 30.905 96.444 1.00 0.00

ATOM 6260 CB LYS 44 16.534 31.296 98.482 1.00 0.00

ATOM 6261 HB1 LYS 44 16.801 32.046 99.225 1.00 0.00

ATOM 6262 HB2 LYS 44 16.839 30.409 99.036 1.00 0.00

ATOM 6263 CG LYS 44 15.065 31.250 98.068 1.00 0.00

ATOM 6264 HG1 LYS 44 14.926 32.114 97.417 1.00 0.00

ATOM 6265 HG2 LYS 44 14.899 30.311 97.541 1.00 0.00

ATOM 6266 CD LYS 44 14.115 31.369 99.257 1.00 0.00

ATOM 6267 HD1 LYS 44 14.147 30.387 99.729 1.00 0.00

ATOM 6268 HD2 LYS 44 14.507 32.203 99.838 1.00 0.00

ATOM 6269 CE LYS 44 12.665 31.645 98.871 1.00 0.00

ATOM 6270 HE1 LYS 44 12.591 32.407 98.095 1.00 0.00

ATOM 6271 HE2 LYS 44 12.342 30.695 98.446 1.00 0.00

ATOM 6272 NZ LYS 44 11.806 32.001 100.010 1.00 0.00

ATOM 6273 HZ1 LYS 44 10.824 32.134 99.814 1.00 0.00

ATOM 6274 HZ2 LYS 44 12.111 32.798 100.552 1.00 0.00

ATOM 6275 HZ3 LYS 44 11.883 31.187 100.602 1.00 0.00

ATOM 6276 C LYS 44 17.554 32.784 96.776 1.00 0.00

ATOM 6277 O LYS 44 16.832 33.124 95.842 1.00 0.00

ATOM 6278 N THR 45 18.550 33.561 97.207 1.00 0.00

ATOM 6279 H THR 45 19.158 33.297 97.969 1.00 0.00

ATOM 6280 CA THR 45 18.811 34.824 96.543 1.00 0.00

ATOM 6281 HA THR 45 17.915 35.394 96.786 1.00 0.00

ATOM 6282 CB THR 45 19.959 35.560 97.227 1.00 0.00

ATOM 6283 HB THR 45 19.675 35.714 98.268 1.00 0.00

ATOM 6284 CG2 THR 45 21.297 34.828 97.183 1.00 0.00

ATOM 6285 1HG2 THR 45 21.132 33.751 97.149 1.00 0.00

ATOM 6286 2HG2 THR 45 21.858 35.049 98.091 1.00 0.00

ATOM 6287 3HG2 THR 45 21.880 35.165 96.326 1.00 0.00

ATOM 6288 OG1 THR 45 20.210 36.797 96.602 1.00 0.00

ATOM 6289 HG1 THR 45 21.137 37.026 96.696 1.00 0.00

ATOM 6290 C THR 45 18.888 34.866 95.024 1.00 0.00

ATOM 6291 O THR 45 18.232 35.685 94.384 1.00 0.00

ATOM 6292 N ALA 46 19.710 34.038 94.377 1.00 0.00

ATOM 6293 H ALA 46 20.266 33.418 94.947 1.00 0.00

ATOM 6294 CA ALA 46 19.712 33.874 92.936 1.00 0.00

ATOM 6295 HA ALA 46 19.958 34.868 92.564 1.00 0.00

ATOM 6296 CB ALA 46 20.912 33.020 92.540 1.00 0.00

ATOM 6297 HB1 ALA 46 21.765 33.696 92.485 1.00 0.00

ATOM 6298 HB2 ALA 46 20.858 32.472 91.599 1.00 0.00

ATOM 6299 HB3 ALA 46 21.103 32.297 93.333 1.00 0.00

ATOM 6300 C ALA 46 18.410 33.434 92.281 1.00 0.00

ATOM 6301 O ALA 46 18.129 33.866 91.165 1.00 0.00

ATOM 6302 N LEU 47 17.567 32.768 93.072 1.00 0.00

ATOM 6303 H LEU 47 17.833 32.611 94.035 1.00 0.00

ATOM 6304 CA LEU 47 16.246 32.470 92.556 1.00 0.00

ATOM 6305 HA LEU 47 16.312 32.166 91.511 1.00 0.00

ATOM 6306 CB LEU 47 15.596 31.306 93.299 1.00 0.00

ATOM 6307 HB1 LEU 47 15.430 31.609 94.332 1.00 0.00

ATOM 6308 HB2 LEU 47 16.242 30.433 93.387 1.00 0.00

ATOM 6309 CG LEU 47 14.267 30.832 92.716 1.00 0.00

ATOM 6310 HG LEU 47 13.652 31.683 92.423 1.00 0.00

ATOM 6311 CD1 LEU 47 14.519 30.140 91.380 1.00 0.00

ATOM 6312 1HD1 LEU 47 15.191 29.289 91.490 1.00 0.00

ATOM 6313 2HD1 LEU 47 14.886 30.780 90.576 1.00 0.00

ATOM 6314 3HD1 LEU 47 13.578 29.712 91.037 1.00 0.00

ATOM 6315 CD2 LEU 47 13.553 29.869 93.660 1.00 0.00

ATOM 6316 1HD2 LEU 47 12.576 29.617 93.244 1.00 0.00

ATOM 6317 2HD2 LEU 47 13.543 30.428 94.596 1.00 0.00

ATOM 6318 3HD2 LEU 47 14.096 28.942 93.839 1.00 0.00

ATOM 6319 C LEU 47 15.357 33.705 92.605 1.00 0.00

ATOM 6320 O LEU 47 14.944 34.153 91.538 1.00 0.00

ATOM 6321 N GLU 48 15.370 34.409 93.738 1.00 0.00

ATOM 6322 H GLU 48 15.950 34.030 94.474 1.00 0.00

ATOM 6323 CA GLU 48 14.704 35.660 94.038 1.00 0.00

ATOM 6324 HA GLU 48 13.648 35.419 93.937 1.00 0.00

ATOM 6325 CB GLU 48 14.970 35.997 95.503 1.00 0.00

ATOM 6326 HB1 GLU 48 14.844 37.079 95.499 1.00 0.00

ATOM 6327 HB2 GLU 48 16.012 35.785 95.741 1.00 0.00

ATOM 6328 CG GLU 48 14.222 35.233 96.594 1.00 0.00

ATOM 6329 HG1 GLU 48 13.165 35.469 96.478 1.00 0.00

ATOM 6330 HG2 GLU 48 14.332 34.153 96.498 1.00 0.00

ATOM 6331 CD GLU 48 14.629 35.639 98.003 1.00 0.00

ATOM 6332 OE1 GLU 48 14.733 34.733 98.858 1.00 0.00

ATOM 6333 OE2 GLU 48 14.818 36.825 98.347 1.00 0.00

ATOM 6334 C GLU 48 14.988 36.784 93.051 1.00 0.00

ATOM 6335 O GLU 48 14.023 37.297 92.489 1.00 0.00

ATOM 6336 N SER 49 16.285 36.933 92.770 1.00 0.00

ATOM 6337 H SER 49 16.945 36.381 93.299 1.00 0.00

ATOM 6338 CA SER 49 16.802 38.053 92.010 1.00 0.00

ATOM 6339 HA SER 49 16.278 38.947 92.347 1.00 0.00

ATOM 6340 CB SER 49 18.281 38.413 92.139 1.00 0.00

ATOM 6341 HB1 SER 49 18.621 39.080 91.350 1.00 0.00

ATOM 6342 HB2 SER 49 18.848 37.544 91.809 1.00 0.00

ATOM 6343 OG SER 49 18.742 38.873 93.389 1.00 0.00

ATOM 6344 HG SER 49 18.142 38.500 94.038 1.00 0.00

ATOM 6345 C SER 49 16.359 37.910 90.561 1.00 0.00

ATOM 6346 O SER 49 15.957 38.894 89.943 1.00 0.00

ATOM 6347 N ALA 50 16.564 36.708 90.019 1.00 0.00

ATOM 6348 H ALA 50 16.943 35.938 90.551 1.00 0.00

ATOM 6349 CA ALA 50 16.228 36.444 88.634 1.00 0.00

ATOM 6350 HA ALA 50 16.765 37.167 88.019 1.00 0.00

ATOM 6351 CB ALA 50 16.694 35.036 88.277 1.00 0.00

ATOM 6352 HB1 ALA 50 16.432 34.934 87.224 1.00 0.00

ATOM 6353 HB2 ALA 50 17.750 34.873 88.425 1.00 0.00

ATOM 6354 HB3 ALA 50 16.071 34.338 88.836 1.00 0.00

ATOM 6355 C ALA 50 14.748 36.627 88.329 1.00 0.00

ATOM 6356 O ALA 50 14.488 37.130 87.240 1.00 0.00

ATOM 6357 N PHE 51 13.801 36.295 89.210 1.00 0.00

ATOM 6358 H PHE 51 14.005 35.637 89.949 1.00 0.00

ATOM 6359 CA PHE 51 12.419 36.720 89.125 1.00 0.00

ATOM 6360 HA PHE 51 12.082 36.457 88.122 1.00 0.00

ATOM 6361 CB PHE 51 11.650 36.011 90.237 1.00 0.00

ATOM 6362 HB1 PHE 51 10.628 36.380 90.168 1.00 0.00

ATOM 6363 HB2 PHE 51 12.023 36.171 91.250 1.00 0.00

ATOM 6364 CG PHE 51 11.316 34.546 90.089 1.00 0.00

ATOM 6365 CD1 PHE 51 10.872 34.109 88.836 1.00 0.00

ATOM 6366 HD1 PHE 51 10.823 34.801 88.008 1.00 0.00

ATOM 6367 CE1 PHE 51 10.704 32.732 88.645 1.00 0.00

ATOM 6368 HE1 PHE 51 10.392 32.399 87.666 1.00 0.00

ATOM 6369 CZ PHE 51 10.909 31.838 89.703 1.00 0.00

ATOM 6370 HZ PHE 51 10.794 30.775 89.547 1.00 0.00

ATOM 6371 CE2 PHE 51 11.399 32.286 90.935 1.00 0.00

ATOM 6372 HE2 PHE 51 11.604 31.587 91.733 1.00 0.00

ATOM 6373 CD2 PHE 51 11.557 33.660 91.146 1.00 0.00

ATOM 6374 HD2 PHE 51 11.888 34.001 92.116 1.00 0.00

ATOM 6375 C PHE 51 12.332 38.239 89.180 1.00 0.00

ATOM 6376 O PHE 51 11.663 38.879 88.373 1.00 0.00

ATOM 6377 N ALA 52 13.022 38.898 90.114 1.00 0.00

ATOM 6378 H ALA 52 13.595 38.382 90.766 1.00 0.00

ATOM 6379 CA ALA 52 12.898 40.325 90.331 1.00 0.00

ATOM 6380 HA ALA 52 11.815 40.377 90.444 1.00 0.00

ATOM 6381 CB ALA 52 13.485 40.604 91.711 1.00 0.00

ATOM 6382 HB1 ALA 52 14.543 40.346 91.772 1.00 0.00

ATOM 6383 HB2 ALA 52 13.365 41.640 92.027 1.00 0.00

ATOM 6384 HB3 ALA 52 13.010 39.840 92.326 1.00 0.00

ATOM 6385 C ALA 52 13.475 41.233 89.254 1.00 0.00

ATOM 6386 O ALA 52 13.261 42.443 89.255 1.00 0.00

ATOM 6387 N ASP 53 14.139 40.600 88.284 1.00 0.00

ATOM 6388 H ASP 53 14.528 39.686 88.465 1.00 0.00

ATOM 6389 CA ASP 53 14.535 41.222 87.036 1.00 0.00

ATOM 6390 HA ASP 53 14.461 42.309 87.032 1.00 0.00

ATOM 6391 CB ASP 53 15.915 40.660 86.710 1.00 0.00

ATOM 6392 HB1 ASP 53 16.530 40.923 87.570 1.00 0.00

ATOM 6393 HB2 ASP 53 15.985 39.590 86.511 1.00 0.00

ATOM 6394 CG ASP 53 16.483 41.341 85.473 1.00 0.00

ATOM 6395 OD1 ASP 53 16.375 40.812 84.345 1.00 0.00

ATOM 6396 OD2 ASP 53 17.126 42.390 85.695 1.00 0.00

ATOM 6397 C ASP 53 13.594 40.822 85.909 1.00 0.00

ATOM 6398 O ASP 53 13.182 41.667 85.117 1.00 0.00

ATOM 6399 N ASP 54 13.249 39.532 85.921 1.00 0.00

ATOM 6400 H ASP 54 13.510 39.008 86.745 1.00 0.00

ATOM 6401 CA ASP 54 12.297 38.985 84.978 1.00 0.00

ATOM 6402 HA ASP 54 12.712 39.288 84.017 1.00 0.00

ATOM 6403 CB ASP 54 12.325 37.460 84.918 1.00 0.00

ATOM 6404 HB1 ASP 54 13.278 36.997 84.682 1.00 0.00

ATOM 6405 HB2 ASP 54 12.097 37.082 85.915 1.00 0.00

ATOM 6406 CG ASP 54 11.327 36.891 83.919 1.00 0.00

ATOM 6407 OD1 ASP 54 11.546 36.908 82.688 1.00 0.00

ATOM 6408 OD2 ASP 54 10.296 36.357 84.380 1.00 0.00

ATOM 6409 C ASP 54 10.935 39.668 84.965 1.00 0.00

ATOM 6410 O ASP 54 10.452 39.894 83.860 1.00 0.00

ATOM 6411 N GLN 55 10.293 40.083 86.061 1.00 0.00

ATOM 6412 H GLN 55 10.761 39.807 86.911 1.00 0.00

ATOM 6413 CA GLN 55 9.132 40.938 86.214 1.00 0.00

ATOM 6414 HA GLN 55 8.344 40.415 85.675 1.00 0.00

ATOM 6415 CB GLN 55 8.683 40.986 87.672 1.00 0.00

ATOM 6416 HB1 GLN 55 8.486 40.024 88.139 1.00 0.00

ATOM 6417 HB2 GLN 55 7.715 41.482 87.737 1.00 0.00

ATOM 6418 CG GLN 55 9.620 41.835 88.528 1.00 0.00

ATOM 6419 HG1 GLN 55 10.668 41.618 88.323 1.00 0.00

ATOM 6420 HG2 GLN 55 9.505 42.884 88.257 1.00 0.00

ATOM 6421 CD GLN 55 9.594 41.759 90.048 1.00 0.00

ATOM 6422 OE1 GLN 55 9.237 40.762 90.658 1.00 0.00

ATOM 6423 NE2 GLN 55 10.013 42.813 90.751 1.00 0.00

ATOM 6424 1HE2 GLN 55 10.265 43.704 90.349 1.00 0.00

ATOM 6425 2HE2 GLN 55 9.986 42.704 91.755 1.00 0.00

ATOM 6426 C GLN 55 9.192 42.348 85.641 1.00 0.00

ATOM 6427 O GLN 55 8.178 43.042 85.604 1.00 0.00

ATOM 6428 N THR 56 10.358 42.861 85.240 1.00 0.00

ATOM 6429 H THR 56 11.107 42.215 85.039 1.00 0.00

ATOM 6430 CA THR 56 10.681 44.233 84.898 1.00 0.00

ATOM 6431 HA THR 56 11.769 44.230 84.835 1.00 0.00

ATOM 6432 CB THR 56 10.077 44.794 83.614 1.00 0.00

ATOM 6433 HB THR 56 10.298 45.859 83.548 1.00 0.00

ATOM 6434 CG2 THR 56 10.675 44.225 82.330 1.00 0.00

ATOM 6435 1HG2 THR 56 10.350 43.191 82.210 1.00 0.00

ATOM 6436 2HG2 THR 56 11.764 44.259 82.346 1.00 0.00

ATOM 6437 3HG2 THR 56 10.221 44.766 81.500 1.00 0.00

ATOM 6438 OG1 THR 56 8.670 44.753 83.544 1.00 0.00

ATOM 6439 HG1 THR 56 8.389 44.163 84.247 1.00 0.00

ATOM 6440 C THR 56 10.508 45.251 86.016 1.00 0.00

ATOM 6441 O THR 56 11.430 45.995 86.341 1.00 0.00

ATOM 6442 N TYR 57 9.386 45.209 86.738 1.00 0.00

ATOM 6443 H TYR 57 8.657 44.566 86.465 1.00 0.00

ATOM 6444 CA TYR 57 9.048 46.096 87.833 1.00 0.00

ATOM 6445 HA TYR 57 8.886 47.078 87.388 1.00 0.00

ATOM 6446 CB TYR 57 7.767 45.540 88.450 1.00 0.00

ATOM 6447 HB1 TYR 57 7.945 44.577 88.927 1.00 0.00

ATOM 6448 HB2 TYR 57 7.030 45.391 87.661 1.00 0.00

ATOM 6449 CG TYR 57 7.209 46.457 89.511 1.00 0.00

ATOM 6450 CD1 TYR 57 6.538 47.628 89.137 1.00 0.00

ATOM 6451 HD1 TYR 57 6.426 47.791 88.076 1.00 0.00

ATOM 6452 CE1 TYR 57 6.303 48.651 90.065 1.00 0.00

ATOM 6453 HE1 TYR 57 5.896 49.590 89.725 1.00 0.00

ATOM 6454 CZ TYR 57 6.509 48.416 91.440 1.00 0.00

ATOM 6455 OH TYR 57 6.229 49.317 92.425 1.00 0.00

ATOM 6456 HH TYR 57 6.578 49.039 93.276 1.00 0.00

ATOM 6457 CE2 TYR 57 6.944 47.143 91.859 1.00 0.00

ATOM 6458 HE2 TYR 57 7.006 46.850 92.896 1.00 0.00

ATOM 6459 CD2 TYR 57 7.363 46.228 90.884 1.00 0.00

ATOM 6460 HD2 TYR 57 7.677 45.230 91.148 1.00 0.00

ATOM 6461 C TYR 57 10.104 46.132 88.930 1.00 0.00

ATOM 6462 O TYR 57 10.774 45.137 89.198 1.00 0.00

ATOM 6463 N PRO 58 10.295 47.244 89.643 1.00 0.00

ATOM 6464 CD PRO 58 9.832 48.543 89.196 1.00 0.00

ATOM 6465 HD1 PRO 58 8.765 48.673 89.380 1.00 0.00

ATOM 6466 HD2 PRO 58 9.955 48.672 88.121 1.00 0.00

ATOM 6467 CG PRO 58 10.546 49.623 90.004 1.00 0.00

ATOM 6468 HG1 PRO 58 9.812 50.302 90.437 1.00 0.00

ATOM 6469 HG2 PRO 58 11.332 50.140 89.461 1.00 0.00

ATOM 6470 CB PRO 58 11.177 48.828 91.143 1.00 0.00

ATOM 6471 HB1 PRO 58 10.544 48.775 92.029 1.00 0.00

ATOM 6472 HB2 PRO 58 12.138 49.289 91.369 1.00 0.00

ATOM 6473 CA PRO 58 11.340 47.399 90.633 1.00 0.00

ATOM 6474 HA PRO 58 12.269 47.273 90.077 1.00 0.00

ATOM 6475 C PRO 58 11.386 46.300 91.685 1.00 0.00

ATOM 6476 O PRO 58 10.392 45.678 92.054 1.00 0.00

ATOM 6477 N PRO 59 12.603 45.961 92.118 1.00 0.00

ATOM 6478 CD PRO 59 13.861 46.548 91.702 1.00 0.00

ATOM 6479 HD1 PRO 59 13.773 47.631 91.785 1.00 0.00

ATOM 6480 HD2 PRO 59 14.118 46.284 90.676 1.00 0.00

ATOM 6481 CG PRO 59 14.938 45.970 92.615 1.00 0.00

ATOM 6482 HG1 PRO 59 14.992 46.631 93.451 1.00 0.00

ATOM 6483 HG2 PRO 59 15.901 45.790 92.137 1.00 0.00

ATOM 6484 CB PRO 59 14.339 44.637 93.055 1.00 0.00

ATOM 6485 HB1 PRO 59 14.670 44.525 94.086 1.00 0.00

ATOM 6486 HB2 PRO 59 14.625 43.779 92.445 1.00 0.00

ATOM 6487 CA PRO 59 12.830 44.863 93.035 1.00 0.00

ATOM 6488 HA PRO 59 12.410 43.976 92.560 1.00 0.00

ATOM 6489 C PRO 59 12.235 45.045 94.425 1.00 0.00

ATOM 6490 O PRO 59 11.667 44.060 94.890 1.00 0.00

ATOM 6491 N GLU 60 12.215 46.230 95.039 1.00 0.00

ATOM 6492 H GLU 60 12.907 46.922 94.787 1.00 0.00

ATOM 6493 CA GLU 60 11.562 46.497 96.304 1.00 0.00

ATOM 6494 HA GLU 60 11.868 47.509 96.570 1.00 0.00

ATOM 6495 CB GLU 60 10.037 46.539 96.263 1.00 0.00

ATOM 6496 HB1 GLU 60 9.663 46.894 97.223 1.00 0.00

ATOM 6497 HB2 GLU 60 9.638 45.526 96.225 1.00 0.00

ATOM 6498 CG GLU 60 9.432 47.468 95.215 1.00 0.00

ATOM 6499 HG1 GLU 60 10.066 48.347 95.101 1.00 0.00

ATOM 6500 HG2 GLU 60 9.528 46.929 94.272 1.00 0.00

ATOM 6501 CD GLU 60 7.981 47.811 95.520 1.00 0.00

ATOM 6502 OE1 GLU 60 7.386 47.090 96.350 1.00 0.00

ATOM 6503 OE2 GLU 60 7.454 48.754 94.893 1.00 0.00

ATOM 6504 C GLU 60 12.035 45.654 97.480 1.00 0.00

ATOM 6505 O GLU 60 11.514 45.896 98.567 1.00 0.00

ATOM 6506 N SER 61 12.935 44.684 97.309 1.00 0.00

ATOM 6507 H SER 61 13.313 44.534 96.384 1.00 0.00

ATOM 6508 CA SER 61 13.195 43.646 98.286 1.00 0.00

ATOM 6509 HA SER 61 12.299 43.096 98.576 1.00 0.00

ATOM 6510 CB SER 61 14.179 42.673 97.644 1.00 0.00

ATOM 6511 HB1 SER 61 15.165 43.132 97.582 1.00 0.00

ATOM 6512 HB2 SER 61 14.349 41.791 98.260 1.00 0.00

ATOM 6513 OG SER 61 13.925 42.185 96.346 1.00 0.00

ATOM 6514 HG SER 61 14.678 41.676 96.038 1.00 0.00

ATOM 6515 C SER 61 13.843 44.131 99.574 1.00 0.00

ATOM 6516 OC1 SER 61 15.072 44.369 99.581 1.00 0.00

ATOM 6517 OC2 SER 61 13.025 44.180 100.517 1.00 0.00

ATOM 6518 N PHE 1 31.825 39.732 164.574 1.00 0.00

ATOM 6519 H1 PHE 1 31.772 40.402 163.821 1.00 0.00

ATOM 6520 H2 PHE 1 31.028 39.846 165.184 1.00 0.00

ATOM 6521 H3 PHE 1 31.799 38.825 164.131 1.00 0.00

ATOM 6522 CA PHE 1 33.138 39.797 165.236 1.00 0.00

ATOM 6523 HA PHE 1 33.830 39.553 164.430 1.00 0.00

ATOM 6524 CB PHE 1 33.554 41.180 165.730 1.00 0.00

ATOM 6525 HB1 PHE 1 32.982 41.577 166.569 1.00 0.00

ATOM 6526 HB2 PHE 1 33.378 41.928 164.958 1.00 0.00

ATOM 6527 CG PHE 1 34.987 41.322 166.182 1.00 0.00

ATOM 6528 CD1 PHE 1 35.939 41.720 165.236 1.00 0.00

ATOM 6529 HD1 PHE 1 35.630 41.845 164.208 1.00 0.00

ATOM 6530 CE1 PHE 1 37.252 41.994 165.639 1.00 0.00

ATOM 6531 HE1 PHE 1 37.951 42.352 164.898 1.00 0.00

ATOM 6532 CZ PHE 1 37.594 42.034 166.996 1.00 0.00

ATOM 6533 HZ PHE 1 38.565 42.344 167.349 1.00 0.00

ATOM 6534 CE2 PHE 1 36.637 41.642 167.939 1.00 0.00

ATOM 6535 HE2 PHE 1 36.926 41.562 168.977 1.00 0.00

ATOM 6536 CD2 PHE 1 35.368 41.219 167.525 1.00 0.00

ATOM 6537 HD2 PHE 1 34.605 40.941 168.237 1.00 0.00

ATOM 6538 C PHE 1 33.241 38.704 166.290 1.00 0.00

ATOM 6539 O PHE 1 33.203 38.970 167.489 1.00 0.00

ATOM 6540 N THR 2 33.155 37.465 165.800 1.00 0.00

ATOM 6541 H THR 2 33.051 37.271 164.814 1.00 0.00

ATOM 6542 CA THR 2 33.429 36.263 166.561 1.00 0.00

ATOM 6543 HA THR 2 34.168 36.530 167.316 1.00 0.00

ATOM 6544 CB THR 2 32.210 35.665 167.258 1.00 0.00

ATOM 6545 HB THR 2 32.580 34.789 167.790 1.00 0.00

ATOM 6546 CG2 THR 2 31.597 36.651 168.249 1.00 0.00

ATOM 6547 1HG2 THR 2 30.853 36.156 168.872 1.00 0.00

ATOM 6548 2HG2 THR 2 31.119 37.495 167.753 1.00 0.00

ATOM 6549 3HG2 THR 2 32.296 37.075 168.969 1.00 0.00

ATOM 6550 OG1 THR 2 31.141 35.189 166.472 1.00 0.00

ATOM 6551 HG1 THR 2 30.690 34.527 167.002 1.00 0.00

ATOM 6552 C THR 2 33.989 35.215 165.608 1.00 0.00

ATOM 6553 O THR 2 33.843 35.300 164.391 1.00 0.00

ATOM 6554 N LEU 3 34.564 34.099 166.060 1.00 0.00

ATOM 6555 H LEU 3 34.855 34.058 167.026 1.00 0.00

ATOM 6556 CA LEU 3 35.186 33.043 165.285 1.00 0.00

ATOM 6557 HA LEU 3 35.707 33.572 164.488 1.00 0.00

ATOM 6558 CB LEU 3 36.195 32.328 166.182 1.00 0.00

ATOM 6559 HB1 LEU 3 36.641 33.095 166.815 1.00 0.00

ATOM 6560 HB2 LEU 3 37.108 32.030 165.668 1.00 0.00

ATOM 6561 CG LEU 3 35.659 31.167 167.014 1.00 0.00

ATOM 6562 HG LEU 3 35.390 30.390 166.297 1.00 0.00

ATOM 6563 CD1 LEU 3 36.800 30.644 167.882 1.00 0.00

ATOM 6564 1HD1 LEU 3 37.683 30.483 167.263 1.00 0.00

ATOM 6565 2HD1 LEU 3 36.493 29.681 168.285 1.00 0.00

ATOM 6566 3HD1 LEU 3 37.116 31.291 168.700 1.00 0.00

ATOM 6567 CD2 LEU 3 34.539 31.596 167.956 1.00 0.00

ATOM 6568 1HD2 LEU 3 34.849 32.523 168.437 1.00 0.00

ATOM 6569 2HD2 LEU 3 34.310 30.851 168.719 1.00 0.00

ATOM 6570 3HD2 LEU 3 33.655 31.907 167.399 1.00 0.00

ATOM 6571 C LEU 3 34.302 32.087 164.498 1.00 0.00

ATOM 6572 O LEU 3 34.806 31.335 163.668 1.00 0.00

ATOM 6573 N ILE 4 32.971 32.099 164.608 1.00 0.00

ATOM 6574 H ILE 4 32.574 32.716 165.300 1.00 0.00

ATOM 6575 CA ILE 4 31.975 31.530 163.723 1.00 0.00

ATOM 6576 HA ILE 4 32.241 30.493 163.521 1.00 0.00

ATOM 6577 CB ILE 4 30.598 31.622 164.377 1.00 0.00

ATOM 6578 HB ILE 4 30.700 31.427 165.445 1.00 0.00

ATOM 6579 CG2 ILE 4 30.038 33.016 164.105 1.00 0.00

ATOM 6580 1HG2 ILE 4 29.177 33.237 164.726 1.00 0.00

ATOM 6581 2HG2 ILE 4 29.681 33.209 163.094 1.00 0.00

ATOM 6582 3HG2 ILE 4 30.750 33.774 164.429 1.00 0.00

ATOM 6583 CG1 ILE 4 29.461 30.775 163.811 1.00 0.00

ATOM 6584 1HG1 ILE 4 28.536 31.038 164.324 1.00 0.00

ATOM 6585 2HG1 ILE 4 29.401 30.739 162.723 1.00 0.00

ATOM 6586 CD ILE 4 29.678 29.279 164.008 1.00 0.00

ATOM 6587 HD1 ILE 4 30.314 28.964 163.179 1.00 0.00

ATOM 6588 HD2 ILE 4 28.738 28.728 164.016 1.00 0.00

ATOM 6589 HD3 ILE 4 30.239 29.143 164.933 1.00 0.00

ATOM 6590 C ILE 4 32.095 32.169 162.348 1.00 0.00

ATOM 6591 O ILE 4 31.624 31.558 161.391 1.00 0.00

ATOM 6592 N GLU 5 32.726 33.339 162.219 1.00 0.00

ATOM 6593 H GLU 5 32.999 33.920 162.999 1.00 0.00

ATOM 6594 CA GLU 5 33.021 33.916 160.923 1.00 0.00

ATOM 6595 HA GLU 5 32.117 33.780 160.329 1.00 0.00

ATOM 6596 CB GLU 5 33.315 35.411 161.008 1.00 0.00

ATOM 6597 HB1 GLU 5 33.355 35.668 159.949 1.00 0.00

ATOM 6598 HB2 GLU 5 34.247 35.686 161.501 1.00 0.00

ATOM 6599 CG GLU 5 32.155 36.066 161.752 1.00 0.00

ATOM 6600 HG1 GLU 5 31.243 35.771 161.233 1.00 0.00

ATOM 6601 HG2 GLU 5 32.037 35.638 162.747 1.00 0.00

ATOM 6602 CD GLU 5 32.294 37.579 161.848 1.00 0.00

ATOM 6603 OE1 GLU 5 31.802 38.303 160.955 1.00 0.00

ATOM 6604 OE2 GLU 5 32.921 38.047 162.823 1.00 0.00

ATOM 6605 C GLU 5 34.186 33.209 160.246 1.00 0.00

ATOM 6606 O GLU 5 34.239 33.025 159.032 1.00 0.00

ATOM 6607 N LEU 6 35.169 32.740 161.018 1.00 0.00

ATOM 6608 H LEU 6 35.197 33.023 161.987 1.00 0.00

ATOM 6609 CA LEU 6 36.245 31.890 160.547 1.00 0.00

ATOM 6610 HA LEU 6 36.581 32.335 159.611 1.00 0.00

ATOM 6611 CB LEU 6 37.423 31.801 161.513 1.00 0.00

ATOM 6612 HB1 LEU 6 37.125 31.631 162.548 1.00 0.00

ATOM 6613 HB2 LEU 6 38.001 32.725 161.471 1.00 0.00

ATOM 6614 CG LEU 6 38.452 30.690 161.317 1.00 0.00

ATOM 6615 HG LEU 6 39.327 31.039 161.864 1.00 0.00

ATOM 6616 CD1 LEU 6 38.006 29.280 161.700 1.00 0.00

ATOM 6617 1HD1 LEU 6 37.296 28.807 161.021 1.00 0.00

ATOM 6618 2HD1 LEU 6 37.487 29.332 162.657 1.00 0.00

ATOM 6619 3HD1 LEU 6 38.785 28.530 161.831 1.00 0.00

ATOM 6620 CD2 LEU 6 39.134 30.694 159.954 1.00 0.00

ATOM 6621 1HD2 LEU 6 38.480 30.148 159.275 1.00 0.00

ATOM 6622 2HD2 LEU 6 40.079 30.161 160.067 1.00 0.00

ATOM 6623 3HD2 LEU 6 39.272 31.724 159.626 1.00 0.00

ATOM 6624 C LEU 6 35.653 30.585 160.033 1.00 0.00

ATOM 6625 O LEU 6 35.856 30.202 158.884 1.00 0.00

ATOM 6626 N LEU 7 34.713 29.970 160.754 1.00 0.00

ATOM 6627 H LEU 7 34.635 30.342 161.689 1.00 0.00

ATOM 6628 CA LEU 7 33.966 28.750 160.517 1.00 0.00

ATOM 6629 HA LEU 7 34.652 27.923 160.343 1.00 0.00

ATOM 6630 CB LEU 7 33.178 28.245 161.723 1.00 0.00

ATOM 6631 HB1 LEU 7 32.500 27.430 161.464 1.00 0.00

ATOM 6632 HB2 LEU 7 32.536 29.089 161.975 1.00 0.00

ATOM 6633 CG LEU 7 34.054 27.942 162.935 1.00 0.00

ATOM 6634 HG LEU 7 34.691 28.782 163.210 1.00 0.00

ATOM 6635 CD1 LEU 7 33.137 27.485 164.067 1.00 0.00

ATOM 6636 1HD1 LEU 7 33.744 27.328 164.957 1.00 0.00

ATOM 6637 2HD1 LEU 7 32.717 26.486 163.950 1.00 0.00

ATOM 6638 3HD1 LEU 7 32.276 28.118 164.280 1.00 0.00

ATOM 6639 CD2 LEU 7 35.028 26.795 162.676 1.00 0.00

ATOM 6640 1HD2 LEU 7 35.760 26.973 161.888 1.00 0.00

ATOM 6641 2HD2 LEU 7 34.373 25.991 162.340 1.00 0.00

ATOM 6642 3HD2 LEU 7 35.541 26.547 163.605 1.00 0.00

ATOM 6643 C LEU 7 33.089 28.802 159.276 1.00 0.00

ATOM 6644 O LEU 7 32.787 27.738 158.740 1.00 0.00

ATOM 6645 N ILE 8 32.782 30.039 158.878 1.00 0.00

ATOM 6646 H ILE 8 33.007 30.818 159.480 1.00 0.00

ATOM 6647 CA ILE 8 32.056 30.281 157.647 1.00 0.00

ATOM 6648 HA ILE 8 31.473 29.367 157.545 1.00 0.00

ATOM 6649 CB ILE 8 30.984 31.367 157.696 1.00 0.00

ATOM 6650 HB ILE 8 31.450 32.272 158.087 1.00 0.00

ATOM 6651 CG2 ILE 8 30.375 31.575 156.312 1.00 0.00

ATOM 6652 1HG2 ILE 8 29.811 30.717 155.948 1.00 0.00

ATOM 6653 2HG2 ILE 8 31.146 31.816 155.581 1.00 0.00

ATOM 6654 3HG2 ILE 8 29.662 32.400 156.299 1.00 0.00

ATOM 6655 CG1 ILE 8 29.844 31.000 158.641 1.00 0.00

ATOM 6656 1HG1 ILE 8 29.176 30.262 158.195 1.00 0.00

ATOM 6657 2HG1 ILE 8 30.207 30.508 159.544 1.00 0.00

ATOM 6658 CD ILE 8 28.970 32.160 159.108 1.00 0.00

ATOM 6659 HD1 ILE 8 28.280 32.402 158.299 1.00 0.00

ATOM 6660 HD2 ILE 8 29.630 33.005 159.303 1.00 0.00

ATOM 6661 HD3 ILE 8 28.445 31.816 159.999 1.00 0.00

ATOM 6662 C ILE 8 33.008 30.452 156.471 1.00 0.00

ATOM 6663 O ILE 8 32.878 29.805 155.435 1.00 0.00

ATOM 6664 N VAL 9 33.937 31.409 156.510 1.00 0.00

ATOM 6665 H VAL 9 34.107 31.742 157.447 1.00 0.00

ATOM 6666 CA VAL 9 34.655 31.929 155.363 1.00 0.00

ATOM 6667 HA VAL 9 33.796 32.045 154.702 1.00 0.00

ATOM 6668 CB VAL 9 35.240 33.285 155.754 1.00 0.00

ATOM 6669 HB VAL 9 34.540 33.743 156.451 1.00 0.00

ATOM 6670 CG1 VAL 9 36.520 33.154 156.575 1.00 0.00

ATOM 6671 1HG1 VAL 9 37.205 32.547 155.983 1.00 0.00

ATOM 6672 2HG1 VAL 9 36.314 32.651 157.520 1.00 0.00

ATOM 6673 3HG1 VAL 9 36.968 34.106 156.858 1.00 0.00

ATOM 6674 CG2 VAL 9 35.479 34.341 154.679 1.00 0.00

ATOM 6675 1HG2 VAL 9 36.366 34.137 154.079 1.00 0.00

ATOM 6676 2HG2 VAL 9 35.608 35.334 155.108 1.00 0.00

ATOM 6677 3HG2 VAL 9 34.578 34.411 154.069 1.00 0.00

ATOM 6678 C VAL 9 35.671 31.053 154.641 1.00 0.00

ATOM 6679 O VAL 9 36.338 31.459 153.693 1.00 0.00

ATOM 6680 N VAL 10 35.982 29.873 155.181 1.00 0.00

ATOM 6681 H VAL 10 35.638 29.614 156.095 1.00 0.00

ATOM 6682 CA VAL 10 36.712 28.833 154.484 1.00 0.00

ATOM 6683 HA VAL 10 37.077 29.290 153.578 1.00 0.00

ATOM 6684 CB VAL 10 37.991 28.345 155.159 1.00 0.00

ATOM 6685 HB VAL 10 37.752 27.675 155.985 1.00 0.00

ATOM 6686 CG1 VAL 10 38.876 27.549 154.205 1.00 0.00

ATOM 6687 1HG1 VAL 10 39.110 28.091 153.289 1.00 0.00

ATOM 6688 2HG1 VAL 10 38.426 26.611 153.930 1.00 0.00

ATOM 6689 3HG1 VAL 10 39.789 27.368 154.773 1.00 0.00

ATOM 6690 CG2 VAL 10 38.758 29.522 155.754 1.00 0.00

ATOM 6691 1HG2 VAL 10 39.038 30.193 154.942 1.00 0.00

ATOM 6692 2HG2 VAL 10 39.662 29.158 156.243 1.00 0.00

ATOM 6693 3HG2 VAL 10 38.172 30.109 156.461 1.00 0.00

ATOM 6694 C VAL 10 35.736 27.732 154.096 1.00 0.00

ATOM 6695 O VAL 10 35.579 27.414 152.920 1.00 0.00

ATOM 6696 N ALA 11 35.018 27.137 155.052 1.00 0.00

ATOM 6697 H ALA 11 35.195 27.321 156.030 1.00 0.00

ATOM 6698 CA ALA 11 34.149 26.005 154.803 1.00 0.00

ATOM 6699 HA ALA 11 34.745 25.123 154.567 1.00 0.00

ATOM 6700 CB ALA 11 33.461 25.670 156.124 1.00 0.00

ATOM 6701 HB1 ALA 11 32.804 26.487 156.421 1.00 0.00

ATOM 6702 HB2 ALA 11 34.119 25.428 156.958 1.00 0.00

ATOM 6703 HB3 ALA 11 32.769 24.838 156.009 1.00 0.00

ATOM 6704 C ALA 11 33.101 26.284 153.736 1.00 0.00

ATOM 6705 O ALA 11 32.847 25.381 152.944 1.00 0.00

ATOM 6706 N ILE 12 32.412 27.427 153.792 1.00 0.00

ATOM 6707 H ILE 12 32.857 28.155 154.331 1.00 0.00

ATOM 6708 CA ILE 12 31.308 27.702 152.894 1.00 0.00

ATOM 6709 HA ILE 12 30.774 26.766 152.727 1.00 0.00

ATOM 6710 CB ILE 12 30.392 28.738 153.542 1.00 0.00

ATOM 6711 HB ILE 12 31.002 29.396 154.160 1.00 0.00

ATOM 6712 CG2 ILE 12 29.586 29.633 152.604 1.00 0.00

ATOM 6713 1HG2 ILE 12 28.872 30.343 153.020 1.00 0.00

ATOM 6714 2HG2 ILE 12 28.923 28.982 152.037 1.00 0.00

ATOM 6715 3HG2 ILE 12 30.110 30.146 151.797 1.00 0.00

ATOM 6716 CG1 ILE 12 29.348 28.133 154.478 1.00 0.00

ATOM 6717 1HG1 ILE 12 28.756 28.888 154.996 1.00 0.00

ATOM 6718 2HG1 ILE 12 28.667 27.479 153.932 1.00 0.00

ATOM 6719 CD ILE 12 29.971 27.349 155.628 1.00 0.00

ATOM 6720 HD1 ILE 12 29.228 27.174 156.407 1.00 0.00

ATOM 6721 HD2 ILE 12 30.852 27.897 155.962 1.00 0.00

ATOM 6722 HD3 ILE 12 30.274 26.388 155.213 1.00 0.00

ATOM 6723 C ILE 12 31.776 28.028 151.484 1.00 0.00

ATOM 6724 O ILE 12 31.086 27.628 150.547 1.00 0.00

ATOM 6725 N ILE 13 33.024 28.485 151.357 1.00 0.00

ATOM 6726 H ILE 13 33.656 28.542 152.143 1.00 0.00

ATOM 6727 CA ILE 13 33.618 28.566 150.037 1.00 0.00

ATOM 6728 HA ILE 13 32.964 28.924 149.242 1.00 0.00

ATOM 6729 CB ILE 13 34.781 29.554 150.075 1.00 0.00

ATOM 6730 HB ILE 13 35.401 29.175 150.886 1.00 0.00

ATOM 6731 CG2 ILE 13 35.673 29.486 148.840 1.00 0.00

ATOM 6732 1HG2 ILE 13 36.514 30.159 149.002 1.00 0.00

ATOM 6733 2HG2 ILE 13 35.149 29.652 147.899 1.00 0.00

ATOM 6734 3HG2 ILE 13 36.168 28.520 148.739 1.00 0.00

ATOM 6735 CG1 ILE 13 34.353 30.984 150.397 1.00 0.00

ATOM 6736 1HG1 ILE 13 33.636 31.247 149.619 1.00 0.00

ATOM 6737 2HG1 ILE 13 33.860 31.002 151.369 1.00 0.00

ATOM 6738 CD ILE 13 35.413 32.078 150.486 1.00 0.00

ATOM 6739 HD1 ILE 13 35.008 32.872 151.114 1.00 0.00

ATOM 6740 HD2 ILE 13 35.585 32.393 149.457 1.00 0.00

ATOM 6741 HD3 ILE 13 36.251 31.684 151.060 1.00 0.00

ATOM 6742 C ILE 13 33.910 27.190 149.458 1.00 0.00

ATOM 6743 O ILE 13 33.828 26.949 148.256 1.00 0.00

ATOM 6744 N GLY 14 34.209 26.248 150.356 1.00 0.00

ATOM 6745 H GLY 14 34.174 26.425 151.350 1.00 0.00

ATOM 6746 CA GLY 14 34.190 24.834 150.037 1.00 0.00

ATOM 6747 HA1 GLY 14 34.993 24.424 149.425 1.00 0.00

ATOM 6748 HA2 GLY 14 34.331 24.270 150.958 1.00 0.00

ATOM 6749 C GLY 14 32.889 24.256 149.499 1.00 0.00

ATOM 6750 O GLY 14 32.891 23.359 148.659 1.00 0.00

ATOM 6751 N ILE 15 31.742 24.884 149.769 1.00 0.00

ATOM 6752 H ILE 15 31.751 25.668 150.405 1.00 0.00

ATOM 6753 CA ILE 15 30.437 24.455 149.307 1.00 0.00

ATOM 6754 HA ILE 15 30.438 23.394 149.057 1.00 0.00

ATOM 6755 CB ILE 15 29.372 24.710 150.370 1.00 0.00

ATOM 6756 HB ILE 15 29.337 25.776 150.593 1.00 0.00

ATOM 6757 CG2 ILE 15 27.998 24.380 149.793 1.00 0.00

ATOM 6758 1HG2 ILE 15 27.184 24.742 150.421 1.00 0.00

ATOM 6759 2HG2 ILE 15 27.979 23.314 149.567 1.00 0.00

ATOM 6760 3HG2 ILE 15 27.838 24.918 148.858 1.00 0.00

ATOM 6761 CG1 ILE 15 29.717 24.088 151.721 1.00 0.00

ATOM 6762 1HG1 ILE 15 29.812 23.010 151.600 1.00 0.00

ATOM 6763 2HG1 ILE 15 30.712 24.272 152.125 1.00 0.00

ATOM 6764 CD ILE 15 28.757 24.461 152.847 1.00 0.00

ATOM 6765 HD1 ILE 15 29.250 24.086 153.744 1.00 0.00

ATOM 6766 HD2 ILE 15 27.848 23.864 152.771 1.00 0.00

ATOM 6767 HD3 ILE 15 28.598 25.537 152.920 1.00 0.00

ATOM 6768 C ILE 15 30.160 25.225 148.024 1.00 0.00

ATOM 6769 O ILE 15 29.626 24.647 147.082 1.00 0.00

ATOM 6770 N LEU 16 30.516 26.507 147.907 1.00 0.00

ATOM 6771 H LEU 16 31.055 26.897 148.667 1.00 0.00

ATOM 6772 CA LEU 16 30.504 27.211 146.641 1.00 0.00

ATOM 6773 HA LEU 16 29.498 27.140 146.226 1.00 0.00

ATOM 6774 CB LEU 16 30.957 28.651 146.859 1.00 0.00

ATOM 6775 HB1 LEU 16 31.223 29.052 145.881 1.00 0.00

ATOM 6776 HB2 LEU 16 31.808 28.685 147.540 1.00 0.00

ATOM 6777 CG LEU 16 29.992 29.689 147.424 1.00 0.00

ATOM 6778 HG LEU 16 30.231 30.592 146.864 1.00 0.00

ATOM 6779 CD1 LEU 16 28.550 29.354 147.055 1.00 0.00

ATOM 6780 1HD1 LEU 16 27.903 30.227 147.131 1.00 0.00

ATOM 6781 2HD1 LEU 16 28.192 28.584 147.740 1.00 0.00

ATOM 6782 3HD1 LEU 16 28.417 28.956 146.049 1.00 0.00

ATOM 6783 CD2 LEU 16 30.077 30.136 148.880 1.00 0.00

ATOM 6784 1HD2 LEU 16 29.560 31.093 148.819 1.00 0.00

ATOM 6785 2HD2 LEU 16 31.146 30.302 149.008 1.00 0.00

ATOM 6786 3HD2 LEU 16 29.539 29.421 149.504 1.00 0.00

ATOM 6787 C LEU 16 31.467 26.565 145.653 1.00 0.00

ATOM 6788 O LEU 16 31.278 26.765 144.457 1.00 0.00

ATOM 6789 N ALA 17 32.545 25.878 146.039 1.00 0.00

ATOM 6790 H ALA 17 32.760 25.850 147.025 1.00 0.00

ATOM 6791 CA ALA 17 33.567 25.357 145.154 1.00 0.00

ATOM 6792 HA ALA 17 34.104 26.188 144.697 1.00 0.00

ATOM 6793 CB ALA 17 34.607 24.602 145.977 1.00 0.00

ATOM 6794 HB1 ALA 17 35.399 24.091 145.430 1.00 0.00

ATOM 6795 HB2 ALA 17 34.141 23.892 146.661 1.00 0.00

ATOM 6796 HB3 ALA 17 35.057 25.421 146.538 1.00 0.00

ATOM 6797 C ALA 17 32.956 24.455 144.092 1.00 0.00

ATOM 6798 O ALA 17 33.343 24.471 142.926 1.00 0.00

ATOM 6799 N ALA 18 31.894 23.730 144.449 1.00 0.00

ATOM 6800 H ALA 18 31.792 23.639 145.450 1.00 0.00

ATOM 6801 CA ALA 18 31.142 22.805 143.625 1.00 0.00

ATOM 6802 HA ALA 18 31.837 21.994 143.409 1.00 0.00

ATOM 6803 CB ALA 18 30.043 22.291 144.549 1.00 0.00

ATOM 6804 HB1 ALA 18 30.523 22.015 145.482 1.00 0.00

ATOM 6805 HB2 ALA 18 29.309 23.082 144.707 1.00 0.00

ATOM 6806 HB3 ALA 18 29.517 21.458 144.085 1.00 0.00

ATOM 6807 C ALA 18 30.636 23.404 142.320 1.00 0.00

ATOM 6808 O ALA 18 30.597 22.716 141.302 1.00 0.00

ATOM 6809 N ILE 19 30.154 24.643 142.428 1.00 0.00

ATOM 6810 H ILE 19 30.295 25.089 143.324 1.00 0.00

ATOM 6811 CA ILE 19 29.692 25.427 141.301 1.00 0.00

ATOM 6812 HA ILE 19 29.362 24.741 140.520 1.00 0.00

ATOM 6813 CB ILE 19 28.434 26.223 141.639 1.00 0.00

ATOM 6814 HB ILE 19 28.239 26.866 140.782 1.00 0.00

ATOM 6815 CG2 ILE 19 27.275 25.269 141.920 1.00 0.00

ATOM 6816 1HG2 ILE 19 27.467 24.683 142.818 1.00 0.00

ATOM 6817 2HG2 ILE 19 27.251 24.538 141.113 1.00 0.00

ATOM 6818 3HG2 ILE 19 26.290 25.732 141.981 1.00 0.00

ATOM 6819 CG1 ILE 19 28.583 27.260 142.749 1.00 0.00

ATOM 6820 1HG1 ILE 19 28.314 26.879 143.733 1.00 0.00

ATOM 6821 2HG1 ILE 19 29.618 27.582 142.861 1.00 0.00

ATOM 6822 CD ILE 19 27.571 28.399 142.657 1.00 0.00

ATOM 6823 HD1 ILE 19 27.356 28.848 143.627 1.00 0.00

ATOM 6824 HD2 ILE 19 26.559 28.024 142.511 1.00 0.00

ATOM 6825 HD3 ILE 19 27.751 29.083 141.827 1.00 0.00

ATOM 6826 C ILE 19 30.768 26.305 140.676 1.00 0.00

ATOM 6827 O ILE 19 30.547 26.777 139.563 1.00 0.00

ATOM 6828 N ALA 20 31.906 26.527 141.337 1.00 0.00

ATOM 6829 H ALA 20 32.001 26.082 142.239 1.00 0.00

ATOM 6830 CA ALA 20 32.939 27.388 140.793 1.00 0.00

ATOM 6831 HA ALA 20 32.455 28.230 140.301 1.00 0.00

ATOM 6832 CB ALA 20 33.701 27.995 141.967 1.00 0.00

ATOM 6833 HB1 ALA 20 34.091 27.155 142.542 1.00 0.00

ATOM 6834 HB2 ALA 20 34.423 28.750 141.656 1.00 0.00

ATOM 6835 HB3 ALA 20 32.989 28.476 142.637 1.00 0.00

ATOM 6836 C ALA 20 33.880 26.703 139.812 1.00 0.00

ATOM 6837 O ALA 20 33.987 27.091 138.651 1.00 0.00

ATOM 6838 N ILE 21 34.492 25.667 140.390 1.00 0.00

ATOM 6839 H ILE 21 34.228 25.477 141.347 1.00 0.00

ATOM 6840 CA ILE 21 35.415 24.771 139.725 1.00 0.00

ATOM 6841 HA ILE 21 36.296 25.383 139.531 1.00 0.00

ATOM 6842 CB ILE 21 35.797 23.731 140.774 1.00 0.00

ATOM 6843 HB ILE 21 34.910 23.175 141.076 1.00 0.00

ATOM 6844 CG2 ILE 21 36.680 22.635 140.184 1.00 0.00

ATOM 6845 1HG2 ILE 21 37.055 21.954 140.947 1.00 0.00

ATOM 6846 2HG2 ILE 21 37.504 23.053 139.607 1.00 0.00

ATOM 6847 3HG2 ILE 21 36.234 22.007 139.414 1.00 0.00

ATOM 6848 CG1 ILE 21 36.493 24.277 142.019 1.00 0.00

ATOM 6849 1HG1 ILE 21 35.808 24.925 142.562 1.00 0.00

ATOM 6850 2HG1 ILE 21 36.531 23.451 142.728 1.00 0.00

ATOM 6851 CD ILE 21 37.836 24.993 141.892 1.00 0.00

ATOM 6852 HD1 ILE 21 38.529 24.315 141.394 1.00 0.00

ATOM 6853 HD2 ILE 21 38.271 25.121 142.884 1.00 0.00

ATOM 6854 HD3 ILE 21 37.742 26.006 141.501 1.00 0.00

ATOM 6855 C ILE 21 34.972 24.190 138.389 1.00 0.00

ATOM 6856 O ILE 21 35.836 24.190 137.514 1.00 0.00

ATOM 6857 N PRO 22 33.749 23.721 138.131 1.00 0.00

ATOM 6858 CD PRO 22 32.695 23.245 139.004 1.00 0.00

ATOM 6859 HD1 PRO 22 32.491 23.920 139.835 1.00 0.00

ATOM 6860 HD2 PRO 22 33.006 22.298 139.446 1.00 0.00

ATOM 6861 CG PRO 22 31.519 22.935 138.083 1.00 0.00

ATOM 6862 HG1 PRO 22 30.985 23.880 137.995 1.00 0.00

ATOM 6863 HG2 PRO 22 30.775 22.251 138.494 1.00 0.00

ATOM 6864 CB PRO 22 32.160 22.563 136.749 1.00 0.00

ATOM 6865 HB1 PRO 22 31.515 22.980 135.975 1.00 0.00

ATOM 6866 HB2 PRO 22 32.388 21.499 136.675 1.00 0.00

ATOM 6867 CA PRO 22 33.474 23.340 136.761 1.00 0.00

ATOM 6868 HA PRO 22 34.266 22.655 136.459 1.00 0.00

ATOM 6869 C PRO 22 33.480 24.422 135.690 1.00 0.00

ATOM 6870 O PRO 22 33.783 24.098 134.544 1.00 0.00

ATOM 6871 N GLN 23 33.221 25.680 136.052 1.00 0.00

ATOM 6872 H GLN 23 33.107 25.737 137.053 1.00 0.00

ATOM 6873 CA GLN 23 32.777 26.750 135.179 1.00 0.00

ATOM 6874 HA GLN 23 32.246 26.211 134.394 1.00 0.00

ATOM 6875 CB GLN 23 31.724 27.618 135.860 1.00 0.00

ATOM 6876 HB1 GLN 23 31.434 28.401 135.160 1.00 0.00

ATOM 6877 HB2 GLN 23 32.082 28.059 136.791 1.00 0.00

ATOM 6878 CG GLN 23 30.451 26.813 136.104 1.00 0.00

ATOM 6879 HG1 GLN 23 30.682 25.984 136.773 1.00 0.00

ATOM 6880 HG2 GLN 23 29.753 27.523 136.547 1.00 0.00

ATOM 6881 CD GLN 23 29.742 26.229 134.890 1.00 0.00

ATOM 6882 OE1 GLN 23 29.944 26.743 133.792 1.00 0.00

ATOM 6883 NE2 GLN 23 29.004 25.127 135.039 1.00 0.00

ATOM 6884 1HE2 GLN 23 28.835 24.803 135.981 1.00 0.00

ATOM 6885 2HE2 GLN 23 28.556 24.701 134.241 1.00 0.00

ATOM 6886 C GLN 23 33.929 27.529 134.559 1.00 0.00

ATOM 6887 O GLN 23 33.709 28.523 133.871 1.00 0.00

ATOM 6888 N PHE 24 35.166 27.077 134.775 1.00 0.00

ATOM 6889 H PHE 24 35.320 26.321 135.427 1.00 0.00

ATOM 6890 CA PHE 24 36.313 27.576 134.041 1.00 0.00

ATOM 6891 HA PHE 24 36.022 27.907 133.044 1.00 0.00

ATOM 6892 CB PHE 24 36.888 28.801 134.743 1.00 0.00

ATOM 6893 HB1 PHE 24 36.093 29.535 134.861 1.00 0.00

ATOM 6894 HB2 PHE 24 37.568 29.261 134.025 1.00 0.00

ATOM 6895 CG PHE 24 37.530 28.386 136.046 1.00 0.00

ATOM 6896 CD1 PHE 24 36.735 28.165 137.177 1.00 0.00

ATOM 6897 HD1 PHE 24 35.666 28.299 137.100 1.00 0.00

ATOM 6898 CE1 PHE 24 37.319 27.746 138.378 1.00 0.00

ATOM 6899 HE1 PHE 24 36.673 27.650 139.238 1.00 0.00

ATOM 6900 CZ PHE 24 38.716 27.672 138.449 1.00 0.00

ATOM 6901 HZ PHE 24 39.215 27.298 139.331 1.00 0.00

ATOM 6902 CE2 PHE 24 39.517 28.036 137.360 1.00 0.00

ATOM 6903 HE2 PHE 24 40.579 27.854 137.445 1.00 0.00

ATOM 6904 CD2 PHE 24 38.927 28.401 136.145 1.00 0.00

ATOM 6905 HD2 PHE 24 39.580 28.648 135.321 1.00 0.00

ATOM 6906 C PHE 24 37.521 26.664 133.892 1.00 0.00

ATOM 6907 O PHE 24 38.373 26.955 133.055 1.00 0.00

ATOM 6908 N SER 25 37.543 25.565 134.650 1.00 0.00

ATOM 6909 H SER 25 36.908 25.375 135.412 1.00 0.00

ATOM 6910 CA SER 25 38.665 24.653 134.559 1.00 0.00

ATOM 6911 HA SER 25 39.576 25.250 134.531 1.00 0.00

ATOM 6912 CB SER 25 38.625 23.795 135.821 1.00 0.00

ATOM 6913 HB1 SER 25 38.720 24.359 136.749 1.00 0.00

ATOM 6914 HB2 SER 25 39.399 23.028 135.797 1.00 0.00

ATOM 6915 OG SER 25 37.475 22.984 135.900 1.00 0.00

ATOM 6916 HG SER 25 36.867 23.335 136.556 1.00 0.00

ATOM 6917 C SER 25 38.795 23.782 133.317 1.00 0.00

ATOM 6918 O SER 25 39.808 23.787 132.621 1.00 0.00

ATOM 6919 N ALA 26 37.695 23.161 132.892 1.00 0.00

ATOM 6920 H ALA 26 37.033 22.999 133.640 1.00 0.00

ATOM 6921 CA ALA 26 37.556 22.362 131.694 1.00 0.00

ATOM 6922 HA ALA 26 38.186 21.489 131.869 1.00 0.00

ATOM 6923 CB ALA 26 36.107 21.901 131.565 1.00 0.00

ATOM 6924 HB1 ALA 26 35.814 21.800 130.520 1.00 0.00

ATOM 6925 HB2 ALA 26 35.968 20.869 131.885 1.00 0.00

ATOM 6926 HB3 ALA 26 35.415 22.540 132.113 1.00 0.00

ATOM 6927 C ALA 26 37.984 23.127 130.450 1.00 0.00

ATOM 6928 O ALA 26 38.834 22.635 129.711 1.00 0.00

ATOM 6929 N ALA 27 37.495 24.361 130.321 1.00 0.00

ATOM 6930 H ALA 27 36.919 24.608 131.112 1.00 0.00

ATOM 6931 CA ALA 27 37.857 25.270 129.251 1.00 0.00

ATOM 6932 HA ALA 27 37.594 24.957 128.241 1.00 0.00

ATOM 6933 CB ALA 27 37.169 26.574 129.643 1.00 0.00

ATOM 6934 HB1 ALA 27 36.097 26.428 129.767 1.00 0.00

ATOM 6935 HB2 ALA 27 37.459 27.357 128.943 1.00 0.00

ATOM 6936 HB3 ALA 27 37.461 26.910 130.637 1.00 0.00

ATOM 6937 C ALA 27 39.353 25.535 129.154 1.00 0.00

ATOM 6938 O ALA 27 39.826 25.674 128.028 1.00 0.00

ATOM 6939 N ARG 28 40.081 25.654 130.267 1.00 0.00

ATOM 6940 H ARG 28 39.577 25.596 131.140 1.00 0.00

ATOM 6941 CA ARG 28 41.498 25.951 130.351 1.00 0.00

ATOM 6942 HA ARG 28 41.783 26.574 129.504 1.00 0.00

ATOM 6943 CB ARG 28 41.698 26.759 131.628 1.00 0.00

ATOM 6944 HB1 ARG 28 41.336 26.116 132.431 1.00 0.00

ATOM 6945 HB2 ARG 28 41.132 27.673 131.451 1.00 0.00

ATOM 6946 CG ARG 28 43.102 27.301 131.879 1.00 0.00

ATOM 6947 HG1 ARG 28 43.038 27.695 132.894 1.00 0.00

ATOM 6948 HG2 ARG 28 43.744 26.421 131.861 1.00 0.00

ATOM 6949 CD ARG 28 43.756 28.291 130.919 1.00 0.00

ATOM 6950 HD1 ARG 28 43.548 27.909 129.920 1.00 0.00

ATOM 6951 HD2 ARG 28 43.249 29.244 131.069 1.00 0.00

ATOM 6952 NE ARG 28 45.220 28.322 130.964 1.00 0.00

ATOM 6953 HE ARG 28 45.818 27.509 131.014 1.00 0.00

ATOM 6954 CZ ARG 28 45.956 29.429 131.114 1.00 0.00

ATOM 6955 NH1 ARG 28 45.446 30.668 131.144 1.00 0.00

ATOM 6956 1HH1 ARG 28 44.498 30.903 130.882 1.00 0.00

ATOM 6957 2HH1 ARG 28 46.023 31.407 131.516 1.00 0.00

ATOM 6958 NH2 ARG 28 47.220 29.335 131.548 1.00 0.00

ATOM 6959 1HH2 ARG 28 47.787 28.513 131.391 1.00 0.00

ATOM 6960 2HH2 ARG 28 47.702 30.196 131.759 1.00 0.00

ATOM 6961 C ARG 28 42.478 24.786 130.321 1.00 0.00

ATOM 6962 O ARG 28 43.618 24.932 129.885 1.00 0.00

ATOM 6963 N VAL 29 41.991 23.592 130.663 1.00 0.00

ATOM 6964 H VAL 29 41.127 23.608 131.183 1.00 0.00

ATOM 6965 CA VAL 29 42.362 22.354 130.007 1.00 0.00

ATOM 6966 HA VAL 29 43.420 22.226 130.229 1.00 0.00

ATOM 6967 CB VAL 29 41.693 21.098 130.560 1.00 0.00

ATOM 6968 HB VAL 29 40.620 21.276 130.634 1.00 0.00

ATOM 6969 CG1 VAL 29 41.978 19.803 129.800 1.00 0.00

ATOM 6970 1HG1 VAL 29 41.482 19.767 128.830 1.00 0.00

ATOM 6971 2HG1 VAL 29 41.730 18.928 130.399 1.00 0.00

ATOM 6972 3HG1 VAL 29 43.033 19.610 129.606 1.00 0.00

ATOM 6973 CG2 VAL 29 42.085 20.893 132.019 1.00 0.00

ATOM 6974 1HG2 VAL 29 41.654 19.967 132.400 1.00 0.00

ATOM 6975 2HG2 VAL 29 41.704 21.693 132.654 1.00 0.00

ATOM 6976 3HG2 VAL 29 43.165 20.836 132.157 1.00 0.00

ATOM 6977 C VAL 29 42.176 22.327 128.498 1.00 0.00

ATOM 6978 O VAL 29 43.196 22.183 127.826 1.00 0.00

ATOM 6979 N LYS 30 40.929 22.466 128.040 1.00 0.00

ATOM 6980 H LYS 30 40.196 22.658 128.707 1.00 0.00

ATOM 6981 CA LYS 30 40.572 22.396 126.637 1.00 0.00

ATOM 6982 HA LYS 30 40.879 21.435 126.226 1.00 0.00

ATOM 6983 CB LYS 30 39.052 22.462 126.519 1.00 0.00

ATOM 6984 HB1 LYS 30 38.682 23.362 127.011 1.00 0.00

ATOM 6985 HB2 LYS 30 38.535 21.641 127.013 1.00 0.00

ATOM 6986 CG LYS 30 38.426 22.451 125.127 1.00 0.00

ATOM 6987 HG1 LYS 30 38.568 23.383 124.581 1.00 0.00

ATOM 6988 HG2 LYS 30 38.709 21.570 124.551 1.00 0.00

ATOM 6989 CD LYS 30 36.925 22.433 125.406 1.00 0.00

ATOM 6990 HD1 LYS 30 36.695 21.463 125.847 1.00 0.00

ATOM 6991 HD2 LYS 30 36.609 23.214 126.098 1.00 0.00

ATOM 6992 CE LYS 30 36.072 22.520 124.146 1.00 0.00

ATOM 6993 HE1 LYS 30 35.820 23.563 123.952 1.00 0.00

ATOM 6994 HE2 LYS 30 36.746 22.284 123.323 1.00 0.00

ATOM 6995 NZ LYS 30 34.948 21.570 124.119 1.00 0.00

ATOM 6996 HZ1 LYS 30 34.417 21.564 124.978 1.00 0.00

ATOM 6997 HZ2 LYS 30 35.213 20.621 123.897 1.00 0.00

ATOM 6998 HZ3 LYS 30 34.322 21.791 123.358 1.00 0.00

ATOM 6999 C LYS 30 41.335 23.414 125.802 1.00 0.00

ATOM 7000 O LYS 30 41.792 23.072 124.713 1.00 0.00

ATOM 7001 N ALA 31 41.539 24.645 126.275 1.00 0.00

ATOM 7002 H ALA 31 41.244 24.823 127.224 1.00 0.00

ATOM 7003 CA ALA 31 42.328 25.664 125.613 1.00 0.00

ATOM 7004 HA ALA 31 41.911 25.941 124.645 1.00 0.00

ATOM 7005 CB ALA 31 42.199 26.865 126.546 1.00 0.00

ATOM 7006 HB1 ALA 31 41.228 27.255 126.855 1.00 0.00

ATOM 7007 HB2 ALA 31 42.756 26.621 127.451 1.00 0.00

ATOM 7008 HB3 ALA 31 42.686 27.730 126.098 1.00 0.00

ATOM 7009 C ALA 31 43.794 25.299 125.410 1.00 0.00

ATOM 7010 O ALA 31 44.467 25.962 124.627 1.00 0.00

ATOM 7011 N TYR 32 44.367 24.219 125.946 1.00 0.00

ATOM 7012 H TYR 32 43.758 23.489 126.285 1.00 0.00

ATOM 7013 CA TYR 32 45.752 23.813 125.807 1.00 0.00

ATOM 7014 HA TYR 32 46.346 24.726 125.848 1.00 0.00

ATOM 7015 CB TYR 32 46.128 22.874 126.950 1.00 0.00

ATOM 7016 HB1 TYR 32 45.844 21.875 126.619 1.00 0.00

ATOM 7017 HB2 TYR 32 45.604 23.065 127.886 1.00 0.00

ATOM 7018 CG TYR 32 47.562 22.870 127.421 1.00 0.00

ATOM 7019 CD1 TYR 32 48.164 23.943 128.091 1.00 0.00

ATOM 7020 HD1 TYR 32 47.587 24.847 128.209 1.00 0.00

ATOM 7021 CE1 TYR 32 49.494 23.899 128.526 1.00 0.00

ATOM 7022 HE1 TYR 32 49.941 24.684 129.118 1.00 0.00

ATOM 7023 CZ TYR 32 50.205 22.693 128.361 1.00 0.00

ATOM 7024 OH TYR 32 51.400 22.480 128.983 1.00 0.00

ATOM 7025 HH TYR 32 51.712 23.322 129.322 1.00 0.00

ATOM 7026 CE2 TYR 32 49.650 21.624 127.632 1.00 0.00

ATOM 7027 HE2 TYR 32 50.186 20.704 127.449 1.00 0.00

ATOM 7028 CD2 TYR 32 48.332 21.728 127.170 1.00 0.00

ATOM 7029 HD2 TYR 32 47.829 20.866 126.756 1.00 0.00

ATOM 7030 C TYR 32 46.152 23.133 124.505 1.00 0.00

ATOM 7031 O TYR 32 47.233 23.403 123.987 1.00 0.00

ATOM 7032 N ASN 33 45.311 22.267 123.936 1.00 0.00

ATOM 7033 H ASN 33 44.449 22.006 124.395 1.00 0.00

ATOM 7034 CA ASN 33 45.610 21.507 122.739 1.00 0.00

ATOM 7035 HA ASN 33 46.274 22.130 122.142 1.00 0.00

ATOM 7036 CB ASN 33 46.221 20.152 123.087 1.00 0.00

ATOM 7037 HB1 ASN 33 46.939 20.311 123.891 1.00 0.00

ATOM 7038 HB2 ASN 33 45.461 19.491 123.506 1.00 0.00

ATOM 7039 CG ASN 33 46.988 19.555 121.915 1.00 0.00

ATOM 7040 OD1 ASN 33 47.313 20.181 120.908 1.00 0.00

ATOM 7041 ND2 ASN 33 47.498 18.343 122.132 1.00 0.00

ATOM 7042 1HD2 ASN 33 47.187 17.777 122.911 1.00 0.00

ATOM 7043 2HD2 ASN 33 48.202 17.971 121.510 1.00 0.00

ATOM 7044 C ASN 33 44.319 21.383 121.944 1.00 0.00

ATOM 7045 O ASN 33 44.328 21.677 120.751 1.00 0.00

ATOM 7046 N SER 34 43.252 21.035 122.668 1.00 0.00

ATOM 7047 H SER 34 43.455 20.701 123.599 1.00 0.00

ATOM 7048 CA SER 34 41.970 20.663 122.103 1.00 0.00

ATOM 7049 HA SER 34 42.124 19.865 121.377 1.00 0.00

ATOM 7050 CB SER 34 41.116 20.067 123.219 1.00 0.00

ATOM 7051 HB1 SER 34 40.159 19.817 122.762 1.00 0.00

ATOM 7052 HB2 SER 34 40.911 20.732 124.057 1.00 0.00

ATOM 7053 OG SER 34 41.630 18.944 123.897 1.00 0.00

ATOM 7054 HG SER 34 41.589 19.167 124.830 1.00 0.00

ATOM 7055 C SER 34 41.291 21.833 121.405 1.00 0.00

ATOM 7056 O SER 34 40.613 21.641 120.399 1.00 0.00

ATOM 7057 N ALA 35 41.338 23.085 121.866 1.00 0.00

ATOM 7058 H ALA 35 41.687 23.235 122.802 1.00 0.00

ATOM 7059 CA ALA 35 40.800 24.234 121.167 1.00 0.00

ATOM 7060 HA ALA 35 39.834 23.995 120.720 1.00 0.00

ATOM 7061 CB ALA 35 40.483 25.254 122.256 1.00 0.00

ATOM 7062 HB1 ALA 35 39.925 24.832 123.091 1.00 0.00

ATOM 7063 HB2 ALA 35 41.401 25.614 122.720 1.00 0.00

ATOM 7064 HB3 ALA 35 39.971 26.084 121.769 1.00 0.00

ATOM 7065 C ALA 35 41.713 24.755 120.065 1.00 0.00

ATOM 7066 O ALA 35 41.279 25.703 119.415 1.00 0.00

ATOM 7067 N ALA 36 42.879 24.156 119.812 1.00 0.00

ATOM 7068 H ALA 36 43.114 23.393 120.430 1.00 0.00

ATOM 7069 CA ALA 36 43.566 24.260 118.538 1.00 0.00

ATOM 7070 HA ALA 36 43.195 25.064 117.903 1.00 0.00

ATOM 7071 CB ALA 36 45.063 24.455 118.756 1.00 0.00

ATOM 7072 HB1 ALA 36 45.395 23.719 119.487 1.00 0.00

ATOM 7073 HB2 ALA 36 45.209 25.400 119.278 1.00 0.00

ATOM 7074 HB3 ALA 36 45.639 24.448 117.830 1.00 0.00

ATOM 7075 C ALA 36 43.438 23.095 117.568 1.00 0.00

ATOM 7076 O ALA 36 43.174 23.343 116.394 1.00 0.00

ATOM 7077 N SER 37 43.430 21.865 118.088 1.00 0.00

ATOM 7078 H SER 37 43.527 21.728 119.084 1.00 0.00

ATOM 7079 CA SER 37 43.033 20.722 117.290 1.00 0.00

ATOM 7080 HA SER 37 43.709 20.684 116.436 1.00 0.00

ATOM 7081 CB SER 37 43.098 19.443 118.118 1.00 0.00

ATOM 7082 HB1 SER 37 42.863 19.780 119.126 1.00 0.00

ATOM 7083 HB2 SER 37 42.350 18.696 117.852 1.00 0.00

ATOM 7084 OG SER 37 44.425 18.994 118.283 1.00 0.00

ATOM 7085 HG SER 37 44.559 18.783 119.210 1.00 0.00

ATOM 7086 C SER 37 41.640 20.763 116.678 1.00 0.00

ATOM 7087 O SER 37 41.562 20.512 115.477 1.00 0.00

ATOM 7088 N SER 38 40.636 21.295 117.377 1.00 0.00

ATOM 7089 H SER 38 40.803 21.539 118.342 1.00 0.00

ATOM 7090 CA SER 38 39.332 21.574 116.809 1.00 0.00

ATOM 7091 HA SER 38 39.120 20.731 116.151 1.00 0.00

ATOM 7092 CB SER 38 38.258 21.669 117.889 1.00 0.00

ATOM 7093 HB1 SER 38 37.294 21.866 117.419 1.00 0.00

ATOM 7094 HB2 SER 38 38.414 22.569 118.484 1.00 0.00

ATOM 7095 OG SER 38 38.213 20.473 118.634 1.00 0.00

ATOM 7096 HG SER 38 37.396 20.364 119.127 1.00 0.00

ATOM 7097 C SER 38 39.277 22.764 115.862 1.00 0.00

ATOM 7098 O SER 38 38.318 22.881 115.102 1.00 0.00

ATOM 7099 N ASP 39 40.221 23.706 115.848 1.00 0.00

ATOM 7100 H ASP 39 41.069 23.596 116.385 1.00 0.00

ATOM 7101 CA ASP 39 40.231 24.789 114.883 1.00 0.00

ATOM 7102 HA ASP 39 39.219 25.119 114.650 1.00 0.00

ATOM 7103 CB ASP 39 40.882 26.035 115.471 1.00 0.00

ATOM 7104 HB1 ASP 39 40.630 25.920 116.524 1.00 0.00

ATOM 7105 HB2 ASP 39 41.947 25.806 115.510 1.00 0.00

ATOM 7106 CG ASP 39 40.518 27.401 114.903 1.00 0.00

ATOM 7107 OD1 ASP 39 40.405 28.305 115.761 1.00 0.00

ATOM 7108 OD2 ASP 39 40.457 27.578 113.669 1.00 0.00

ATOM 7109 C ASP 39 40.898 24.289 113.609 1.00 0.00

ATOM 7110 O ASP 39 40.318 24.431 112.534 1.00 0.00

ATOM 7111 N LEU 40 41.966 23.506 113.773 1.00 0.00

ATOM 7112 H LEU 40 42.264 23.367 114.728 1.00 0.00

ATOM 7113 CA LEU 40 42.646 22.828 112.687 1.00 0.00

ATOM 7114 HA LEU 40 42.877 23.652 112.013 1.00 0.00

ATOM 7115 CB LEU 40 43.958 22.172 113.110 1.00 0.00

ATOM 7116 HB1 LEU 40 44.454 21.672 112.287 1.00 0.00

ATOM 7117 HB2 LEU 40 43.733 21.358 113.801 1.00 0.00

ATOM 7118 CG LEU 40 45.031 23.101 113.671 1.00 0.00

ATOM 7119 HG LEU 40 44.576 23.620 114.515 1.00 0.00

ATOM 7120 CD1 LEU 40 46.224 22.245 114.083 1.00 0.00

ATOM 7121 1HD1 LEU 40 46.728 21.824 113.213 1.00 0.00

ATOM 7122 2HD1 LEU 40 45.999 21.392 114.724 1.00 0.00

ATOM 7123 3HD1 LEU 40 46.876 22.859 114.705 1.00 0.00

ATOM 7124 CD2 LEU 40 45.406 24.166 112.645 1.00 0.00

ATOM 7125 1HD2 LEU 40 44.550 24.828 112.514 1.00 0.00

ATOM 7126 2HD2 LEU 40 45.653 23.684 111.699 1.00 0.00

ATOM 7127 3HD2 LEU 40 46.256 24.734 113.026 1.00 0.00

ATOM 7128 C LEU 40 41.743 21.885 111.905 1.00 0.00

ATOM 7129 O LEU 40 41.929 21.674 110.709 1.00 0.00

ATOM 7130 N ARG 41 40.775 21.263 112.584 1.00 0.00

ATOM 7131 H ARG 41 40.706 21.499 113.563 1.00 0.00

ATOM 7132 CA ARG 41 39.759 20.328 112.144 1.00 0.00

ATOM 7133 HA ARG 41 40.325 19.535 111.657 1.00 0.00

ATOM 7134 CB ARG 41 38.899 19.723 113.251 1.00 0.00

ATOM 7135 HB1 ARG 41 38.512 20.535 113.867 1.00 0.00

ATOM 7136 HB2 ARG 41 39.534 19.147 113.923 1.00 0.00

ATOM 7137 CG ARG 41 37.761 18.799 112.826 1.00 0.00

ATOM 7138 HG1 ARG 41 37.207 19.137 111.951 1.00 0.00

ATOM 7139 HG2 ARG 41 38.223 17.833 112.632 1.00 0.00

ATOM 7140 CD ARG 41 36.869 18.622 114.052 1.00 0.00

ATOM 7141 HD1 ARG 41 36.202 17.763 113.970 1.00 0.00

ATOM 7142 HD2 ARG 41 37.553 18.390 114.868 1.00 0.00

ATOM 7143 NE ARG 41 35.998 19.780 114.254 1.00 0.00

ATOM 7144 HE ARG 41 35.623 20.130 113.383 1.00 0.00

ATOM 7145 CZ ARG 41 35.496 20.286 115.387 1.00 0.00

ATOM 7146 NH1 ARG 41 35.609 19.732 116.601 1.00 0.00

ATOM 7147 1HH1 ARG 41 36.291 18.988 116.648 1.00 0.00

ATOM 7148 2HH1 ARG 41 35.314 20.183 117.456 1.00 0.00

ATOM 7149 NH2 ARG 41 34.770 21.404 115.259 1.00 0.00

ATOM 7150 1HH2 ARG 41 34.920 22.014 114.467 1.00 0.00

ATOM 7151 2HH2 ARG 41 34.162 21.594 116.041 1.00 0.00

ATOM 7152 C ARG 41 38.847 20.889 111.062 1.00 0.00

ATOM 7153 O ARG 41 38.677 20.236 110.037 1.00 0.00

ATOM 7154 N ASN 42 38.414 22.143 111.212 1.00 0.00

ATOM 7155 H ASN 42 38.737 22.654 112.020 1.00 0.00

ATOM 7156 CA ASN 42 37.647 22.995 110.326 1.00 0.00

ATOM 7157 HA ASN 42 36.863 22.393 109.867 1.00 0.00

ATOM 7158 CB ASN 42 36.984 24.034 111.228 1.00 0.00

ATOM 7159 HB1 ASN 42 36.476 23.548 112.061 1.00 0.00

ATOM 7160 HB2 ASN 42 37.707 24.694 111.705 1.00 0.00

ATOM 7161 CG ASN 42 36.027 24.853 110.373 1.00 0.00

ATOM 7162 OD1 ASN 42 34.898 24.482 110.056 1.00 0.00

ATOM 7163 ND2 ASN 42 36.445 26.085 110.084 1.00 0.00

ATOM 7164 1HD2 ASN 42 37.370 26.336 110.402 1.00 0.00

ATOM 7165 2HD2 ASN 42 36.037 26.577 109.300 1.00 0.00

ATOM 7166 C ASN 42 38.561 23.460 109.201 1.00 0.00

ATOM 7167 O ASN 42 38.095 23.548 108.067 1.00 0.00

ATOM 7168 N LEU 43 39.809 23.826 109.501 1.00 0.00

ATOM 7169 H LEU 43 39.948 24.073 110.471 1.00 0.00

ATOM 7170 CA LEU 43 40.743 24.329 108.515 1.00 0.00

ATOM 7171 HA LEU 43 40.263 25.201 108.068 1.00 0.00

ATOM 7172 CB LEU 43 42.033 24.710 109.234 1.00 0.00

ATOM 7173 HB1 LEU 43 42.478 23.743 109.465 1.00 0.00

ATOM 7174 HB2 LEU 43 41.726 25.237 110.139 1.00 0.00

ATOM 7175 CG LEU 43 43.128 25.433 108.453 1.00 0.00

ATOM 7176 HG LEU 43 43.352 25.056 107.455 1.00 0.00

ATOM 7177 CD1 LEU 43 42.749 26.896 108.247 1.00 0.00

ATOM 7178 1HD1 LEU 43 41.759 26.943 107.792 1.00 0.00

ATOM 7179 2HD1 LEU 43 43.501 27.308 107.573 1.00 0.00

ATOM 7180 3HD1 LEU 43 42.579 27.373 109.212 1.00 0.00

ATOM 7181 CD2 LEU 43 44.375 25.319 109.325 1.00 0.00

ATOM 7182 1HD2 LEU 43 44.692 24.293 109.511 1.00 0.00

ATOM 7183 2HD2 LEU 43 44.104 25.794 110.269 1.00 0.00

ATOM 7184 3HD2 LEU 43 45.212 25.801 108.821 1.00 0.00

ATOM 7185 C LEU 43 40.960 23.262 107.452 1.00 0.00

ATOM 7186 O LEU 43 41.099 23.613 106.282 1.00 0.00

ATOM 7187 N LYS 44 40.894 21.989 107.853 1.00 0.00

ATOM 7188 H LYS 44 40.905 21.700 108.820 1.00 0.00

ATOM 7189 CA LYS 44 41.086 20.892 106.927 1.00 0.00

ATOM 7190 HA LYS 44 41.988 21.055 106.337 1.00 0.00

ATOM 7191 CB LYS 44 41.321 19.569 107.650 1.00 0.00

ATOM 7192 HB1 LYS 44 40.363 19.344 108.120 1.00 0.00

ATOM 7193 HB2 LYS 44 41.993 19.788 108.478 1.00 0.00

ATOM 7194 CG LYS 44 41.883 18.507 106.709 1.00 0.00

ATOM 7195 HG1 LYS 44 41.034 18.262 106.071 1.00 0.00

ATOM 7196 HG2 LYS 44 42.750 18.934 106.204 1.00 0.00

ATOM 7197 CD LYS 44 42.273 17.237 107.461 1.00 0.00

ATOM 7198 HD1 LYS 44 42.998 17.437 108.249 1.00 0.00

ATOM 7199 HD2 LYS 44 41.330 16.941 107.921 1.00 0.00

ATOM 7200 CE LYS 44 42.783 16.075 106.615 1.00 0.00

ATOM 7201 HE1 LYS 44 42.739 15.218 107.287 1.00 0.00

ATOM 7202 HE2 LYS 44 42.033 15.836 105.860 1.00 0.00

ATOM 7203 NZ LYS 44 44.201 16.207 106.244 1.00 0.00

ATOM 7204 HZ1 LYS 44 44.780 16.254 107.070 1.00 0.00

ATOM 7205 HZ2 LYS 44 44.316 16.996 105.624 1.00 0.00

ATOM 7206 HZ3 LYS 44 44.498 15.378 105.749 1.00 0.00

ATOM 7207 C LYS 44 40.057 20.809 105.809 1.00 0.00

ATOM 7208 O LYS 44 40.363 20.444 104.676 1.00 0.00

ATOM 7209 N THR 45 38.814 21.091 106.203 1.00 0.00

ATOM 7210 H THR 45 38.678 21.251 107.191 1.00 0.00

ATOM 7211 CA THR 45 37.633 21.087 105.364 1.00 0.00

ATOM 7212 HA THR 45 37.835 20.324 104.611 1.00 0.00

ATOM 7213 CB THR 45 36.437 20.610 106.184 1.00 0.00

ATOM 7214 HB THR 45 35.542 20.909 105.638 1.00 0.00

ATOM 7215 CG2 THR 45 36.437 19.093 106.344 1.00 0.00

ATOM 7216 1HG2 THR 45 37.302 18.834 106.955 1.00 0.00

ATOM 7217 2HG2 THR 45 36.448 18.576 105.384 1.00 0.00

ATOM 7218 3HG2 THR 45 35.511 18.805 106.842 1.00 0.00

ATOM 7219 OG1 THR 45 36.425 21.165 107.480 1.00 0.00

ATOM 7220 HG1 THR 45 35.542 21.542 107.473 1.00 0.00

ATOM 7221 C THR 45 37.385 22.369 104.582 1.00 0.00

ATOM 7222 O THR 45 36.821 22.280 103.494 1.00 0.00

ATOM 7223 N ALA 46 37.944 23.510 104.992 1.00 0.00

ATOM 7224 H ALA 46 38.317 23.537 105.929 1.00 0.00

ATOM 7225 CA ALA 46 37.688 24.829 104.448 1.00 0.00

ATOM 7226 HA ALA 46 36.806 24.739 103.814 1.00 0.00

ATOM 7227 CB ALA 46 37.382 25.753 105.622 1.00 0.00

ATOM 7228 HB1 ALA 46 36.742 25.367 106.417 1.00 0.00

ATOM 7229 HB2 ALA 46 37.104 26.734 105.236 1.00 0.00

ATOM 7230 HB3 ALA 46 38.351 25.896 106.099 1.00 0.00

ATOM 7231 C ALA 46 38.804 25.345 103.551 1.00 0.00

ATOM 7232 O ALA 46 38.523 25.997 102.548 1.00 0.00

ATOM 7233 N LEU 47 40.018 24.950 103.940 1.00 0.00

ATOM 7234 H LEU 47 40.120 24.388 104.774 1.00 0.00

ATOM 7235 CA LEU 47 41.197 25.576 103.378 1.00 0.00

ATOM 7236 HA LEU 47 40.823 26.341 102.697 1.00 0.00

ATOM 7237 CB LEU 47 42.040 26.341 104.394 1.00 0.00

ATOM 7238 HB1 LEU 47 43.090 26.109 104.233 1.00 0.00

ATOM 7239 HB2 LEU 47 41.851 26.068 105.433 1.00 0.00

ATOM 7240 CG LEU 47 41.996 27.861 104.266 1.00 0.00

ATOM 7241 HG LEU 47 42.695 28.175 105.040 1.00 0.00

ATOM 7242 CD1 LEU 47 42.568 28.388 102.953 1.00 0.00

ATOM 7243 1HD1 LEU 47 43.560 27.952 102.834 1.00 0.00

ATOM 7244 2HD1 LEU 47 42.812 29.438 103.111 1.00 0.00

ATOM 7245 3HD1 LEU 47 41.865 28.251 102.130 1.00 0.00

ATOM 7246 CD2 LEU 47 40.635 28.377 104.726 1.00 0.00

ATOM 7247 1HD2 LEU 47 40.654 29.427 105.018 1.00 0.00

ATOM 7248 2HD2 LEU 47 40.262 27.869 105.614 1.00 0.00

ATOM 7249 3HD2 LEU 47 39.883 28.205 103.957 1.00 0.00

ATOM 7250 C LEU 47 42.031 24.588 102.574 1.00 0.00

ATOM 7251 O LEU 47 42.300 24.809 101.397 1.00 0.00

ATOM 7252 N GLU 48 42.565 23.542 103.208 1.00 0.00

ATOM 7253 H GLU 48 42.302 23.432 104.177 1.00 0.00

ATOM 7254 CA GLU 48 43.078 22.333 102.596 1.00 0.00

ATOM 7255 HA GLU 48 44.028 22.575 102.121 1.00 0.00

ATOM 7256 CB GLU 48 43.495 21.310 103.650 1.00 0.00

ATOM 7257 HB1 GLU 48 42.740 20.920 104.332 1.00 0.00

ATOM 7258 HB2 GLU 48 44.274 21.728 104.287 1.00 0.00

ATOM 7259 CG GLU 48 44.157 20.094 103.011 1.00 0.00

ATOM 7260 HG1 GLU 48 43.388 19.523 102.489 1.00 0.00

ATOM 7261 HG2 GLU 48 44.981 20.446 102.390 1.00 0.00

ATOM 7262 CD GLU 48 44.836 19.190 104.030 1.00 0.00

ATOM 7263 OE1 GLU 48 44.363 18.045 104.193 1.00 0.00

ATOM 7264 OE2 GLU 48 45.784 19.615 104.724 1.00 0.00

ATOM 7265 C GLU 48 42.227 21.787 101.457 1.00 0.00

ATOM 7266 O GLU 48 42.763 21.261 100.484 1.00 0.00

ATOM 7267 N SER 49 40.897 21.891 101.515 1.00 0.00

ATOM 7268 H SER 49 40.510 22.417 102.284 1.00 0.00

ATOM 7269 CA SER 49 39.981 21.233 100.604 1.00 0.00

ATOM 7270 HA SER 49 40.420 20.242 100.484 1.00 0.00

ATOM 7271 CB SER 49 38.551 21.218 101.135 1.00 0.00

ATOM 7272 HB1 SER 49 38.425 20.536 101.976 1.00 0.00

ATOM 7273 HB2 SER 49 37.881 20.727 100.430 1.00 0.00

ATOM 7274 OG SER 49 38.191 22.560 101.378 1.00 0.00

ATOM 7275 HG SER 49 37.555 22.514 102.095 1.00 0.00

ATOM 7276 C SER 49 40.005 21.895 99.234 1.00 0.00

ATOM 7277 O SER 49 39.966 21.217 98.210 1.00 0.00

ATOM 7278 N ALA 50 40.210 23.214 99.222 1.00 0.00

ATOM 7279 H ALA 50 40.343 23.593 100.149 1.00 0.00

ATOM 7280 CA ALA 50 40.336 24.048 98.044 1.00 0.00

ATOM 7281 HA ALA 50 39.607 23.806 97.269 1.00 0.00

ATOM 7282 CB ALA 50 40.291 25.523 98.435 1.00 0.00

ATOM 7283 HB1 ALA 50 40.347 26.128 97.530 1.00 0.00

ATOM 7284 HB2 ALA 50 39.369 25.738 98.976 1.00 0.00

ATOM 7285 HB3 ALA 50 41.198 25.768 98.986 1.00 0.00

ATOM 7286 C ALA 50 41.705 23.847 97.415 1.00 0.00

ATOM 7287 O ALA 50 41.684 23.747 96.190 1.00 0.00

ATOM 7288 N PHE 51 42.769 23.658 98.200 1.00 0.00

ATOM 7289 H PHE 51 42.716 23.885 99.183 1.00 0.00

ATOM 7290 CA PHE 51 44.086 23.348 97.682 1.00 0.00

ATOM 7291 HA PHE 51 44.511 24.013 96.931 1.00 0.00

ATOM 7292 CB PHE 51 45.092 23.375 98.829 1.00 0.00

ATOM 7293 HB1 PHE 51 44.755 22.761 99.664 1.00 0.00

ATOM 7294 HB2 PHE 51 45.144 24.396 99.209 1.00 0.00

ATOM 7295 CG PHE 51 46.529 23.061 98.488 1.00 0.00

ATOM 7296 CD1 PHE 51 47.412 24.147 98.515 1.00 0.00

ATOM 7297 HD1 PHE 51 47.042 25.130 98.768 1.00 0.00

ATOM 7298 CE1 PHE 51 48.773 23.971 98.237 1.00 0.00

ATOM 7299 HE1 PHE 51 49.445 24.811 98.324 1.00 0.00

ATOM 7300 CZ PHE 51 49.250 22.683 97.967 1.00 0.00

ATOM 7301 HZ PHE 51 50.307 22.530 97.809 1.00 0.00

ATOM 7302 CE2 PHE 51 48.384 21.583 97.987 1.00 0.00

ATOM 7303 HE2 PHE 51 48.820 20.602 97.877 1.00 0.00

ATOM 7304 CD2 PHE 51 47.011 21.785 98.176 1.00 0.00

ATOM 7305 HD2 PHE 51 46.310 20.963 98.165 1.00 0.00

ATOM 7306 C PHE 51 44.020 21.976 97.025 1.00 0.00

ATOM 7307 O PHE 51 44.674 21.799 96.000 1.00 0.00

ATOM 7308 N ALA 52 43.342 21.028 97.676 1.00 0.00

ATOM 7309 H ALA 52 42.890 21.284 98.542 1.00 0.00

ATOM 7310 CA ALA 52 43.243 19.653 97.230 1.00 0.00

ATOM 7311 HA ALA 52 44.263 19.329 97.017 1.00 0.00

ATOM 7312 CB ALA 52 42.800 18.792 98.409 1.00 0.00

ATOM 7313 HB1 ALA 52 42.440 17.835 98.033 1.00 0.00

ATOM 7314 HB2 ALA 52 43.740 18.470 98.858 1.00 0.00

ATOM 7315 HB3 ALA 52 42.058 19.248 99.063 1.00 0.00

ATOM 7316 C ALA 52 42.310 19.394 96.054 1.00 0.00

ATOM 7317 O ALA 52 42.539 18.460 95.289 1.00 0.00

ATOM 7318 N ASP 53 41.408 20.286 95.640 1.00 0.00

ATOM 7319 H ASP 53 41.229 21.085 96.230 1.00 0.00

ATOM 7320 CA ASP 53 40.866 20.440 94.304 1.00 0.00

ATOM 7321 HA ASP 53 40.820 19.462 93.824 1.00 0.00

ATOM 7322 CB ASP 53 39.570 21.230 94.472 1.00 0.00

ATOM 7323 HB1 ASP 53 38.910 20.678 95.141 1.00 0.00

ATOM 7324 HB2 ASP 53 39.728 22.213 94.916 1.00 0.00

ATOM 7325 CG ASP 53 38.773 21.316 93.178 1.00 0.00

ATOM 7326 OD1 ASP 53 37.868 20.485 92.951 1.00 0.00

ATOM 7327 OD2 ASP 53 38.974 22.242 92.363 1.00 0.00

ATOM 7328 C ASP 53 41.836 21.076 93.320 1.00 0.00

ATOM 7329 O ASP 53 41.832 20.687 92.154 1.00 0.00

ATOM 7330 N ASP 54 42.675 22.051 93.676 1.00 0.00

ATOM 7331 H ASP 54 42.779 22.295 94.651 1.00 0.00

ATOM 7332 CA ASP 54 43.430 22.800 92.692 1.00 0.00

ATOM 7333 HA ASP 54 42.810 22.949 91.807 1.00 0.00

ATOM 7334 CB ASP 54 43.640 24.213 93.227 1.00 0.00

ATOM 7335 HB1 ASP 54 42.784 24.863 93.049 1.00 0.00

ATOM 7336 HB2 ASP 54 43.809 24.278 94.302 1.00 0.00

ATOM 7337 CG ASP 54 44.844 24.812 92.515 1.00 0.00

ATOM 7338 OD1 ASP 54 44.603 25.164 91.340 1.00 0.00

ATOM 7339 OD2 ASP 54 45.865 25.047 93.199 1.00 0.00

ATOM 7340 C ASP 54 44.704 22.024 92.388 1.00 0.00

ATOM 7341 O ASP 54 45.129 22.001 91.235 1.00 0.00

ATOM 7342 N GLN 55 45.332 21.351 93.355 1.00 0.00

ATOM 7343 H GLN 55 44.968 21.426 94.294 1.00 0.00

ATOM 7344 CA GLN 55 46.518 20.552 93.126 1.00 0.00

ATOM 7345 HA GLN 55 46.970 20.898 92.196 1.00 0.00

ATOM 7346 CB GLN 55 47.489 20.715 94.291 1.00 0.00

ATOM 7347 HB1 GLN 55 48.328 20.026 94.192 1.00 0.00

ATOM 7348 HB2 GLN 55 47.005 20.506 95.245 1.00 0.00

ATOM 7349 CG GLN 55 48.034 22.139 94.357 1.00 0.00

ATOM 7350 HG1 GLN 55 47.172 22.765 94.588 1.00 0.00

ATOM 7351 HG2 GLN 55 48.763 22.113 95.168 1.00 0.00

ATOM 7352 CD GLN 55 48.678 22.651 93.076 1.00 0.00

ATOM 7353 OE1 GLN 55 49.643 22.142 92.512 1.00 0.00

ATOM 7354 NE2 GLN 55 47.926 23.533 92.416 1.00 0.00

ATOM 7355 1HE2 GLN 55 47.189 23.999 92.927 1.00 0.00

ATOM 7356 2HE2 GLN 55 48.137 23.763 91.456 1.00 0.00

ATOM 7357 C GLN 55 46.164 19.111 92.787 1.00 0.00

ATOM 7358 O GLN 55 47.088 18.355 92.497 1.00 0.00

ATOM 7359 N THR 56 44.904 18.673 92.878 1.00 0.00

ATOM 7360 H THR 56 44.137 19.292 93.098 1.00 0.00

ATOM 7361 CA THR 56 44.393 17.319 92.939 1.00 0.00

ATOM 7362 HA THR 56 43.309 17.377 92.834 1.00 0.00

ATOM 7363 CB THR 56 44.878 16.507 91.740 1.00 0.00

ATOM 7364 HB THR 56 45.823 16.021 91.982 1.00 0.00

ATOM 7365 CG2 THR 56 43.893 15.457 91.235 1.00 0.00

ATOM 7366 1HG2 THR 56 43.717 14.750 92.044 1.00 0.00

ATOM 7367 2HG2 THR 56 44.396 14.884 90.456 1.00 0.00

ATOM 7368 3HG2 THR 56 42.860 15.770 91.077 1.00 0.00

ATOM 7369 OG1 THR 56 44.985 17.226 90.540 1.00 0.00

ATOM 7370 HG1 THR 56 44.178 17.734 90.590 1.00 0.00

ATOM 7371 C THR 56 44.600 16.530 94.224 1.00 0.00

ATOM 7372 O THR 56 43.768 15.655 94.445 1.00 0.00

ATOM 7373 N TYR 57 45.568 16.898 95.066 1.00 0.00

ATOM 7374 H TYR 57 46.104 17.719 94.825 1.00 0.00

ATOM 7375 CA TYR 57 45.951 16.218 96.287 1.00 0.00

ATOM 7376 HA TYR 57 45.088 15.661 96.650 1.00 0.00

ATOM 7377 CB TYR 57 47.135 15.309 95.971 1.00 0.00

ATOM 7378 HB1 TYR 57 47.554 14.818 96.850 1.00 0.00

ATOM 7379 HB2 TYR 57 47.927 15.916 95.539 1.00 0.00

ATOM 7380 CG TYR 57 46.707 14.347 94.890 1.00 0.00

ATOM 7381 CD1 TYR 57 47.112 14.623 93.579 1.00 0.00

ATOM 7382 HD1 TYR 57 47.631 15.529 93.396 1.00 0.00

ATOM 7383 CE1 TYR 57 46.917 13.696 92.549 1.00 0.00

ATOM 7384 HE1 TYR 57 47.263 13.951 91.558 1.00 0.00

ATOM 7385 CZ TYR 57 46.167 12.536 92.829 1.00 0.00

ATOM 7386 OH TYR 57 45.799 11.703 91.813 1.00 0.00

ATOM 7387 HH TYR 57 45.193 10.992 92.034 1.00 0.00

ATOM 7388 CE2 TYR 57 45.774 12.273 94.156 1.00 0.00

ATOM 7389 HE2 TYR 57 45.251 11.363 94.409 1.00 0.00

ATOM 7390 CD2 TYR 57 46.109 13.124 95.216 1.00 0.00

ATOM 7391 HD2 TYR 57 45.747 12.827 96.190 1.00 0.00

ATOM 7392 C TYR 57 46.382 17.247 97.324 1.00 0.00

ATOM 7393 O TYR 57 47.035 18.259 97.090 1.00 0.00

ATOM 7394 N PRO 58 46.083 17.027 98.605 1.00 0.00

ATOM 7395 CD PRO 58 45.301 15.896 99.063 1.00 0.00

ATOM 7396 HD1 PRO 58 45.719 14.908 98.874 1.00 0.00

ATOM 7397 HD2 PRO 58 44.258 15.914 98.780 1.00 0.00

ATOM 7398 CG PRO 58 45.310 15.839 100.587 1.00 0.00

ATOM 7399 HG1 PRO 58 46.255 15.393 100.898 1.00 0.00

ATOM 7400 HG2 PRO 58 44.411 15.414 101.032 1.00 0.00

ATOM 7401 CB PRO 58 45.508 17.327 100.859 1.00 0.00

ATOM 7402 HB1 PRO 58 45.893 17.551 101.854 1.00 0.00

ATOM 7403 HB2 PRO 58 44.539 17.787 100.663 1.00 0.00

ATOM 7404 CA PRO 58 46.403 17.863 99.745 1.00 0.00

ATOM 7405 HA PRO 58 46.191 18.918 99.568 1.00 0.00

ATOM 7406 C PRO 58 47.889 17.769 100.064 1.00 0.00

ATOM 7407 O PRO 58 48.595 16.900 99.558 1.00 0.00

ATOM 7408 N PRO 59 48.463 18.588 100.947 1.00 0.00

ATOM 7409 CD PRO 59 47.848 19.774 101.509 1.00 0.00

ATOM 7410 HD1 PRO 59 47.511 19.606 102.532 1.00 0.00

ATOM 7411 HD2 PRO 59 47.084 20.213 100.869 1.00 0.00

ATOM 7412 CG PRO 59 48.897 20.875 101.627 1.00 0.00

ATOM 7413 HG1 PRO 59 48.669 21.561 102.443 1.00 0.00

ATOM 7414 HG2 PRO 59 48.926 21.411 100.679 1.00 0.00

ATOM 7415 CB PRO 59 50.132 20.000 101.825 1.00 0.00

ATOM 7416 HB1 PRO 59 50.165 19.699 102.872 1.00 0.00

ATOM 7417 HB2 PRO 59 51.042 20.561 101.620 1.00 0.00

ATOM 7418 CA PRO 59 49.903 18.741 100.992 1.00 0.00

ATOM 7419 HA PRO 59 50.218 18.954 99.971 1.00 0.00

ATOM 7420 C PRO 59 50.732 17.558 101.474 1.00 0.00

ATOM 7421 O PRO 59 51.795 17.202 100.972 1.00 0.00

ATOM 7422 N GLU 60 50.285 16.900 102.545 1.00 0.00

ATOM 7423 H GLU 60 49.387 17.163 102.922 1.00 0.00

ATOM 7424 CA GLU 60 50.919 15.802 103.248 1.00 0.00

ATOM 7425 HA GLU 60 50.530 15.716 104.263 1.00 0.00

ATOM 7426 CB GLU 60 50.558 14.472 102.594 1.00 0.00

ATOM 7427 HB1 GLU 60 50.981 13.703 103.241 1.00 0.00

ATOM 7428 HB2 GLU 60 50.980 14.284 101.607 1.00 0.00

ATOM 7429 CG GLU 60 49.058 14.199 102.533 1.00 0.00

ATOM 7430 HG1 GLU 60 48.568 14.762 101.739 1.00 0.00

ATOM 7431 HG2 GLU 60 48.940 13.135 102.330 1.00 0.00

ATOM 7432 CD GLU 60 48.288 14.406 103.831 1.00 0.00

ATOM 7433 OE1 GLU 60 48.416 13.671 104.833 1.00 0.00

ATOM 7434 OE2 GLU 60 47.384 15.261 103.937 1.00 0.00

ATOM 7435 C GLU 60 52.420 15.923 103.477 1.00 0.00

ATOM 7436 O GLU 60 53.156 15.054 103.016 1.00 0.00

ATOM 7437 N SER 61 52.928 17.037 104.009 1.00 0.00

ATOM 7438 H SER 61 52.228 17.677 104.354 1.00 0.00

ATOM 7439 CA SER 61 54.222 17.499 104.471 1.00 0.00

ATOM 7440 HA SER 61 54.626 16.664 105.044 1.00 0.00

ATOM 7441 CB SER 61 55.078 17.887 103.270 1.00 0.00

ATOM 7442 HB1 SER 61 54.561 18.534 102.561 1.00 0.00

ATOM 7443 HB2 SER 61 55.932 18.459 103.631 1.00 0.00

ATOM 7444 OG SER 61 55.411 16.623 102.745 1.00 0.00

ATOM 7445 HG SER 61 54.640 16.102 102.985 1.00 0.00

ATOM 7446 C SER 61 54.112 18.695 105.405 1.00 0.00

ATOM 7447 OC1 SER 61 53.003 19.273 105.441 1.00 0.00

ATOM 7448 OC2 SER 61 55.003 18.813 106.273 1.00 0.00

ATOM 7449 N PHE 1 30.851 36.559 175.023 1.00 0.00

ATOM 7450 H1 PHE 1 30.451 35.673 175.299 1.00 0.00

ATOM 7451 H2 PHE 1 30.075 37.093 174.658 1.00 0.00

ATOM 7452 H3 PHE 1 31.445 36.421 174.218 1.00 0.00

ATOM 7453 CA PHE 1 31.642 37.190 176.089 1.00 0.00

ATOM 7454 HA PHE 1 32.069 38.056 175.584 1.00 0.00

ATOM 7455 CB PHE 1 30.785 37.790 177.201 1.00 0.00

ATOM 7456 HB1 PHE 1 30.342 38.743 176.912 1.00 0.00

ATOM 7457 HB2 PHE 1 31.411 37.957 178.077 1.00 0.00

ATOM 7458 CG PHE 1 29.545 36.995 177.536 1.00 0.00

ATOM 7459 CD1 PHE 1 28.323 37.332 176.941 1.00 0.00

ATOM 7460 HD1 PHE 1 28.213 38.183 176.285 1.00 0.00

ATOM 7461 CE1 PHE 1 27.197 36.586 177.311 1.00 0.00

ATOM 7462 HE1 PHE 1 26.226 36.812 176.898 1.00 0.00

ATOM 7463 CZ PHE 1 27.259 35.491 178.181 1.00 0.00

ATOM 7464 HZ PHE 1 26.381 34.958 178.515 1.00 0.00

ATOM 7465 CE2 PHE 1 28.532 35.111 178.622 1.00 0.00

ATOM 7466 HE2 PHE 1 28.651 34.262 179.278 1.00 0.00

ATOM 7467 CD2 PHE 1 29.676 35.879 178.368 1.00 0.00

ATOM 7468 HD2 PHE 1 30.649 35.727 178.813 1.00 0.00

ATOM 7469 C PHE 1 32.759 36.259 176.540 1.00 0.00

ATOM 7470 O PHE 1 32.841 35.890 177.709 1.00 0.00

ATOM 7471 N THR 2 33.687 35.943 175.634 1.00 0.00

ATOM 7472 H THR 2 33.693 36.408 174.737 1.00 0.00

ATOM 7473 CA THR 2 34.883 35.159 175.869 1.00 0.00

ATOM 7474 HA THR 2 35.159 35.195 176.923 1.00 0.00

ATOM 7475 CB THR 2 34.569 33.698 175.560 1.00 0.00

ATOM 7476 HB THR 2 33.896 33.551 174.715 1.00 0.00

ATOM 7477 CG2 THR 2 35.815 32.829 175.417 1.00 0.00

ATOM 7478 1HG2 THR 2 35.599 31.763 175.478 1.00 0.00

ATOM 7479 2HG2 THR 2 36.399 32.997 176.322 1.00 0.00

ATOM 7480 3HG2 THR 2 36.367 33.078 174.510 1.00 0.00

ATOM 7481 OG1 THR 2 33.934 33.142 176.689 1.00 0.00

ATOM 7482 HG1 THR 2 33.320 32.506 176.314 1.00 0.00

ATOM 7483 C THR 2 36.112 35.609 175.093 1.00 0.00

ATOM 7484 O THR 2 36.026 35.726 173.873 1.00 0.00

ATOM 7485 N LEU 3 37.232 35.841 175.782 1.00 0.00

ATOM 7486 H LEU 3 37.221 35.761 176.789 1.00 0.00

ATOM 7487 CA LEU 3 38.398 36.484 175.210 1.00 0.00

ATOM 7488 HA LEU 3 38.028 37.450 174.866 1.00 0.00

ATOM 7489 CB LEU 3 39.401 36.673 176.344 1.00 0.00

ATOM 7490 HB1 LEU 3 39.894 35.707 176.452 1.00 0.00

ATOM 7491 HB2 LEU 3 38.790 36.914 177.214 1.00 0.00

ATOM 7492 CG LEU 3 40.386 37.812 176.094 1.00 0.00

ATOM 7493 HG LEU 3 40.883 37.550 175.159 1.00 0.00

ATOM 7494 CD1 LEU 3 39.895 39.256 176.063 1.00 0.00

ATOM 7495 1HD1 LEU 3 39.492 39.420 175.065 1.00 0.00

ATOM 7496 2HD1 LEU 3 40.762 39.916 176.058 1.00 0.00

ATOM 7497 3HD1 LEU 3 39.161 39.515 176.827 1.00 0.00

ATOM 7498 CD2 LEU 3 41.364 37.691 177.259 1.00 0.00

ATOM 7499 1HD2 LEU 3 41.874 36.728 177.211 1.00 0.00

ATOM 7500 2HD2 LEU 3 40.903 37.766 178.243 1.00 0.00

ATOM 7501 3HD2 LEU 3 42.094 38.500 177.259 1.00 0.00

ATOM 7502 C LEU 3 39.072 35.759 174.055 1.00 0.00

ATOM 7503 O LEU 3 39.357 36.385 173.036 1.00 0.00

ATOM 7504 N ILE 4 39.071 34.428 174.151 1.00 0.00

ATOM 7505 H ILE 4 38.978 34.002 175.062 1.00 0.00

ATOM 7506 CA ILE 4 39.584 33.689 173.014 1.00 0.00

ATOM 7507 HA ILE 4 40.560 34.070 172.715 1.00 0.00

ATOM 7508 CB ILE 4 39.889 32.269 173.479 1.00 0.00

ATOM 7509 HB ILE 4 40.666 32.329 174.242 1.00 0.00

ATOM 7510 CG2 ILE 4 38.664 31.534 174.017 1.00 0.00

ATOM 7511 1HG2 ILE 4 38.428 31.868 175.027 1.00 0.00

ATOM 7512 2HG2 ILE 4 38.810 30.454 174.015 1.00 0.00

ATOM 7513 3HG2 ILE 4 37.812 31.689 173.356 1.00 0.00

ATOM 7514 CG1 ILE 4 40.464 31.356 172.399 1.00 0.00

ATOM 7515 1HG1 ILE 4 40.418 30.308 172.695 1.00 0.00

ATOM 7516 2HG1 ILE 4 39.782 31.396 171.550 1.00 0.00

ATOM 7517 CD ILE 4 41.876 31.736 171.959 1.00 0.00

ATOM 7518 HD1 ILE 4 42.061 32.759 171.632 1.00 0.00

ATOM 7519 HD2 ILE 4 42.311 31.101 171.188 1.00 0.00

ATOM 7520 HD3 ILE 4 42.507 31.636 172.841 1.00 0.00

ATOM 7521 C ILE 4 38.746 33.806 171.748 1.00 0.00

ATOM 7522 O ILE 4 39.290 33.731 170.648 1.00 0.00

ATOM 7523 N GLU 5 37.466 34.137 171.934 1.00 0.00

ATOM 7524 H GLU 5 37.171 34.397 172.864 1.00 0.00

ATOM 7525 CA GLU 5 36.460 34.327 170.908 1.00 0.00

ATOM 7526 HA GLU 5 36.567 33.636 170.073 1.00 0.00

ATOM 7527 CB GLU 5 35.029 34.050 171.364 1.00 0.00

ATOM 7528 HB1 GLU 5 35.040 33.820 172.428 1.00 0.00

ATOM 7529 HB2 GLU 5 34.770 33.107 170.883 1.00 0.00

ATOM 7530 CG GLU 5 33.815 34.932 171.086 1.00 0.00

ATOM 7531 HG1 GLU 5 34.259 35.925 171.060 1.00 0.00

ATOM 7532 HG2 GLU 5 33.476 34.804 170.057 1.00 0.00

ATOM 7533 CD GLU 5 32.759 34.895 172.183 1.00 0.00

ATOM 7534 OE1 GLU 5 32.217 33.807 172.473 1.00 0.00

ATOM 7535 OE2 GLU 5 32.397 35.949 172.748 1.00 0.00

ATOM 7536 C GLU 5 36.589 35.678 170.221 1.00 0.00

ATOM 7537 O GLU 5 36.179 35.830 169.072 1.00 0.00

ATOM 7538 N LEU 6 37.279 36.652 170.820 1.00 0.00

ATOM 7539 H LEU 6 37.626 36.458 171.748 1.00 0.00

ATOM 7540 CA LEU 6 37.634 37.963 170.316 1.00 0.00

ATOM 7541 HA LEU 6 36.977 38.230 169.489 1.00 0.00

ATOM 7542 CB LEU 6 37.327 38.950 171.437 1.00 0.00

ATOM 7543 HB1 LEU 6 37.583 39.977 171.171 1.00 0.00

ATOM 7544 HB2 LEU 6 37.956 38.565 172.239 1.00 0.00

ATOM 7545 CG LEU 6 35.939 38.918 172.072 1.00 0.00

ATOM 7546 HG LEU 6 35.527 37.949 172.353 1.00 0.00

ATOM 7547 CD1 LEU 6 35.912 39.746 173.353 1.00 0.00

ATOM 7548 1HD1 LEU 6 34.893 40.103 173.496 1.00 0.00

ATOM 7549 2HD1 LEU 6 36.561 40.616 173.248 1.00 0.00

ATOM 7550 3HD1 LEU 6 36.245 39.137 174.194 1.00 0.00

ATOM 7551 CD2 LEU 6 34.905 39.433 171.076 1.00 0.00

ATOM 7552 1HD2 LEU 6 33.976 39.696 171.583 1.00 0.00

ATOM 7553 2HD2 LEU 6 34.632 38.664 170.353 1.00 0.00

ATOM 7554 3HD2 LEU 6 35.359 40.229 170.486 1.00 0.00

ATOM 7555 C LEU 6 39.070 38.040 169.813 1.00 0.00

ATOM 7556 O LEU 6 39.355 38.724 168.833 1.00 0.00

ATOM 7557 N LEU 7 39.896 37.135 170.343 1.00 0.00

ATOM 7558 H LEU 7 39.487 36.552 171.060 1.00 0.00

ATOM 7559 CA LEU 7 41.311 37.183 170.038 1.00 0.00

ATOM 7560 HA LEU 7 41.599 38.234 170.036 1.00 0.00

ATOM 7561 CB LEU 7 42.019 36.587 171.251 1.00 0.00

ATOM 7562 HB1 LEU 7 41.687 35.556 171.376 1.00 0.00

ATOM 7563 HB2 LEU 7 41.619 37.201 172.058 1.00 0.00

ATOM 7564 CG LEU 7 43.537 36.560 171.401 1.00 0.00

ATOM 7565 HG LEU 7 43.893 36.029 172.284 1.00 0.00

ATOM 7566 CD1 LEU 7 44.175 35.801 170.241 1.00 0.00

ATOM 7567 1HD1 LEU 7 43.875 36.217 169.279 1.00 0.00

ATOM 7568 2HD1 LEU 7 43.780 34.797 170.393 1.00 0.00

ATOM 7569 3HD1 LEU 7 45.265 35.820 170.249 1.00 0.00

ATOM 7570 CD2 LEU 7 43.931 38.034 171.409 1.00 0.00

ATOM 7571 1HD2 LEU 7 44.979 38.053 171.705 1.00 0.00

ATOM 7572 2HD2 LEU 7 43.380 38.672 172.101 1.00 0.00

ATOM 7573 3HD2 LEU 7 43.812 38.338 170.369 1.00 0.00

ATOM 7574 C LEU 7 41.534 36.557 168.669 1.00 0.00

ATOM 7575 O LEU 7 42.164 37.145 167.792 1.00 0.00

ATOM 7576 N ILE 8 40.982 35.377 168.378 1.00 0.00

ATOM 7577 H ILE 8 40.467 34.900 169.104 1.00 0.00

ATOM 7578 CA ILE 8 41.151 34.656 167.132 1.00 0.00

ATOM 7579 HA ILE 8 42.205 34.405 167.016 1.00 0.00

ATOM 7580 CB ILE 8 40.292 33.398 167.223 1.00 0.00

ATOM 7581 HB ILE 8 39.431 33.643 167.845 1.00 0.00

ATOM 7582 CG2 ILE 8 39.695 32.812 165.947 1.00 0.00

ATOM 7583 1HG2 ILE 8 39.314 31.807 166.132 1.00 0.00

ATOM 7584 2HG2 ILE 8 40.426 32.856 165.139 1.00 0.00

ATOM 7585 3HG2 ILE 8 38.889 33.438 165.563 1.00 0.00

ATOM 7586 CG1 ILE 8 41.249 32.349 167.783 1.00 0.00

ATOM 7587 1HG1 ILE 8 41.972 32.111 167.003 1.00 0.00

ATOM 7588 2HG1 ILE 8 41.877 32.743 168.582 1.00 0.00

ATOM 7589 CD ILE 8 40.642 31.057 168.319 1.00 0.00

ATOM 7590 HD1 ILE 8 39.680 31.310 168.764 1.00 0.00

ATOM 7591 HD2 ILE 8 41.402 30.581 168.941 1.00 0.00

ATOM 7592 HD3 ILE 8 40.323 30.537 167.416 1.00 0.00

ATOM 7593 C ILE 8 40.829 35.546 165.939 1.00 0.00

ATOM 7594 O ILE 8 41.570 35.581 164.960 1.00 0.00

ATOM 7595 N VAL 9 39.783 36.374 165.963 1.00 0.00

ATOM 7596 H VAL 9 39.344 36.472 166.866 1.00 0.00

ATOM 7597 CA VAL 9 39.358 37.115 164.790 1.00 0.00

ATOM 7598 HA VAL 9 39.325 36.378 163.988 1.00 0.00

ATOM 7599 CB VAL 9 38.032 37.870 164.841 1.00 0.00

ATOM 7600 HB VAL 9 38.036 38.651 164.081 1.00 0.00

ATOM 7601 CG1 VAL 9 36.857 36.938 164.560 1.00 0.00

ATOM 7602 1HG1 VAL 9 36.927 36.074 165.221 1.00 0.00

ATOM 7603 2HG1 VAL 9 36.971 36.619 163.524 1.00 0.00

ATOM 7604 3HG1 VAL 9 35.912 37.473 164.651 1.00 0.00

ATOM 7605 CG2 VAL 9 37.825 38.485 166.222 1.00 0.00

ATOM 7606 1HG2 VAL 9 36.860 38.968 166.378 1.00 0.00

ATOM 7607 2HG2 VAL 9 38.660 39.163 166.395 1.00 0.00

ATOM 7608 3HG2 VAL 9 38.016 37.712 166.967 1.00 0.00

ATOM 7609 C VAL 9 40.384 38.144 164.338 1.00 0.00

ATOM 7610 O VAL 9 40.313 38.550 163.180 1.00 0.00

ATOM 7611 N VAL 10 41.284 38.552 165.236 1.00 0.00

ATOM 7612 H VAL 10 41.305 38.153 166.163 1.00 0.00

ATOM 7613 CA VAL 10 42.468 39.331 164.924 1.00 0.00

ATOM 7614 HA VAL 10 42.293 39.914 164.021 1.00 0.00

ATOM 7615 CB VAL 10 42.822 40.388 165.967 1.00 0.00

ATOM 7616 HB VAL 10 43.378 39.879 166.753 1.00 0.00

ATOM 7617 CG1 VAL 10 43.598 41.627 165.528 1.00 0.00

ATOM 7618 1HG1 VAL 10 43.717 42.305 166.373 1.00 0.00

ATOM 7619 2HG1 VAL 10 42.948 42.117 164.803 1.00 0.00

ATOM 7620 3HG1 VAL 10 44.638 41.487 165.234 1.00 0.00

ATOM 7621 CG2 VAL 10 41.564 40.981 166.594 1.00 0.00

ATOM 7622 1HG2 VAL 10 41.703 41.860 167.222 1.00 0.00

ATOM 7623 2HG2 VAL 10 41.044 40.291 167.259 1.00 0.00

ATOM 7624 3HG2 VAL 10 40.997 41.218 165.694 1.00 0.00

ATOM 7625 C VAL 10 43.743 38.535 164.692 1.00 0.00

ATOM 7626 O VAL 10 44.465 38.808 163.741 1.00 0.00

ATOM 7627 N ALA 11 43.958 37.432 165.412 1.00 0.00

ATOM 7628 H ALA 11 43.251 37.266 166.114 1.00 0.00

ATOM 7629 CA ALA 11 45.102 36.569 165.193 1.00 0.00

ATOM 7630 HA ALA 11 46.000 37.182 165.115 1.00 0.00

ATOM 7631 CB ALA 11 45.226 35.647 166.403 1.00 0.00
[truncated: 486,351 more chars]
